# Dual Regulation of Molecular Rigidity and Orbital Engineering of Pt(II) Emitters for High‐Performance Deep‐Blue OLEDs

**DOI:** 10.1002/advs.202509722

**Published:** 2025-10-03

**Authors:** Chengyao Zhang, Kewei Xu, Yun‐Fang Yang, Yuanbin She, Guijie Li

**Affiliations:** ^1^ Center for Phosphorescent Material Research State Key Laboratory of Green Chemical Synthesis and Conversion College of Chemical Engineering Zhejiang University of Technology Hangzhou Zhejiang 310014 P. R. China

**Keywords:** deep‐blue OLED, F‐containing, high color purity, high luminancenarrow emission, tetradentate platinum(II) emitter

## Abstract

Blue phosphorescent organic light‐emitting diodes (PhOLEDs) face critical challenges in terms of low color purity and severe efficiency roll‐off. In this study, four novel tetradentate Pt(II) emitters (PtCY, PtCY‐F, PtCY‐*t*Bu, PtCY‐*t*BuF) are designed through a synergistic strategy of molecular orbital engineering and steric hindrance effect. By introducing a bulky diisopropylbiphenyl (diPrPh) group at the *N*‐heterocyclic carbene (NHC) moiety to increase molecular rigidity and suppress intermolecular interactions, the Pt(II) emitters achieve narrowband deep‐blue emission (452–457 nm) in dichloromethane, with full width at half maximum (FWHM) values of 18–23 nm. Combined with the introduction of fluorine atoms (─F) away from the lowest unoccupied molecular orbital (LUMO) distribution, the external quantum efficiency (EQE) is improved while avoiding the breakage of the C─F bond. PtCY‐*t*BuF‐based device B4 achieved a high maximum luminance of 45621 cd m^−2^, and record high EQEs of 27.1%, 24.3%, and 21.7% at high brightness levels of 1000, 5000, and 10 000 cd m^−^^2^ respectively and lowest efficiency roll‐off of 3.2% at 1000 cd m^−2^, among reported Pt(II)‐based deep‐blue OLEDs with CIE_y_ < 0.15. This study provides a novel strategy for the development of highly efficient tetradentate Pt(II) emitters with high color purity for high‐performance deep‐blue PhOLED applications.

## Graphical abstract

Four novel tetradentate Pt(II) emitters (PtCY, PtCY‐F, PtCY‐*t*Bu, PtCY‐*t*BuF) are designed through synergistic strategy of molecular orbital engineering and steric hindrance effect. PtCY‐tBuF‐based device B4 achieves a high maximum luminance of 45 621 cd m^−2^, and record high external quantum efficiency (EQE) of 27.1%, 24.3%, and 21.7% at high brightness levels of 1000, 5000, and 10 000 cd m^−^^2^ respectively and lowest efficiency roll‐off of 3.2% at 1000 cd m^−2^, among reported Pt(II)‐based deep‐blue OLEDs with CIE_y_ < 0.15.

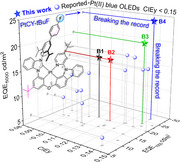

## 1 Introduction

Since the pioneering research of Tang and Vanslyke in 1987^[^ ^1^ ^]^ organic light‐emitting diodes (OLEDs) have been regarded as an emerging application technology for the next generation of displays due to their low energy consumption, high color purity, self‐luminescence, ultrathinness, flexibility and foldability.^[^ ^2^ ^]^ Among the three primary colors of red, green, and blue required for full‐color displays, red and green phosphorescent OLEDs (PhOLEDs) have reached commercialization standards, but blue PhOLEDs still face significant challenges in terms of device efficiency, color purity, and operational stability.^[^ ^3^, ^4^, ^5^, ^6^, ^7^, ^8^ ^]^ In particular, the latest BT.2020 ultra‐high definition (UHD) display standard defines the blue color coordinates as (0.131, 0.046), imposing stricter requirements on the color saturation of blue‐emitting materials.^[^ ^9^ ^]^ Therefore, the design and development of high‐efficiency narrowband deep‐blue phosphorescent materials with a CIE_y_ < 0.15 are crucial for high‐performance OLED technology.

Phosphorescent Ir(III) and Pt(II) complexes can efficiently harvest both electrogenerated singlet and triplet excitons due to their strong spin–orbit coupling (SOC), thereby enabling internal quantum efficiencies (IQEs) approaching 100%, making them highly promising luminescent dopants in the PhOLEDs.^[^ ^10^ ^]^ The FIrpic system^[^ ^11^ ^]^ and its derivatives based on 2‐(2,4‐difluorophenyl)pyridine ligands dominated the early development of high‐efficiency blue Ir(III)‐phosphorescent OLED materials;^[^ ^12^, ^13^, ^14^, ^15^ ^]^ this molecular strategy introduces electron‐withdrawing fluorine atoms (─F) into the aromatic moiety, which lowers the highest occupied molecular orbital (HOMO) energy level and widens the energy gap (Δ*E*_g_), thereby achieving a blue shift in the emission spectrum. However, due to their octahedral coordination geometry, the HOMO and the lowest unoccupied molecular orbital (LUMO) typically distribute on different ligands; thus, trs‐bidentate and bis‐tridentate Ir(III) complexes often exhibit significant spectral broadening arising from inherent intramolecular ligand‐to‐ligand charge transfer (^3^LLCT) and metal‐to‐ligand charge transfer (^3^MLCT) transitions.

**Figure 1:**
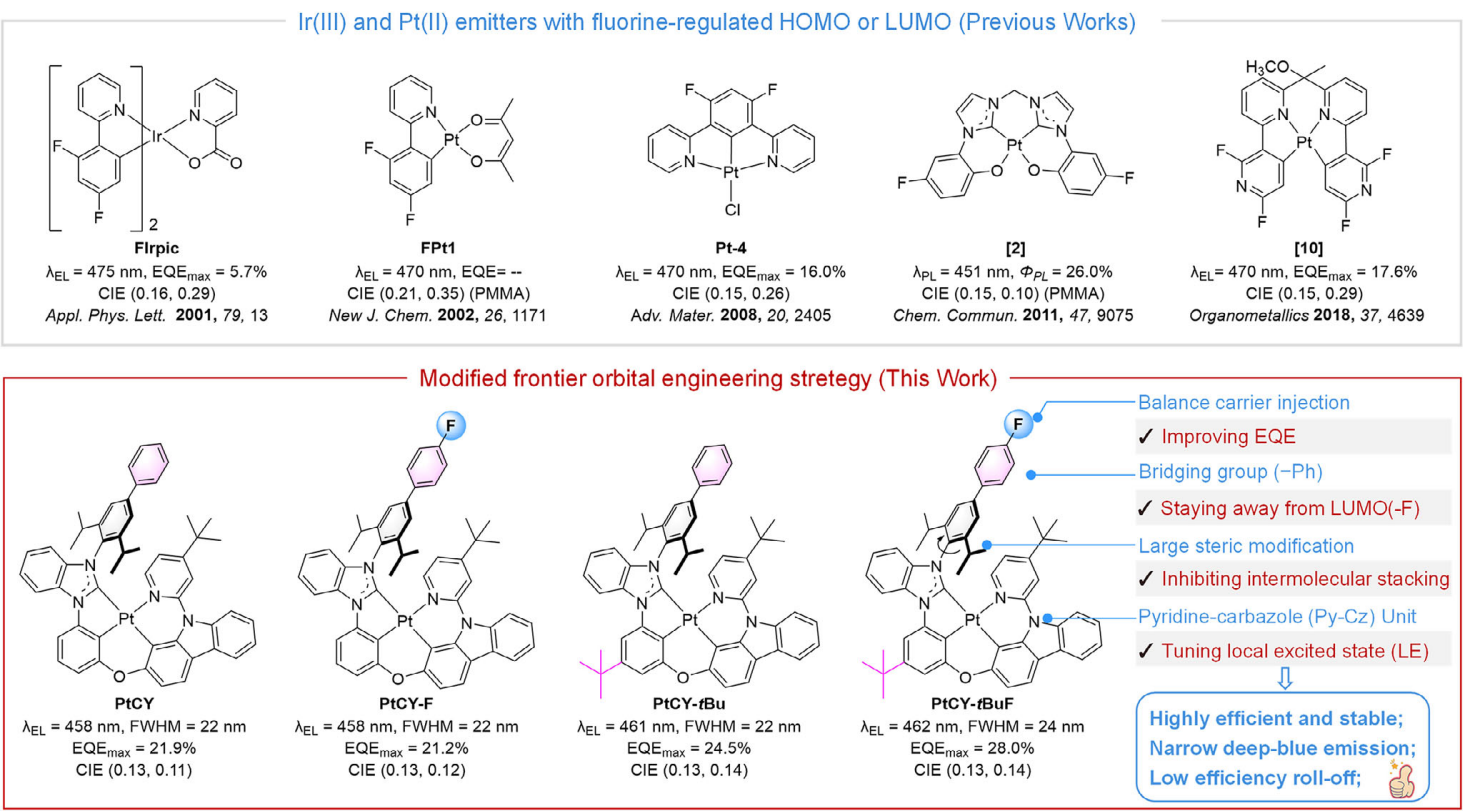
Molecular design and chemical structures of tetradentate Pt(II) complexes.

In contrast, the square‐planar coordination geometry of Pt(II) centers readily accommodates the construction of various ligands, enabling precise modulation of the photophysical properties of Pt(II) complexes.^[^ ^16^ ^]^ Among the reported fluorinated blue‐emitting Pt(II) complexes, most are either bis‐bidentate or tridentate coordination forms, while tetradentate structures remain relatively rare (**Figure** **1**). In 2002, Thompson et al.^[^ ^17^ ^]^ employed fluorine‐containing bidentate Pt(II) complex (FPt1) as phosphorescent dopants to fabricate OLEDs, achieving sky‐blue emission at 470 nm and CIE coordinates of (0.21, 0.35); however, did not report its EQE value. Subsequently, in 2008, Li et al.^[^ ^18^ ^]^ reported a fluorine‐containing tridentate Pt(II) complex (Pt4), which exhibited a lower EQE_max_ of only 16.0% and CIE coordinates of (0.15, 0.26). In 2011, Che et al.^[^ ^19^ ^]^ introduced fluorine atoms into a tetradentate Pt(II) complex featuring an NHC ligand (Pt2), achieving blue emission at 451 nm in PMMA film, with *Φ*_PL_ of 26.0% and CIE coordinates of (0.15, 0.10). In 2018, Lee et al.^[^ ^20^ ^]^ designed 2,3′‐bipyridine‐based tetradentate Pt(II) complex (Pt10) by introducing fluorine atoms into the pyridine ring of the LUMO, resulting in blue OLED peaking at 470 nm with EQE_max_ of 17.6% (Figure 1). However, energy‐level regulation of molecules through fluorine substitution at HOMO/LUMO positions limits the application of blue OLEDs, because of homolytic cleavage of the C‒F bond induced by electroreduction and the generation of stable radical intermediates; these intermediates evolved into non‐radiative recombination or exciton quenching centers, thereby impairing charge transport, recombination as well as energy transfer processes, and ultimately accelerating material degradation.^[^ ^3^, ^21^, ^22^, ^23^, ^24^, ^25^, ^26^ ^]^ This instability not only diminishes blue PhOLEDs device performances, such as reduced color purity and serious efficiency roll‐off, but also hinders the commercialization of blue phosphorescent materials.

Tetradentate Pt(II) complexes possess distinct advantages in modulating excited‐state properties owing to their rigid geometric configurations. However, the complexes adopted a square‐planar coordination geometry as a result of *dsp* ^2^ hybridization of the central Pt(II) ion. This geometry often promotes planar molecular stacking in solid‐state films, which arises from strong intermolecular π–π interactions and Pt···Pt interactions. Such stacking facilitates non‐radiative decay pathways, including triplet–triplet annihilation (TTA) and triplet–polaron annihilation (TPA), ultimately leading to heavy efficiency roll‐off in OLED devices.^[^ ^27^ ^]^ The emission spectra of the tetradentate Pt(II) complexes could be effectively regulated by balancing ^3^MLCT and locally excited state (^3^LE) characteristics in their lowest triplet state (T_1_), which enabled narrow‐band emission spectra with FWHM values below 30 nm.^[^ ^28^, ^29^, ^30^, ^31^, ^32^, ^33^, ^34^, ^35^, ^36^, ^37^, ^38^, ^39^, ^40^ ^]^ Early in 2014, Li et al. had designed a tetradentate Pt(II) complex, PtON7‐dtb, employing *N*‐heterocyclic carbene (NHC) and 2‐(4‐*tert*‐butylpyridyl)carbazole‐based ligand, which enabled a ^3^LE‐dominated T_1_ state mixed with large ^3^MLCT character, and realized an EQE_max_ > 20% and CIE_y_ < 0.1 for the first time; however, serious efficiency roll‐off was observed, and the EQE was only about 11% at 1000 cd m^−^^2^.^[^ ^28^ ^]^ To address the above issues, various steric strategies have been developed. In 2022, Lee et al. designed a tetradentate Pt(II) complex, PtON‐TBBI, by introducing a bulky 3,5‐di‐*tert*‐butylphenyl group into the NHC moiety, and the PtON‐TBBI‐based OLED employing exciplex hosts achieved a high EQE of 23.4% at 1000 cd m^−^^2^ with long operational lifetime, however, the electroluminescent (EL) spectrum was broadened with FWHM and CIE_y_ values of 43 nm and 0.197, respectively, revealing strong intermolecular interactions^[^ ^31^ ^]^ In 2024, Kim's group incorporated spirofluorene group into the carbazole moiety, enabling improved device performances by suppressing molecular vibrations and *π*–*π* interactions. The device exhibited EQE of about 20% at 1000 cd m^−^^2^, with a small FWHM of 22 nm and CIE_y_ value of 0.131.^[^ ^38^ ^]^ However, after device fabrication, the emission shoulder of the Pt(II) complex becomes significantly more pronounced, leading to a reduction in color purity. In 2025, Che et al. designed two tetradentate Pt(II) complexes (Pt1 and Pt2) containing 9,9‐dialkyl‐3‐aryl‐3,9‐dihydrofluoreno[2,3‐*d*]imidazoles (DAAFI)‐type NHC ligands with narrow FWHM of 21 nm, EQE_max_ of 28.6%, and CIE coordinates of (0.13, 0.15).^[^ ^39^ ^]^ Therefore, these research results show that the suppression of intermolecular interactions through steric hindrance was an effective strategy for improving the device performance of tetradentate Pt(II) complex‐based blue PhOLEDs.

In this work, we designed and synthesized four novel tetradentate Pt(II) complexes, namely PtCY, PtCY‐F, PtCY‐*t*Bu and PtCY‐*t*BuF (Figure 1). First, a bulky diisopropylphenyl (diPrPh) group was introduced to the *ortho*‐position of the NHC ligand to enhance molecular rigidity; this steric hindrance would facilitate effectively suppressing intermolecular interactions, reducing non‐radiative transitions and achieving narrowband spectral emission. Second, a *tert*‐butyl group was incorporated into the phenyl moiety of PtCY‐*t*Bu and PtCY‐*t*BuF, which could improve solubility and inhibit molecular aggregation. In addition, a fluorine atom was introduced into PtCY‐F and PtCY‐*t*BuF at positions remote from the LUMO distributions via molecular orbital engineering, which facilitates to balancing carrier mobility and reducing the device efficiency roll‐off.^[^ ^41^, ^42^ ^]^ This design strategy avoids the risk of C─F bond reduction or cleavage of the Pt(II) complexes in the emitting layer (EML), while improving the conductivity through enhanced intermolecular charge transport. The Pt(II) complexes‐based PhOLED exhibited deep‐blue emission with CIE_y_ < 0.15 and EQE_max_ of 21.2‒28.0% with low efficiency roll‐off.

## 2 Results and Discussion

### 2.1 Synthesis and Characterization

**Figure 2:**
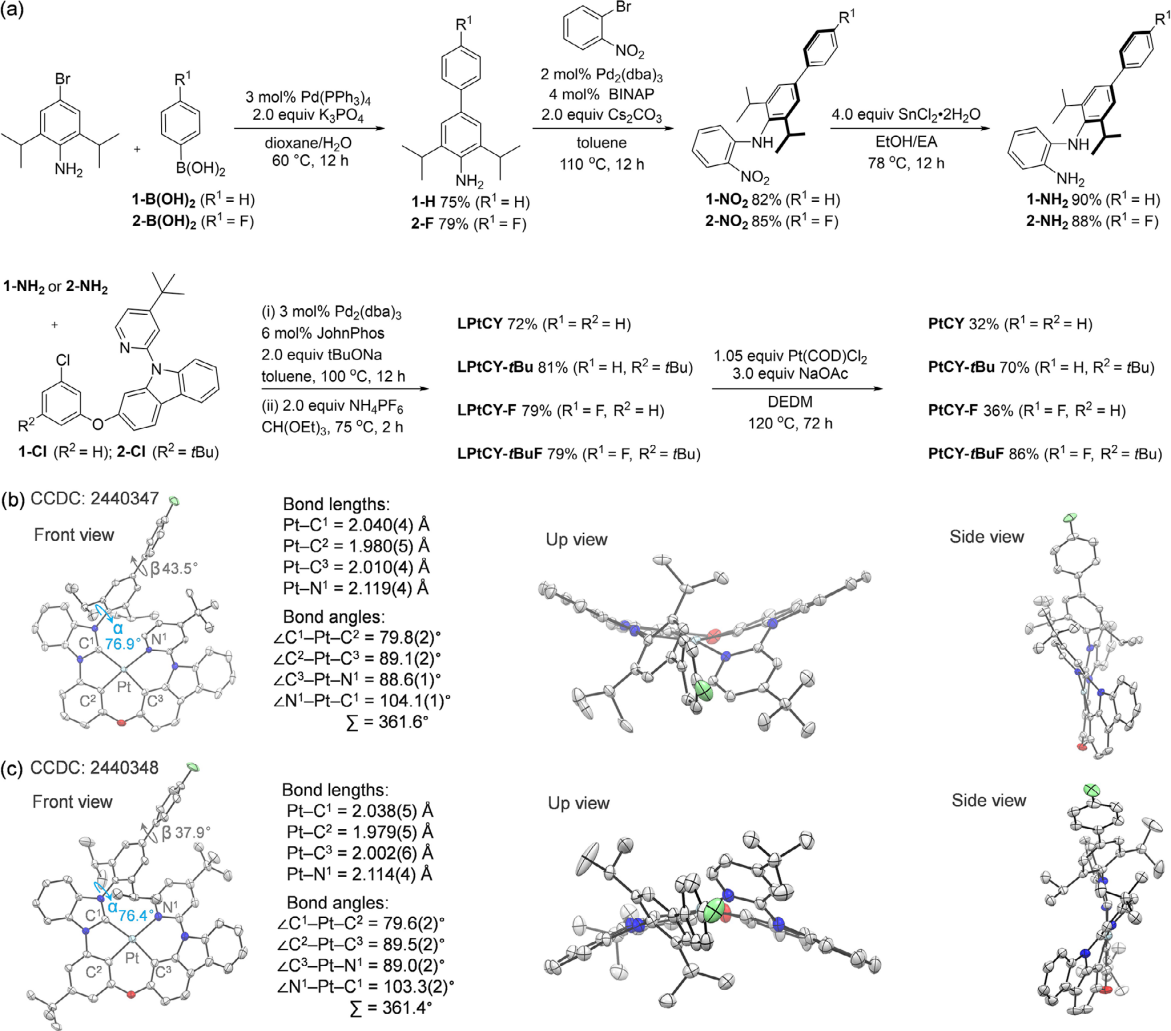
a) Synthesis of Pt(II) complexes. b,c) Different views of single‐crystal X‐ray diffraction molecular structure of PtCY‐F (CCDC 2440347) and PtCY‐tBuF (CCDC 2440348); hydrogen atoms are omitted for clarity.

The synthetic route of the tetradentate Pt(II) complexes is illustrated in **Figure** **2**a. Taking PtCY as an example, a Suzuki coupling reaction between 4‐bromo‐2,6‐diisopropylaniline and phenylboronic acid catalyzed by Pd(PPh_3_), afforded biphenyl amine intermediate **1‐H**. Subsequent Pd_2_(dba)_3_‐catalyzed C‒N cross‐coupling of **1‐H** with 1‐bromo‐2‐nitrobenzene yielded 3,5‐diisopropyl‐*N*‐(2‐nitrophenyl)‐[1,1′‐biphenyl]‐4‐amine (**1‐NO_2_**), which was then reduced using SnCl_2_·H_2_O to obtain the key diamine intermediate **1‐NH_2_**. Then, **1‐NH_2_** and **1‐Cl** underwent Pd_2_(dba)_3_‐catalyzed C─N coupling to afford an aromatic diamine intermediate, which was then subjected to a ring‐closure reaction in the presence of NH_4_PF_6_ to yield the benzimidazolium salt ligand **LCY**. Finally, metalation of **LCY** using Pt(COD)Cl_2_ [COD: (1*Z*,5*Z*)‐cycloocta‐1,5‐diene] in the presence of sodium acetate (NaOAc) in diethylene glycol dimethyl ether (DEDM) afforded the Pt(II) complex **PtCY**. The synthesized molecules were characterized by ^1^H nuclear magnetic resonance (^1^H‐NMR), ^13^C‐NMR and high‐resolution mass spectrometry (HRMS), with further details provided in the Supporting Information. In addition, to evaluate the importance of the bulky diisopropylbiphenyl (diPrPh‐Ph) moiety in PtCY, we additionally synthesized PtON5‐diPrPh. Details of its synthesis, characterization, photophysical properties, and device performance were discussed in the Supporting Information.

To further determine the absolute configuration and spatial geometry of molecules, single crystals of PtCY‐*t*Bu and PtCY‐*t*BuF were grown from a mixed solution of dichloromethane and methanol, and analyzed by X‐ray crystallography (Figure 2b,c; Tables S3 and S4, Supporting Information). Single‐crystal structural analysis revealed that the diPrPh plane and the NHC plane adopted a nearly perpendicular twisted conformation, with dihedral angles (α) of 76.9°and 76.4° in PtCY‐*t*Bu and PtCY‐*t*BuF, respectively (Figure 2b,c). The dihedral angles (*β*) between the diPrPh group and the bridging phenyl plane are 43.5° for PtCY‐*t*Bu and 37.9° for PtCY‐*t*BuF, as shown in Figure 2b,c. Notably, no intermolecular *π*–*π* interactions were observed in the crystal packing of PtCY‐*t*Bu and PtCY‐*t*BuF (Figures S3 and S4, Supporting Information). These results indicate that the incorporation of a bulky diPrPh group enhances the molecular rigidity, thereby suppressing non‐radiative transitions, limits intermolecular interactions in the solid state, and would contribute to suppressing non‐radiative transitions, resulting in *Φ*_PL_ enhancement of the Pt(II) complexes.

### 2.2 Theoretical Calculations

**Figure 3:**
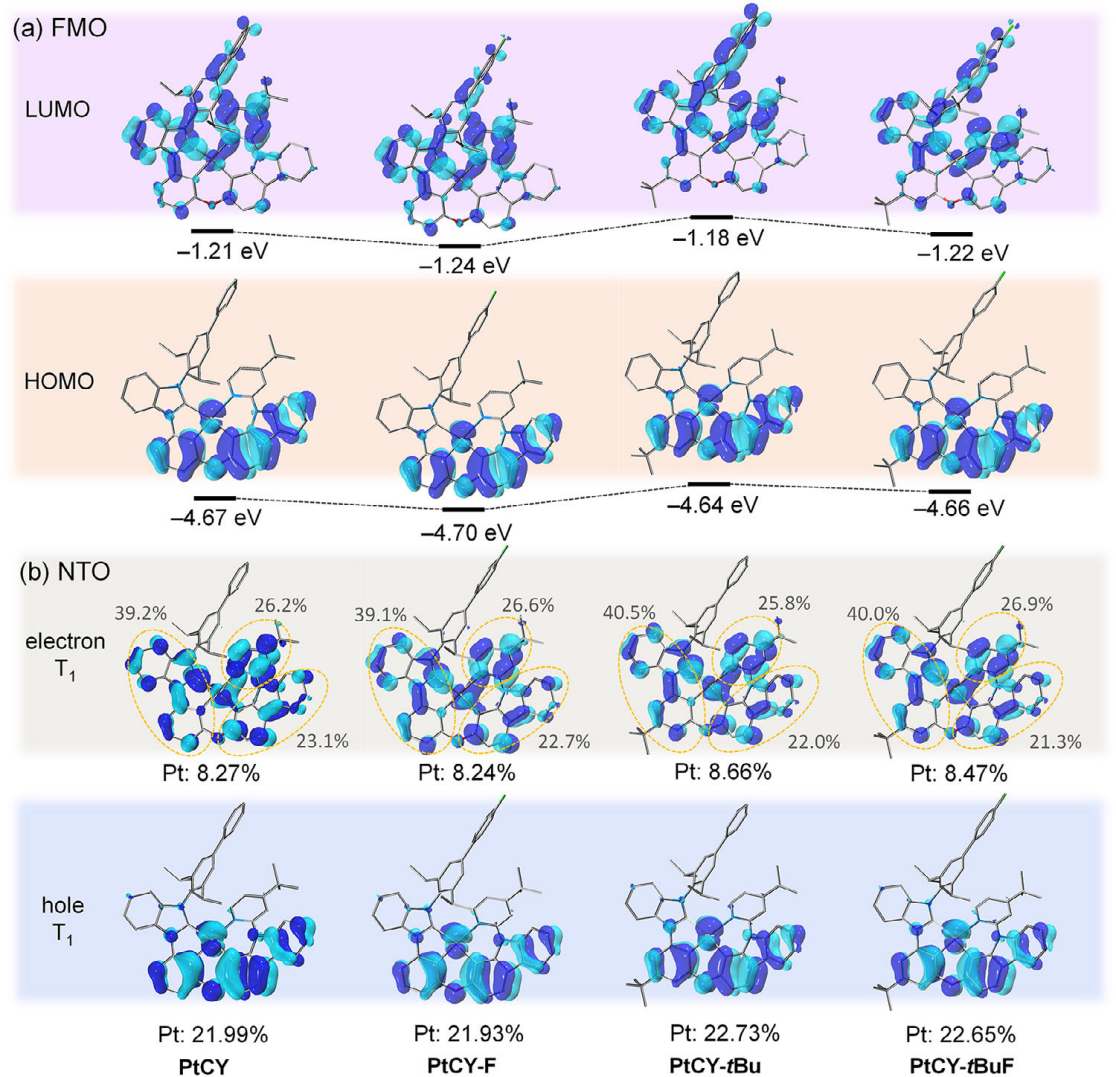
a) Frontier orbitals comparison of the Pt(II) complexes based on optimized S_0_ geometries. The hydrofen atoms have been omitted for clarity. b) Natural transition orbital analyses of the Pt(II) complexes for their T_1_ state.

To investigate the frontier molecular orbitals (FMOs) of the Pt(II) complexes, density functional theory (DFT) calculations were performed at the B3LYP/6‐31G(d)/LANL2DZ level for the ground state (S_0_) using the Gaussian 09 software package.^[^ ^43^, ^44^, ^45^ ^]^ All the Pt(II) complexes adopted a distorted square‐planar coordination geometry in the S_0_ state, as further evidenced by the close agreement between the calculated structural parameters (including bond lengths, bond angles, and dihedral angles) and those obtained from single‐crystal X‐ray diffraction analysis (Figure 2b,c; Tables S1 and S2, Supporting Information). In addition, the Pt(II) complexes exhibited similar frontier FMOs. The HOMOs are primarily localized on the π‐orbitals of the phenyl and carbazole moiety (π_PhCz_) and the d‐orbital of the Pt center, with energy levels ranging from −4.41 to −4.51 eV. The LUMOs are mainly distributed over the *π*‐orbitals of the pyridine (π_Py_), the phenyl/NHC (π_Ph/NHC_), and partially delocalized to π_diPrPh/Ph_ orbitals, with energy levels between −1.21 and −1.24 eV (**Figure** **3**a). Consequently, these Pt(II) complexes exhibit relatively large energy gaps (Δ*E*_g_ = 3.44–3.46 eV). To further study the excited state properties of the Pt(II) complexes, time‐dependent density functional theory (TD‐DFT) was performed based on the optimized S_0_ geometry and their natural transition orbitals (NTOs) were analyzed^[^ ^46^, ^47^, ^48^ ^]^ (Figure 3b). In the T_1_, all Pt(II) complexes exhibit ^3^LE (π_Cz_* → π_Cz_, 21.3−23.1%) state mixed with ^3^MLCT (π_Py_* → π_Ph_d_Pt_, 25.8−26.9%) and intramolecular ligand charge transfer (^3^ILCT, π_Ph/NHC_* → π_Ph_, 39.1−40.5%) characters. Compared with the previously reported fluorine‐containing tetradentate Pt(II) complexes that introduced −F atom at the LUMO,^[^ ^19^, ^20^ ^]^ the complexes designed in this work (PtCY‐F and PtCY‐*t*BuF) introduced −F atom, with only a minor portion of the LUMO distribution delocalized toward the fluorine atom (Figure 3a). Notably, the sterically bulky and rigid diPrPh group prevents electron distribution at the fluorine atom in the T_1_ state (Figure 3b). These findings demonstrate that the fluorine substituents do not participate in the radiative transition process (T_1_ → S_0_) and effectively mitigate C─F bond reduction and cleavage in the EML of OLEDs, facilitating the achievement of efficient and stable deep‐blue emission, which is consistent with the above molecular design strategy.

### 2.3 Thermal and Electrochemical Properties

Thermogravimetric analysis (TGA) revealed that the Pt(II) complexes had high thermal stability (Figure S10b, Supporting Information), with the thermal decomposition temperature at 5% mass loss (Δ*T*_5wt.%_) being 428–455 °C. Notably, Δ*T*_5wt.%_ of PtCY‐F (455 °C) and PtCY‐*t*BuF (455 °C) were higher than those of PtCY (450 °C) and PtCY‐*t*Bu (428 °C), which indicated that the introduction of fluorine atoms also improved the thermal stability of the Pt(II) complexes. In addition, the redox potential (*E*_ox_/*E*_red_) of the Pt(II) complexes was investigated by cyclic voltammetry (CV) (Figure S11, Supporting Information) and differential pulse voltammetry (DPV) (Figure S12, Supporting Information). The Pt(II) emitters possess oxidation potentials with a region of 0.56−0.57 V, and their reduction potentials are in the range of −2.70 – −2.72 V (Figure S12, Supporting Information). The HOMO and LUMO levels of the Pt(II) emitters calculated from the redox potentials are in line with the tendency of the DFT calculations. All the Pt(II) complexes exhibited irreversible redox behavior, primarily due to the presence of vacant d orbitals at the Pt(II) center, which were susceptible to attack by nucleophilic polar solvent molecules.^[^ ^49^, ^50^, ^51^ ^]^ Notably, they had two reduction peaks, which were attributed to electronic reductions occurring at the pyridine and NHC moiety, respectively.

### 2.4 Photophysical Properties

**Figure 4:**
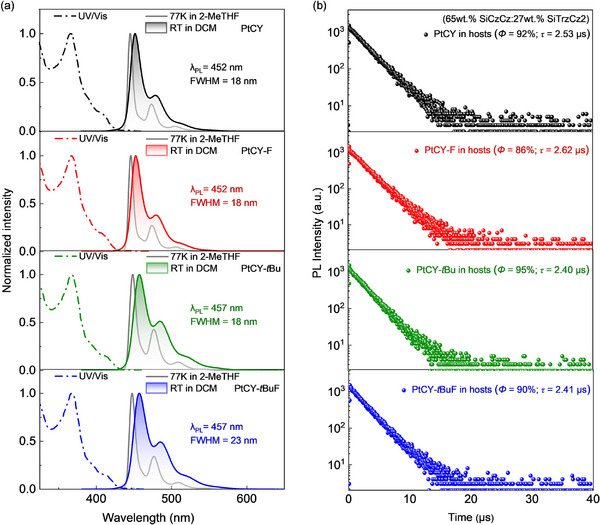
a) UV–vis spectra in dichloromethane at room temperature, and PL spectra in dichloromethane at room temperature and in 2‐methyltetrahydrofuran at 77 K. b) Transient PL decay curves of thermally evaporated 8 wt.% Pt(II) emitter: 65 wt.% SiCzCz:27 wt.% SiTrzCz2 thin films. The quantum efficiencies (*Φ*_PL_) and excited state lifetimes (*τ*) of the thin films are shown in the inset.

The UV‐visible absorption spectra of the Pt(II) complexes in dichloromethane (DCM) solution are shown in **Figure** **4**a. A strong absorption band was observed in the 350–400 nm, which was attributed to the ^1^MLCT (π_Ph_d_Pt_ → π_Py_* and π_Cz_d_Pt_ → π_NHC_*). Additionally, a weaker band corresponding to the ^3^MLCT absorption appeared in the 400–450 nm. The photoluminescence (PL) spectra in dichloromethane (DCM) at room temperature (RT) and in 2‐Methyltetrahydrofuran (2‐MeTHF) at 77 K are also recorded in Figure 4a and **Table** **1**. The tetradentate Pt(II) complexes exhibited narrowband deep‐blue emission in DCM at RT. Notably, the emission spectra of fluorine‐containing complexes of PtCY‐F (*λ*_PL_ = 452 nm) and PtCY‐*t*BuF (*λ*_PL_ = 457 nm) remain unchanged relative to no‐fluorine of PtCY (*λ*_PL_ = 452 nm) and PtCY‐*t*Bu (*λ*_PL_ = 457 nm); all the spectra showed small FWHM values of 18–23 nm. These observations were consistent with the results of the NTO analyses (Figure 3b). Notably, compared to PtCY‐tBu, the PtON5‐diPrPh exhibited a similar emission peak (*λ*_PL_ = 456 nm) and a lower vibronic shoulder but a larger FWHM (21 nm) in DCM at RT (Figure S10a, Supporting Information). Furthermore, the Pt(II) complexes exhibited obviously sharper and narrower‐band emission in 2‐MeTHF at 77 K (*λ*_PL_ = 445–448 nm, FWHM = 8.0–11.0 nm) compared to the PL spectra at RT, which is mainly attributed to the dominance of ^3^LE characteristics. The lowest triplet energy levels (*E*_T1_), calculated based on the main emission peaks, are as high as 2.77–2.79 eV. Thus, based on the energy level matching principle, 65 wt.% SiCzCz:27 wt.% SiTrzCz2 (*E*_T1_ = 2.84 eV^[^ ^31^ ^]^) was selected as the exciplex host materials, and thin films doped with 8 wt.% Pt(II) complexes were subsequently prepared by thermal evaporation to investigate their photophysical properties in the solid condition. On the one hand, the Pt(II) complexes achieved high *Φ*_PL_ with 86–95%, while having short, excited state lifetimes (*τ*) of 2.40–2.62 µs in exciplex host films (Figure 4b). On the other hand, they also showed blue‐shift narrowband emission in host films (*λ*_PL_ = 457–461 nm, FWHM = 19–22 nm) without observing host material emission (Figure S9, Supporting Information), which indicated an efficient transfer of energy from host to guest molecules.^[^ ^37^ ^]^ Additionally, for all the Pt(II) complexes, the calculated radiative transition rates (*k*_r_ = 3.2 × 10^5^– 3.4 × 10^5^) were one order of magnitude higher than the non‐radiative transition rates (*k*_nr_ = 2.1 × 10^4^– 4.1× 10^4^) (Table 1). Therefore, the photophysical properties of these films demonstrated that the incorporation of the bulky diPrPh group in the Pt(II) complexes effectively suppresses intermolecular interactions, achieving high color purity, high *Φ*_PL_, short *τ*, and rapid radiative transition processes (T_1_ → S_0_), which thereby contributed to further improving the device efficiency roll‐off.

**Table 1:** Comparison of photophysical and electrochemical properties of Pt(II) complexes.

Complex	*λ*_PL_ [nm]/FWHM [nm]^a)^	*λ*_PL_ [nm]/FWHM [nm] ^b)^	*Φ*_PL_/*τ* [µs] ^c)^	*E*_T1_ [eV] ^d)^	*k*_r_ (10^5^)/*k*_nr_ (10^4^)
PtCY	445/8	452/18	92%/2.53	2.79	3.6/3.2
PtCY‐F	445/8	452/18	86%/2.62	2.79	3.3/5.3
PtCY‐*t*Bu	448/11	457/18	95%/2.40	2.78	3.4/2.1
PtCY‐*t*BuF	447/10	457/23	90%/2.41	2.77	3.2/4.1

^a)^ Measured in 2‐MeTHF at 77 K;

^b)^ Measured in dichloromethane at room temperature;

^c)^ Measured in thermally evaporated 8 wt.% Pt(II) emitter:65 wt.% SiCzCz:27 wt.% SiTrzCz2 film, excitation wavelength = 340 nm;

^d)^ Estimated from phosphorescent spectrum at 77 K, *E*_T1_ = 1240/*λ*_PL_.

### 2.5 Device Performance

**Figure 5:**
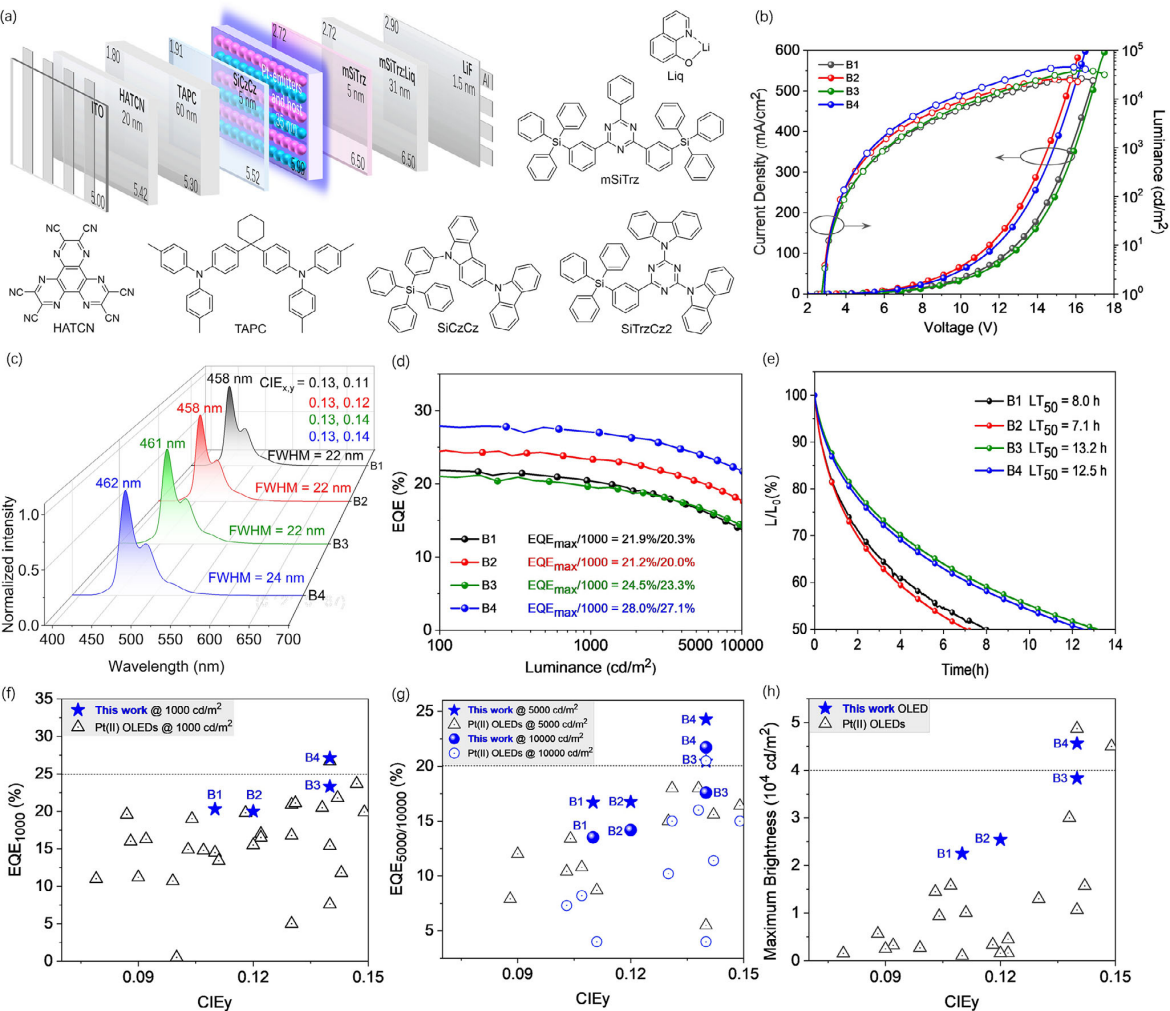
a) Energy level diagram of device structure. b) Current density‐voltage‐luminance curve. c) EL spectra of deep‐blue OLEDs at 1000 cd m^−2^. d) External quantum efficiency (EQE) vs luminance curves. e) The operational lifetimes of OLEDs based on Pt(II) emitters with a doping concentration of 8 wt.% at an *L*_0_ of 1000 cd m^−2^. f,g) CIE_y_ vs EQE data at 1000, 5000, and 10000 cd m^−2^ for Pt(II)‐based deep‐blue OLEDs with CIE_y_ < 0.15. h) CIE_y_ vs maximum brightness data for Pt(II)‐based deep‐blue OLEDs with CIE_y_ < 0.15.

The electroluminescent (EL) properties of Pt(II) complexes were studied, and PhOLED devices B1 (PtCY), B2 (PtCY‐F), B3 (PtCY‐*t*Bu), B4 (PtCY‐*t*BuF) and B5 (PtON5‐diPrPh)were fabricated by thermally evaporated vacuum deposition, respectively, using the same device structure of ITO/HATCN (20 nm)/TAPC (60 nm)/SiCzCz (5 nm)/8 wt.% Pt(II) emttier: 65 wt.% SiCzCz:27 wt.% SiTrzCz2 (35 nm)/mSiTrz (5 nm)/50 wt.% mSiTrz:50 wt.% Liq (31 nm)/LiF (1.5 nm)/Al. The device energy level diagram and chemical structures of each functional layer material of PhOLEDs are shown in **Figure** **5**a. Among them, 2,3,6,7,10,11‐hexacyano‐1,4,5,8,9,12‐hexaazatriphenylene (HATCN) and LiF were used as hole injection layer (HIL) and electron injection layer (EIL), respectively; 1,1′‐bis[4‐(di‐*p*‐tolylamino)phenyl]cyclohexane (TAPC) and mSiTrz:Liq (31 nm) as hole transport layer (HTL) and electron transport layer (ETL), respectively; 9‐(3‐(triphenylsilyl)phenyl)‐9*H*‐3,9′‐bicarbazole (SiCzCz) had a higher LUMO as an electron blocking layer (EBL) and 2‐phenyl‐4,6‐bis(3‐(triphenylsilyl)phenyl)‐1,3,5‐triazine (mSiTrz), had a lower HOMO as a hole blocking layer (HBL), thereby increasing the recombination probability of excitons in the EML and avoiding energy loss.

**Figure 6:**
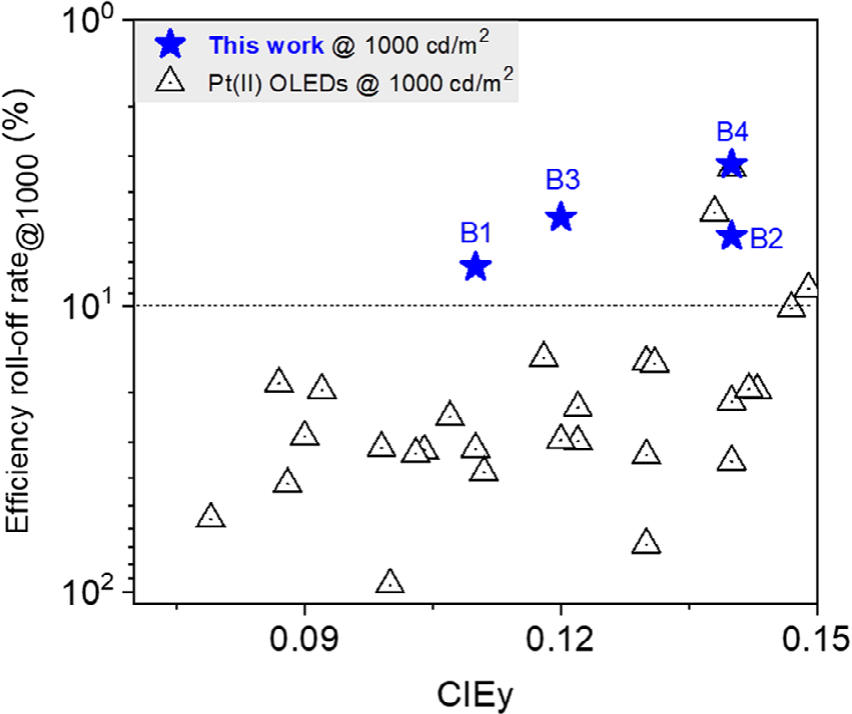
CIE_y_ vs Efficiency roll‐off rate at 1000 cd m^−2^ for Pt(II)‐based deep‐blue OLEDs with CIE_y_ < 0.15.

The EL performance of the PhOLEDs is shown in Figure 5b–f and the device data was summarized in **Table** **2**. Figure 5b illustrates the current–voltage–luminance (*J*–*V*–*L*) characteristic curves. The devices B1, B2, B3 and B4 exhibited low turn‐on voltages (*V*_on_) of 2.5, 2.9, 2.5 and 2.7 V and maximum luminous brightness (*L*_max_) values of 26 594, 25 423, 38 306 and 45 621 cd m^−2^ respectively. Compared with the fluorine‐free device, the fluorine‐containing device exhibits higher current density and brightness at the same driving voltage, which indicates that the introduction of the fluorine atom significantly improved carrier injection and transport ability. For example, At 12 V, compared to B3, device B4 achieved higher current density (121.7 vs 72.8 mA cm^−^^2^) and greater luminance (2.19 × 10⁴ vs 1.33 × 10⁴ cd m^−^^2^). The EL spectra of the PhOLEDs were almost consistent with those emission spectra measured in the doped films, and no emission signal from the host materials or Pt(II) complex aggregates was observed, indicating that the bulky diPrPh group design in the molecular system effectively suppressed intermolecular interactions. Devices B1, B2, B3, and B4 exhibited narrowband deep‐blue emission spectra peaking at 458, 458, 461, and 462 nm with FWHM values of 22, 22, 22 and 24 nm, respectively, resulting in all CIE_y_ values < 0.15; and their corresponding CIE coordinates were (0.13, 0.11), (0.13, 0.12), (0.13, 0.14), and (0.13, 0.14), respectively (Figure 5c). The FWHM values were comparable to those of deep‐blue multi‐resonant thermally activated delayed fluorescence (MR‐TADF) OLEDs.^[^ ^52^ ^]^ Additionally, as shown in Figure 5d, devices B1–B4 also achieved high EQE_max_ of 21.9%, 21.2%, 24.5%, and 28.0%, and still remained high EQE values of 20.3%, 20.0%, 23.3%, and 27.1% at a practical luminance of 1000 cd m^−^^2^, respectively, indicating low efficiency roll‐off of only 7.3%, 5.7%, 4.9% and 3.2%. Among them, the EQE values of PtCY‐F‐based device B2 and PtCY‐*t*BuF‐based device B4 are significantly higher than those of PtCY‐based device B1 and PtCY‐*t*Bu‐based device B3 at the same luminance. Due to the smaller atomic radius of the ─F atom, the molecular horizontal dipole orientation ratios (*Θ*) in PtCY‐*t*Bu and PtCY‐*t*BuF was expected to exhibit minimal difference. However, the EQE values of devices B3 (PtCY‐*t*Bu) and B4 (PtCY‐*t*BuF) show significant difference. Therefore, we speculated that *Θ* of the fluorine‐containing Pt(II) complexes was unlikely to be directly and primarily the factor leading to enhancement in EQE of the devices. This notable enhancement in EQE further demonstrated that the introduction of ─F into the Pt(II) emitters improved the balance of carrier transport in the emitting layer, thereby expanding the carrier recombination area beyond the interface between the emitting layer and the carrier transport layer. In addition, compared to conventional HTLs, TAPC may help reduce waveguide‐mode losses and enhance light outcoupling efficiency due to its relatively low refractive index, thereby benefiting the achievement of high EQE. Notably, to the best of our knowledge, device B4 achieved record high EQEs of 27.1%, 24.3%, and 21.7% at 1000, 5000, and 10 000 cd m^−2^ respectively and lowest efficiency roll‐off of 3.2% at 1000 cd m^−2^, among the reported Pt(II)‐based deep‐blue PhOLEDs with CIE_y_ < 0.15 (Figures 6 and 5f,g; Figure S14, Table S6, Supporting Information), while simultaneously reaching one of the highest *L*_max_ values of 45 621 cd m^−^^2^ (Figure 5h).

**Table 2:** Summary of device performance for Pt(II)‐based deep‐blue OLEDs.

Device	Emitter	*V*_on_ [V]	*λ*_EL_ [nm]	FWHM [nm]	CIE (x, y)	EQE ^a)^ [%]	*L*_max_ [cd m^−2^]	LT_50_ [h]
B1	PtCY	2.5	458	22	(0.13, 0.11)	21.9/20.3/16.7/13.5	26594	8.0
B2	PtCY‐F	2.9	458	22	(0.13, 0.12)	21.2/20.0/16.8/14.2	25423	7.1
B3	PtCY‐*t*Bu	2.5	461	22	(0.13, 0.14)	24.5/23.3/20.4/17.6	38306	13.2
B4	PtCY‐*t*BuF	2.7	462	24	(0.13, 0.14)	28.0/27.1/24.3/21.7	45621	12.5

^a)^ Maximum EQE, along with EQEs at 1000, 5000, and 10 000 cd m^−2^.

Additionally, the EL performances of PtON5‐diPrPh (B5) and PtCY (B1–B4) were compared as shown in Figure S13 (Supporting Information), and the corresponding device data are summarized in Table S5 (Supporting Information). The PtON5‐diPrPh‐doped in B5 also exhibited narrow‐band deep‐blue electroluminescence (*λ*_EL_ = 460 nm); however, its emission shoulder was slightly higher than that of devices B1–B4, resulting in a marginally larger FWHM of 23 nm (Figure S13a, Supporting Information). This observation suggested that the bulky diPrPh‐Ph moiety had an obvious advantage over the diPrPh group in Pt(II) complexes by more effectively suppressing intermolecular interactions, thereby improving the color purity of the devices. The turn‐on voltage of device B5 (2.7 V) was slightly higher than that of B3 (2.5 V). The *L*_max_ of device B5 reached 38 079 cd m^−2^, which was slightly lower than that of device B3 (38 306 cd m^−^^2^) (Figure S13b, Supporting Information). Meanwhile, device B5 achieved a high EQE_max_ of 25.6%, with EQE values maintained at 24.0, 20.4 and 17.4% at 1000, 5000 and 10 000 cd m^−2^, respectively, which comparable to the performance of device B3 (Figure S13c, Supporting Information). However, device B5 exhibited significantly lower EQE and maximum luminance compared to device B4 based on PtCY‐*t*BuF emitter with bulky steric diPrPh‐PhF moiety (Table S5, Supporting Information). These results further validated our molecular orbital engineering strategy: introducing a phenyl bridge to spatially separate the electron‐withdrawing the ─F atom from the LUMO‐distributed diPrPh moiety. It can effectively minimize the adverse influence of the ─F atom on the LUMO, thereby enhancing the optoelectronic properties of the F‐containing Pt(II) emitters.

To further evaluate the stability of the F‐containing Pt(II) emitters (PtCY‐F and PtCY‐*t*BuF), the operational lifetimes of devices B1–B4 were measured (Figure 5e). At an initial luminance (*L*_0_) of 1000 cd m^−^^2^, the LT_50_ values (the time required for the luminance to drop to 50% of the initial value) of devices B2 and B4 were 7.1 and 12.5 h, respectively, compared to 8.0 and 12.5 h for devices B1 and B3, suggesting no significant difference. Although the LT_50_ of device B5 based on PtON5‐diPrPh was not directly measured, it was estimated to be slightly longer than that of device B3 (Figure S13d, Supporting Information). This result indicates that the introduction of a fluorine atom, positioned away from the LUMO, did not lead to significant C─F bond cleavage. Furthermore, compared with other reported Pt(II)‐based OLEDs,^[^ ^28^, ^29^, ^30^, ^31^, ^32^, ^33^, ^34^, ^35^, ^36^, ^37^, ^38^, ^39^, ^40^, ^53^ ^]^ the relatively poor stability observed in devices B1–B4 may be attributed to the susceptibility of the tertiary C(sp^3^)─H bonds in the diPrPh moiety to photocatalytic degradation.^[^ ^54^ ^]^ This likely results in the decomposition of the Pt(II) complex under long‐term photoexcitation or electrical excitation conditions, thereby reducing the operational lifetime of the device.

## 3 Conclusion

In summary, we designed and developed four rigid tetradentate Pt(II) emitters based on a bulky diPrPh group, namely PtCY, PtCY‐F, PtCY‐*t*Bu, and PtCY‐*t*BuF. Systematically experimental studies combined with theoretical calculations demonstrated that the steric effect of the bulky diPrPh group significantly enhances the molecular rigidity, suppresses intermolecular interactions, and reduces non‐radiative decay processes. As a result, the Pt(II) emitters exhibited efficient, narrowband deep‐blue emission spectra with FWHM values of 18–23 nm and *Φ*_PL_ up to 95%. Furthermore, by employing a molecular orbital regulation strategy, a fluorine atom was directionally introduced into regions distant from the LUMO distribution. This approach not only effectively balances carrier recombination but also avoids C–F bond cleavage, thereby mitigating efficiency roll‐off in deep‐blue PhOLEDs. Devices B1–B4 all achieved deep‐blue emission with CIE_y_ < 0.15, small FWHM of 22–24 nm and EQE_max_ of 21.9%, 21.2%, 24.5%, and 28.0%, respectively, with low efficiency roll‐off. Notably, device B4 based on PtCY‐*t*BuF achieved record EQEs of 27.1%, 24.3%, and 21.7% at 1000, 5000, and 10 000 cd m^−^^2^ respectively and lowest efficiency roll‐off of 3.2% at 1000 cd m^−2^, along with one of the highest *L*_max_ of 45 621 cd m^−^^2^ in publicly reported Pt(II)‐based deep‐blue PhOLEDs with CIE_y_ < 0.15. Overall, this work innovatively developed a series of efficient deep‐blue tetradentate Pt(II) emitters with high color purity through the dual regulation of molecular rigidity and orbital engineering, thus providing valuable insights for the future development of high‐performance deep‐blue PhOLEDs (**Figure** **6**).

## Conflict of Interest

The authors declare no conflict of interest.

## Supporting information



Supporting Information

## Data Availability

The data that support the findings of this study are available in the supplementary material of this article.

## References

[1] C. W. Tang , S. A. VanSlyke , Appl. Phys. Lett. 1987, 51, 913.

[2] S. R. Forrest , Nature 2004, 428, 911. 15118718 10.1038/nature02498

[3] W. Song , J. Y. Lee , Adv. Opt. Mater. 2017, 5, 1600901.

[4] K. Klimes , Z.‐Q. Zhu , J. Li , Adv. Funct. Mater. 2019, 29, 1903068.

[5] J.‐M. Kim , K. H. Lee , J. Y. Lee , Adv. Opt. Mater. 2021, 10, 2101444.

[6] I. Siddiqui , S. Kumar , Y.‐F. Tsai , P. Gautam , Shahnawaz, K. K. , J.‐T. Lin , L. Khai , K.‐H. Chou , A. Choudhury , S. Grigalevicius , J.‐H. Jou , Nanomaterials 2023, 13, 2521. 37764550 10.3390/nano13182521 PMC10536903

[7] G. Li , L. Ameri , B. Dorame , Z.‐Q. Zhu , J. Li , Adv. Funct. Mater. 2024, 34, 2405066.

[8] Q.‐Y. Meng , X.‐L. Wen , J. Qiao , J. Phys. Chem. Lett. 2024, 15, 12571. 39680682 10.1021/acs.jpclett.4c03097

[9] Y. Kondo , K. Yoshiura , S. Kitera , H. Nishi , S. Oda , H. Gotoh , Y. Sasada , M. Yanai , T. Hatakeyama , Nat. Photon. 2019, 13, 678.

[10] M. A. Baldo , D. F. O'Brien , Y. You , A. Shoustikov , S. Sibley , M. E. Thompson , S. R. Forrest , Nature 1998, 395, 151.

[11] C. Adachi , R. C. Kwong , P. Djurovich , V. Adamovich , M. A. Baldo , M. E. Thompson , S. R. Forrest , Appl. Phys. Lett. 2001, 79, 2082.

[12] J.‐B. Kim , S.‐H. Han , K. Yang , S.‐K. Kwon , J.‐J. Kim , Y.‐H. Kim , Chem. Commun. 2015, 51, 58. 10.1039/c4cc07768g 25407660

[13] J. Xue , S. Chen , J. Liang , H. Bi , Y. Liu , Y. Wang , Chem. Eng. J. 2023, 463, 1424923.

[14] H. Shin , J.‐H. Lee , C.‐K. Moon , J.‐S. Huh , B. Sim , J.‐J. Kim , Adv. Mater. 2016, 28, 4920. 27060851 10.1002/adma.201506065

[15] C. Wu , K.‐N. Tong , K. Shi , W. He , M. Huang , J. Yan , S. Li , Z. Jin , X. Wang , S. Jung , J. Ma , Y. Zhuang , R.‐J. Xie , C. Yu , H. Meng , X. W. Sun , C. Yang , Y. Chi , F. Kang , G. Wei , Light Sci. Appl. 2025, 14, 156. 40204722 10.1038/s41377-025-01817-x PMC11982528

[16] G. Li , Y. She , in Light Emitting Diode–An Outlook on the Empirical Features and Its Recent Technological Advancements (Eds: J. Thirumalai ), IntechOpen, London 2018, Ch. 5.

[17] V. Adamovich , J. Brooks , A. Tamayo , A. M. Alexander , P. I. Djurovich , B. W. D'Andrade , C. Adachi , S. R. Forrest , New J. Chem. 2002, 26, 1171.

[18] X. Yang , Z. Wang , S. Madakuni , J. Li , G. E. Jabbour , Adv. Mater. 2008, 20, 2405.

[19] K. Li , X. Guan , C.‐W. Ma , W. Lu , Y. Chen , C.‐M. Che , Chem. Commun. 2011, 47, 9075. 10.1039/c1cc12943k 21761052

[20] C. Lee , R. Zaen , K.‐M. Park , K. H. Lee , J. Y. Lee , Y. Kang , Organometallics 2018, 37, 4639.

[21] D. Y. Kondakov , W. C. Lenhart , W. F. Nichols , J. Appl. Phys. 2007, 101, 024512.

[22] D. Y. Kondakov , T. D. Pawlik , W. F. Nichols , W. C. Lenhart , J. Soc. Inf. Disp. 2008, 16, 37.

[23] V. Sivasubramaniam , F. Brodkorb , S. Hanning , H. P. Loebl , V. Elsbergen , H. Boerner , U. Scherf , M. J. Kreyenschmidt , J. Fluor. Chem. 2009, 130, 640.

[24] R. Seifert , I. R. Moraes , S. Scholz , M. C. Gather , B. Lüssem , K. Leo , Org. Electron. 2013, 14, 115.

[25] G. Sato , D. Son , T. Ito , F. Osawa , Y. Cho , K. Marumoto , Phys. Status Solidi A. 2018, 215, 1700731.

[26] J. Jiang , J. Y. Lee , Mater. Today 2023, 68, 204.

[27] D. Wang , C. Cheng , T. Tsuboi , Q. Zhang , CCS Chem. 2020, 2, 1278.

[28] T. Fleetham , G. Li , L. Wen , J. Li , Adv. Mater. 2014, 26, 7116. 25207726 10.1002/adma.201401759

[29] H. Ma , K. Shen , Y. Wu , F. Xia , F. Yu , Z. Sun , C. Qian , Q. Peng , H.‐H. Zhang , C. You , G. Xie , X.‐C. Hang , W. Huang , Mater. Chem. Front. 2019, 3, 2448.

[30] J.‐S. Huh , M. J. Sung , S.‐K. Kwon , Y.‐H. Kim , J.‐J. Kim , Adv. Funct. Mater. 2021, 31, 2100967.

[31] J. Sun , H. Ahn , S. Kang , S.‐B. Ko , D. Song , H. A. Um , S. Kim , Y. Lee , P. Jeon , S.‐H. Hwang , Y. You , C. Chu , S. Kim , Nat. Photon. 2022, 16, 212.

[32] J.‐S. Huh , D. Y. Lee , K. H. Park , S.‐K. Kwon , Y.‐H. Kim , J.‐J. Kim , Chem. Eng. J. 2022, 450, 137836.

[33] H. J. Park , J.‐H. Jang , J.‐H. Lee , D.‐H. H , ACS Appl. Mater. Interfaces 2022, 14, 34901. 35867806 10.1021/acsami.2c06891

[34] K. Cheong , U. Jo , W. P. Hong , J. Y. Lee , Small Methods 2024, 8, 2300862. 10.1002/smtd.202300862 37926779

[35] K. Cheong , S. W. Han , J. Y. Lee , Small Methods 2024, 8, 2301710. 10.1002/smtd.202301710 38368260

[36] C. H. Ryu , U. Jo , I. Shin , M. Kim , K. Cheong , J. K. Bin , J. Y. Lee , K. M. Lee , Adv. Opt. Mater. 2024, 12, 2303109.

[37] J. Zhu , M. Huang , Y. Zhang , Z. Chen , Y. Deng , H. Zhang , X. Wang , C. Yang , Angew. Chem. Int. Ed. 2024, 64, 202418770. 10.1002/anie.202418770 39632276

[38] H. Lee , B. Park , G. R. Han , M. S. Mun , S. Kang , W. P. Hong , H. Y. Oh , T. Kim , Adv. Mater. 2024, 36, 2409394. 10.1002/adma.202409394 39263757

[39] H. Li , F.‐F. Hung , S. Wu , J. Qiu , C. Li , S. Nie , J. Yang , L. Duan , P. Zhou , G. Cheng , C.‐M. Che , Small 2025, 21, 2409662. 39916566 10.1002/smll.202409662 PMC11922001

[40] J. Kang , D. J. Shin , J. Y. Lee , Adv. Opt. Mater. 2025, 13, 2402653.

[41] P. Tao , W.‐L. Li , J. Zhang , S. Guo , Q. Zhao , H. Wang , B. Wei , S.‐J. Liu , X.‐H. Zhou , Q. Yu , B.‐S. Xu , W. Huang , Adv. Funct. Mater. 2016, 26, 881.

[42] F. Babudri , G. M. Farinola , F. Naso , R. Ragni , Chem. Commun. 2007, 10, 1003. 10.1039/b611336b 17325792

[43] G. Li , K. Xu , J. Zheng , X. Fang , Y.‐F. Yang , W. Lou , Q. Chu , J. Dai , Q. Chen , Y. Yang , Y.‐B. She , Nat. Commun. 2023, 14, 7089. 37925472 10.1038/s41467-023-42973-1 PMC10625603

[44] G. Li , S. Liu , Y. Sun , W. Lou , Y.‐F. Yang , Y. She , J. Mater. Chem. C. 2022, 10, 210.

[45] G. Li , H. Guo , X. Fang , Y.‐F. Yang , Y. Sun , W. Lou , Q. Zhang , Y. She , Chin. J. Chem. 2022, 40, 223.

[46] G. Li , K. Xu , J. Zheng , X. Fang , W. Lou , F. Zhan , C. Deng , Y.‐F. Yang , Q. Zhang , Y. She , J. Am. Chem. Soc. 2024, 146, 1667. 38175122 10.1021/jacs.3c12517

[47] G. Li , L. Ameri , T. Fleetham , Z.‐Q. Zhu , J. Li , Appl. Phys. Lett. 2020, 117, 253301.

[48] G. Li , X. Zhao , T. Fleetham , Q. Chen , F. Zhan , J. Zheng , Y.‐F. Yang , W. Lou , Y. Yang , K. Fang , Z. Shao , Q. Zhang , Y. She , Chem. Mater. 2020, 32, 537.

[49] Y. She , K. Xu , X. Fang , Y.‐F. Yang , W. Lou , Y. Hu , Q. Zhang , G. Li , Inorg. Chem. 2021, 60, 12972. 34374530 10.1021/acs.inorgchem.1c01405

[50] G. Li , A. Wolfe , J. Brooks , Z.‐Q. Zhu , J. Li , Inorg. Chem. 2017, 56, 8244. 28649846 10.1021/acs.inorgchem.7b00961

[51] G. Li , T. Fleetham , E. Turner , X.‐C. Hang , J. Li , Adv. Opt. Mater. 2015, 3, 390.

[52] T. Hatakeyama , K. Shiren , K. Nakajima , S. Nomura , S. Nakatsuka , K. Kinoshita , J. Ni , Y. Ono , T. Ikuta , Adv. Mater. 2016, 28, 2777. 26865384 10.1002/adma.201505491

[53] G. Li , Q. Chu , H. Yao , K. Wu , Y.‐B. She , Nat. Photon. 2025, 19, 977.

[54] Y. Liu , G.‐H. Xue , Z. He , J.‐P. Yue , M. Pan , L. Song , W. Zhang , J.‐H. Ye , D.‐G. Yu , J. Am. Chem. Soc. 2024, 146, 28350. 10.1021/jacs.4c09558 39374105

