# Supplementary material for: Dual Regulation of Molecular Rigidity and Orbital Engineering of Pt(II) Emitters for High‐Performance Deep‐Blue OLEDs
Source: Adv Sci (Weinh). 2025 Oct 3;12(48):e09722. doi: 10.1002/advs.202509722 (PMC12752585; doi:10.1002/advs.202509722)
Supplement: Supplementary file 1 — Supporting Information [file ADVS-12-e09722-s001.pdf]

Supplementary Information

Dual Regulation of Molecular Rigidity and Orbital Engineering of Pt(II) Emitters for High-Performance Deep-Blue OLEDs

Chengyao Zhang[#], Kewei Xu[#], Yun-Fang Yang*, Yuanbin She*, Guijie Li*

[1] Center for Phosphorescent Material Research, College of Chemical Engineering, Zhejiang University of Technology, Hangzhou, Zhejiang 310014, P. R. China

*Corresponding Author(s): Yun-Fang Yang: yangyf@zjut.edu.cn; Yuanbin She: sheyb@zjut.edu.cn; Guijie Li: guijieli@zjut.edu.cn

Table of Contents

1	**Supplementary Methods**	S3−S4
2	**Synthesis and characterization of tetradentate Pt(II) complexes**	S4−S17
3	^{1}H, ^{13}C NMR spectra and HRMS spectra of intermediates, ligand and Pt(II) complexes	S18−S51
4	**Table S1. DFT calculations for Pt(II) complexes**	S52
5	**Table S2. Selected bond lengths (Å), bond Angles (°) and dihedral (°) for tetradentate Pt(II) complexes based on the X-ray crystallographic analysis and DFT calculation analysis.**	S53
6	**Figure S1. Density functional theory (DFT) calculated frontier orbits of Pt(II) complexes.**	S54
7	**Figure S2. Time-dependent density functional theory (TD-DFT) calculations of Pt(II) complexes.**	S55
8	**Table S3. Crystal data and structure refinements of PtCY-F.**	S56
9	**Table S4. Crystal data and structure refinements of PtCY-*t*BuF.**	S57
10	**Figure S3. The molecular packing structure of PtCY-F.**	S58
11	**Figure S4. The molecular packing structure of PtCY-*t*BuF.**	S58
12	**Figure S5. HPLC analysis of PtCY-sublimated sample**.	S59
13	**Figure S6. HPLC analysis of PtCY-F-sublimated sample**.	S59
14	**Figure S7. HPLC analysis of PtCY-*t*Bu-sublimated sample**.	S60
15	**Figure S8. HPLC analysis of PtCY- *t*BuF-sublimated sample**.	S60
16	**Figure S9. PL spectra of Pt(Ⅱ) complexes at RT in host (65 wt.% SiCzCz:27 wt.% SiTrzCz2) film.**	S61
17	**Figure S10. (a) Comparison of PL spectra in dichloromethane at room temperature for the Pt(II) complex. (b) TGA curves of Pt(II) complexes.**	S61
18	**Figure S11. Electrochemical properties of Pt(II) complexes.** Cyclic voltammograms.	S62
19	**Figure S12. Electrochemical properties of Pt(II) complexes.** differential pulse voltammetry (DPV) curves.	S62
20	**Figure S13. EL performances of PtON5-diPrPh (B5) and PtCY (B1–B4)**	S63
21	**Table S5**. **Summary of device performance for Pt(II)-based deep-blue OLEDs**	S63
22	**Figure S14. Pt(II) complexes-based deep-blue emitters.**	S64
23	**Table S6. Device performance data for Pt(II)-based single-layer deep-blue phosphorescent OLEDs with CIE_y < 0.15**	S65
23	**Supplementary References**	S66
24	**Cartesian coordinates of the optimized structures**	S67−S77

Materials and Methods

Synthesis and Structural Characterization. Unless noted, all commercial reagents were purchased and used as received without further purification. ^{1}H NMR spectra were recorded at 400 or 500 MHz, and ^{13}C NMR spectra were recorded at 100 or 125 MHz NMR Bruker instruments in $CDCl_3$ or DMSO-d_6 solutions and chemical shifts were referenced to tetramethylsilane (TMS) or residual protiated solvent. If $CDCl_3$ was used as solvent, ^{1}H NMR spectra were recorded with TMS (δ = 0.00 ppm) or residual $CHCl_3$ (δ = 7.26 ppm) as internal references; ^{13}C NMR spectra were recorded with TMS (δ = 0.00 ppm) or $CDCl_3$ (δ = 77.00 ppm) as internal references. If DMSO-d_6 was used as solvent, ^{1}H NMR spectra were recorded with TMS (δ = 0.00 ppm) or residual DMSO (δ = 2.50 ppm) as internal references; ^{13}C NMR spectra were recorded with TMS (δ = 0.00 ppm) and DMSO-d_6 (δ = 39.52 ppm) as internal references. The following abbreviations (or combinations thereof) were used to explain ^{1}H NMR ultiplicities: s = singlet, d = doublet, t = triplet, q = quartet, p = quintet, m = multiplet, br = broad. All of the new compounds were analyzed for HRMS on a Waters mass spectrometer using electrospray ionization in positive ion mode of ESI-Q-TOF.

X-ray Crystallography. X-ray diffraction data were collected at 170 K on a Bruker D8 Venture diffractometer using graphite-monochromated Mo-Kα radiation (λ = 0.71073 Å) from a rotating anode generator.

Quantum Chemical Calculations. The theoretical calculations were performed using Gaussian 09 package. The molecular geometries of ground states (S_0) were optimized with the density functional theory (DFT) method at the B3LYP level. The DFT calculations were performed using a B3LYP function with a basis set of 6-31G(d) for C, H, O and N atoms; the LANL2DZ basis set with ECP was used for Pt atoms.

Electrochemistry. Cyclic voltammetry and different pulsed voltammetry were performed using a CH1760E electrochemical analyzeraccording previous report. 0.1 M tetra-*n*-butylammonium hexafluorophosphate was used as the supporting electrolyte, anhydrous *N*, *N*-dimethylformamide, was used as the solvents for the E_{ox} and E_{red} measurements, and the solutions were bubbled with nitrogen for 15 min prior to the test. Silver wire, platinum wire and glassy carbon were used as pseudoreference electrode, counter electrode, and working electrode respectively. Scan rate was 300 mV/s. The redox potentials are based on the values measured from different pulsed voltammetry and are reported relative to an internal reference ferrocenium/ferrocene (Cp_2Fe/Cp_2Fe^+). The reversibility of reduction or oxidation was determined using CV. As defined, if the magnitudes of the peak anodic and the peak

cathodic current have an equal magnitude as scan speeds of 100 mV/s or slower, then the process is considered reversible; if the magnitudes of the peak anodic and the peak cathodic currents are not equal, but the return sweeps are nonzero, the process is considered quasi-reversible; otherwise, the process is considered irreversible.

Photophysical Measurements. The absorption spectra were measured on a Hitachi U-3900 UV−VIS Spectrometer. Steady state emission experiments were performed on HITACHI F-7000 spectrometer. Low temperature (77 K) emission spectra and lifetimes were measured in 2-MeTHF cooled with liquid nitrogen. Lifetime measurements and quantum efficiency were measured using an Edinburgh FS5 Spectrofluorometer equipped with an integrating sphere.

Synthesis and Characterization of Tetradentate Pt(II) Complexes

Synthesis of **1-NH2**:

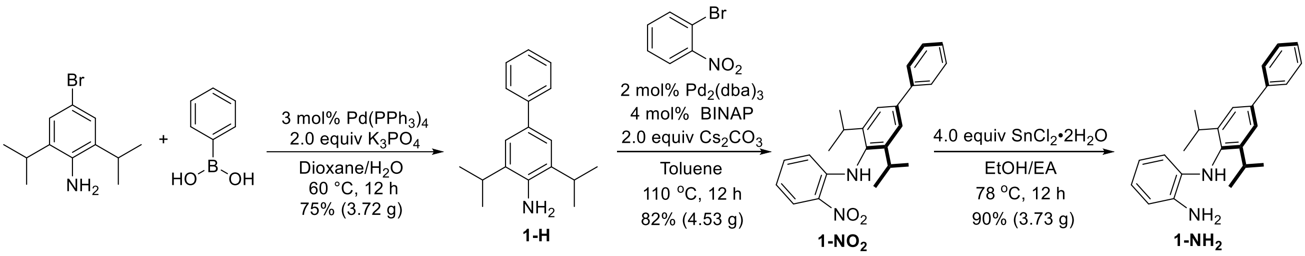

Synthesis of **1-H**: 2-(2-amino-5-bromo-3-isopropylphenyl)propan-1-ylium (5.00 g, 19.59 mmol, 1.0 equiv), phenylboronic acid (3.58 g, 29.39 mmol, 1.5 equiv), K_3PO_4 (8.32 g, 39.19 mmol, 2.0 equiv), $Pd(PPh_3)_4$ (679 mg, 0.59 mmol, 3 mol%) were added sequentially to a dry three-necked flask equipped with a magnetic stir bar. The flask was evacuated and backfilled with nitrogen, this evacuation and backfill procedure was repeated twice. Then dioxane (40 mL) and H_2O (40 mL) were added into the flask under nitrogen atmosphere at room temperature. Then the flask was placed in an oil bath and the reaction mixture was stirred and heated at 60 °C for 12 hours, the reaction was monitored by TLC until the reaction was completed. The reaction mixture was cooled down to room temperature, then the solvent was removed under reduced pressure and the residue was diluted with ethyl acetate. The mixture was washed with water, the organic layer was separated, and dried over Na_2SO_4, filtered. The filtrate was concentrated under reduced pressure and the residue was purified through column chromatography on silica gel (eluent: petroleum ether/ dichloromethane = 20:1–10:1) to obtain the desired product as a red liquid 3.72 g in 75% yield. ^1H NMR (500 MHz, DMSO-d_6): δ (ppm) 1.20 (s,

6H), 1.22 (s, 6H), 3.04–3.10 (m, 2H), 4.73 (s, 2H), 7.16 (s, 2H), 7.19–7.23 (m, 1H), 7.36–7.39 (m, 2H), 7.52 (dd, J = 8.0, 1.0 Hz, 2H).

Syntesis of **1-NO₂**: **1-H** (3.72 g, 14.68 mmol, 1.0 equiv), 1-bromo-2-nitrobenzene (2.97 g, 14.68 mmol, 1.0 equiv), $Pd_2(dba)_3$ (269 mg, 0.29 mmol, 2 mol%), 1.1'-Binaphthyl-2.2'-diphemyl phosphine (BINAP 366 mg, 0.59 mmol, 4 mol%), and Cs_2CO_3 (9.57 g, 29.36 mmol, 2.0 equiv) were added sequentially to a dry three-necked flask equipped with a magnetic stir bar. The flask was evacuated and backfilled with nitrogen, this evacuation and backfill procedure was repeated twice. Then toluene (40 mL) was added and the mixture was heated at 110 °C under a nitrogen atmosphere for 12 hours. After the reaction was completed, the mixture was cooled to room temperature and concentrated under reduced pressure. The residue was purified through column chromatography on silica gel (eluent: petroleum ether/ dichloromethane = 30:1–20:1) to obtain the desired product as a yellow solid 4.53 g in 82% yield. ^{1}H NMR (400 MHz, DMSO-d_6): δ (ppm) 1.10 (d, J = 7.2 Hz, 6H), 1.20 (d, J = 6.8 Hz, 6H), 2.97–3.04 (m, 2H), 6.36 (dd, J = 8.4, 0.8 Hz, 1H), 6.73–6.78 (m, 1H), 7.38–7.45 (m, 2H), 7.50(t, J = 7.6 Hz, 2H), 7.54 (s, 2H), 7.72–7.74 (m, 2H), 8.14 (dd, J = 8.8, 1.6 Hz, 1H), 9.33 (s, 1H).

Synthesis of **1-NH₂**: **1-NO₂** (4.53 g, 12.03 mmol, 1.0 equiv) and $SnCl_2{\cdot}2H_2O$ (10.86 g, 48.13 mmol, 4.0 equiv) were added sequentially to a dry three-necked flask equipped with a magnetic stir bar. The flask was evacuated and backfilled with nitrogen, this evacuation and backfill procedure was repeated twice. Then EtOH (20 mL) and ethyl acetate (20 mL) were added into the flask under nitrogen atmosphere at room temperature. Then the flask was placed in an oil bath and the reaction mixture was stirred and heated at 78 °C for 12 hours, the reaction was monitored by TLC until the reaction was completed. The reaction mixture was cooled down to room temperature, and quenched with $NaHCO_3$. The mixture was extracted with ethyl acetate, the organic layer was separated, and dried over Na_2SO_4, filtered. The filtrate was concentrated under reduced pressure and the residue was purified through column chromatography on silica gel (eluent: petroleum ether/ dichloromethane = 20:1–15:1) to obtain the desired product as a purple solid 3.73 g in 90% yield. ^{1}H NMR (500 MHz, DMSO-d_6): δ (ppm) 1.13 (d, J = 30.5 Hz, 12H), 3.08–3.18 (m, 2H), 4.82 (s, 2H), 5.86 (dd, J = 9.5, 2.5 Hz, 1H), 6.00 (s, 1H), 6.32 (td, J = 9.5, 1.5 Hz, 1H), 6.44 (td, J = 9.0, 1.0 Hz, 1H), 6.61 (dd, J = 9.5, 1.5 Hz, 1H), 7.35 (t, J = 9.0 Hz, 1H), 7.44 (s, 2H), 7.47 (t, J = 9.5 Hz, 2H), 7.68 (d, J = 9.5Hz, 2H).

Synthesis of **2-NH₂**:

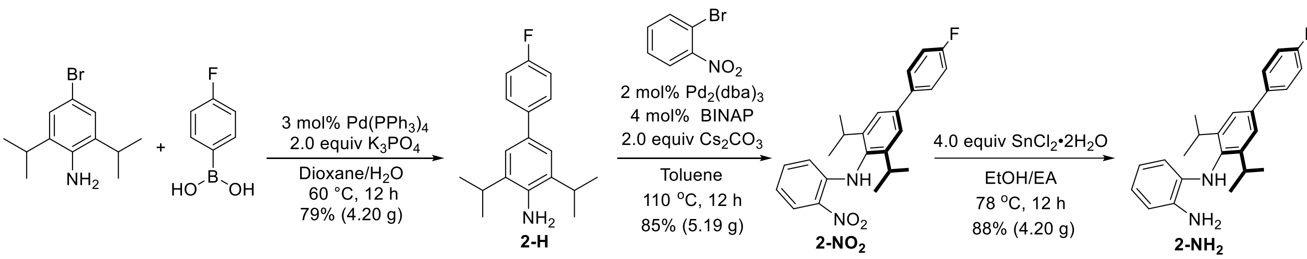

Synthesis of **2-H:** 2-(2-amino-5-bromo-3-isopropylphenyl)propan-1-ylium (5.00 g, 19.59 mmol, 1.0 equiv), (4-fluorophenyl)boronic acid (4.11 g, 29.39 mmol, 1.5 equiv), K_3PO_4 (8.32 g, 39.19 mmol, 2.0 equiv), $Pd(PPh_3)_4$ (679 mg, 0.59 mmol, 3 mol%) were added sequentially to a dry three-necked flask equipped with a magnetic stir bar. The flask was evacuated and backfilled with nitrogen, this evacuation and backfill procedure was repeated twice. Then dioxane (40 mL) and H_2O (40 mL) were added into the flask under nitrogen atmosphere at room temperature. Then the flask was placed in an oil bath and the reaction mixture was stirred and heated at 60 °C for 12 hours, the reaction was monitored by TLC until the reaction was completed. The reaction mixture was cooled down to room temperature, then the solvent was removed under reduced pressure and the residue was diluted with ethyl acetate. The mixture was washed with water, the organic layer was separated, and dried over Na_2SO_4, filtered. The filtrate was concentrated under reduced pressure and the residue was purified through column chromatography on silica gel (eluent: petroleum ether/ dichloromethane = 20:1–10:1) to obtain the desired product as a red liquid 4.20 g in 79% yield. ^1H NMR (500 MHz, DMSO-d_6): δ (ppm) 1.20 (s, 6H), 1.21 (s, 6H), 3.02–3.10 (m, 2H), 4.73 (s, 2H), 7.12 (s, 2H), 7.17–7.20 (m, 2H), 7.53–7.56 (m, 2H).

Syntesis of **2-NO₂**: **2-H** (4.20 g, 15.48 mmol, 1.0 equiv), 1-bromo-2-nitrobenzene (3.13 g, 15.48 mmol, 1.0 equiv), $Pd_2(dba)_3$ (283 mg, 0.31 mmol, 2 mol%), 1.1'-Binaphthyl-2.2'-diphemyl phosphine (BINAP 385 mg, 0.62 mmol, 4 mol%), and Cs_2CO_3 (10.09 g, 30.95 mmol, 2.0 equiv) were added sequentially to a dry three-necked flask equipped with a magnetic stir bar. The flask was evacuated and backfilled with nitrogen, this evacuation and backfill procedure was repeated twice. Then toluene (40 mL) was added and the mixture was heated at 110 °C under a nitrogen atmosphere for 12 hours. After the reaction was completed, the mixture was cooled to room temperature and concentrated under reduced pressure. The residue was purified through column chromatography on silica gel (eluent: petroleum

ether/ dichloromethane = 30:1–20:1) to obtain the desired product as a yellow solid 5.19 g in 85% yield. ^{1}H NMR (500 MHz, DMSO-d_6): δ (ppm) 1.11 (d, J = 7.0 Hz, 6H), 1.20 (d, J = 6.5 Hz, 6H), 2.98–3.03 (m, 2H), 6.34 (dd, J = 8.5, 1.0 Hz, 1H), 6.73–6.77 (m, 1H), 7.30–7.33 (m, 2H), 7.41–7.45 (m, 1H), 7.52 (s, 2H), 7.76–7.79 (m, 2H), 8.14 (dd, J = 8.5, 1.5 Hz, 1H), 9.30 (s, 1H).

Synthesis of **2-NH₂**: **2-NO₂** (5.19 g, 13.16 mmol, 1.0 equiv) and $SnCl_2{\cdot}2H_2O$ (11.87 g, 52.62 mmol, 4.0 equiv) were added sequentially to a dry three-necked flask equipped with a magnetic stir bar. The flask was evacuated and backfilled with nitrogen, this evacuation and backfill procedure was repeated twice. Then EtOH (25 mL) and ethyl acetate (25 mL) were added into the flask under nitrogen atmosphere at room temperature. Then the flask was placed in an oil bath and the reaction mixture was stirred and heated at 78 °C for 12 hours, the reaction was monitored by TLC until the reaction was completed. The reaction mixture was cooled down to room temperature, and quenched with $NaHCO_3$. The mixture was extracted with ethyl acetate, the organic layer was separated, and dried over Na_2SO_4, filtered. The filtrate was concentrated under reduced pressure and the residue was purified through column chromatography on silica gel (eluent: petroleum ether/ dichloromethane = 20:1–15:1) to obtain the desired product as a purple solid 4.20 g in 88% yield. ^{1}H NMR (500 MHz, DMSO-d_6): δ (ppm) 1.12 (d, J = 23.0 Hz, 12H), 3.09–3.16 (m, 2H), 4.82 (s, 2H), 5.85 (dd, J = 9.5, 1.5 Hz, 1H), 6.00 (s, 1H), 6.32 (td, J = 9.5, 1.5 Hz, 1H), 6.44 (td, J = 9.5, 1.5 Hz, 1H), 6.61 (dd, J = 10.0, 2.0 Hz, 1H), 7.26–7.31 (m, 2H), 7.41 (s, 2H), 7.71–7.75 (m, 2H).

Synthesis of **PtCY**:

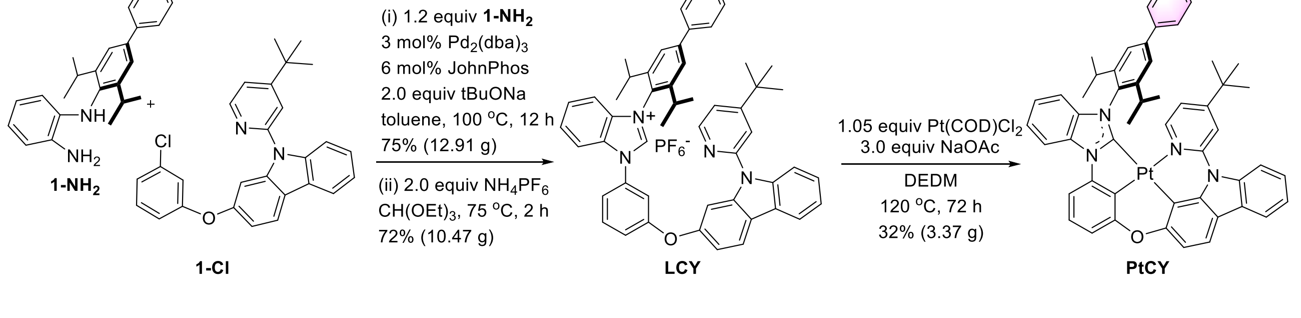

Syntesis of **LCY**: Compound **1-Cl** (10.00 g, 23.42 mmol, 1.0 equiv), **1-NH₂** (9.68 g, 28.11 mmol, 1.2 equiv), $Pd_2(dba)_3$ (643 mg, 0.70 mmol, 3 mol%), 2-(di-*tert*-butylphosphino)biphenyl (JohnPhos, 420 mg, 1.40 mmol, 6 mol%), and *t*-BuONa (4.50 g, 46.84 mmol, 2.0 equiv) were added sequentially to a dry three-necked flask equipped with a magnetic stir bar. The flask was evacuated and backfilled with

nitrogen, this evacuation and backfill procedure was repeated twice. Then toluene (100 mL) was added and the mixture was heated at 100 °C under a nitrogen atmosphere for 12 hours. After the reaction was completed, the mixture was cooled to room temperature and concentrated under reduced pressure. The residue was purified through column chromatography on silica gel (eluent: petroleum ether/ethyl acetate = 30:1) to obtain the desired product diamine as powder solid 12.91 g in 75% yield. The intermediate diamine was not stable enough and easily oxidized by air, thereby, directly used for the next step. The diamine (12.00 g, 16.33 mmol, 1.0 equiv), and NH_4PF_6 (5.32 g, 32.65 mmol, 2.0 equiv) were added to dry three-necked flask equipped with a magnetic stir bar. Then $CH(OEt)_3$ (80 mL) was added and the mixture was heated at 75 °C under a nitrogen atmosphere for 2 hours. After the reaction was completed by TLC monitoring, the mixture was cooled down to room temperature. Then concentrated under reduced pressure. The residue was purified through column chromatography on silica gel (eluent: petroleum ether/dichloromethane = 1:1–dichloromethane/ethyl acetate = 50:1) to obtain the desired product as powder solid 10.47 g in 72% yield. ^1H NMR (500 MHz, $CDCl_3$): δ (ppm) 1.08 (d, J = 6.5 Hz, 6H), 1.28 (d, J = 6.5 Hz, 6H), 1.40 (s, 9H), 2.20–2.27 (m, 2H), 7.19 (dd, J = 8.5, 2.0 Hz, 1H), 7.32–7.36 (m, 4H), 7.39 (d, J = 8.0 Hz, 1H), 7.43–7.47 (m, 2H), 7.52 (t, J = 7.5 Hz, 2H), 7.56–7.57 (m, 3H), 7.61 (d, J = 1.0 Hz, 1H), 7.65 (d, J = 7.0 Hz, 2H), 7.66–7.75 (m, 5H), 7.96 (d, J = 8.5 Hz, 1H), 8.11 (d, J = 7.5 Hz, 1H), 8.16 (d, J = 8.5 Hz, 1H), 8.59 (d, J = 5.5 Hz, 1H), 9.61 (s, 1H). ^{13}C NMR (125 MHz, $CDCl_3$): δ (ppm) 24.07, 24.24, 29.14, 30.53, 35.17, 103.53, 110.65, 113.36, 113.74, 114.37, 116.30, 119.17, 119.32, 120.13, 120.57, 121.15, 121.29, 121.54, 123.83, 124.07, 126.00, 127.47, 128.31, 128.73, 128.90, 128.97, 130.92, 132.51, 132.98, 133.43, 140.05, 140.08, 140.76, 141.02, 145.56, 146.54, 149.47, 151.34, 154.18, 160.00, 163.51. HRMS (ESI): calcd for $C_{52}H_{49}N_4O$ $[M-PF_6]^+$ 745.3901, found 745.3931.

Synthesis of **PtCY**: Ligand **LCY** (10.00 g, 11.22 mmol, 1.00 equiv), $Pt(COD)Cl_2$ (4.41 g, 11.79 mmol, 1.05 equiv), and NaOAc (2.76 g, 33.67 mmol, 3.00 equiv) were added sequentially to a dry three-necked flask equipped with a magnetic stir bar. The flask was evacuated and backfilled with nitrogen, this evacuation and backfill procedure was repeated twice. Then DEDM (100 mL) was added and the mixture was stirred at 120 °C for 72 hours under a nitrogen atmosphere. After the reaction was completed, the mixture was cooled down to room temperature and extracted with dichloromethane three times, dried over Na_2SO_4, filtered, and the filtrate was concentrated under reduced pressure. The

residue was purified through column chromatography on silica gel (eluent: petroleum ether/dichloromethane = 4:1–2:1) to obtain the desired product as yellow solid 3.37 g in 32% yield. ^{1}H NMR (500 MHz, $CDCl_3$): δ (ppm) 0.75–1.00 (br, 6H), 1.00 (s, 9H), 1.11–1.35 (br, 6H), 2.97–3.35 (br, 2H), 6.08 (dd, J = 6.5, 2.0 Hz, 1H), 7.02 (d, J = 8.0 Hz, 1H), 7.19 (d, J = 8.0 Hz, 1H), 7.29 (dd, J = 13.0, 5.0 Hz, 2H), 7.33 (d, J = 7.0 Hz, 1H), 7.35–7.38 (m, 2H), 7.41 (d, J = 8.0 Hz, 1H), 7.44 (dd, J = 7.0, 1.5 Hz, 2H), 7.53 (t, J = 7.5 Hz, 3H), 7.67 (t, J = 6.5 Hz, 3H), 7.73 (d, J = 8.0 Hz, 1H), 7.78 (d, J = 8.0 Hz, 1H), 7.89 (d, J = 1.5 Hz, 1H), 8.01 (d, J = 7.5 Hz, 1H), 8.20 (d, J = 8.0 Hz, 1H), 8.71 (d, J = 6.5 Hz, 1H). ^{13}C NMR (125 MHz, $CDCl_3$) δ (ppm) 22.76 (br), 25.08, 28.49, 29.88, 35.10, 108.21, 111.55, 111.81, 112.03, 112.22, 112.97, 113.83, 113.89, 114.59, 115.61, 115.99, 116.60, 120.18, 122.33, 122.84, 123.09, 123.76, 124.79, 124.88, 126.95, 128.09, 128.96, 129.28, 131.52, 131.95, 137.59, 138.92, 139.85, 142.64, 144.17, 147.49, 148.70, 149.73, 153.99, 154.96, 155.11, 162.75, 191.98. HRMS (ESI): calcd for $C_{52}H_{47}N_4OPt$ $[M+H]^+$ 938.3392, found 938.3411.

Synthesis of **PtCY-F**:

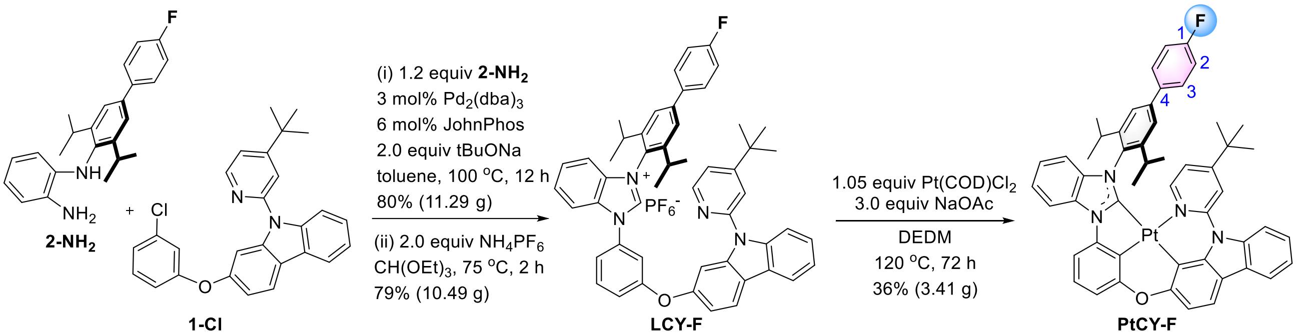

Syntesis of **LCY-F**: Compound **2-NH₂** (8.00 g, 18.74 mmol, 1.0 equiv), **6** (8.15 g, 22.49 mmol, 1.2 equiv), $Pd_2(dba)_3$ (515 mg, 0.56 mmol, 3 mol%), 2-(Di-*tert*-butylphosphino)biphenyl (JohnPhos, 336 mg, 1.12 mmol, 6 mol%), and *t*-BuONa (3.60 g, 37.48 mmol, 2.0 equiv) were added sequentially to a dry three-necked flask equipped with a magnetic stir bar. The flask was evacuated and backfilled with nitrogen, this evacuation and backfill procedure was repeated twice. Then toluene (80 mL) was added and the mixture was heated at 100 °C under a nitrogen atmosphere for 12 hours. After the reaction was completed, the mixture was cooled to room temperature and concentrated under reduced pressure. The residue was purified through column chromatography on silica gel (eluent: petroleum ether/ethyl acetate = 30:1) to obtain the desired product diamine as powder solid 11.29 g in 80% yield. The intermediate diamine was not stable enough and easily oxidized by air, thereby, directly used for the

next step. The diamine (11.00 g, 14.61 mmol, 1.0 equiv), and NH_4PF_6 (4.76 g, 29.22 mmol, 2.0 equiv) were added to dry three-necked flask equipped with a magnetic stir bar. Then $CH(OEt)_3$ (100 mL) was added and the mixture was heated at 75 °C under a nitrogen atmosphere for 2 hours. After the reaction was completed by TLC monitoring, the mixture was cooled down to room temperature. Then concentrated under reduced pressure. The residue was purified through column chromatography on silica gel (eluent: petroleum ether/dichloromethane = 1:1–dichloromethane/ethyl acetate = 50:1) to obtain the desired product as powder solid 10.49 g in 79% yield. ^{1}H NMR (500 MHz, $CDCl_3$): δ (ppm) 1.07 (d, J = 7.0 Hz, 6H), 1.26 (d, J = 6.5 Hz, 6H), 1.39 (s, 9H), 2.20–2.26 (m, 2H), 7.17– 7.21 (m, 3H), 7.31–7.35 (m, 4H), 7.38 (d, J = 8.0 Hz, 1H), 7.45 (td, J = 7.0, 1.0 Hz, 1H), 7.51 (s, 2H), 7.54 (dd, J = 8.5, 1.5 Hz, 1H), 7.59–7.62 (m, 3H), 7.65–7.72 (m, 4H), 7.73 (t, J = 8.0 Hz, 1H), 7.96 (d, J = 8.5 Hz, 1H), 8.10 (d, J = 8.0 Hz, 1H), 8.15 (d, J = 8.5 Hz, 1H), 8.58 (d, J = 5.5 Hz, 1H), 9.67 (s, 1H). ^{13}C NMR (125 MHz, $CDCl_3$): δ (ppm) 23.99, 24.16, 29.08, 30.48, 35.13, 103.54, 110.62, 113.31, 113.62, 113.74, 114.33, 115.85 (d, $^2J_{CF}$ = 22.5 Hz), 116.24, 119.14, 120.10, 120.46, 121.13, 121.27, 121.52, 123.78, 123.85, 125.98, 126.01, 128.89, 129.03, 129.14 (d, $^3J_{CF}$ = 8.8 Hz), 130.84, 132.40, 132.95, 133.32, 136.13 (d, $^4J_{CF}$ = 3.8 Hz), 140.03, 140.71, 140.80, 144.46, 146.64, 149.43, 151.31, 154.09, 160.02, 162.97 (d, $^1J_{CF}$ = 246.3 Hz), 163.47. HRMS (ESI): calcd for $C_{52}H_{48}FN_4O$ $[M-PF_6]^+$ 763.3807, found 763.3823.

Synthesis of **PtCY-F**: Ligand **LCY-F** (9.00 g, 9.90 mmol, 1.00 equiv), $Pt(COD)Cl_2$ (3.89 g, 10.40 mmol, 1.05 equiv), and NaOAc (2.44 g, 29.70 mmol, 3.00 equiv) were added sequentially to a dry three-necked flask equipped with a magnetic stir bar. The flask was evacuated and backfilled with nitrogen, this evacuation and backfill procedure was repeated twice. Then DEDM (90 mL) was added and the mixture was stirred at 120 °C for 72 hours under a nitrogen atmosphere. After the reaction was completed, the mixture was cooled down to room temperature and extracted with dichloromethane three times, dried over Na_2SO_4, filtered, and the filtrate was concentrated under reduced pressure. The residue was purified through column chromatography on silica gel (eluent: petroleum ether/dichloromethane = 4:1–2:1) to obtain the desired product as yellow solid 3.41 g in 36% yield. ^{1}H NMR (500 MHz, $CDCl_3$): δ (ppm) 0.82–1.24 (br, 12H), 1.02 (s, 9H), 2.81–3.46 (br, 2H), 6.07 (dd, J = 6.5, 5.0 Hz, 1H), 7.00 (d, J = 8.0 Hz, 1H), 7.20 (dd, J = 8.0, 1.0 Hz, 1H), 7.22 (t, J = 8.5 Hz, 2H), 7.28 (t, J = 7.5 Hz, 2H), 7.33 (td, J = 7.5, 1.0 Hz, 1H), 7.35–7.38 (m, 2H), 7.41 (d, J = 8.0 Hz, 1H), 7.43–

7.46 (m, 2H), 7.65 (dd, J = 17.5, 0.5 Hz, 1H), 7.65 (d, J = 3.5 Hz, 1H), 7.66 (d, J = 2.0 Hz, 1H), 7.72 (d, J = 8.0 Hz, 1H), 7.78 (d, J = 8.0 Hz, 1H), 7.89 (d, J = 2.0 Hz, 1H), 8.01 (dd, J = 7.5, 1.0 Hz, 1H), 8.20 (d, J = 8.0 Hz, 1H), 8.71 (d, J = 6.0 Hz, 1H). ^{13}C NMR (125 MHz, $CDCl_3$): δ (ppm) 22.78 (br), 25.07, 28.48, 29.92, 35.11, 108.23, 111.51, 111.85, 112.00, 112.17, 113.00, 113.73, 113.86, 114.62, 115.57, 115.90 (d, $^2J_{CF}$ = 21.3 Hz), 116.00, 116.63, 120.20, 122.37, 122.65, 123.10, 123.77, 124.82, 124.91, 128.56 (d, $^3J_{CF}$ = 8.8 Hz), 129.30, 131.53, 132.01, 136.00 (d, $^4J_{CF}$ = 3.8 Hz), 137.55, 138.89, 141.58, 144.14, 147.67, 148.75, 149.70, 153.97, 154.97, 155.10, 162.70, 162.86 (d, $^1J_{CF}$ = 246.3 Hz), 191.97. HRMS (ESI): calcd for $C_{52}H_{46}FN_4OPt$ $[M+H]^+$ 956.3298, found 956.3282.

Synthesis of **PtCY-*t*Bu**:

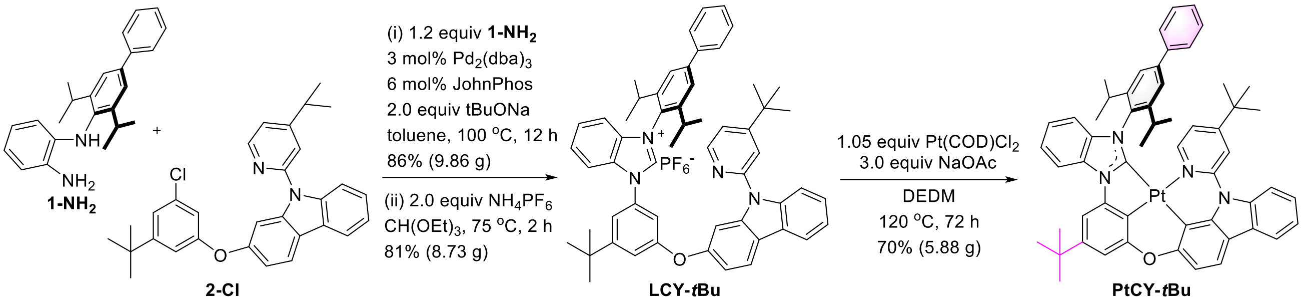

Syntesis of **LCY-*t*Bu**: Compound **2-Cl** (7.00 g, 14.49 mmol, 1.0 equiv), **1-NH₂** (5.99 g, 17.39 mmol, 1.2 equiv), $Pd_2(dba)_3$ (398 mg, 0.43 mmol, 3 mol%), 2-(di-*tert*-butylphosphino)biphenyl (JohnPhos, 260 mg, 0.86 mmol, 6 mol%), and *t*-BuONa (2.79 g, 28.98 mmol, 2.0 equiv) were added sequentially to a dry three-necked flask equipped with a magnetic stir bar. The flask was evacuated and backfilled with nitrogen, this evacuation and backfill procedure was repeated twice. Then toluene (70 mL) was added and the mixture was heated at 100 °C under a nitrogen atmosphere for 12 hours. After the reaction was completed, the mixture was cooled to room temperature and concentrated under reduced pressure. The residue was purified through column chromatography on silica gel (eluent: petroleum ether/ethyl acetate = 30:1) to obtain the desired product diamine as powder solid 9.86 g in 86% yield. The intermediate diamine was not stable enough and easily oxidized by air, thereby, directly used for the next step. The diamine (9.00 g, 11.38 mmol, 1.0 equiv), and NH_4PF_6 (3.71 g, 22.75 mmol, 2.0 equiv) were added to dry three-necked flask equipped with a magnetic stir bar. Then $CH(OEt)_3$ (90 mL) was added and the mixture was heated at 75 °C under a nitrogen atmosphere for 2 hours. After the reaction was completed by TLC monitoring, the mixture was cooled down to room temperature.

Then concentrated under reduced pressure. The residue was purified through column chromatography on silica gel (eluent: petroleum ether/dichloromethane = 1:1–dichloromethane/ethyl acetate = 50:1) to obtain the desired product as powder solid 8.73 g in 81% yield. ^{1}H NMR (400 MHz, $CDCl_3$): δ (ppm) 1.08 (d, J = 6.8 Hz, 6H), 1.27 (d, J = 6.8 Hz, 6H), 1.40 (d, J = 4.4 Hz, 18H), 2.19–2.29 (m, 2H), 7.07 (t, J = 2.0 Hz, 1H), 7.18 (dd, J = 8.4, 2.4 Hz, 1H), 7.32–7.39(m, 3H), 7.42–7.46(m, 4H), 7.51 (t, J = 7.2 Hz, 2H), 7.56 (s, 2H), 7.61–7.72(m, 7H), 7.91(d, J = 8.4 Hz, 1H), 8.10(d, J = 7.6 Hz, 1H), 8.15(d, J = 8.4 Hz, 1H), 8.59(d, J = 5.2 Hz, 1H), 9.71 (s, 1H). ^{13}C NMR (100 MHz, $CDCl_3$) δ (ppm) 24.00, 24.23, 29.07, 30.51, 30.87, 35.14, 35.48, 103.29, 110.44, 110.57, 113.54, 113.66, 114.33, 116.26, 116.49, 118.29, 119.15, 120.08, 121.04, 121.10, 121.47, 123.82, 123.96, 125.91, 126.06, 127.45, 128.22, 128.65, 128.78, 128.91, 131.00, 132.65, 133.38, 139.99, 140.07, 140.74, 140.99, 145.34, 146.46, 149.49, 151.34, 154.34, 157.09, 159.60, 163.40.

Synthesis of **PtCY-*t*Bu**: Ligand **LCY-*t*Bu** (8.00 g, 8.45 mmol, 1.00 equiv), Pt(COD)Cl_2 (3.32 g, 8.87 mmol, 1.05 equiv), and NaOAc (2.08 g, 25.34 mmol, 3.00 equiv) were added sequentially to a dry three-necked flask equipped with a magnetic stir bar. The flask was evacuated and backfilled with nitrogen, this evacuation and backfill procedure was repeated twice. Then DEDM (80 mL) was added and the mixture was stirred at 120 °C for 72 hours under a nitrogen atmosphere. After the reaction was completed, the mixture was cooled down to room temperature and extracted with dichloromethane three times, dried over Na_2SO_4, filtered, and the filtrate was concentrated under reduced pressure. The residue was purified through column chromatography on silica gel (eluent: petroleum ether/dichloromethane = 4:1–2:1) to obtain the desired product as yellow solid 5.88 g in 70% yield. ^{1}H NMR (500 MHz, $CDCl_3$): δ (ppm) 0.82–0.93 (br, 6H), 1.00 (s, 9H), 1.09–1.26 (br, 6H), 1.52 (s, 9H), 3.00–3.32 (br, 2H), 6.08 (dd, J = 6.5, 2.0 Hz, 1H), 7.02 (d, J = 8.0 Hz, 1H), 7.24 (d, J = 1.5 Hz, 1H), 7.27 (t, J = 7.5 Hz, 1H), 7.32 (t, J = 7.5 Hz, 1H), 7.37 (td, J = 7.0, 1.0 Hz, 1H), 7.40–7.42 (m, 1H), 7.45 (td, J = 7.0, 1.5 Hz, 2H), 7.48–7.51 (m, 1H), 7.53 (t, J = 7.0 Hz, 3H), 7.67 (d, J = 7.0 Hz, 2H), 7.71 (d, J = 1.5 Hz, 1H), 7.73 (d, J = 8.0 Hz, 1H), 7.77 (d, J = 8.0 Hz, 1H), 7.88 (d, J = 2.0 Hz, 1H), 8.01 (d, J = 7.5 Hz, 1H), 8.18 (d, J = 8.5 Hz, 1H), 8.71 (d, J = 6.0 Hz, 1H). ^{13}C NMR (125 MHz, $CDCl_3$) δ (ppm) 22.80 (br), 25.10, 28.49, 29.89, 31.65, 34.89, 35.09, 106.11, 107.87, 111.42, 111.79, 112.07, 112.22, 112.90, 113.80, 113.91, 115.62, 115.87, 116.46, 120.13, 122.27, 122.84, 122.99, 123.67, 124.72, 126.97, 128.06, 128.95, 129.32, 131.59, 132.00, 137.64, 138.95, 139.91, 142.61,

144.27, 147.49, 148.67, 148.74, 149.49, 153.51, 155.01, 155.31, 162.56, 192.22. HRMS (ESI): calcd for $C_{56}H_{55}N_4OPt$ $[M+H]^+$ 994.4018, found 994.4015.

Synthesis of **PtCY-*t*BuF**:

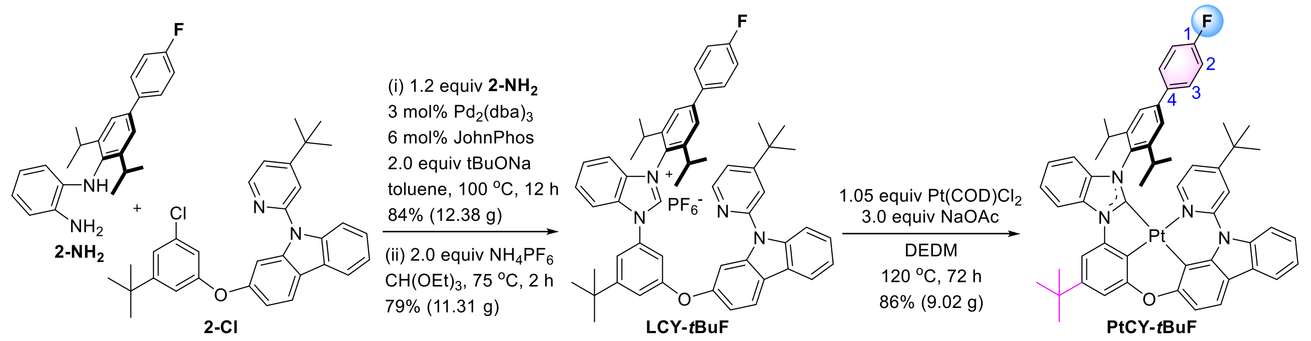

Syntesis of **LCY-*t*BuF**: Compound **2-Cl** (8.80 g, 18.22 mmol, 1.0 equiv), **2-NH_2** (7.92 g, 21.86 mmol, 1.2 equiv), $Pd_2(dba)_3$ (500 mg, 0.55 mmol, 3 mol%), 2-(di-*tert*-butylphosphino)biphenyl (JohnPhos, 327 mg, 1.09 mmol, 6 mol%), and *t*-BuONa (3.50 g, 36.44 mmol, 2.0 equiv) were added sequentially to a dry three-necked flask equipped with a magnetic stir bar. The flask was evacuated and backfilled with nitrogen, this evacuation and backfill procedure was repeated twice. Then toluene (100 mL) was added and the mixture was heated at 100 °C under a nitrogen atmosphere for 12 hours. After the reaction was completed, the mixture was cooled to room temperature and concentrated under reduced pressure. The residue was purified through column chromatography on silica gel (eluent: petroleum ether/ethyl acetate = 30:1) to obtain the desired product diamine as powder solid 12.38 g in 84% yield. The intermediate diamine was not stable enough and easily oxidized by air, thereby, directly used for the next step. The diamine (12.00 g, 14.83 mmol, 1.0 equiv), and NH_4PF_6 (4.84 g, 29.66 mmol, 2.0 equiv) were added to dry three-necked flask equipped with a magnetic stir bar. Then $CH(OEt)_3$ (100 mL) was added and the mixture was heated at 75 °C under a nitrogen atmosphere for 2 hours. After the reaction was completed by TLC monitoring, the mixture was cooled down to room temperature. Then concentrated under reduced pressure. The residue was purified through column chromatography on silica gel (eluent: petroleum ether/dichloromethane = 1:1–dichloromethane/ethyl acetate = 50:1) to obtain the desired product as powder solid 11.31 g in 79% yield. ^1H NMR (500 MHz, $CDCl_3$): δ (ppm) 1.07 (d, J = 7.0 Hz, 6H), 1.27 (d, J = 7.0 Hz, 6H), 1.40 (d, J = 2.5 Hz, 18H), 2.20–2.26 (br, 2H), 7.07 (t, J = 2.0 Hz, 1H), 7.18–7.21 (m, 3H), 7.32–7.35 (m, 2H), 7.37 (d, J = 8.0 Hz, 1H), 7.43–7.46 (m, 3H), 7.51 (s, 2H), 7.59–7.66 (m, 4H), 7.68–7.72 (m, 3H), 7.91 (d, J = 8.5 Hz, 1H), 8.10 (d, J = 7.5 Hz,

1H), 8.16 (d, J = 8.5 Hz, 1H), 8.59 (d, J = 5.5 Hz, 1H), 9.69 (s, 1H). ^{13}C NMR (125 MHz, $CDCl_3$): δ (ppm) 23.95, 24.26, 29.11, 30.52, 30.88, 35.17, 35.50, 103.30, 110.46, 110.61, 113.56, 113.67, 114.38, 115.85 (d, $^2J_{CF}$ = 21.3 Hz), 116.29, 116.50, 118.36, 119.16, 120.09, 121.09, 121.13, 121.47, 123.85, 123.86, 125.94, 126.15, 128.65, 128.77, 129.17 (d, $^3J_{CF}$ = 8.8 Hz), 131.04, 132.66, 133.41, 136.24 (d, $^4J_{CF}$ = 2.5 Hz), 140.04, 140.79, 141.14, 144.38, 146.61, 149.48, 151.36, 154.39, 157.18, 159.63, 162.98 (d, $^1J_{CF}$ = 246.3Hz), 163.48. HRMS (ESI): calcd for $C_{56}H_{56}FN_4O$ $[M-PF_6]^+$ 819.4433, found 819.4445.

Synthesis of **PtCY-*t*BuF**: Ligand **LCY-*t*BuF** (10.00 g, 10.36 mmol, 1.00 equiv), $Pt(COD)Cl_2$ (4.07 g, 10.88 mmol, 1.05 equiv), and NaOAc (2.55 g, 31.09 mmol, 3.00 equiv) were added sequentially to a dry three-necked flask equipped with a magnetic stir bar. The flask was evacuated and backfilled with nitrogen, this evacuation and backfill procedure was repeated twice. Then DEDM (100 mL) was added and the mixture was stirred at 120 °C for 72 hours under a nitrogen atmosphere. After the reaction was completed, the mixture was cooled down to room temperature and extracted with dichloromethane three times, dried over Na_2SO_4, filtered, and the filtrate was concentrated under reduced pressure. The residue was purified through column chromatography on silica gel (eluent: petroleum ether/dichloromethane = 4:1–2:1) to obtain the desired product as yellow solid 9.02 g in 86% yield. ^{1}H NMR (500 MHz, $CDCl_3$): δ (ppm) 0.82–1.24 (br, 12H), 1.01 (s, 9H), 1.51 (s, 9H), 3.04–3.33 (br, 2H), 6.06 (dd, J = 6.0, 2.0 Hz, 1H), 7.00 (d, J = 8.0 Hz, 1H), 7.22 (d, J = 18.5 Hz, 1H), 7.23 (d, J = 8.5 Hz, 2H), 7.28 (t, J = 7.5 Hz, 1H), 7.32 (td, J = 7.5, 1.0 Hz, 1H), 7.36 (td, J = 7.0, 1.0 Hz, 1H), 7.41 (d, J = 8.0 Hz, 1H), 7.45–7.48 (m, 3H), 7.64 (td, J = 5.5, 2.5 Hz, 2H), 7.70 (d, J = 1.5 Hz, 1H), 7.72 (d, J = 8.0 Hz, 1H), 7.77 (d, J = 8.0 Hz, 1H), 7.88 (d, J = 1.5 Hz, 1H), 8.01 (dd, J = 2.0, 0.5 Hz, 1H), 8.18 (d, J = 8.0 Hz, 1H), 8.71 (d, J = 6.0 Hz, 1H). ^{13}C NMR (125 MHz, $CDCl_3$): δ (ppm) 22.84 (br), 25.08, 28.48, 29.92, 31.64, 34.89, 35.10, 106.12, 107.84, 111.45, 111.82, 112.03, 112.16, 112.93, 113.70, 113.89, 115.58, 115.87 (d, $^2J_{CF}$ = 21.3 Hz), 115.88, 116.49, 120.16, 122.32, 122.63, 123.00, 123.68, 124.76, 128.56 (d, $^3J_{CF}$ = 7.5 Hz), 129.34, 131.59, 132.06, 136.05 (d, $^4J_{CF}$ = 2.5 Hz), 137.60, 138.93, 141.55, 144.23, 147.69, 148.71, 148.77, 149.46, 153.48, 155.01, 155.29, 162.51, 162.85 (d, $^1J_{CF}$ = 246.3 Hz), 192.21. HRMS (ESI): calcd for $C_{56}H_{54}FN_4OPt$ $[M+H]^+$ 1012.3924, found 1012.3928.

Synthesis of **PtON-diPrPh**:

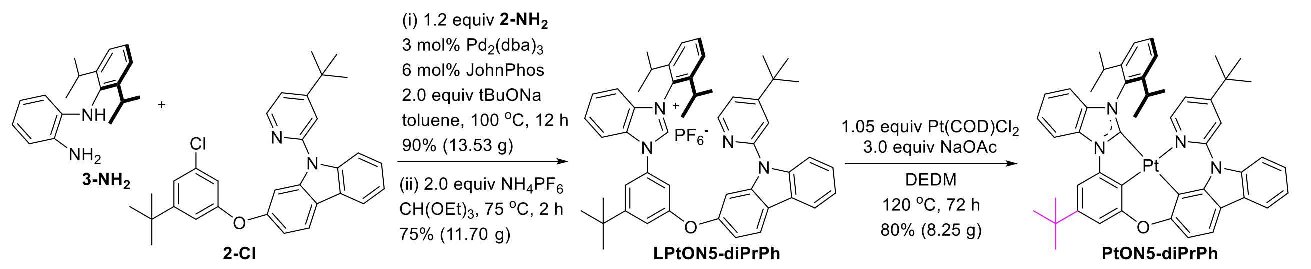

Syntesis of **LPtON5-diPrPh**: Compound 2-Cl (10.00 g, 20.70 mmol, 1.0 equiv), **3-NH2** (6.67 g, 24.84 mmol, 1.2 equiv), $Pd_2(dba)_3$ (569 mg, 0.62 mmol, 3 mol%), 2-(di-*tert*-butylphosphino)biphenyl (JohnPhos, 371 mg, 1.24 mmol, 6 mol%), and *t*-BuONa (3.98 g, 41.40 mmol, 2.0 equiv) were added sequentially to a dry three-necked flask equipped with a magnetic stir bar. The flask was evacuated and backfilled with nitrogen, this evacuation and backfill procedure was repeated twice. Then toluene (100 mL) was added and the mixture was heated at 100 °C under a nitrogen atmosphere for 12 hours. After the reaction was completed, the mixture was cooled to room temperature and concentrated under reduced pressure. The residue was purified through column chromatography on silica gel (eluent: petroleum ether/ethyl acetate = 30:1) to obtain the desired product diamine as powder solid 13.53 g in 90% yield. The intermediate diamine was not stable enough and easily oxidized by air, thereby, directly used for the next step. The diamine (13.00 g, 17.91 mmol, 1.0 equiv), and NH_4PF_6 (5.84 g, 35.81 mmol, 2.0 equiv) were added to dry three-necked flask equipped with a magnetic stir bar. Then $CH(OEt)_3$ (100 mL) was added and the mixture was heated at 75 °C under a nitrogen atmosphere for 2 hours. After the reaction was completed by TLC monitoring, the mixture was cooled down to room temperature. Then concentrated under reduced pressure. The residue was purified through column chromatography on silica gel (eluent: petroleum ether/dichloromethane = 1:1–dichloromethane/ethyl acetate = 50:1) to obtain the desired product as powder solid 11.70 g in 75% yield. ^{1}H NMR (500 MHz, $CDCl_3$): δ (ppm) 1.02 (d, J = 7.0 Hz, 6H), 1.23 (d, J = 7.0 Hz, 6H), 1.39 (s, 9H), 1.40 (s, 9H), 2.13–2.19 (m, 2H), 7.07 (t, J = 2.0 Hz, 1H), 7.18 (dd, J = 8.5, 2.0 Hz, 1H), 7.31 (d, J = 8.5 Hz, 1H), 7.32–7.35 (m, 2H), 7.41 (d, J = 7.5 Hz, 2H), 7.43–7.46 (m, 3H), 7.61 (d, J = 1.5 Hz, 1H), 7.63 (d, J = 8.0 Hz, 2H), 7.65–7.70 (m, 3H), 7.91 (d, J = 8.0 Hz, 1H), 8.10 (d, J = 7.5 Hz, 1H), 8.15 (d, J = 8.5 Hz, 1H), 8.59 (d, J = 5.5 Hz, 1H), 9.68 (s, 1H). ^{13}C NMR (125 MHz, $CDCl_3$): δ (ppm) 23.91, 24.23, 28.99, 30.53, 30.90, 35.16, 35.52, 103.30, 110.43, 110.62, 113.54, 114.33, 116.30, 116.48, 118.33, 119.16,

120.10, 121.09, 121.13, 121.47, 123.88, 125.12, 126.94, 128.55, 128.68, 130.98, 132.48, 132.70, 133.43, 140.07, 140.82, 141.10, 146.11, 149.54, 151.41, 154.40, 157.23, 159.64, 163.44.

Synthesis of **PtON5-diPrPh**: Ligand **LPtON5-diPrPh** (10.00 g, 11.48 mmol, 1.00 equiv), Pt(COD)Cl_2 (4.51 g, 12.06 mmol, 1.05 equiv), and NaOAc (2.83 g, 34.44 mmol, 3.00 equiv) were added sequentially to a dry three-necked flask equipped with a magnetic stir bar. The flask was evacuated and backfilled with nitrogen, this evacuation and backfill procedure was repeated twice. Then DEDM (100 mL) was added and the mixture was stirred at 120 °C for 72 hours under a nitrogen atmosphere. After the reaction was completed, the mixture was cooled down to room temperature and extracted with dichloromethane three times, dried over Na_2SO_4, filtered, and the filtrate was concentrated under reduced pressure. The residue was purified through column chromatography on silica gel (eluent: petroleum ether/dichloromethane = 4:1–2:1) to obtain the desired product as yellow solid 8.25 g in 80% yield. ^{1}H NMR (500 MHz, $CDCl_3$): δ (ppm) 0.90 (s, 6H), 1.13 (s, 6H), 1.26 (s, 9H), 1.51 (s, 9H), 2.84–3.34 (m, 2H), 6.04 (dd, J = 6.0, 1.5 Hz, 1H), 6.94(d, J = 8.0 Hz, 1H), 7.19–7.23 (br, 1H), 7.24(d, J = 1.5 Hz, 1H), 7.25–7.28 (m, 2H), 7.31(td, J = 7.5, 0.5 Hz, 1H), 7.35–7.41 (m, 3H), 7.43–7.46 (m, 1H), 7.69 (d, J = 1.5 Hz, 1H), 7.76 (d, J = 8.0 Hz, 2H), 7.94 (d, J = 1.5 Hz, 1H), 7.80 (dd, J = 7.5, 0.5 Hz, 1H), 8.16 (d, J = 8.5 Hz, 1H), 8.163 (d, J = 6.0 Hz, 1H). ^{13}C NMR (125 MHz, $CDCl_3$): δ (ppm) 25.07, 28.32, 30.03, 31.65, 34.89, 35.20, 106.09, 108.24, 111.14, 111.39, 111.74, 112.14, 112.95, 114.10, 114.25, 115.86, 115.87, 116.39, 120.09, 122.25, 122.91, 123.63, 124.34, 124.65, 129.39, 130.43, 131.55, 132.81, 137.60, 138.89, 144.02, 147.17, 148.31, 148.70, 149.57, 153.44, 154.55, 155.21, 161.89, 192.27.

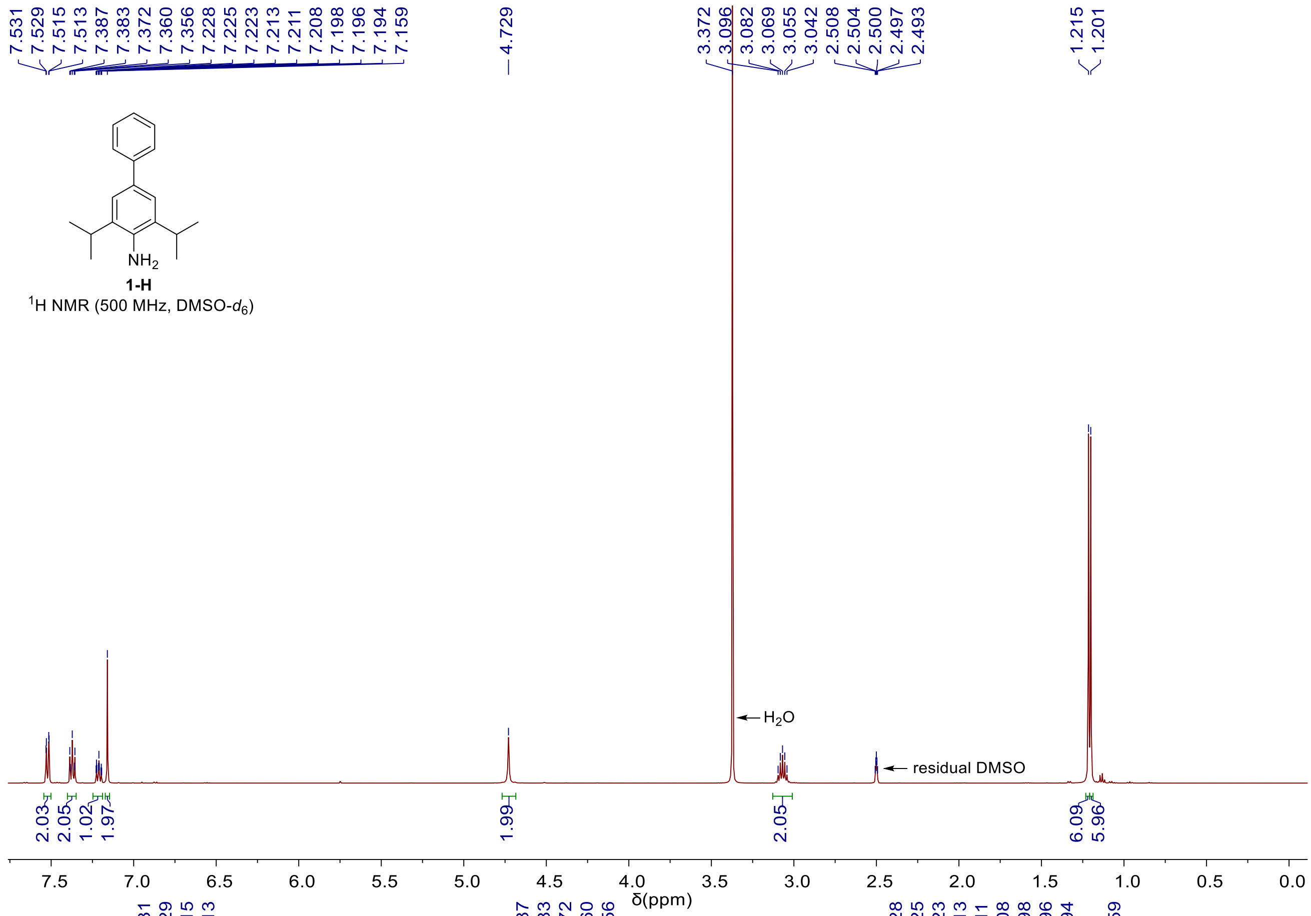
7.531 7.529 7.515 7.513 7.387 7.383 7.372 7.360 7.356 7.228 7.225 7.223 7.213 7.211 7.208 7.198 7.196 7.194 7.159
4.729
3.372 3.096 3.082 3.069 3.055 3.042 2.508 2.504 2.500 2.497 2.493
1.215 1.201
NH_2
1-H
^1H NMR (500 MHz, DMSO-d_6)
H_2O
residual DMSO
2.03 2.05 1.02 1.97 1.99 2.05 6.09 5.96
δ(ppm)

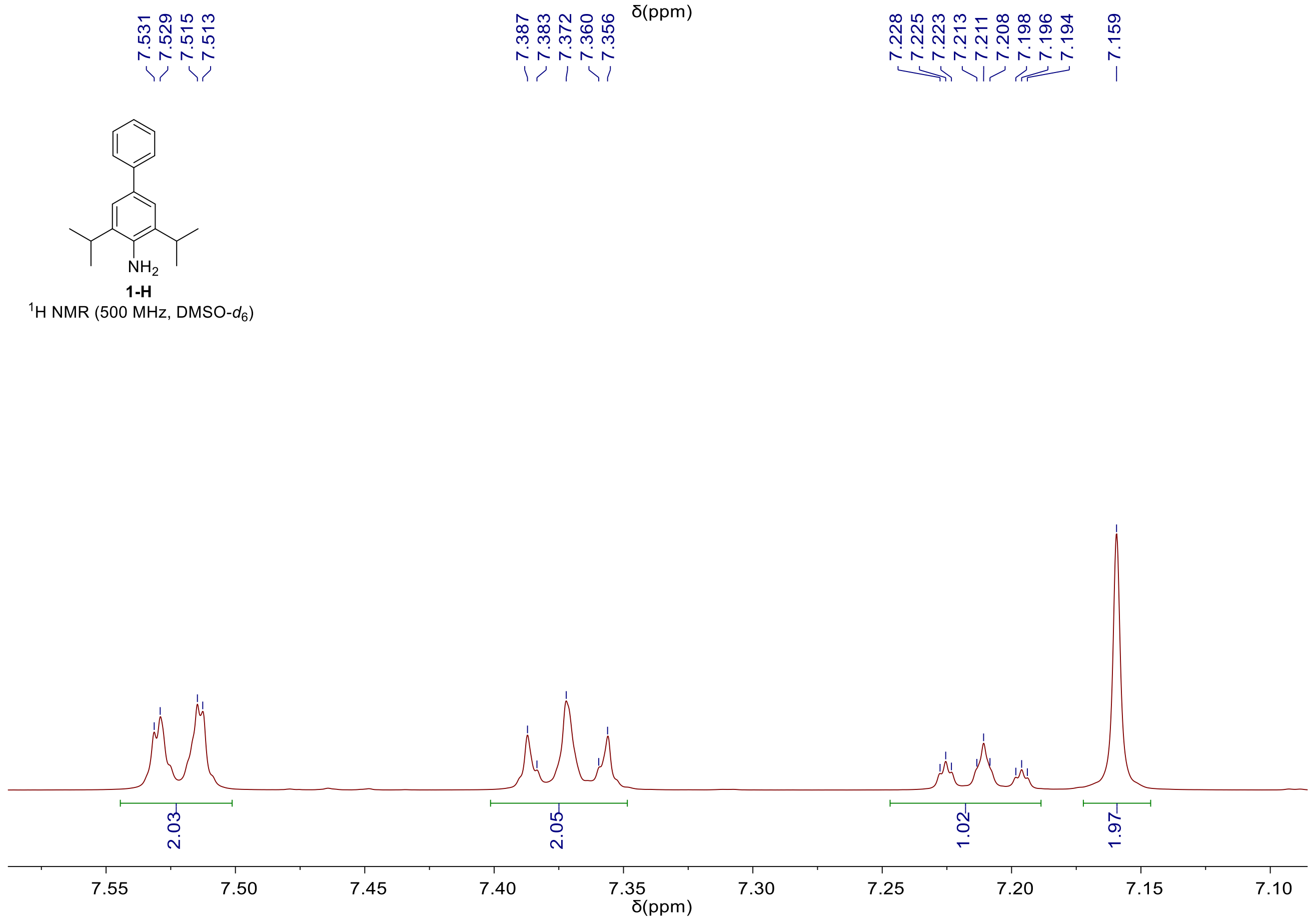
7.531 7.529 7.515 7.513
7.387 7.383 7.372 7.360 7.356
7.228 7.225 7.223 7.213 7.211 7.208 7.198 7.196 7.194
7.159
NH_2
1-H
^1H NMR (500 MHz, DMSO-d_6)
2.03 2.05 1.02 1.97
δ(ppm)

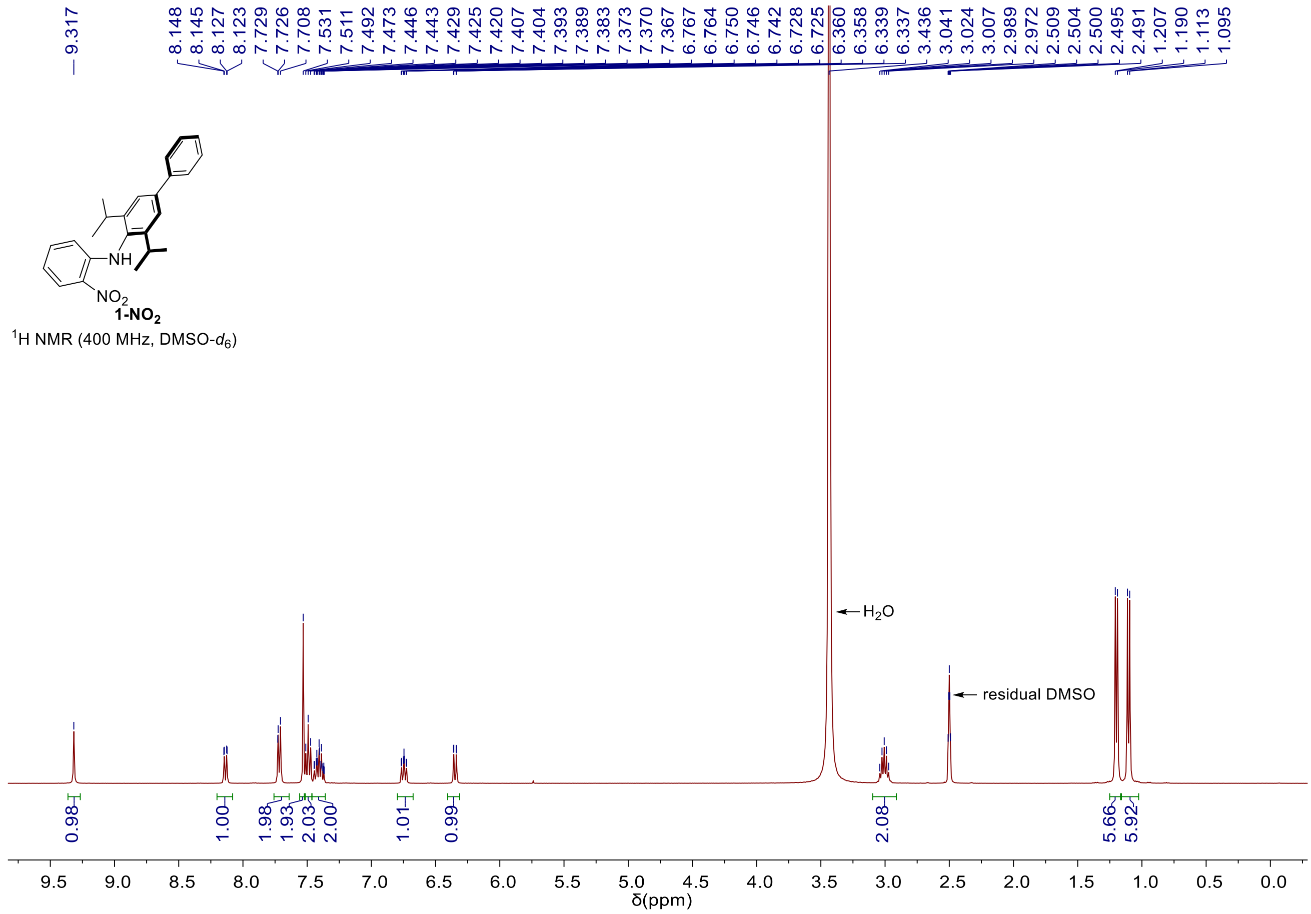
NH
NO2
1-NO2
1H NMR (400 MHz, DMSO-d6)
H2O
residual DMSO
δ(ppm)

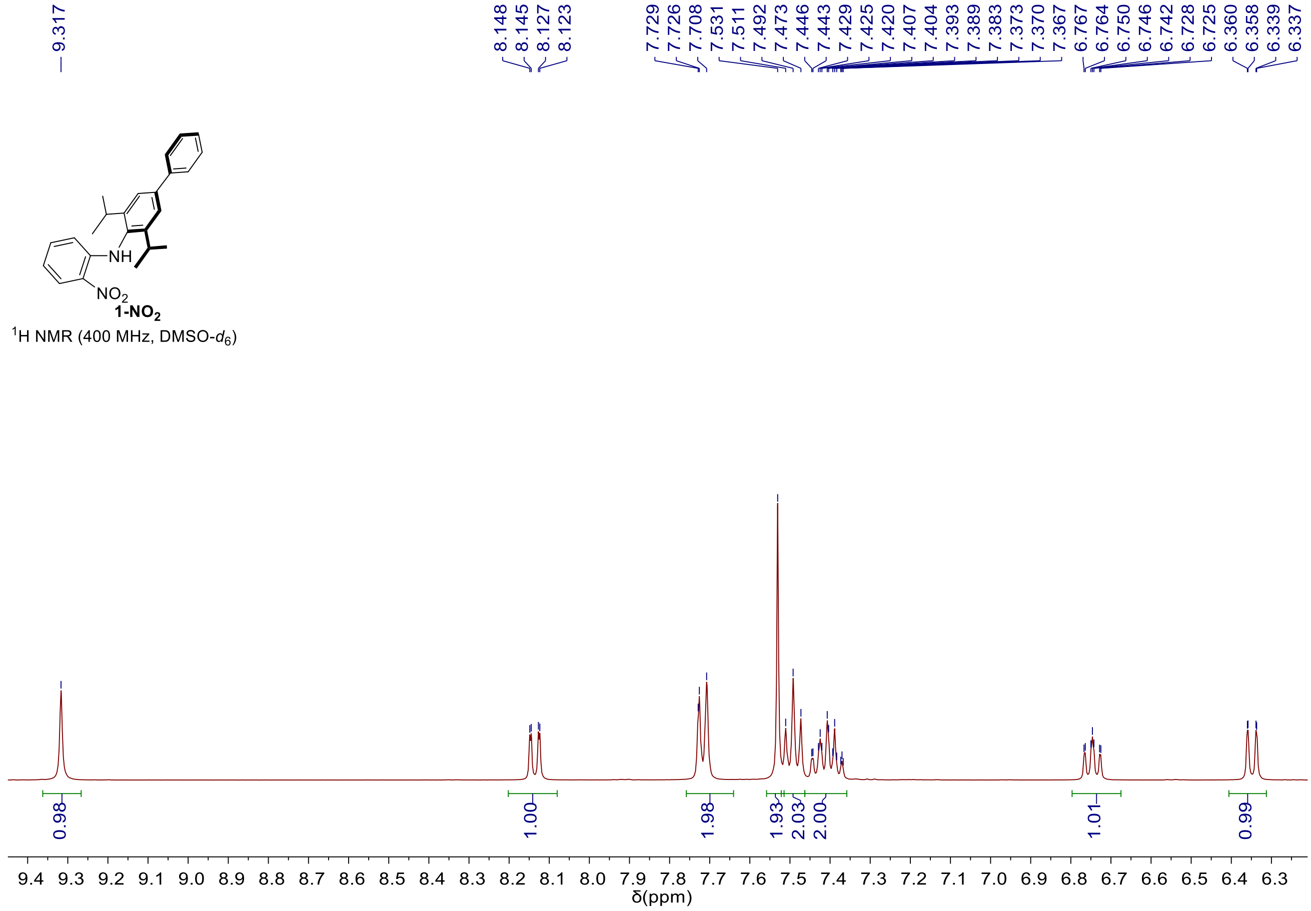
NH
NO2
1-NO2
1H NMR (400 MHz, DMSO-d6)
δ(ppm)

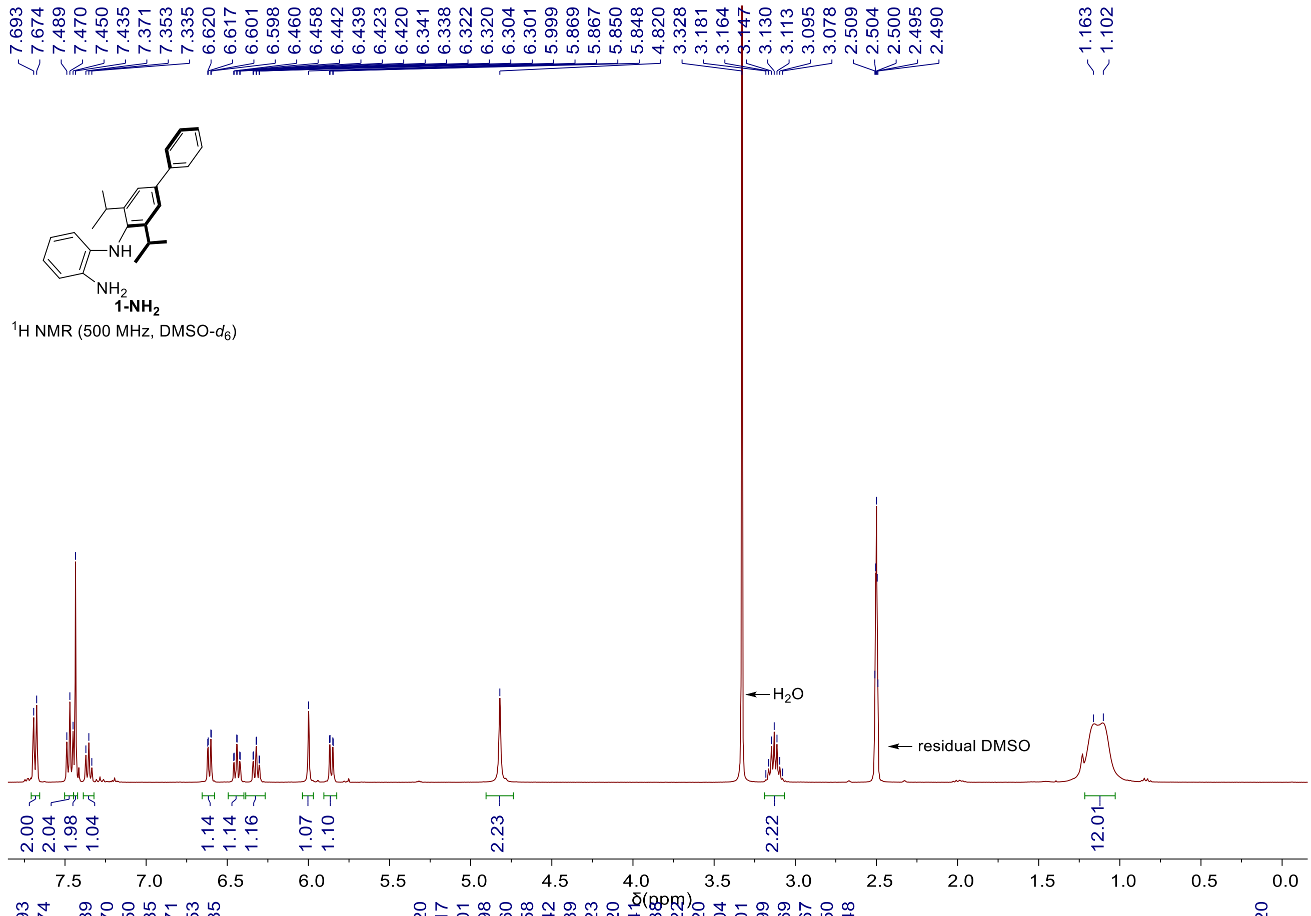
7.693 7.674 7.489 7.470 7.450 7.435 7.371 7.353 7.335 6.620 6.617 6.601 6.598 6.460 6.458 6.442 6.439 6.423 6.420 6.341 6.338 6.322 6.320 6.304 6.301 5.999 5.869 5.867 5.850 5.848 4.820 3.328 3.181 3.164 3.147 3.130 3.113 3.095 3.078 2.509 2.504 2.500 2.495 2.490 1.163 1.102
NH
NH2
1-NH2
1H NMR (500 MHz, DMSO-d6)
H2O
residual DMSO
2.00 2.04 1.98 1.04 1.14 1.14 1.16 1.07 1.10 2.23 2.22 12.01
δ(ppm)

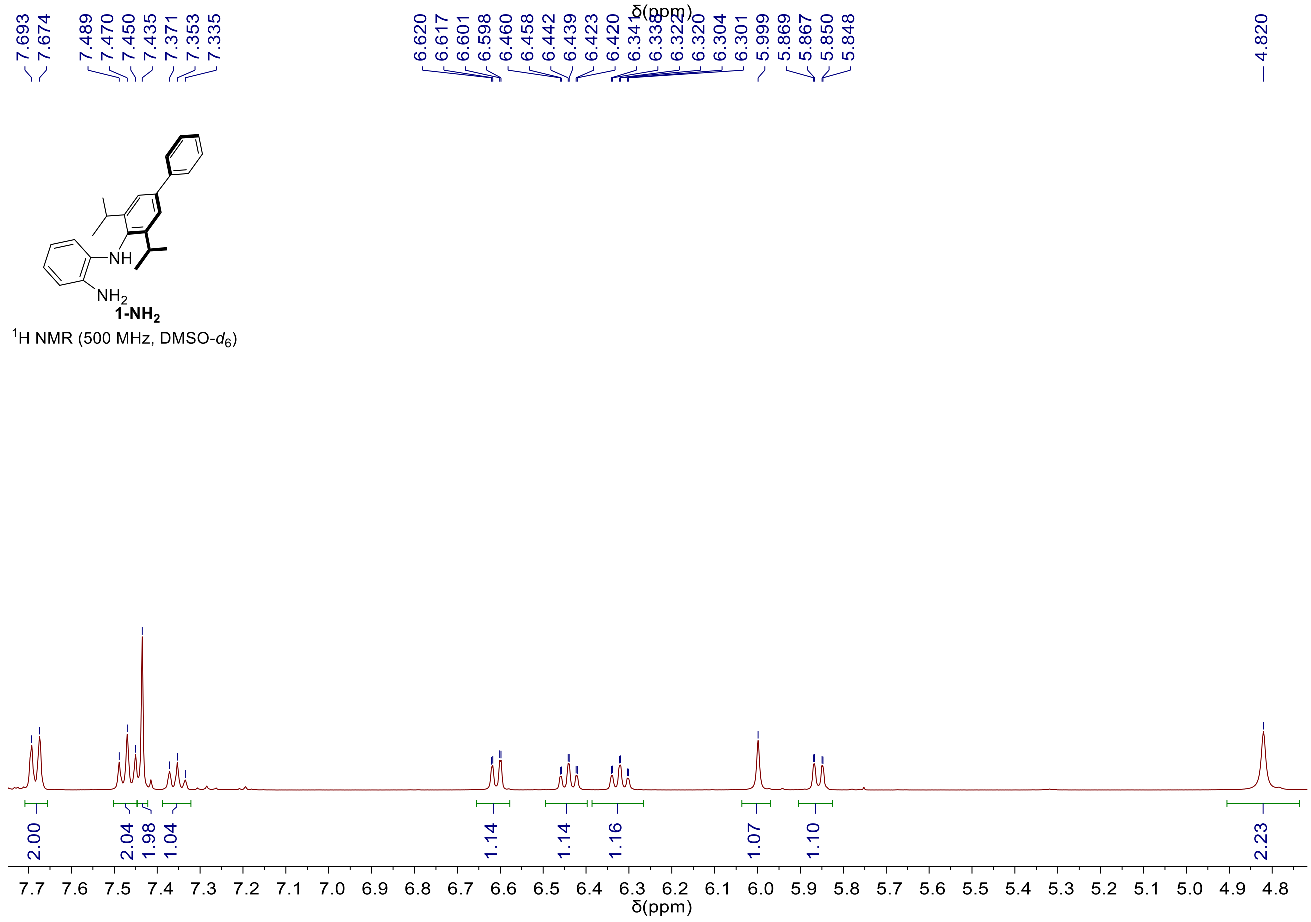
7.693 7.674 7.489 7.470 7.450 7.435 7.371 7.353 7.335 6.620 6.617 6.601 6.598 6.460 6.458 6.442 6.439 6.423 6.420 6.341 6.338 6.322 6.320 6.304 6.301 5.999 5.869 5.867 5.850 5.848 4.820
NH
NH2
1-NH2
1H NMR (500 MHz, DMSO-d6)
2.00 2.04 1.98 1.04 1.14 1.14 1.16 1.07 1.10 2.23
δ(ppm)

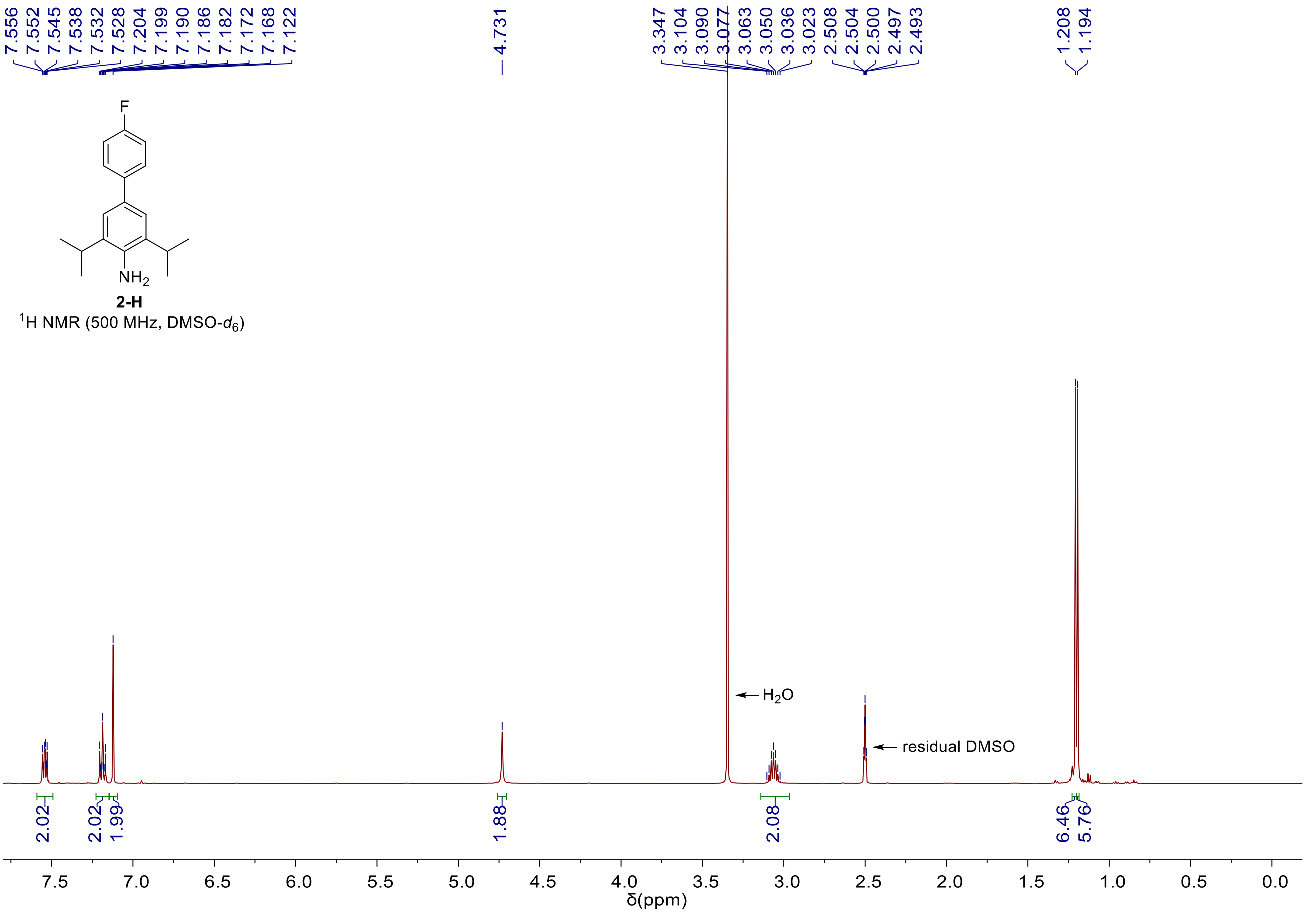
7.556 7.552 7.545 7.538 7.532 7.528 7.204 7.199 7.190 7.186 7.182 7.172 7.168 7.122
4.731
3.347 3.104 3.090 3.077 3.063 3.050 3.036 3.023 2.508 2.504 2.500 2.497 2.493
1.208 1.194
F
NH2
2-H
1H NMR (500 MHz, DMSO-d6)
H2O
residual DMSO
2.02 2.02 1.99 1.88 2.08 6.46 5.76
7.5 7.0 6.5 6.0 5.5 5.0 4.5 4.0 3.5 3.0 2.5 2.0 1.5 1.0 0.5 0.0
δ(ppm)

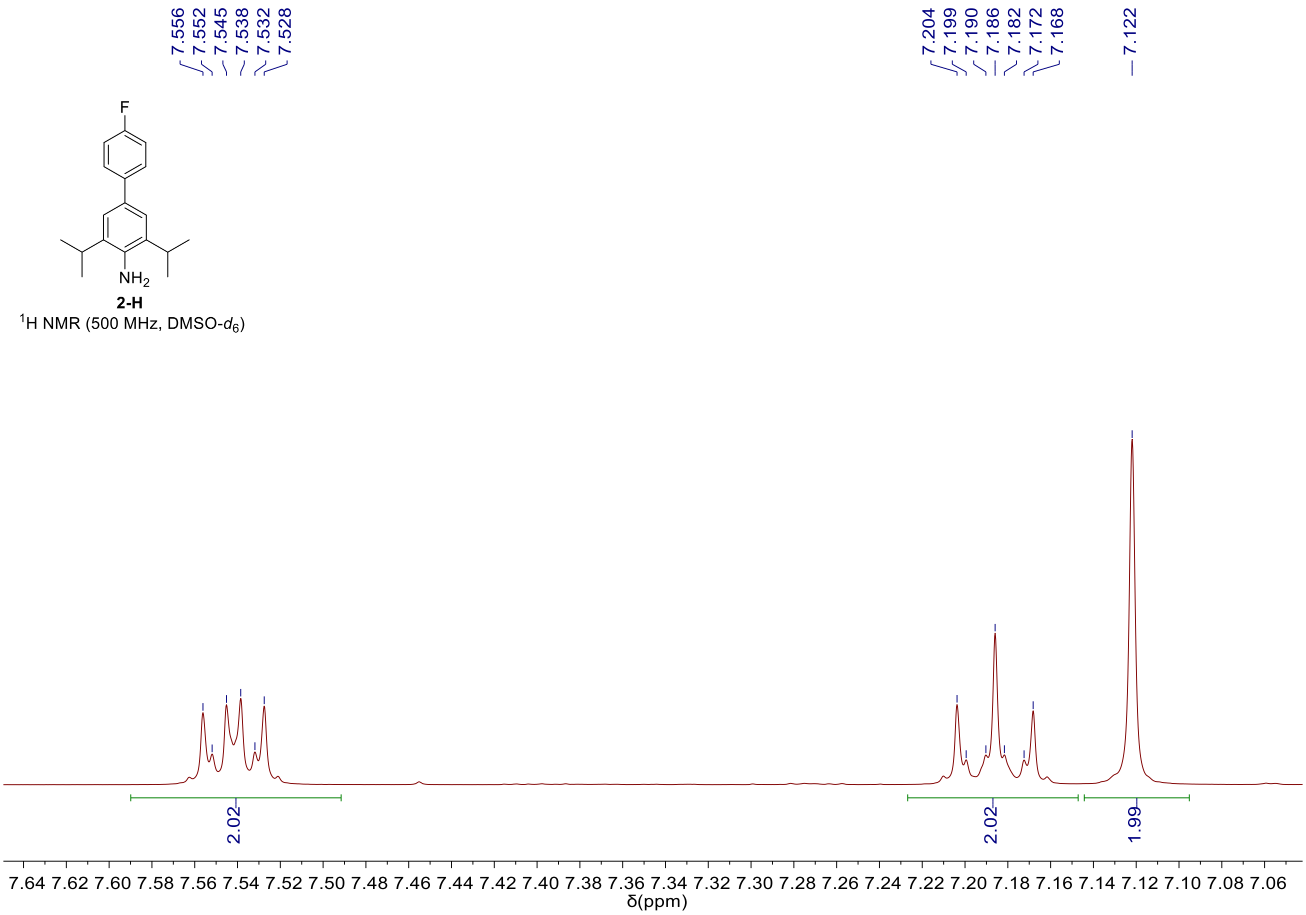
7.556 7.552 7.545 7.538 7.532 7.528
7.204 7.199 7.190 7.186 7.182 7.172 7.168
7.122
F
NH2
2-H
1H NMR (500 MHz, DMSO-d6)
2.02 2.02 1.99
7.64 7.62 7.60 7.58 7.56 7.54 7.52 7.50 7.48 7.46 7.44 7.42 7.40 7.38 7.36 7.34 7.32 7.30 7.28 7.26 7.24 7.22 7.20 7.18 7.16 7.14 7.12 7.10 7.08 7.06
δ(ppm)

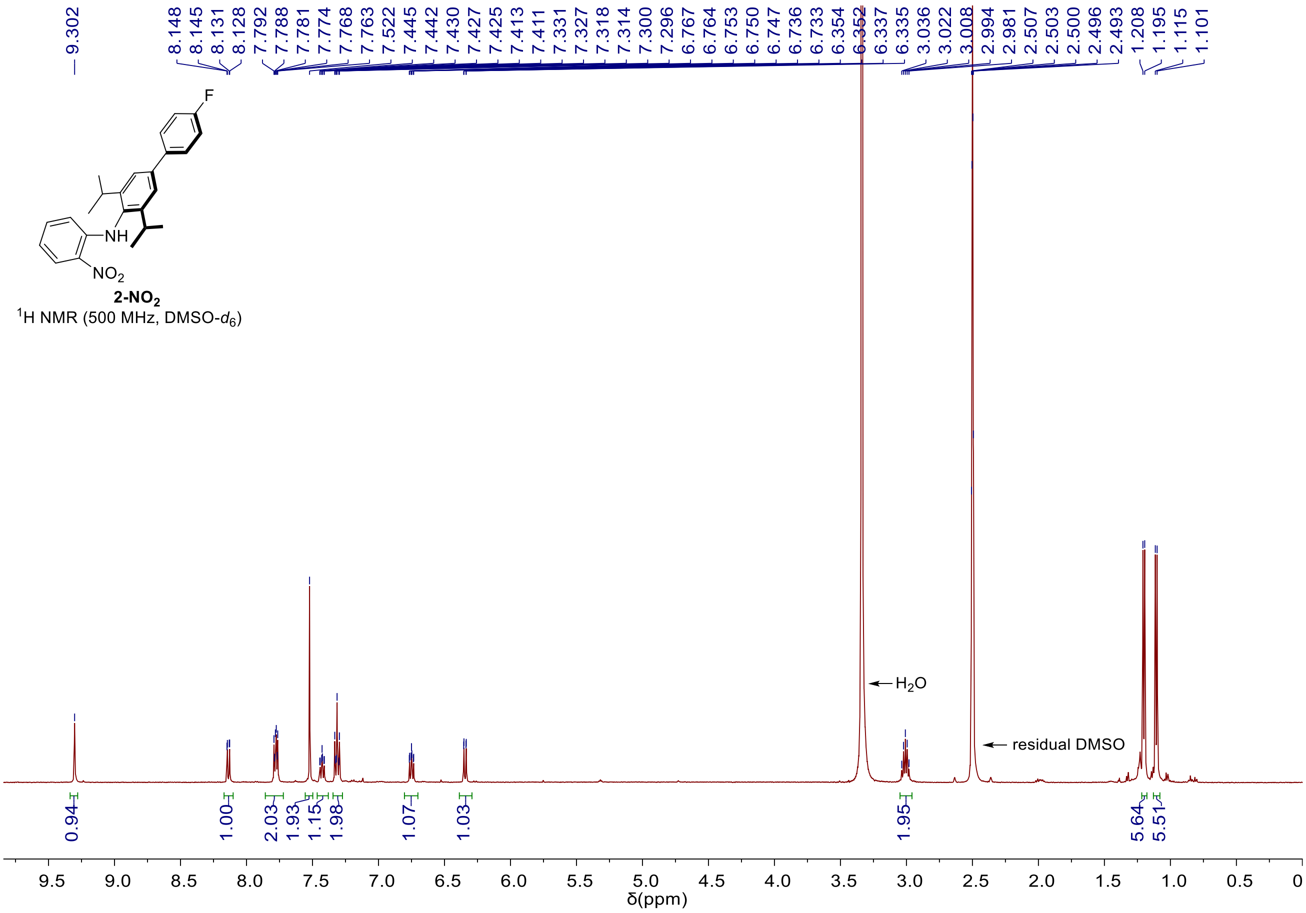
2-NO2
1H NMR (500 MHz, DMSO-d6)
H2O
residual DMSO
δ(ppm)

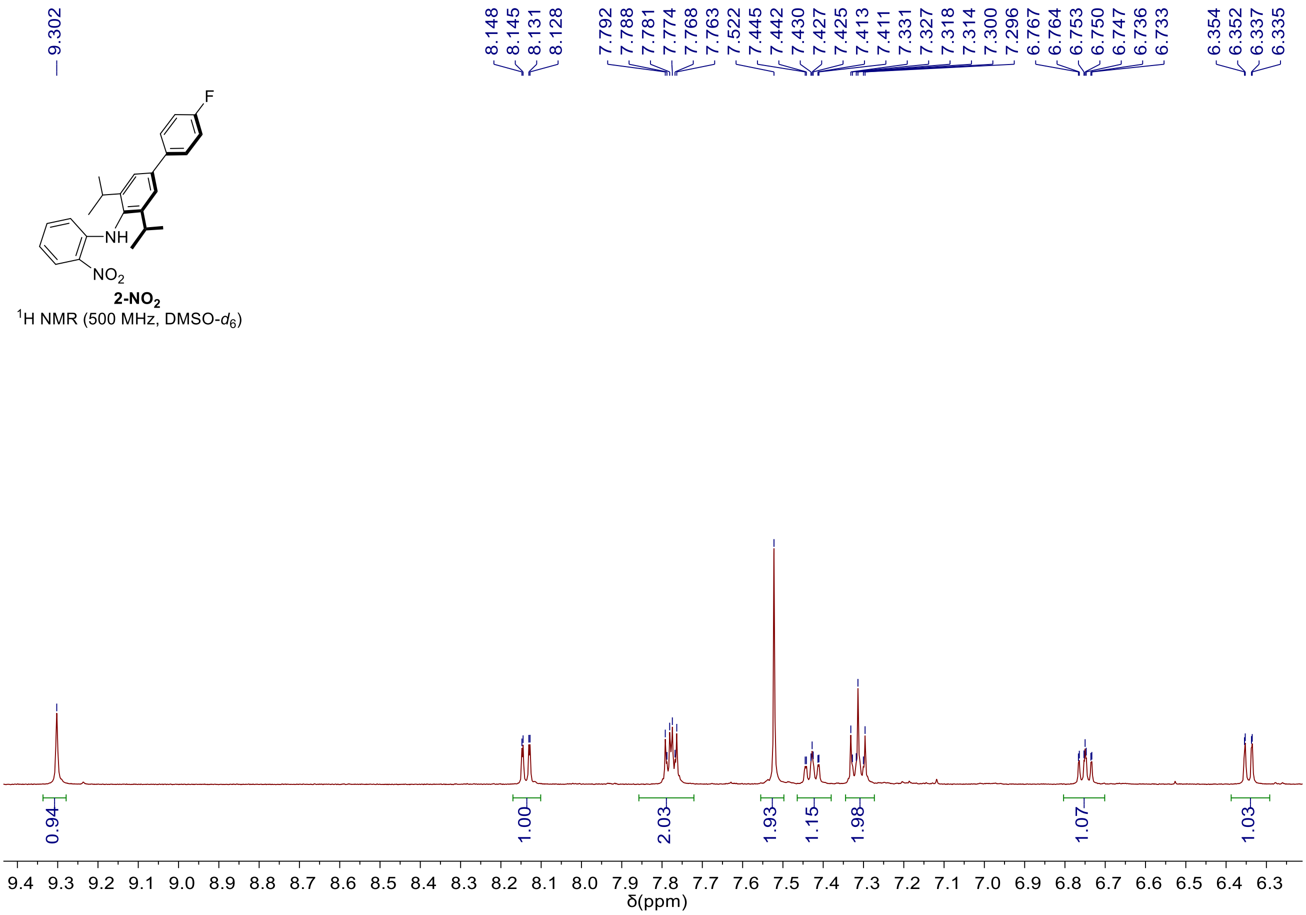
2-NO2
1H NMR (500 MHz, DMSO-d6)
δ(ppm)

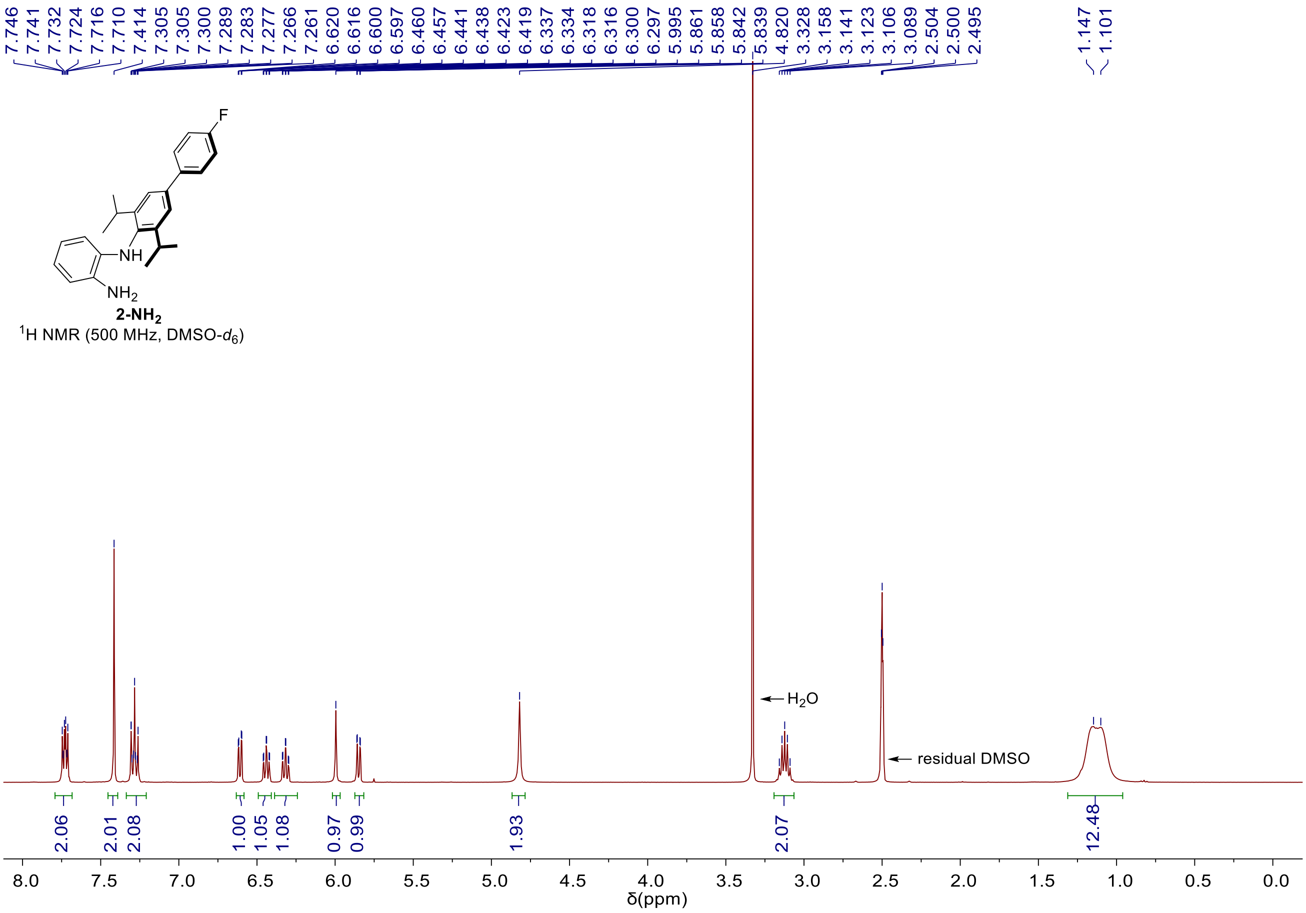
7.746 7.741 7.732 7.724 7.716 7.710 7.414 7.305 7.300 7.289 7.283 7.277 7.266 7.261 6.620 6.616 6.600 6.597 6.460 6.457 6.441 6.438 6.423 6.419 6.337 6.334 6.318 6.316 6.300 6.297 5.995 5.861 5.858 5.842 5.839 4.820 3.328 3.158 3.141 3.123 3.106 3.089 2.504 2.500 2.495
1.147 1.101
F
NH
NH_2
2-NH_2
^1H NMR (500 MHz, DMSO-d_6)
← H_2O
← residual DMSO
2.06 2.01 2.08 1.00 1.05 1.08 0.97 0.99 1.93 2.07 12.48
8.0 7.5 7.0 6.5 6.0 5.5 5.0 4.5 4.0 3.5 3.0 2.5 2.0 1.5 1.0 0.5 0.0
δ(ppm)

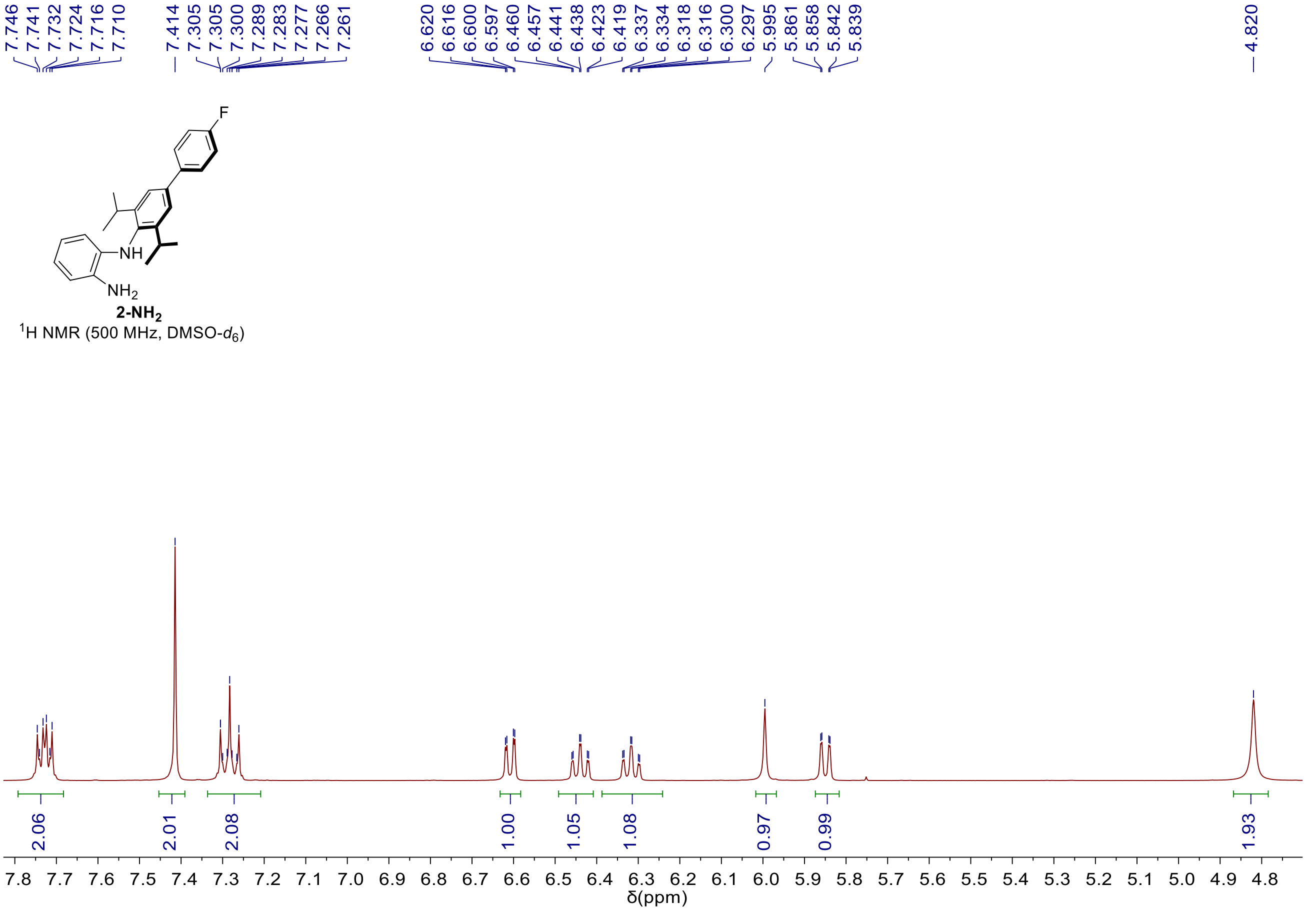
7.746 7.741 7.732 7.724 7.716 7.710 7.414 7.305 7.300 7.289 7.283 7.277 7.266 7.261 6.620 6.616 6.600 6.597 6.460 6.457 6.441 6.438 6.423 6.419 6.337 6.334 6.318 6.316 6.300 6.297 5.995 5.861 5.858 5.842 5.839 4.820
F
NH
NH_2
2-NH_2
^1H NMR (500 MHz, DMSO-d_6)
2.06 2.01 2.08 1.00 1.05 1.08 0.97 0.99 1.93
7.8 7.7 7.6 7.5 7.4 7.3 7.2 7.1 7.0 6.9 6.8 6.7 6.6 6.5 6.4 6.3 6.2 6.1 6.0 5.9 5.8 5.7 5.6 5.5 5.4 5.3 5.2 5.1 5.0 4.9 4.8
δ(ppm)

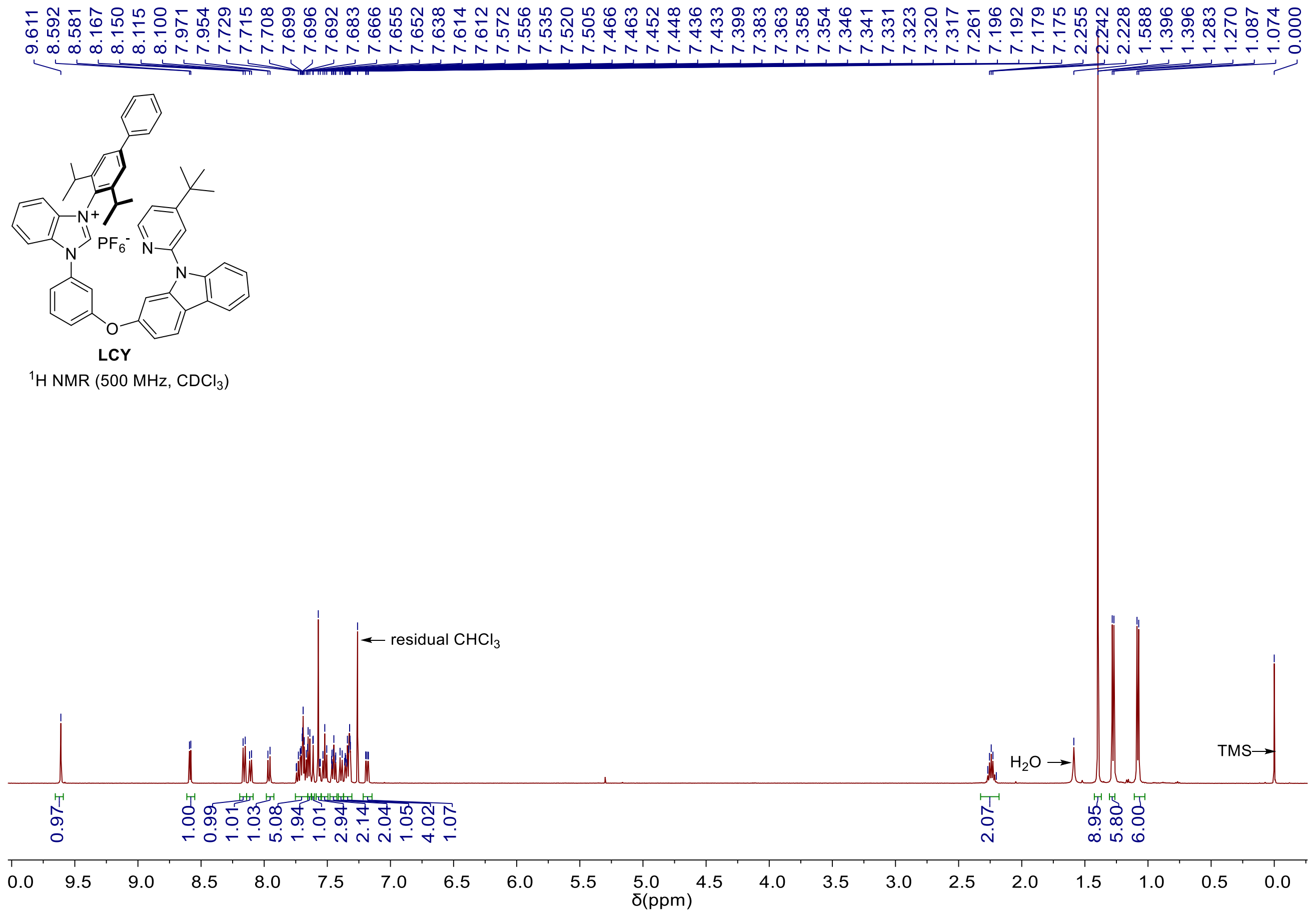
9.611 8.592 8.581 8.167 8.150 8.115 8.100 7.971 7.954 7.729 7.715 7.708 7.699 7.696 7.692 7.683 7.666 7.655 7.652 7.638 7.614 7.612 7.572 7.556 7.535 7.520 7.505 7.466 7.463 7.452 7.448 7.436 7.433 7.399 7.383 7.363 7.358 7.354 7.346 7.341 7.331 7.323 7.320 7.317 7.261 7.196 7.192 7.179 7.175 2.255 2.242 2.228 1.588 1.396 1.396 1.283 1.270 1.087 1.074 0.000
LCY
^{1}H NMR (500 MHz, $CDCl_3$)
residual $CHCl_3$
H_2O
TMS
0.97 1.00 0.99 1.01 1.03 5.08 1.94 1.01 2.94 2.14 2.04 1.05 4.02 1.07 2.07 8.95 5.80 6.00
0.0 9.5 9.0 8.5 8.0 7.5 7.0 6.5 6.0 5.5 5.0 4.5 4.0 3.5 3.0 2.5 2.0 1.5 1.0 0.5 0.0
δ(ppm)

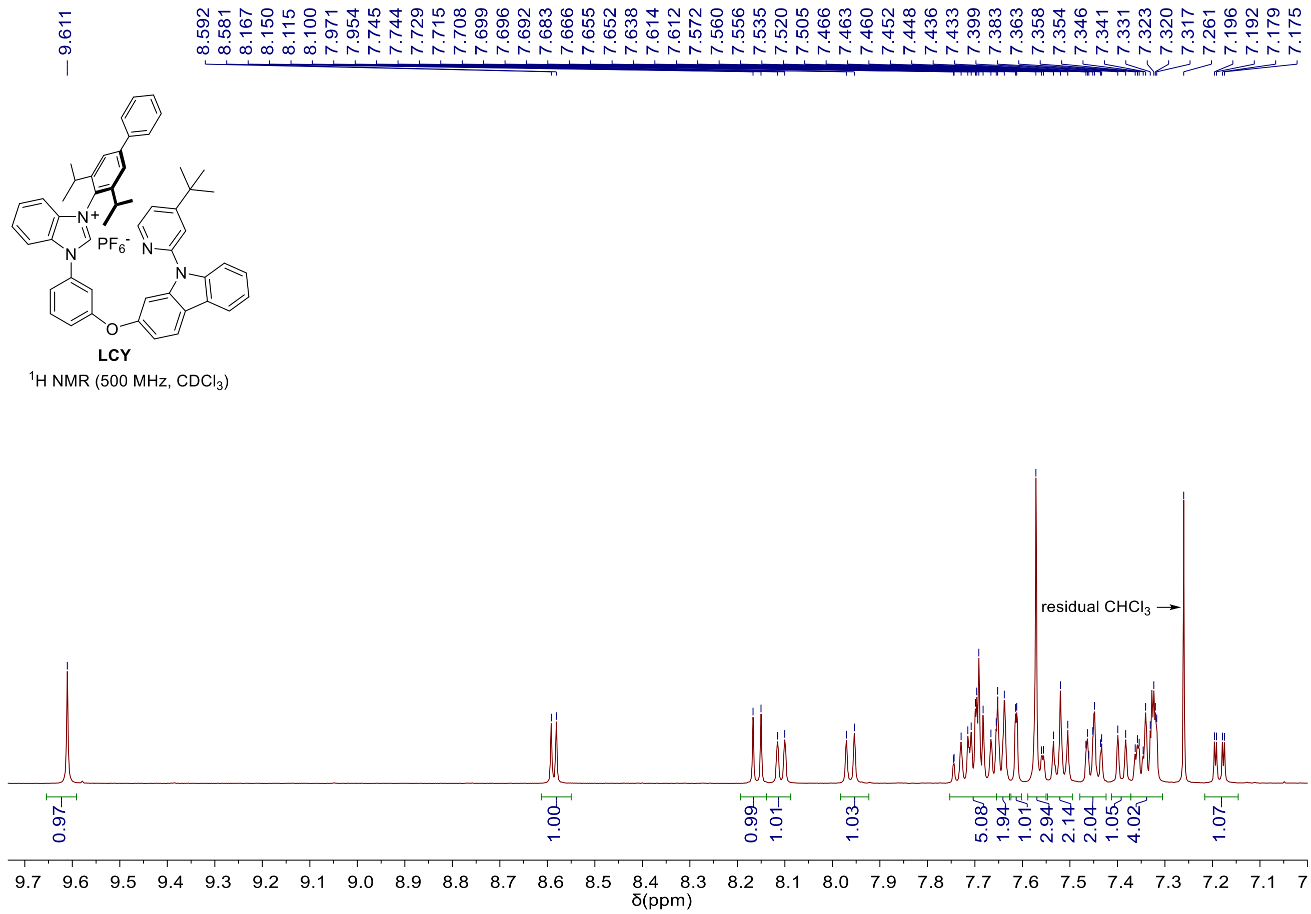
9.611 8.592 8.581 8.167 8.150 8.115 8.100 7.971 7.954 7.745 7.744 7.729 7.715 7.708 7.699 7.696 7.692 7.683 7.666 7.655 7.652 7.638 7.614 7.612 7.572 7.560 7.556 7.535 7.520 7.505 7.466 7.463 7.460 7.452 7.448 7.436 7.433 7.399 7.383 7.363 7.358 7.354 7.346 7.341 7.331 7.323 7.320 7.317 7.261 7.196 7.192 7.179 7.175
LCY
^{1}H NMR (500 MHz, $CDCl_3$)
residual $CHCl_3$
0.97 1.00 0.99 1.01 1.03 5.08 1.94 1.01 2.94 2.14 2.04 1.05 4.02 1.07
9.7 9.6 9.5 9.4 9.3 9.2 9.1 9.0 8.9 8.8 8.7 8.6 8.5 8.4 8.3 8.2 8.1 8.0 7.9 7.8 7.7 7.6 7.5 7.4 7.3 7.2 7.1 7
δ(ppm)

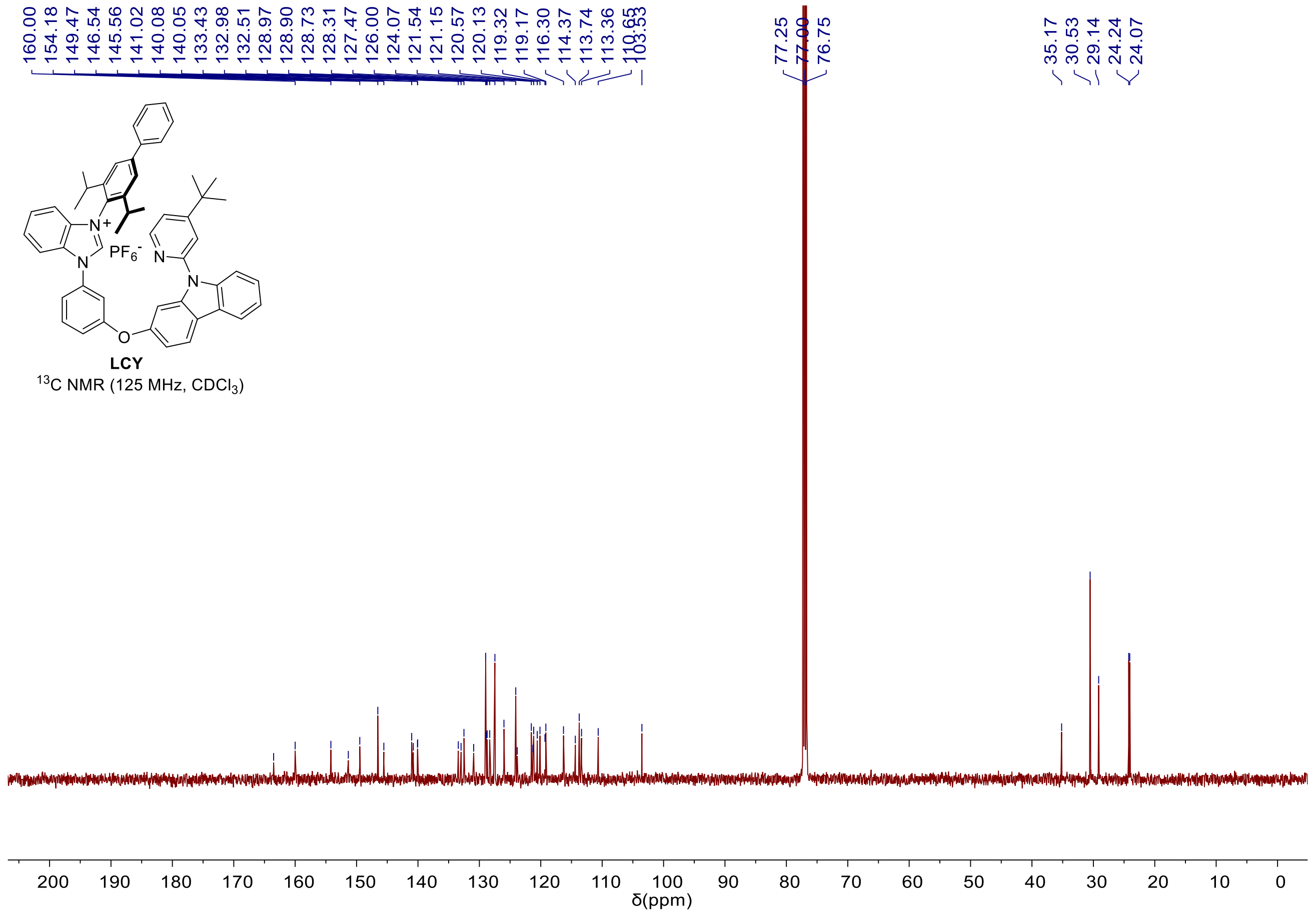
160.00
154.18
149.47
146.54
145.56
141.02
140.08
140.05
133.43
132.98
132.51
128.97
128.90
128.73
128.31
127.47
126.00
124.07
121.54
121.15
120.57
120.13
119.32
119.17
116.30
114.37
113.74
113.36
110.65
103.53
77.25
77.00
76.75
35.17
30.53
29.14
24.24
24.07
PF6-
N
O
LCY
13C NMR (125 MHz, CDCl3)
200 190 180 170 160 150 140 130 120 110 100 90 80 70 60 50 40 30 20 10 0
δ(ppm)

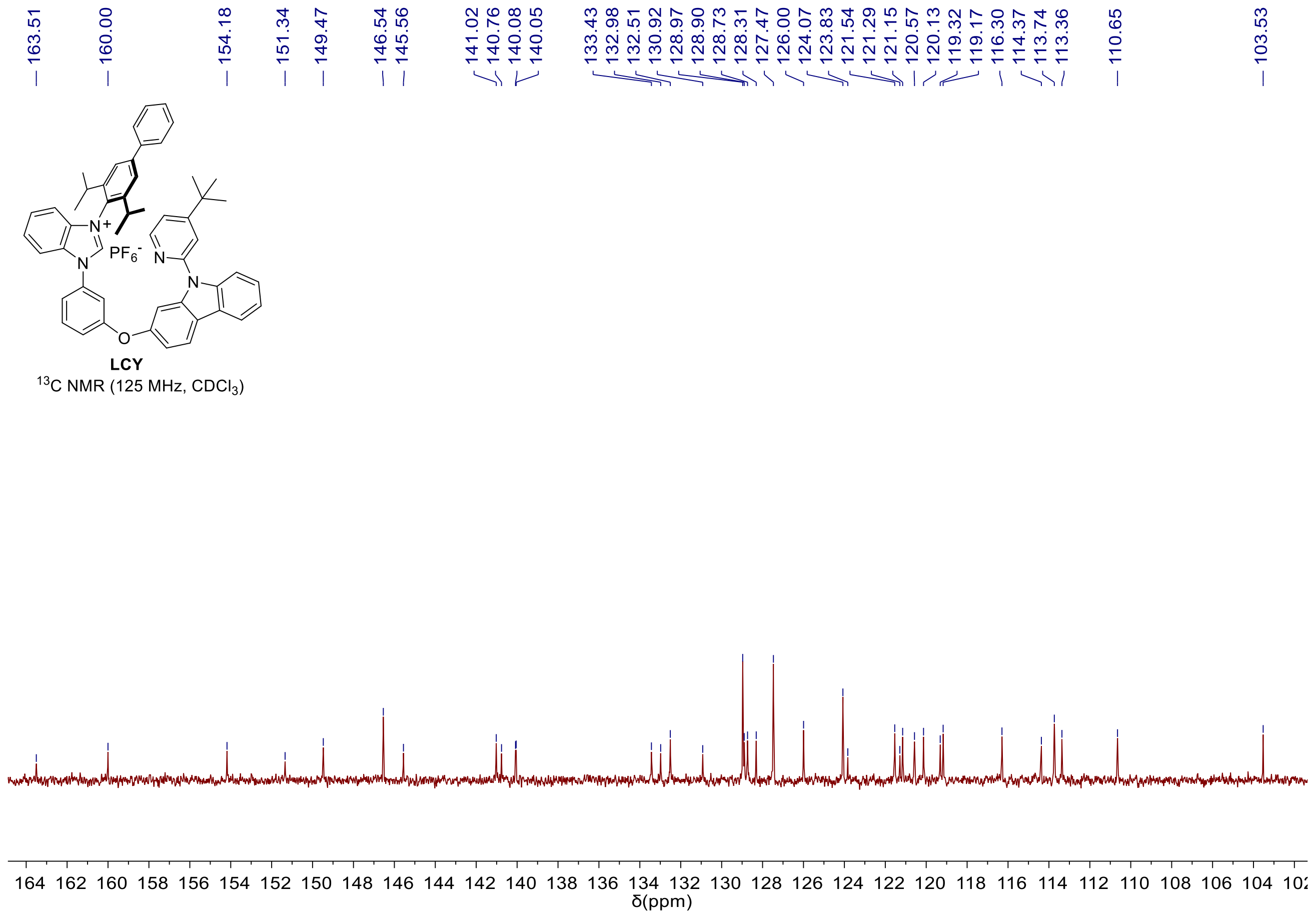
163.51
160.00
154.18
151.34
149.47
146.54
145.56
141.02
140.76
140.08
140.05
133.43
132.98
132.51
130.92
128.97
128.90
128.73
128.31
127.47
126.00
124.07
123.83
121.54
121.29
121.15
120.57
120.13
119.32
119.17
116.30
114.37
113.74
113.36
110.65
103.53
PF6-
N
O
LCY
13C NMR (125 MHz, CDCl3)
164 162 160 158 156 154 152 150 148 146 144 142 140 138 136 134 132 130 128 126 124 122 120 118 116 114 112 110 108 106 104
δ(ppm)

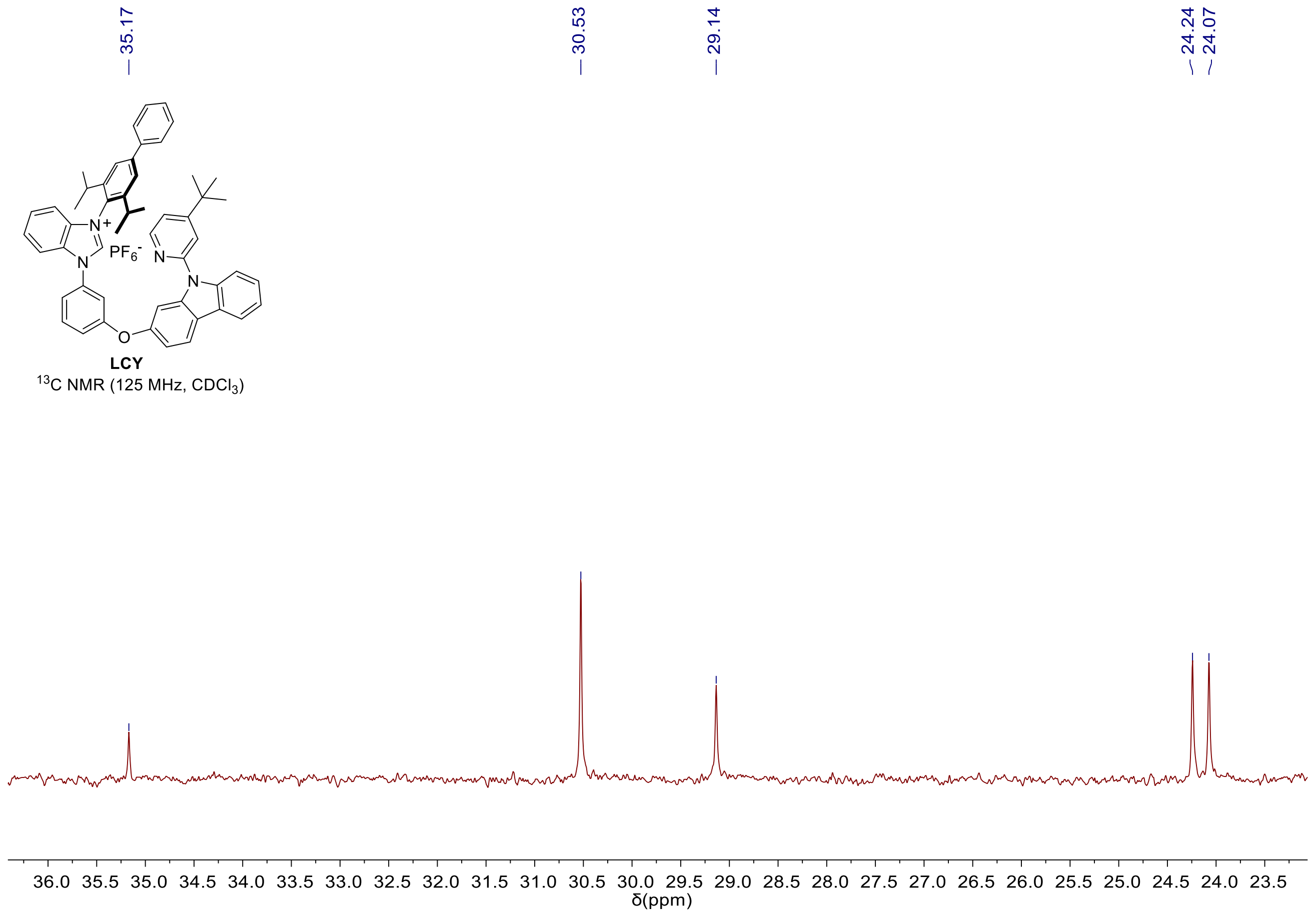
35.17
30.53
29.14
24.24
24.07
N+
PF6-
N
N
N
O
LCY
13C NMR (125 MHz, CDCl3)
36.0 35.5 35.0 34.5 34.0 33.5 33.0 32.5 32.0 31.5 31.0 30.5 30.0 29.5 29.0 28.5 28.0 27.5 27.0 26.5 26.0 25.5 25.0 24.5 24.0 23.5
δ(ppm)

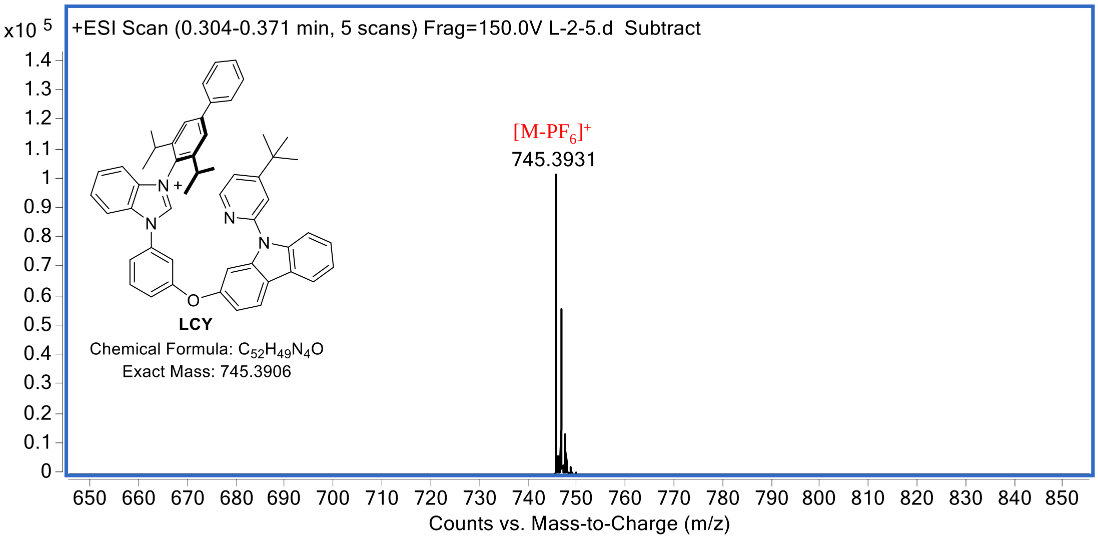
x10 5
+ESI Scan (0.304-0.371 min, 5 scans) Frag=150.0V L-2-5.d Subtract
[M-PF6]+
745.3931
LCY
Chemical Formula: C52H49N4O
Exact Mass: 745.3906
1.4
1.3
1.2
1.1
1
0.9
0.8
0.7
0.6
0.5
0.4
0.3
0.2
0.1
0
650 660 670 680 690 700 710 720 730 740 750 760 770 780 790 800 810 820 830 840 850
Counts vs. Mass-to-Charge (m/z)

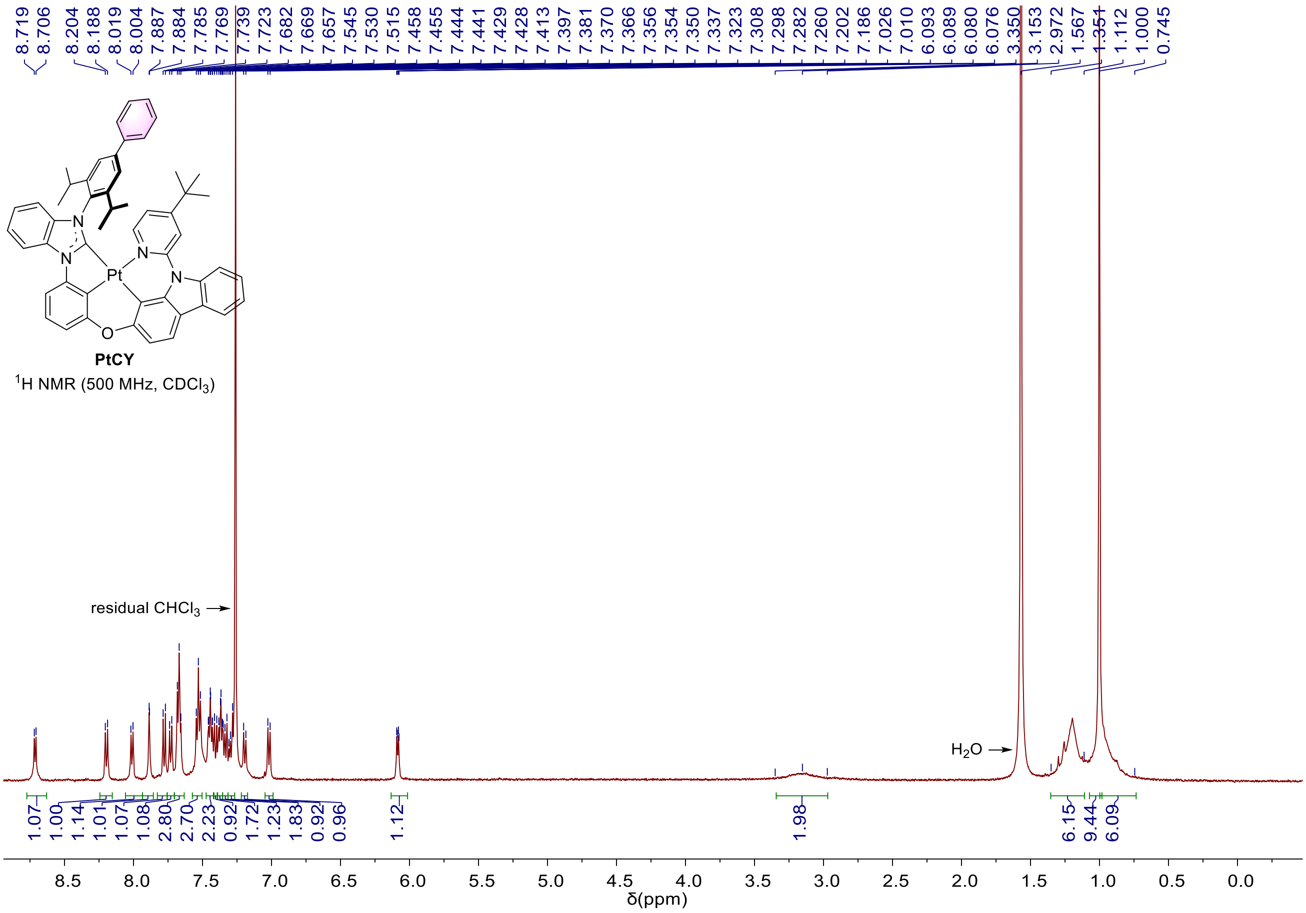
PtCY
^{1}H NMR (500 MHz, $CDCl_3$)
residual $CHCl_3$
H_2O
δ(ppm)

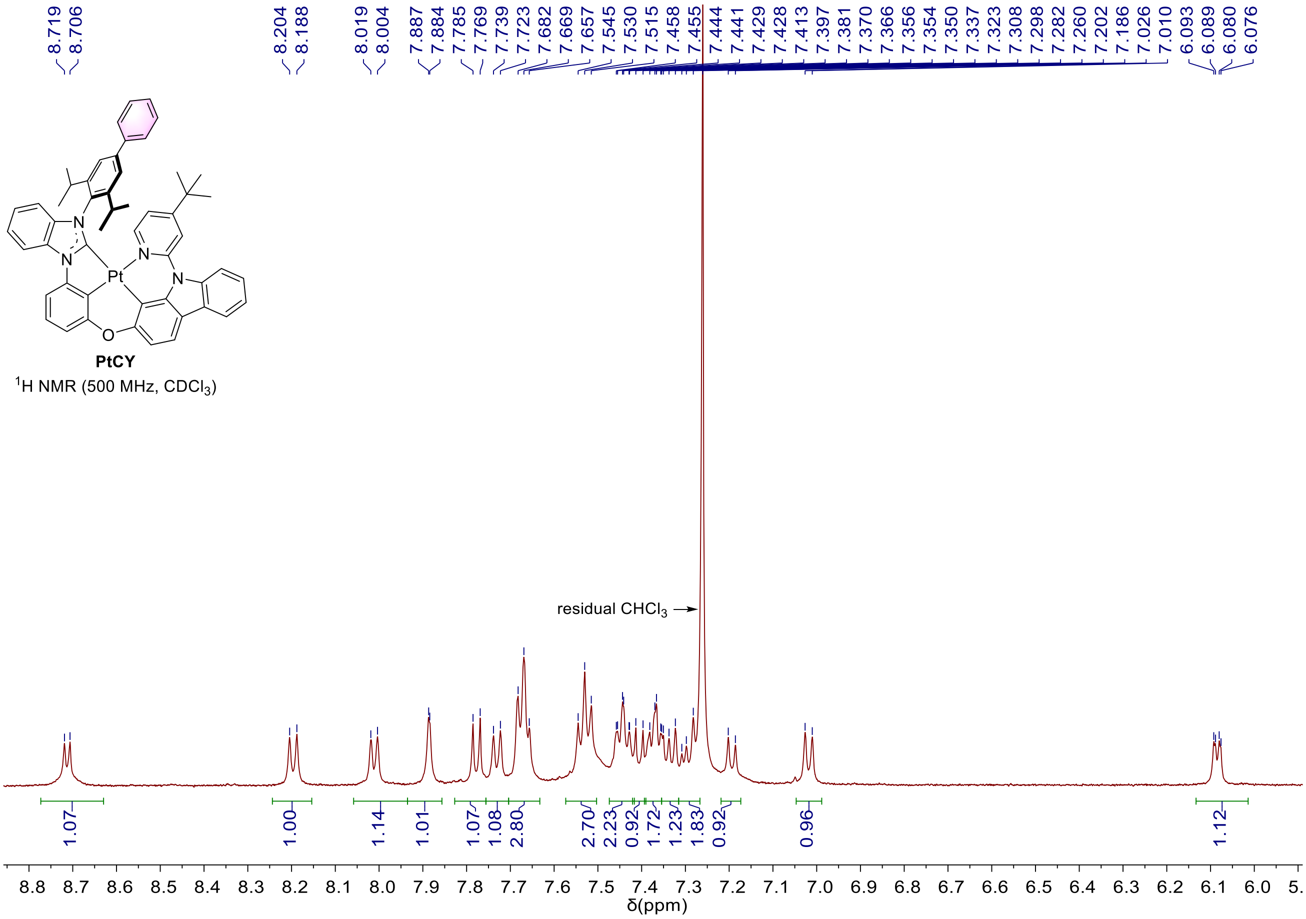
PtCY
^{1}H NMR (500 MHz, $CDCl_3$)
residual $CHCl_3$
δ(ppm)

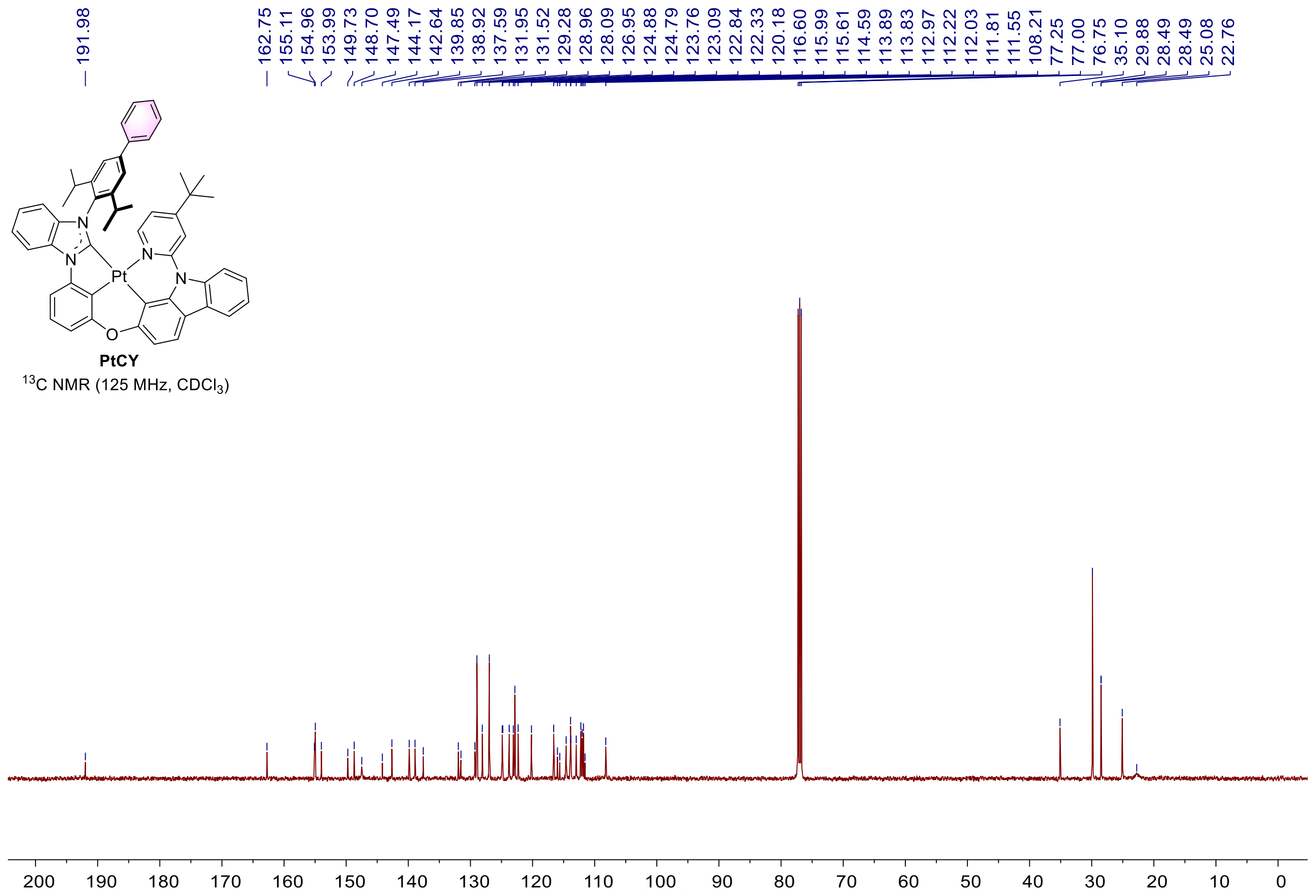
191.98
162.75
155.11
154.96
153.99
149.73
148.70
147.49
144.17
142.64
139.85
138.92
137.59
131.95
131.52
129.28
128.96
128.09
126.95
124.88
124.79
123.76
123.09
122.84
122.33
120.18
116.60
115.99
115.61
114.59
113.89
113.83
112.97
112.22
112.03
111.81
111.55
108.21
77.25
77.00
76.75
35.10
29.88
28.49
28.49
25.08
22.76
PtCY
13C NMR (125 MHz, CDCl3)
200 190 180 170 160 150 140 130 120 110 100 90 80 70 60 50 40 30 20 10 0
δ(ppm)

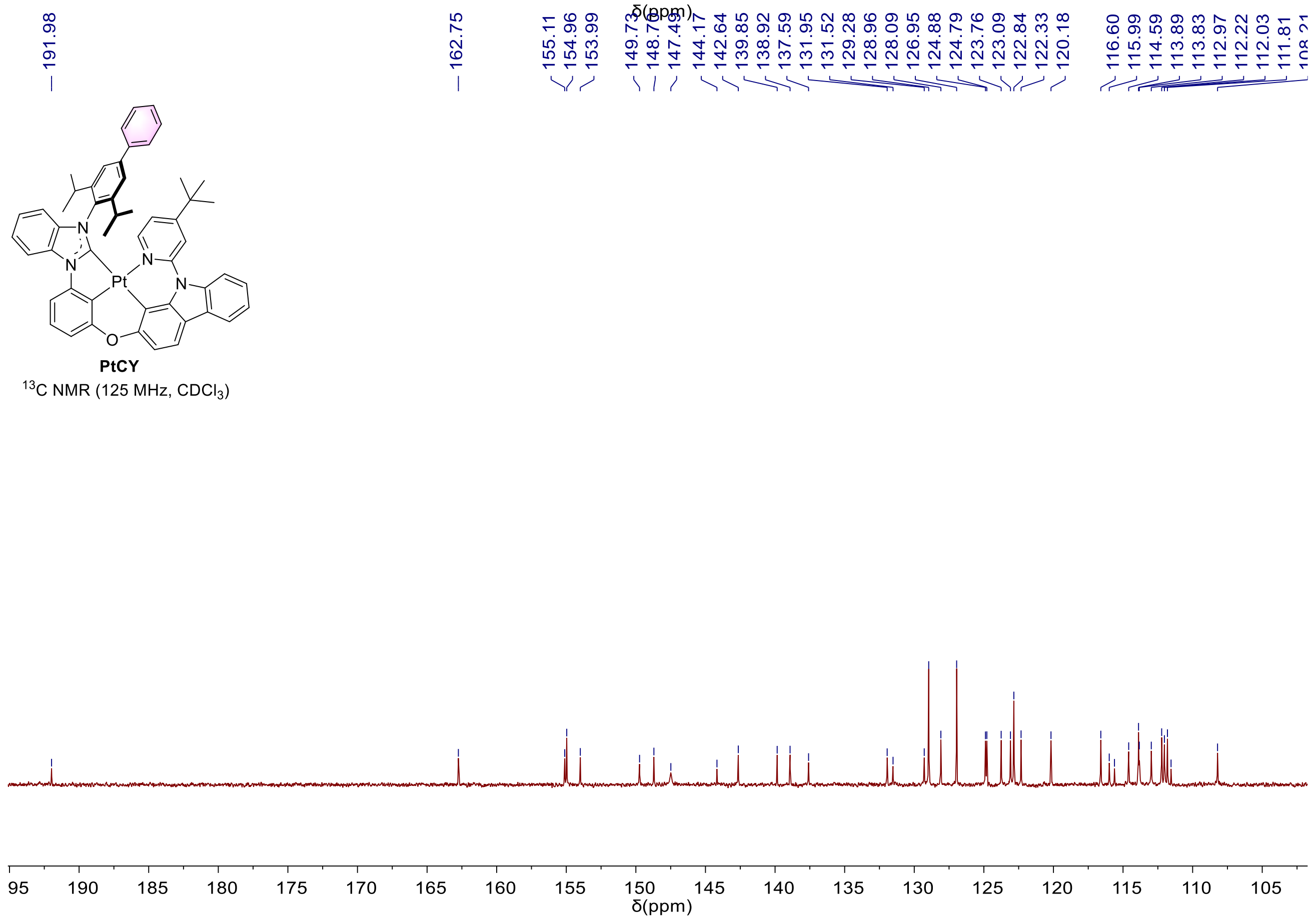
191.98
162.75
155.11
154.96
153.99
149.73
148.70
147.49
144.17
142.64
139.85
138.92
137.59
131.95
131.52
129.28
128.96
128.09
126.95
124.88
124.79
123.76
123.09
122.84
122.33
120.18
116.60
115.99
114.59
113.89
113.83
112.97
112.22
112.03
111.81
PtCY
13C NMR (125 MHz, CDCl3)
95 190 185 180 175 170 165 160 155 150 145 140 135 130 125 120 115 110 105
δ(ppm)

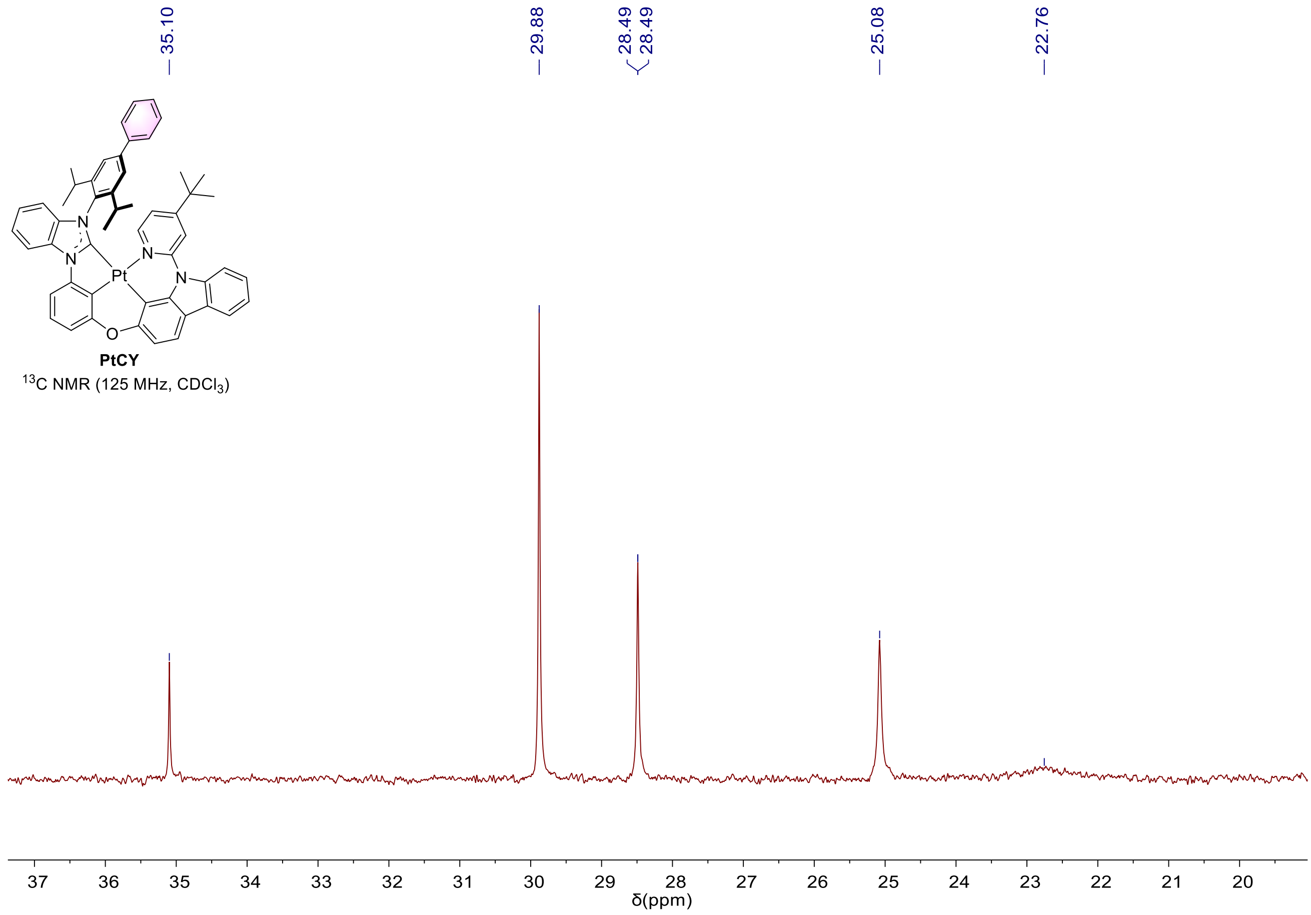

35.10
29.88
28.49
28.49
25.08
22.76
PtCY
^{13}C NMR (125 MHz, CDCl$_3$)
δ(ppm)

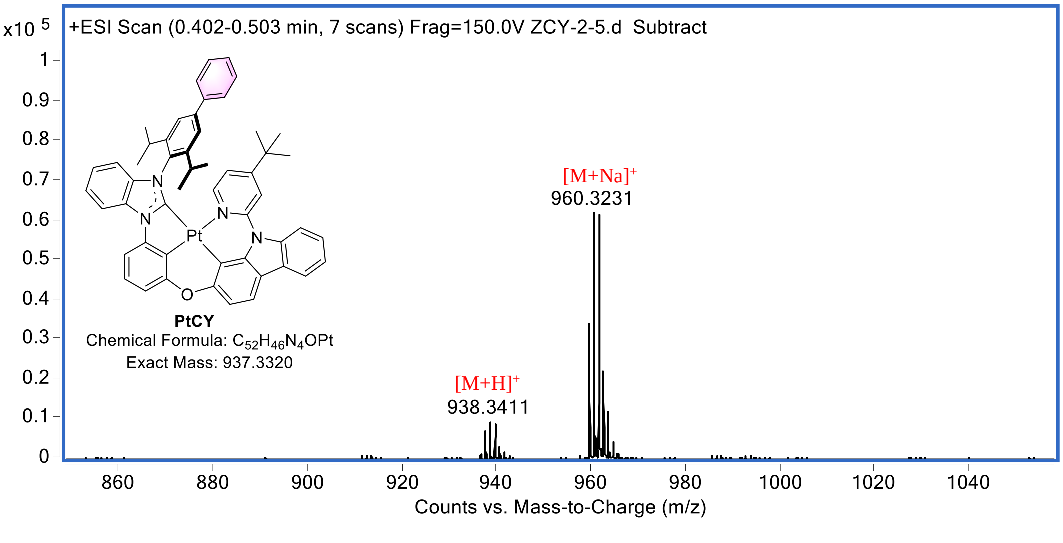

+ESI Scan (0.402-0.503 min, 7 scans) Frag=150.0V ZCY-2-5.d Subtract
x10 5
PtCY
Chemical Formula: $C_{52}H_{46}N_4OPt$
Exact Mass: 937.3320
[M+Na]+
960.3231
[M+H]+
938.3411
Counts vs. Mass-to-Charge (m/z)

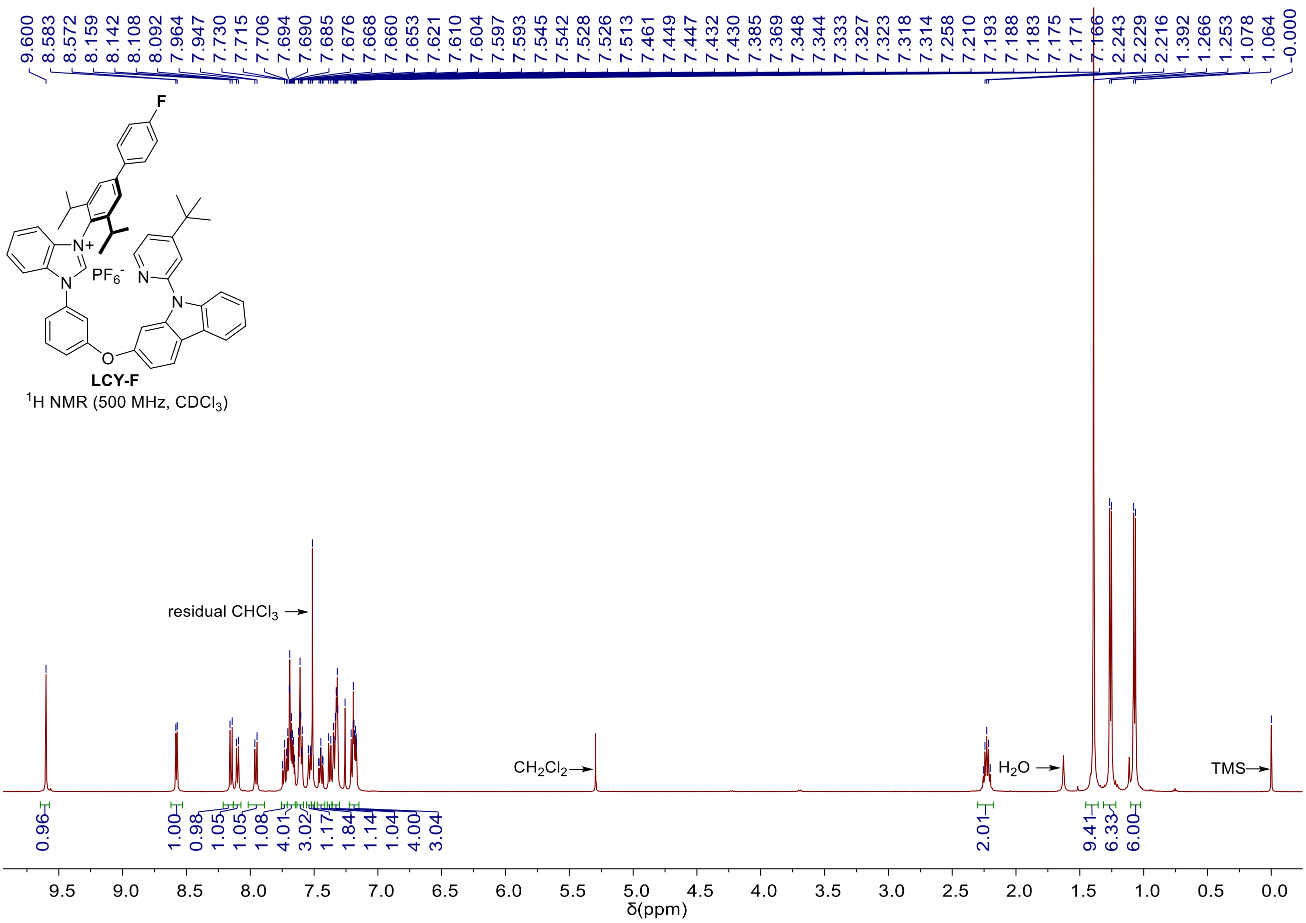
9.600 8.583 8.572 8.159 8.142 8.108 8.092 7.964 7.947 7.730 7.715 7.706 7.694 7.690 7.685 7.676 7.668 7.660 7.653 7.621 7.610 7.604 7.597 7.593 7.545 7.542 7.528 7.526 7.513 7.461 7.449 7.447 7.432 7.430 7.385 7.369 7.348 7.344 7.333 7.327 7.323 7.318 7.314 7.258 7.210 7.193 7.188 7.183 7.175 7.171 7.166 2.243 2.229 2.216 1.392 1.266 1.253 1.078 1.064 -0.000
F
N+
PF6-
N
N
N
O
LCY-F
1H NMR (500 MHz, CDCl3)
residual CHCl3
CH2Cl2
H2O
TMS
0.96 1.00 0.98 1.05 1.05 1.08 4.01 3.02 1.17 1.84 1.14 1.04 4.00 3.04 2.01 9.41 6.33 6.00
9.5 9.0 8.5 8.0 7.5 7.0 6.5 6.0 5.5 5.0 4.5 4.0 3.5 3.0 2.5 2.0 1.5 1.0 0.5 0.0
δ(ppm)

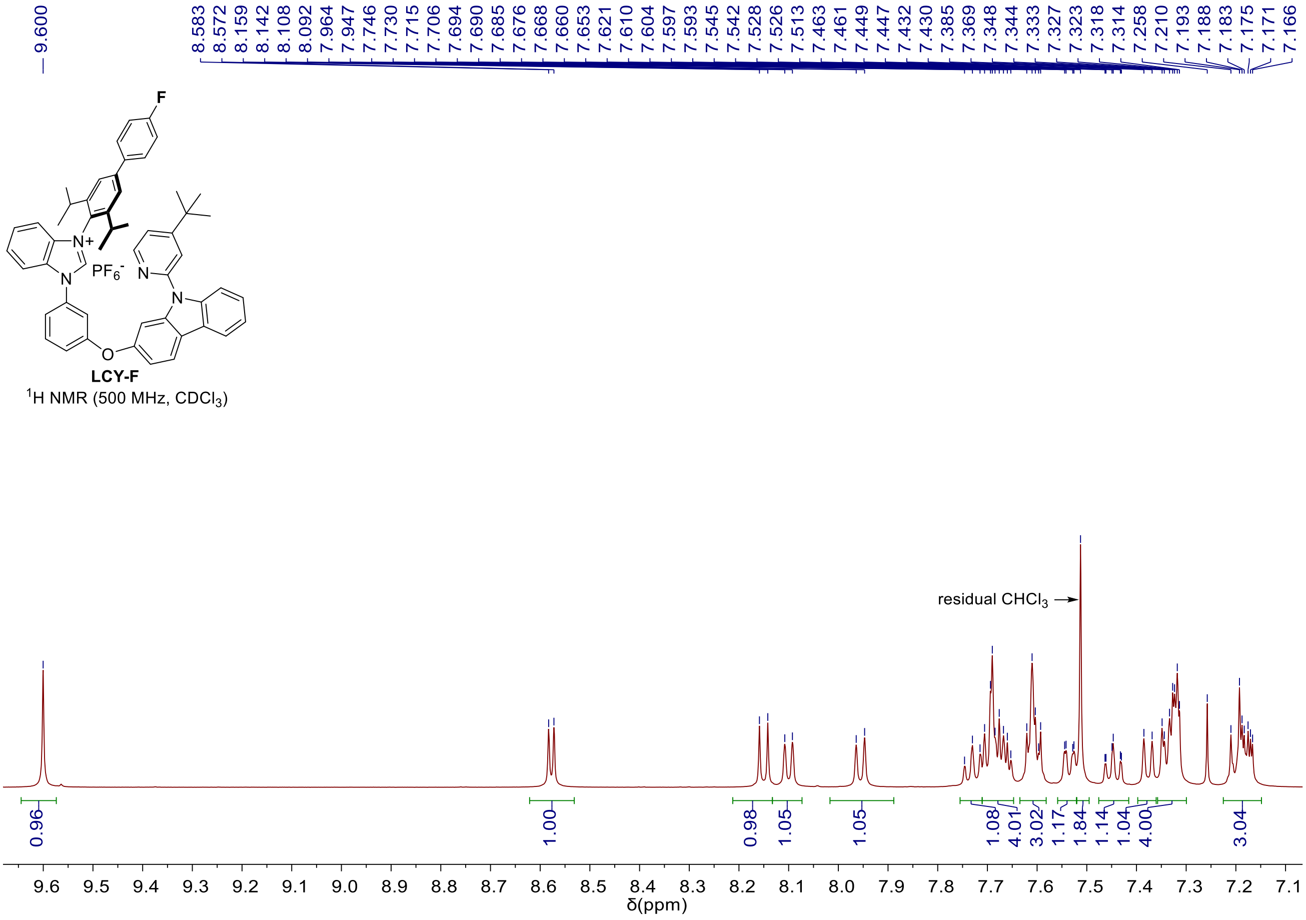
9.600 8.583 8.572 8.159 8.142 8.108 8.092 7.964 7.947 7.746 7.730 7.715 7.706 7.694 7.690 7.685 7.676 7.668 7.660 7.653 7.621 7.610 7.604 7.597 7.593 7.545 7.542 7.528 7.526 7.513 7.463 7.461 7.449 7.447 7.432 7.430 7.385 7.369 7.348 7.344 7.333 7.327 7.323 7.318 7.314 7.258 7.210 7.193 7.188 7.183 7.175 7.171 7.166
F
N+
PF6-
N
N
N
O
LCY-F
1H NMR (500 MHz, CDCl3)
residual CHCl3
0.96 1.00 0.98 1.05 1.05 1.08 4.01 3.02 1.17 1.84 1.14 1.04 4.00 3.04
9.6 9.5 9.4 9.3 9.2 9.1 9.0 8.9 8.8 8.7 8.6 8.5 8.4 8.3 8.2 8.1 8.0 7.9 7.8 7.7 7.6 7.5 7.4 7.3 7.2 7.1
δ(ppm)

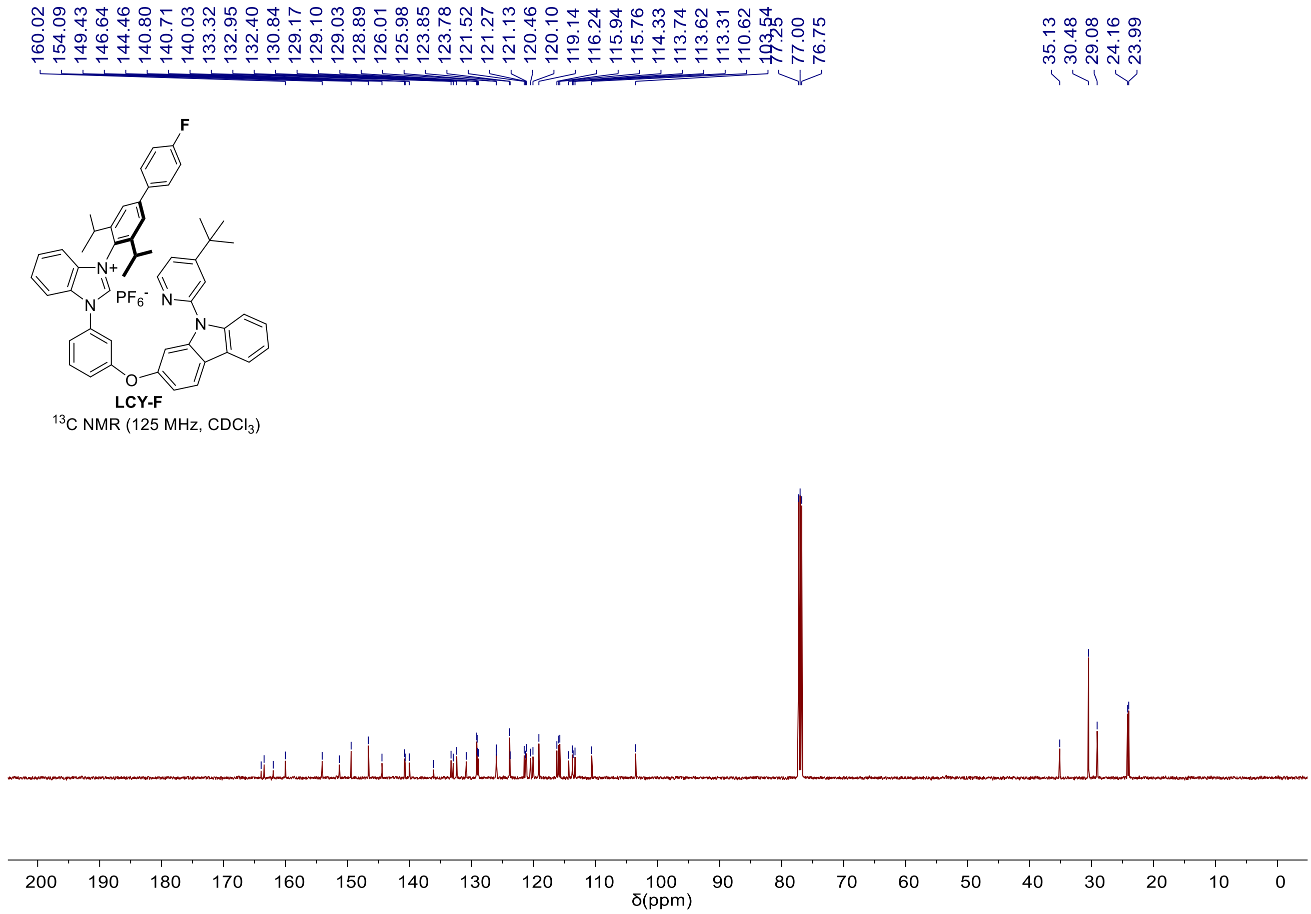
160.02 154.09 149.43 146.64 144.46 140.80 140.71 140.03 133.32 132.95 132.40 130.84 129.17 129.10 129.03 128.89 126.01 125.98 123.85 123.78 121.52 121.27 121.13 120.46 120.10 119.14 116.24 115.94 115.76 114.33 113.74 113.62 113.31 110.62 103.54 77.25 77.00 76.75
35.13 30.48 29.08 24.16 23.99
F
N+
N
PF6-
N
N
O
LCY-F
13C NMR (125 MHz, CDCl3)
200 190 180 170 160 150 140 130 120 110 100 90 80 70 60 50 40 30 20 10 0
δ(ppm)

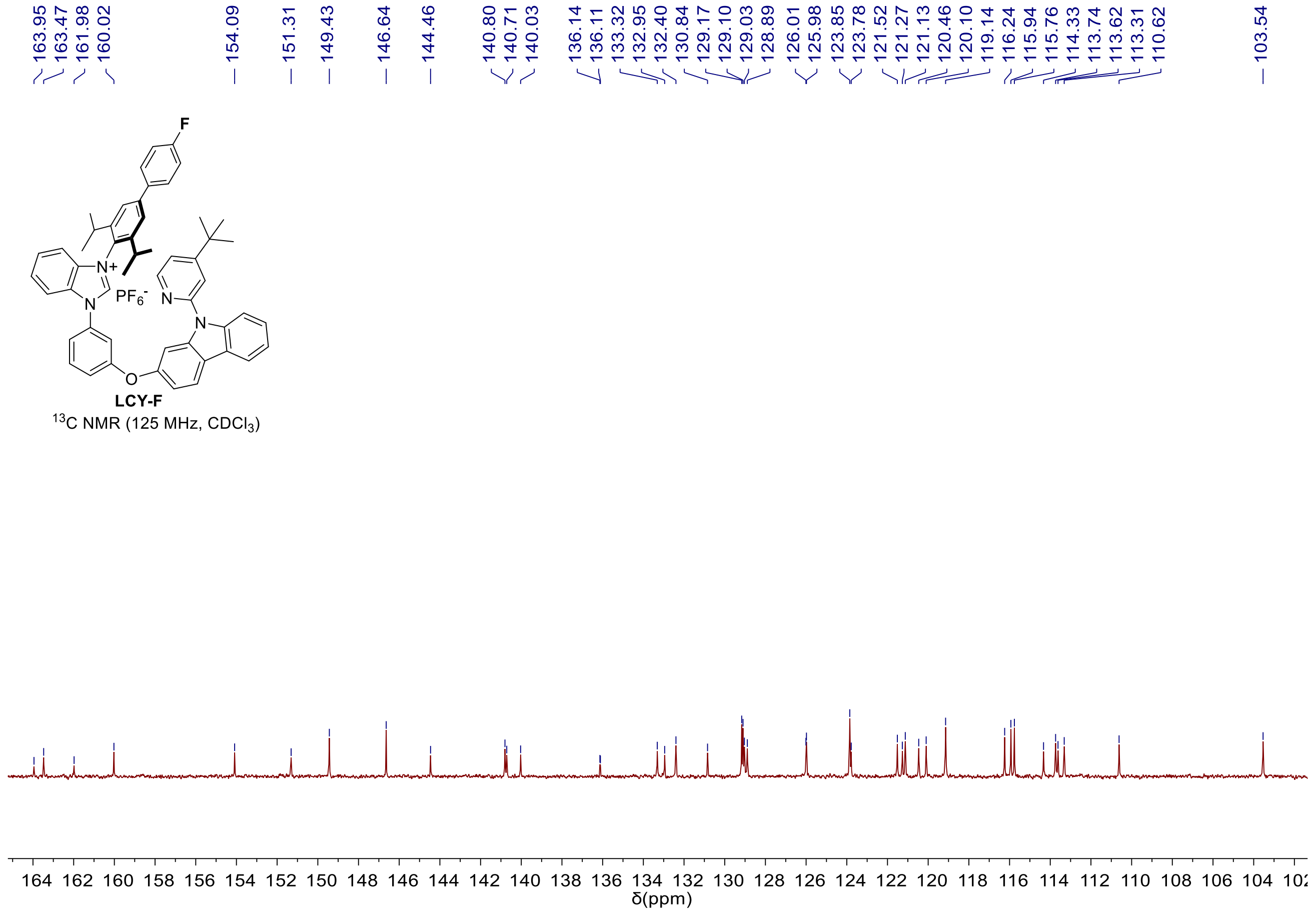
163.95 163.47 161.98 160.02 154.09 151.31 149.43 146.64 144.46 140.80 140.71 140.03 136.14 136.11 133.32 132.95 132.40 130.84 129.17 129.10 129.03 128.89 126.01 125.98 123.85 123.78 121.52 121.27 121.13 120.46 120.10 119.14 116.24 115.94 115.76 114.33 113.74 113.62 113.31 110.62 103.54
F
N+
N
PF6-
N
N
O
LCY-F
13C NMR (125 MHz, CDCl3)
164 162 160 158 156 154 152 150 148 146 144 142 140 138 136 134 132 130 128 126 124 122 120 118 116 114 112 110 108 106 104
δ(ppm)

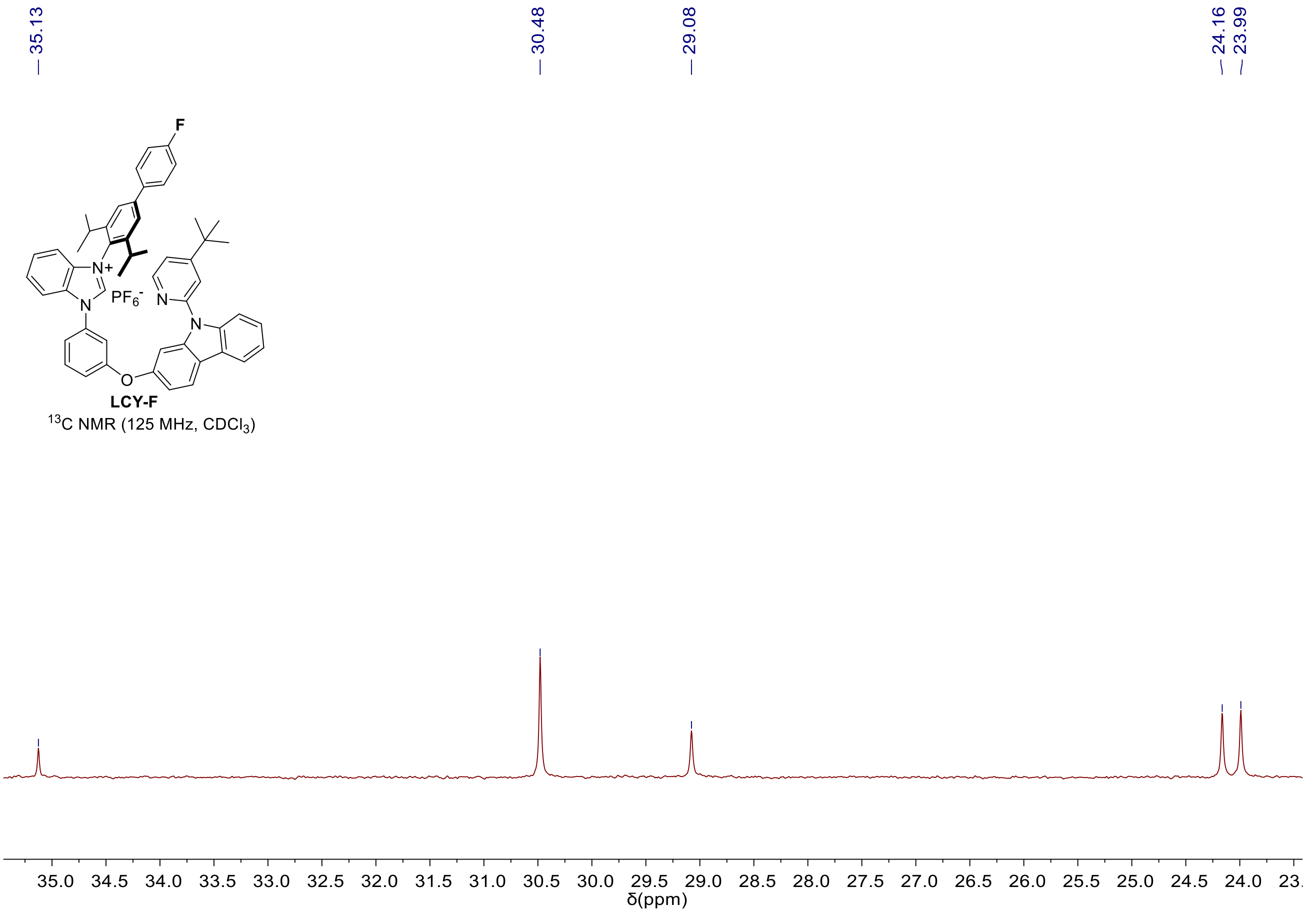
35.13
30.48
29.08
24.16
23.99
F
N+
PF6-
N
N
N
O
LCY-F
13C NMR (125 MHz, CDCl3)
35.0 34.5 34.0 33.5 33.0 32.5 32.0 31.5 31.0 30.5 30.0 29.5 29.0 28.5 28.0 27.5 27.0 26.5 26.0 25.5 25.0 24.5 24.0
δ(ppm)

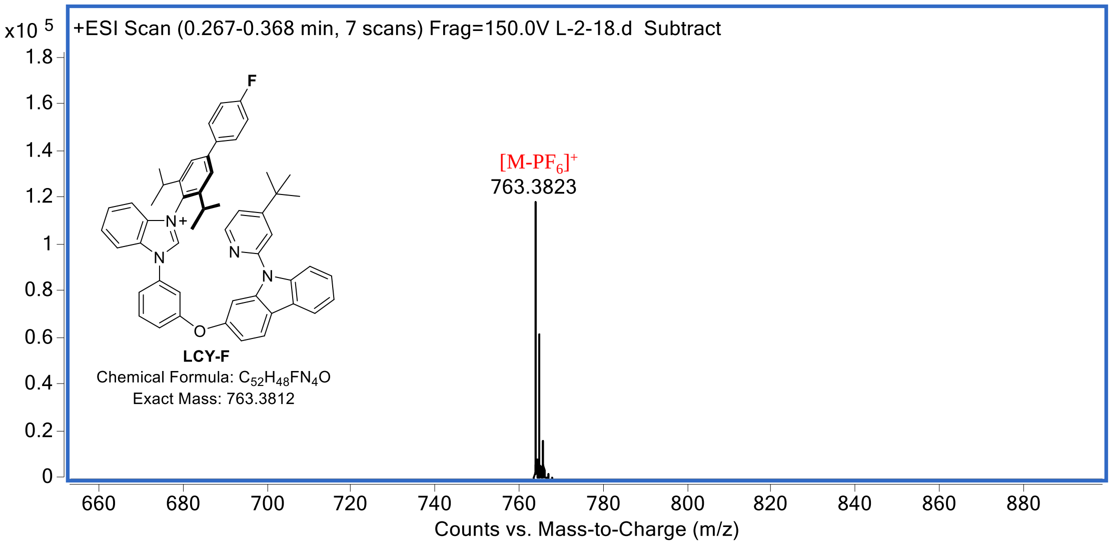
x10 5
+ESI Scan (0.267-0.368 min, 7 scans) Frag=150.0V L-2-18.d Subtract
1.8
1.6
1.4
1.2
1
0.8
0.6
0.4
0.2
0
[M-PF6]+
763.3823
F
N+
N
N
N
O
LCY-F
Chemical Formula: C52H48FN4O
Exact Mass: 763.3812
660 680 700 720 740 760 780 800 820 840 860 880
Counts vs. Mass-to-Charge (m/z)

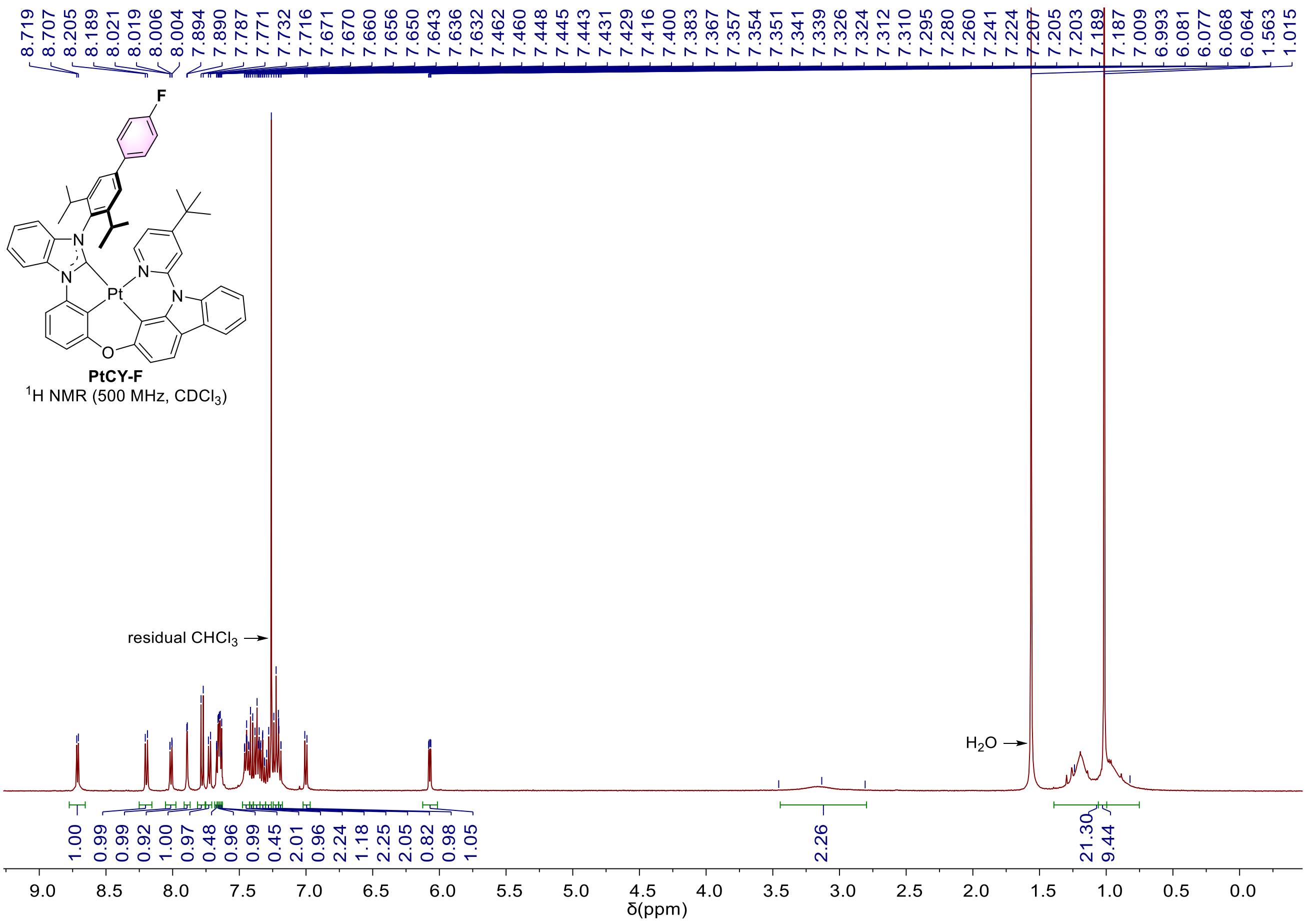
8.719 8.707 8.205 8.189 8.021 8.019 8.006 8.004 7.894 7.890 7.787 7.771 7.732 7.716 7.671 7.670 7.660 7.656 7.650 7.643 7.636 7.632 7.462 7.460 7.448 7.445 7.443 7.431 7.429 7.416 7.400 7.383 7.367 7.357 7.354 7.351 7.341 7.339 7.326 7.324 7.312 7.310 7.295 7.280 7.260 7.241 7.224 7.207 7.205 7.203 7.189 7.187 7.009 6.993 6.081 6.077 6.068 6.064 1.563 1.015
F
N
N
Pt
N
N
O
PtCY-F
^{1}H NMR (500 MHz, $CDCl_3$)
residual $CHCl_3$
H_2O
1.00 0.99 0.99 0.92 1.00 0.97 0.48 0.96 0.99 0.45 2.01 0.96 2.24 1.18 2.25 2.05 0.82 0.98 1.05
2.26
21.30 9.44
9.0 8.5 8.0 7.5 7.0 6.5 6.0 5.5 5.0 4.5 4.0 3.5 3.0 2.5 2.0 1.5 1.0 0.5 0.0
δ(ppm)

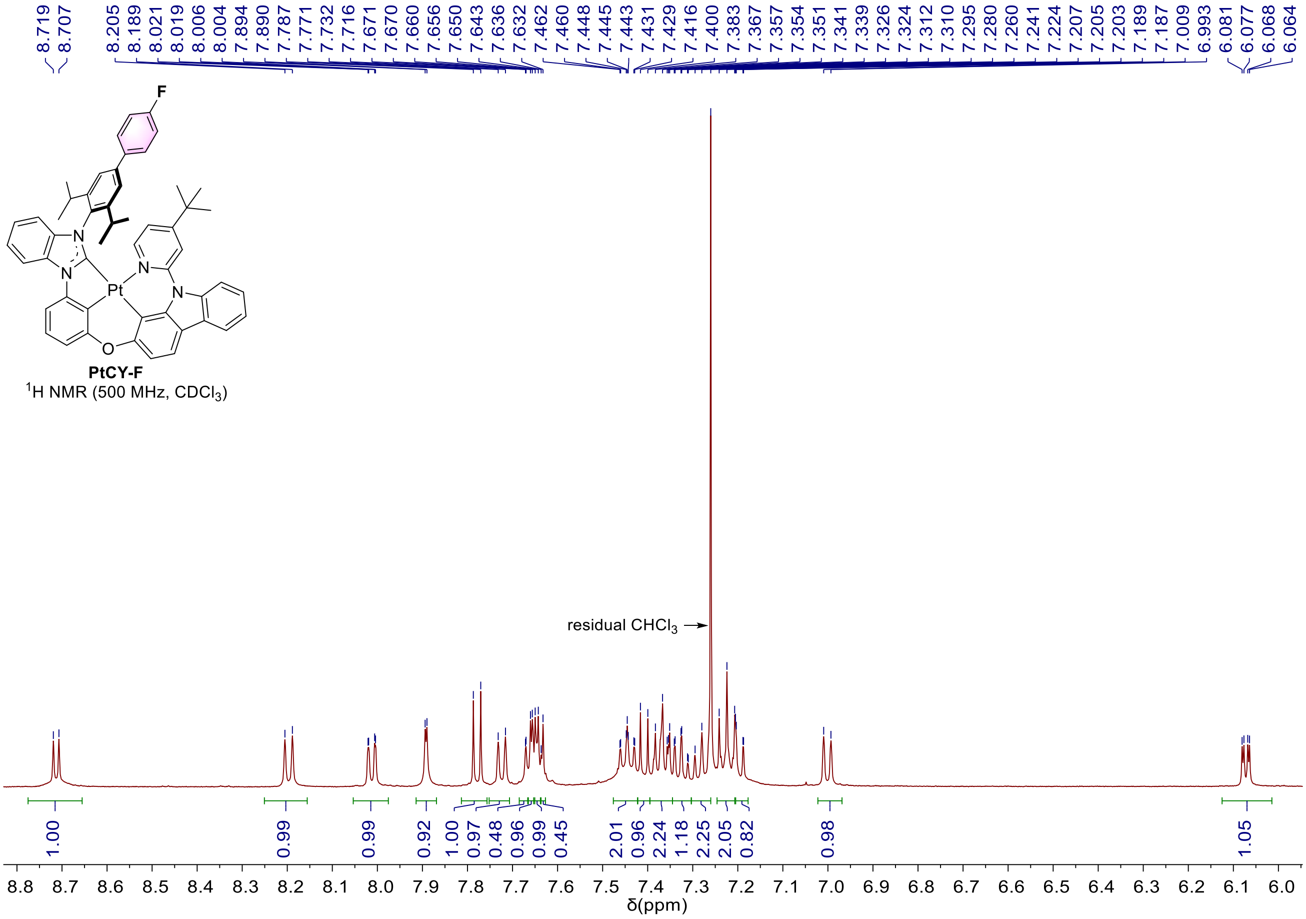
8.719 8.707 8.205 8.189 8.021 8.019 8.006 8.004 7.894 7.890 7.787 7.771 7.732 7.716 7.671 7.670 7.660 7.656 7.650 7.643 7.636 7.632 7.462 7.460 7.448 7.445 7.443 7.431 7.429 7.416 7.400 7.383 7.367 7.357 7.354 7.351 7.341 7.339 7.326 7.324 7.312 7.310 7.295 7.280 7.260 7.241 7.224 7.207 7.205 7.203 7.189 7.187 7.009 6.993 6.081 6.077 6.068 6.064
F
N
N
Pt
N
N
O
PtCY-F
^{1}H NMR (500 MHz, $CDCl_3$)
residual $CHCl_3$
1.00 0.99 0.99 0.92 1.00 0.97 0.48 0.96 0.99 0.45 2.01 0.96 2.24 1.18 2.25 2.05 0.82 0.98 1.05
8.8 8.7 8.6 8.5 8.4 8.3 8.2 8.1 8.0 7.9 7.8 7.7 7.6 7.5 7.4 7.3 7.2 7.1 7.0 6.9 6.8 6.7 6.6 6.5 6.4 6.3 6.2 6.1 6.0
δ(ppm)

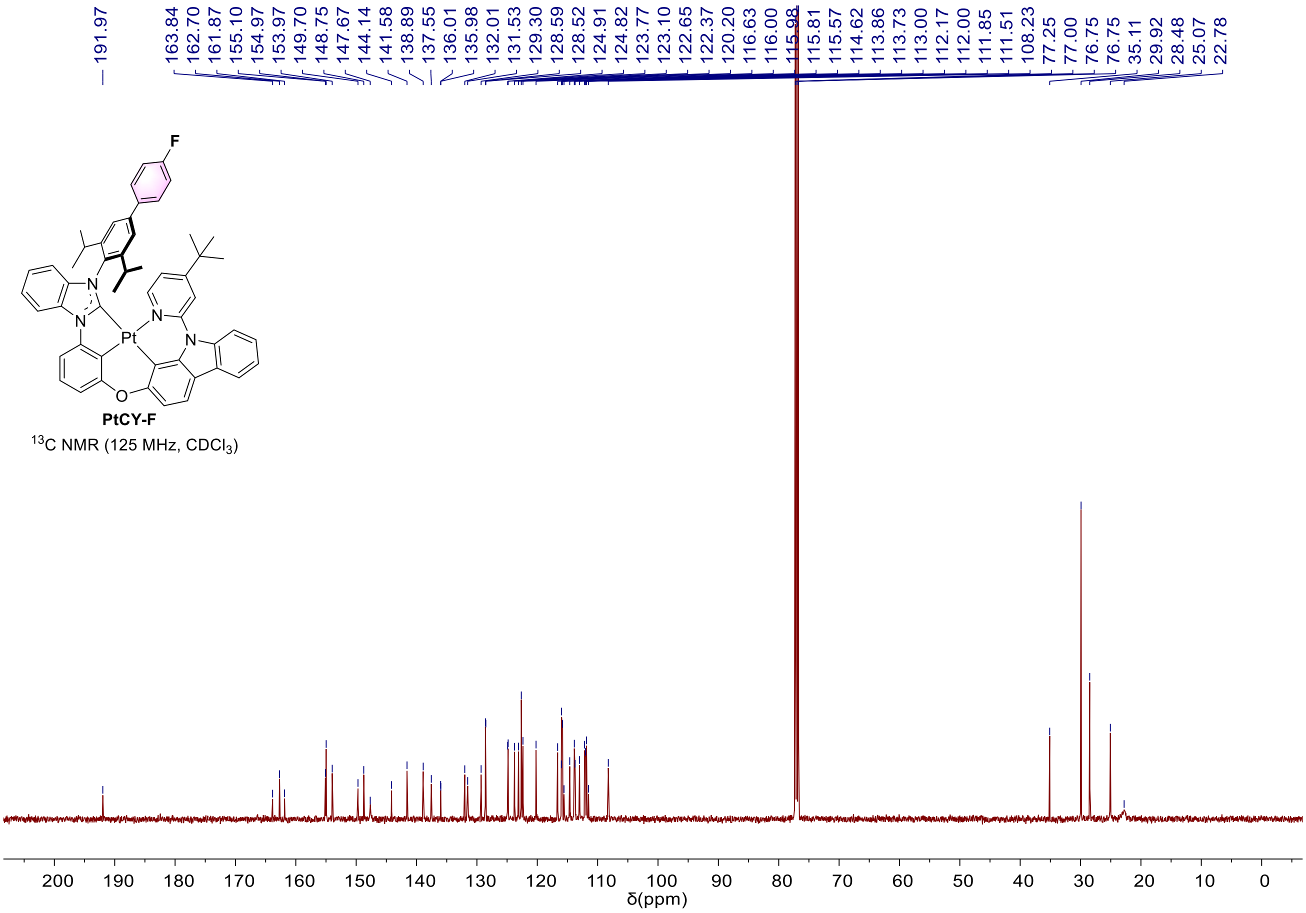
191.97
163.84 162.70 161.87 155.10 154.97 153.97 149.70 148.75 147.67 144.14 141.58 138.89 137.55 136.01 135.98 132.01 131.53 129.30 128.59 128.52 124.91 124.82 123.77 123.10 122.65 122.37 120.20 116.63 116.00 115.98 115.81 115.57 114.62 113.86 113.73 113.00 112.17 112.00 111.85 111.51 108.23 77.25 77.00 76.75 35.11 29.92 28.48 25.07 22.78
F
N
Pt
O
PtCY-F
^{13}C NMR (125 MHz, $CDCl_3$)
δ(ppm)

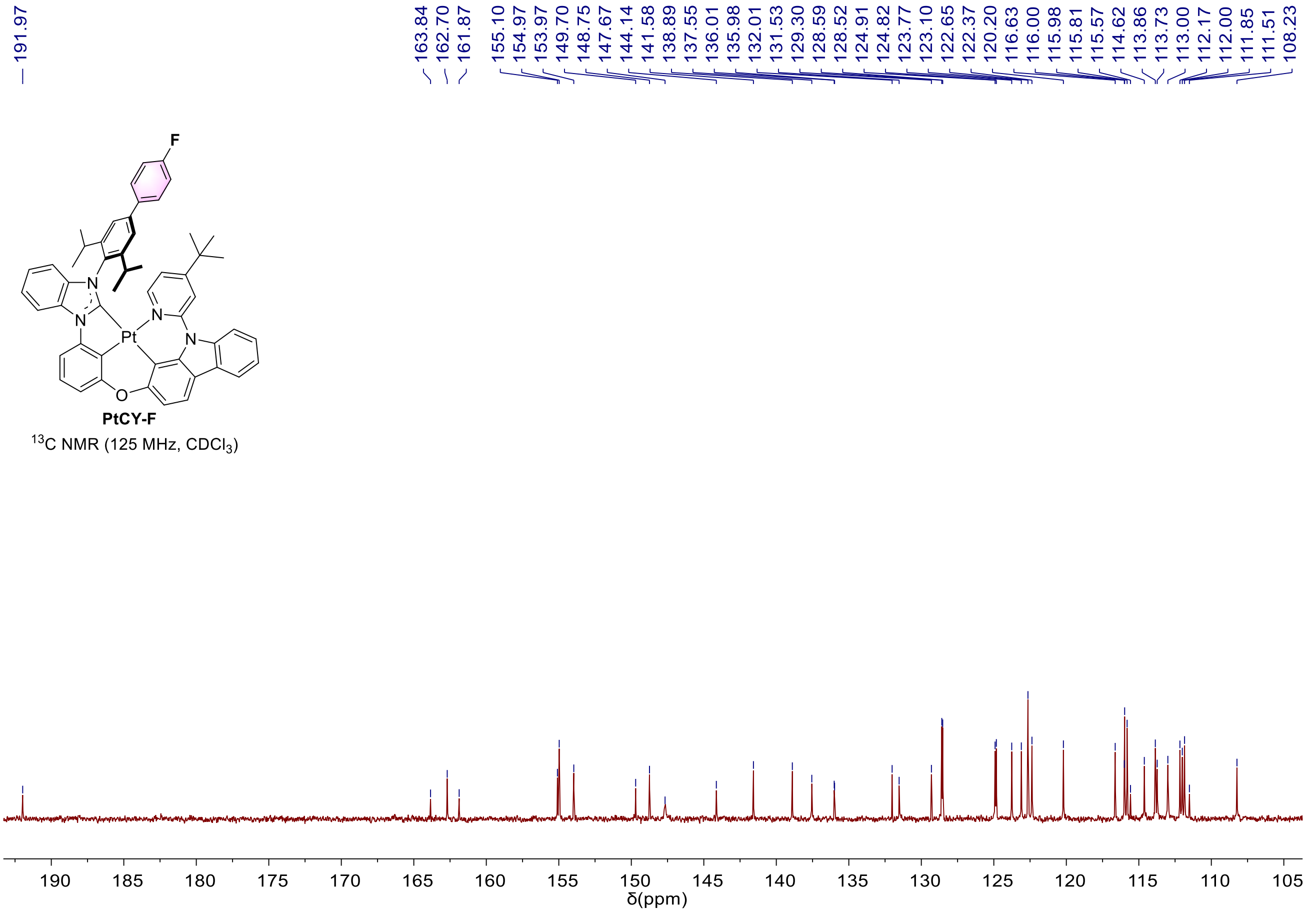
191.97
163.84 162.70 161.87 155.10 154.97 153.97 149.70 148.75 147.67 144.14 141.58 138.89 137.55 136.01 135.98 132.01 131.53 129.30 128.59 128.52 124.91 124.82 123.77 123.10 122.65 122.37 120.20 116.63 116.00 115.98 115.81 115.57 114.62 113.86 113.73 113.00 112.17 112.00 111.85 111.51 108.23
F
N
Pt
O
PtCY-F
^{13}C NMR (125 MHz, $CDCl_3$)
δ(ppm)

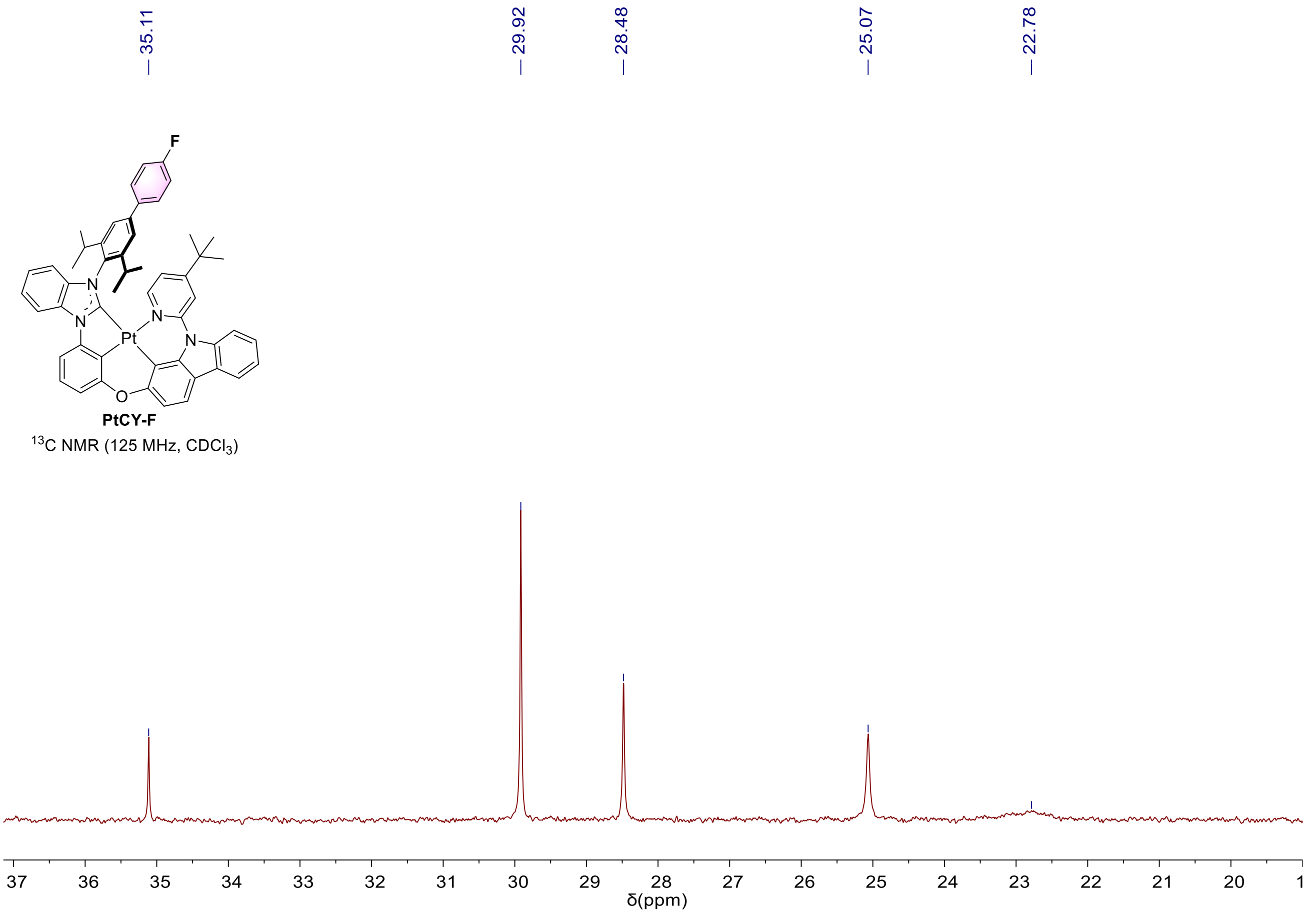
35.11
29.92
28.48
25.07
22.78
F
N
N
Pt
N
N
O
PtCY-F
13C NMR (125 MHz, CDCl3)
37
36
35
34
33
32
31
30
29
28
27
26
25
24
23
22
21
20
1
δ(ppm)

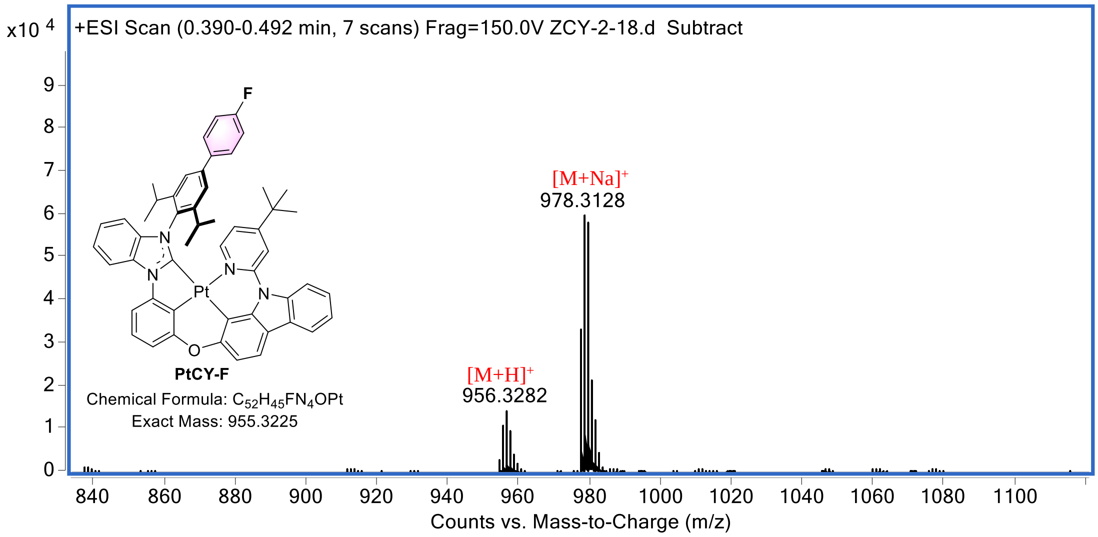
x10 4
+ESI Scan (0.390-0.492 min, 7 scans) Frag=150.0V ZCY-2-18.d Subtract
9
8
7
6
5
4
3
2
1
0
F
N
N
Pt
N
N
O
PtCY-F
Chemical Formula: C52H45FN4OPt
Exact Mass: 955.3225
[M+Na]+
978.3128
[M+H]+
956.3282
840
860
880
900
920
940
960
980
1000
1020
1040
1060
1080
1100
Counts vs. Mass-to-Charge (m/z)

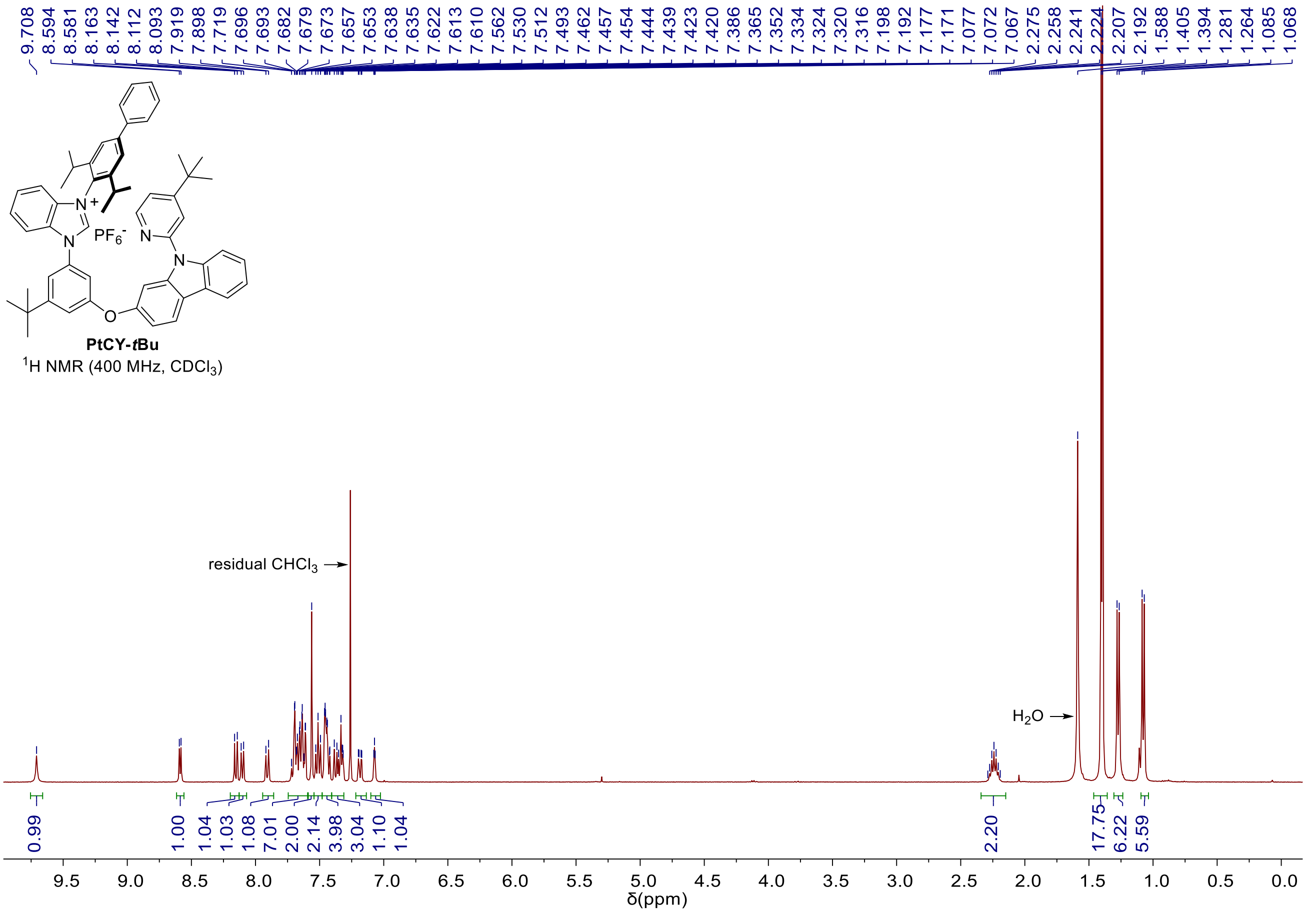
9.708 8.594 8.581 8.163 8.142 8.112 8.093 7.919 7.898 7.719 7.696 7.693 7.682 7.679 7.673 7.657 7.653 7.638 7.635 7.622 7.613 7.610 7.562 7.530 7.512 7.493 7.462 7.457 7.454 7.444 7.439 7.423 7.420 7.386 7.365 7.352 7.334 7.324 7.320 7.316 7.198 7.192 7.177 7.171 7.077 7.072 7.067 2.275 2.258 2.241 2.224 2.207 2.192 1.588 1.405 1.394 1.281 1.264 1.085 1.068
PtCY-tBu
^{1}H NMR (400 MHz, CDCl$_3$)
N
N$^+$
PF$_6^-$
N
N
O
residual CHCl$_3$
H$_2$O
0.99
1.00
1.04
1.03
1.08
7.01
2.00
2.14
3.98
3.04
1.10
1.04
2.20
17.75
6.22
5.59
9.5 9.0 8.5 8.0 7.5 7.0 6.5 6.0 5.5 5.0 4.5 4.0 3.5 3.0 2.5 2.0 1.5 1.0 0.5 0.0
δ(ppm)

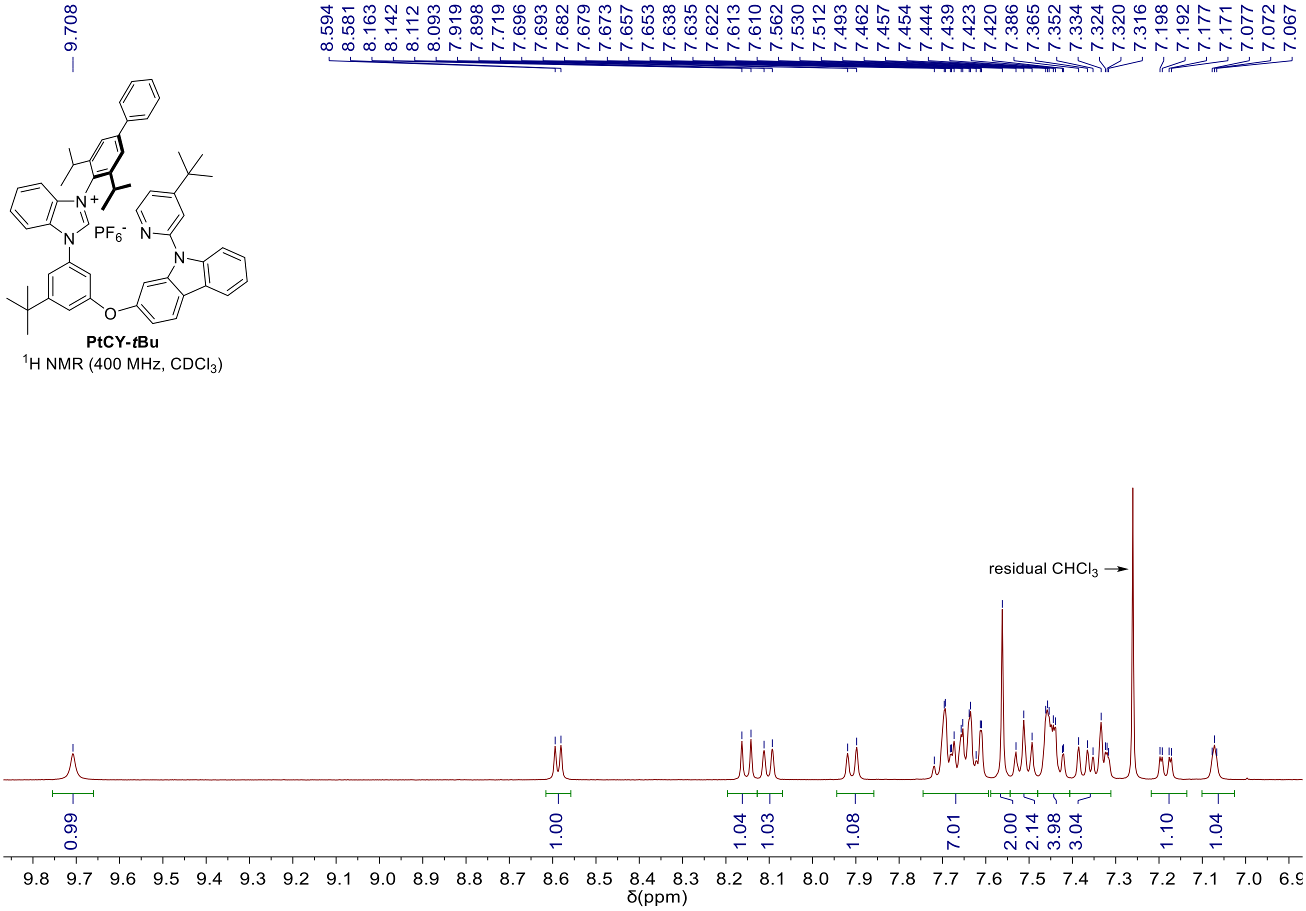
9.708
8.594 8.581 8.163 8.142 8.112 8.093 7.919 7.898 7.719 7.696 7.693 7.682 7.679 7.673 7.657 7.653 7.638 7.635 7.622 7.613 7.610 7.562 7.530 7.512 7.493 7.462 7.457 7.454 7.444 7.439 7.423 7.420 7.386 7.365 7.352 7.334 7.324 7.320 7.316 7.198 7.192 7.177 7.171 7.077 7.072 7.067
PtCY-tBu
^{1}H NMR (400 MHz, CDCl$_3$)
N
N$^+$
PF$_6^-$
N
N
O
residual CHCl$_3$
0.99
1.00
1.04
1.03
1.08
7.01
2.00
2.14
3.98
3.04
1.10
1.04
9.8 9.7 9.6 9.5 9.4 9.3 9.2 9.1 9.0 8.9 8.8 8.7 8.6 8.5 8.4 8.3 8.2 8.1 8.0 7.9 7.8 7.7 7.6 7.5 7.4 7.3 7.2 7.1 7.0 6.9
δ(ppm)

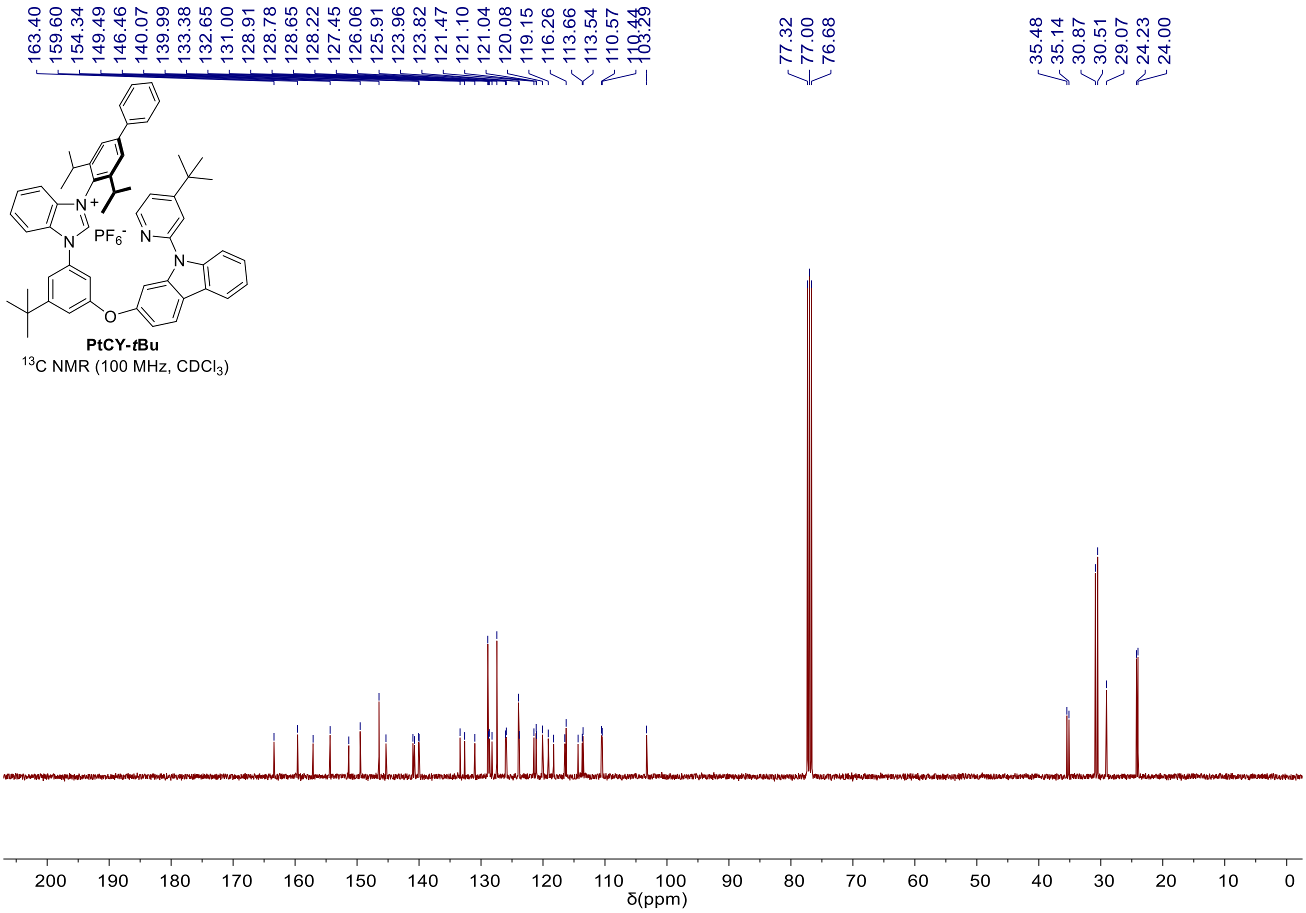
163.40
159.60
154.34
149.49
146.46
140.07
139.99
133.38
132.65
131.00
128.91
128.78
128.65
128.22
127.45
126.06
125.91
123.96
123.82
121.47
121.10
121.04
120.08
119.15
116.26
113.66
113.54
110.57
110.44
103.29
77.32
77.00
76.68
35.48
35.14
30.87
30.51
29.07
24.23
24.00
N
PF6-
N
N
O
PtCY-tBu
13C NMR (100 MHz, CDCl3)
δ(ppm)

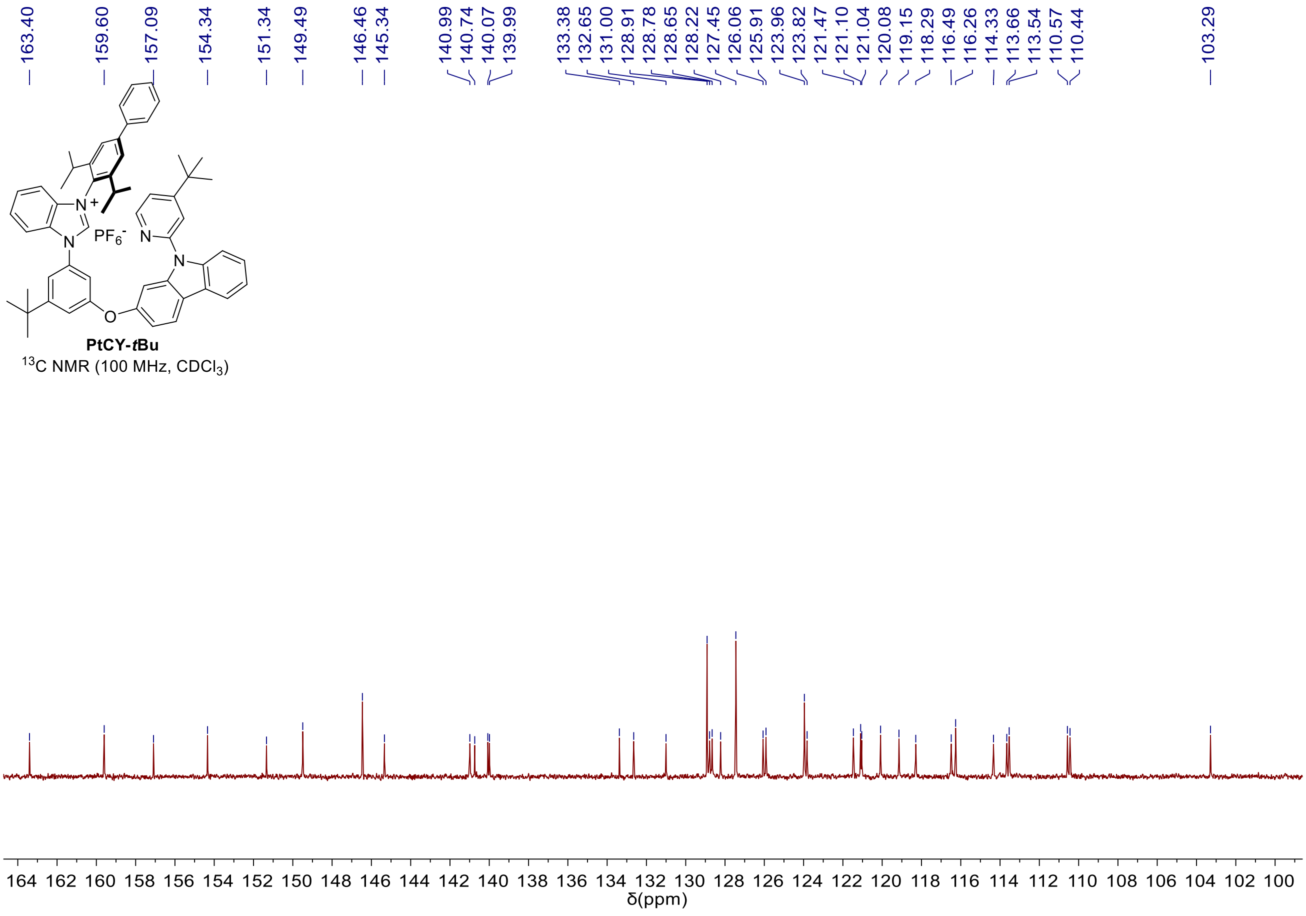
163.40
159.60
157.09
154.34
151.34
149.49
146.46
145.34
140.99
140.74
140.07
139.99
133.38
132.65
131.00
128.91
128.78
128.65
128.22
127.45
126.06
125.91
123.96
123.82
121.47
121.10
121.04
120.08
119.15
118.29
116.49
116.26
114.33
113.66
113.54
110.57
110.44
103.29
N
PF6-
N
N
O
PtCY-tBu
13C NMR (100 MHz, CDCl3)
δ(ppm)

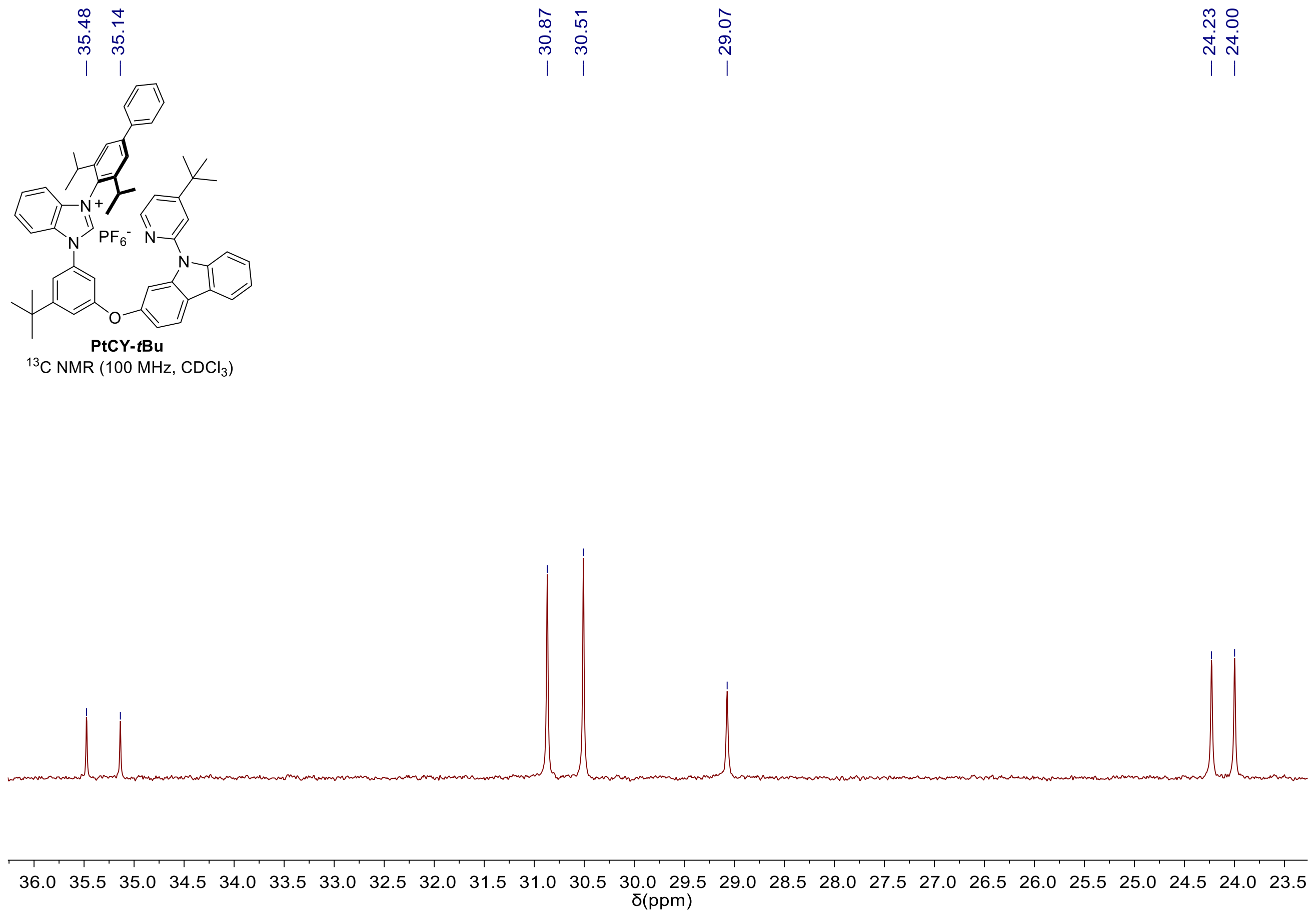
35.48
35.14
30.87
30.51
29.07
24.23
24.00
PtCY-tBu
^{13}C NMR (100 MHz, $CDCl_3$)
36.0 35.5 35.0 34.5 34.0 33.5 33.0 32.5 32.0 31.5 31.0 30.5 30.0 29.5 29.0 28.5 28.0 27.5 27.0 26.5 26.0 25.5 25.0 24.5 24.0 23.5
δ(ppm)

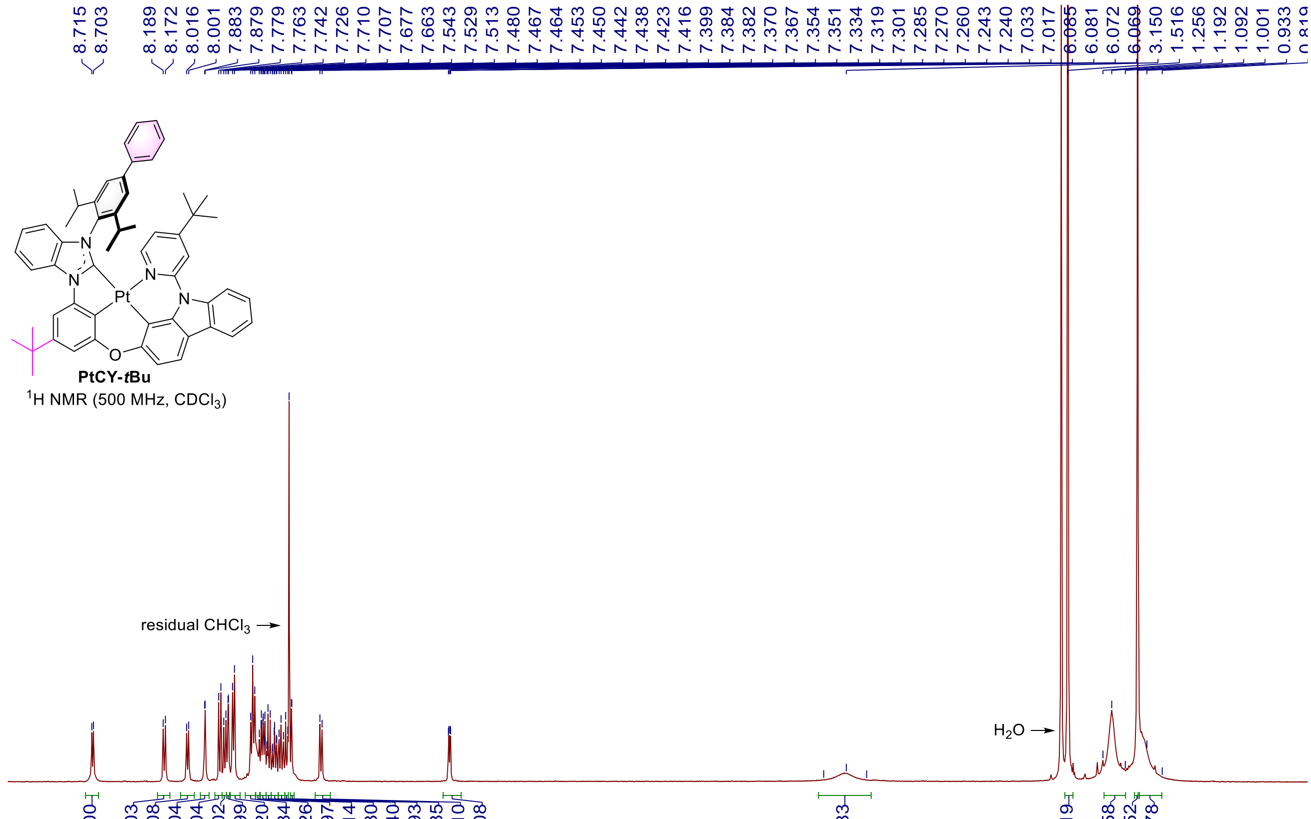
8.715
8.703
8.189
8.172
8.016
8.001
7.883
7.879
7.779
7.763
7.742
7.726
7.710
7.707
7.677
7.663
7.543
7.529
7.513
7.480
7.467
7.464
7.453
7.450
7.442
7.438
7.423
7.416
7.399
7.384
7.382
7.370
7.367
7.354
7.351
7.334
7.319
7.301
7.285
7.270
7.260
7.243
7.240
7.033
7.017
6.085
6.081
6.072
6.069
3.150
1.516
1.256
1.192
1.092
1.001
0.933
PtCY-tBu
^{1}H NMR (500 MHz, $CDCl_3$)
residual $CHCl_3$
H_2O
1.00
1.03
1.08
1.04
1.04
1.02
0.99
2.20
2.84
1.26
1.97
1.14
1.30
1.40
0.93
0.85
1.10
1.08
1.83
9.19
5.58
9.52
5.78
9.0 8.5 8.0 7.5 7.0 6.5 6.0 5.5 5.0 4.5 4.0 3.5 3.0 2.5 2.0 1.5 1.0 0.5 0.0
δ(ppm)

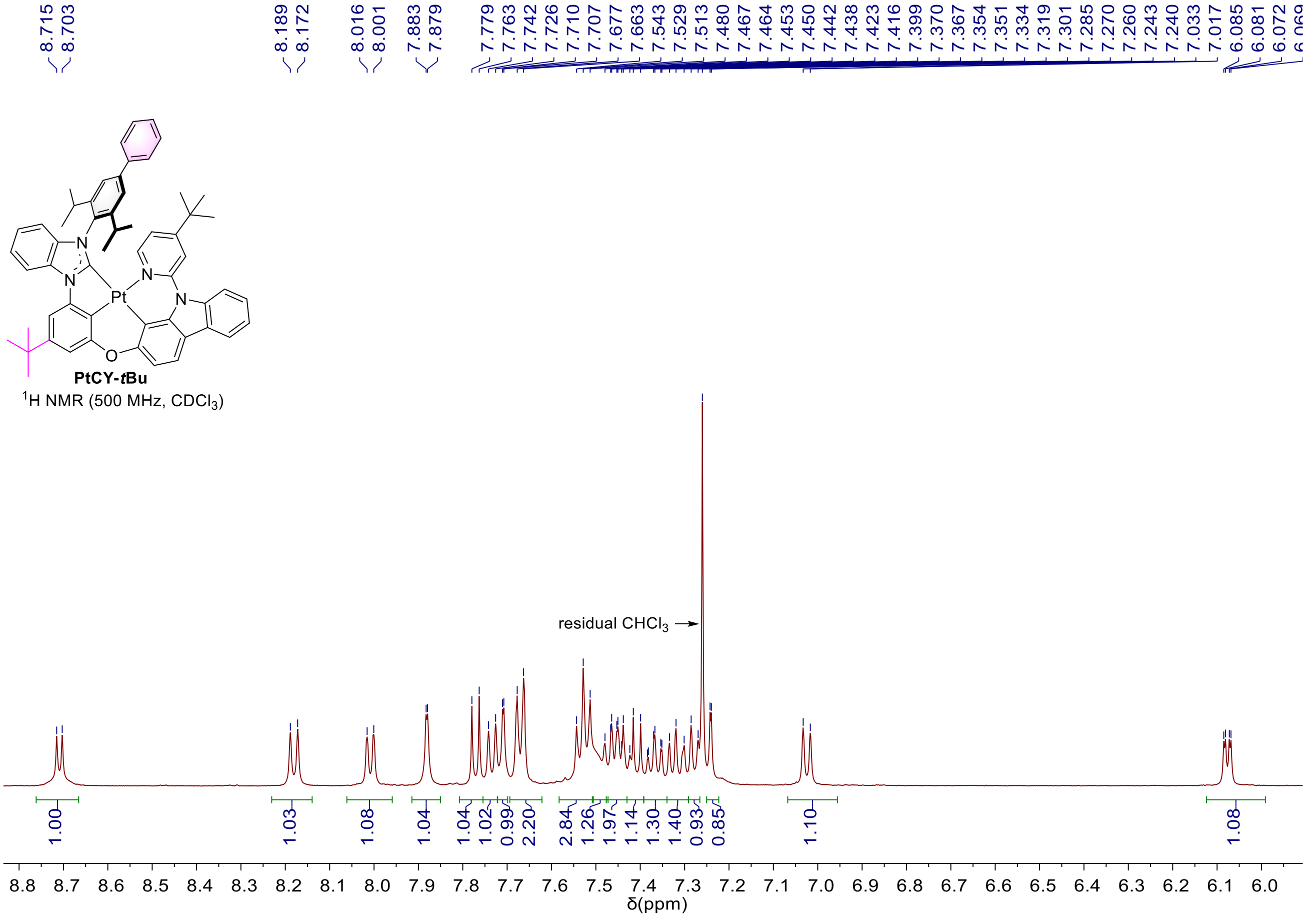
8.715
8.703
8.189
8.172
8.016
8.001
7.883
7.879
7.779
7.763
7.742
7.726
7.710
7.707
7.677
7.663
7.543
7.529
7.513
7.480
7.467
7.464
7.453
7.450
7.442
7.438
7.423
7.416
7.399
7.370
7.367
7.354
7.351
7.334
7.319
7.301
7.285
7.270
7.260
7.243
7.240
7.033
7.017
6.085
6.081
6.072
PtCY-tBu
^{1}H NMR (500 MHz, $CDCl_3$)
residual $CHCl_3$
1.00
1.03
1.08
1.04
1.04
1.02
0.99
2.20
2.84
1.26
1.97
1.14
1.30
1.40
0.93
0.85
1.10
1.08
δ(ppm)

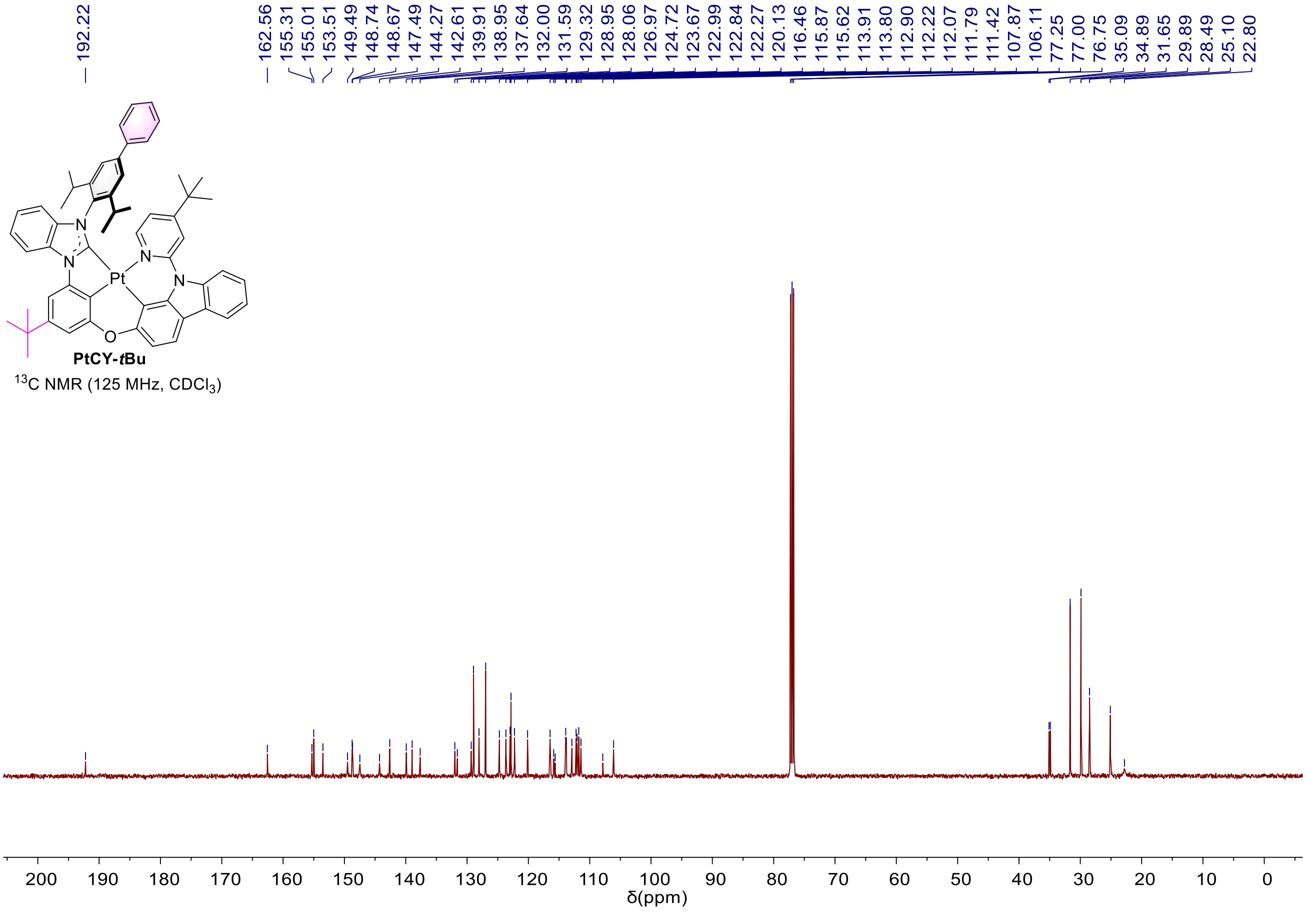
192.22
162.56
155.31
155.01
153.51
149.49
148.74
148.67
147.49
144.27
142.61
139.91
138.95
137.64
132.00
131.59
129.32
128.95
128.06
126.97
124.72
123.67
122.99
122.84
122.27
120.13
116.46
115.87
115.62
113.91
113.80
112.90
112.22
112.07
111.79
111.42
107.87
106.11
77.25
77.00
76.75
35.09
34.89
31.65
29.89
28.49
25.10
22.80
PtCY-tBu
^{13}C NMR (125 MHz, $CDCl_3$)
δ(ppm)

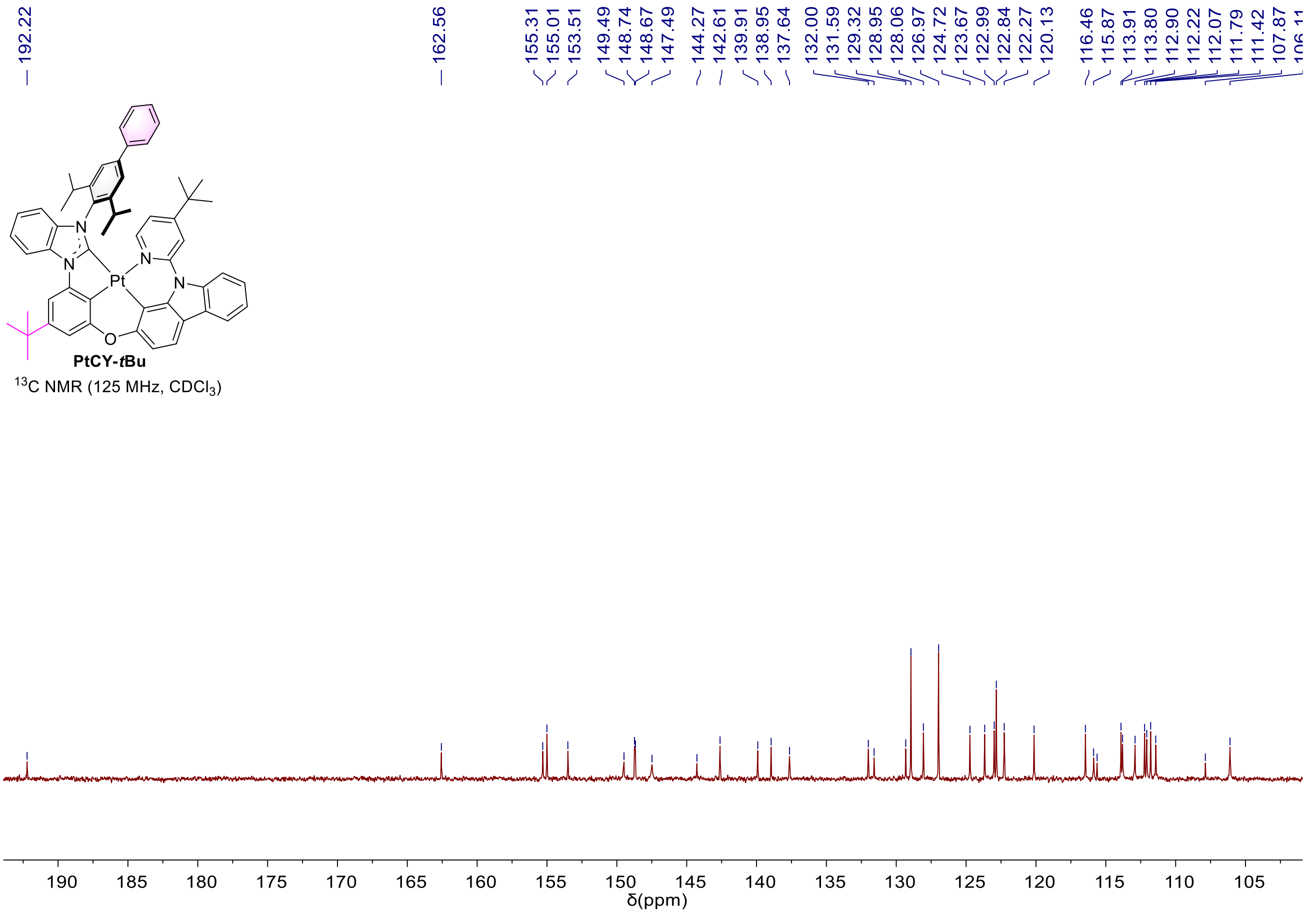
192.22
162.56
155.31
155.01
153.51
149.49
148.74
148.67
147.49
144.27
142.61
139.91
138.95
137.64
132.00
131.59
129.32
128.95
128.06
126.97
124.72
123.67
122.99
122.84
122.27
120.13
116.46
115.87
113.91
113.80
112.90
112.22
112.07
111.79
111.42
107.87
PtCY-tBu
^{13}C NMR (125 MHz, CDCl$_3$)
δ(ppm)

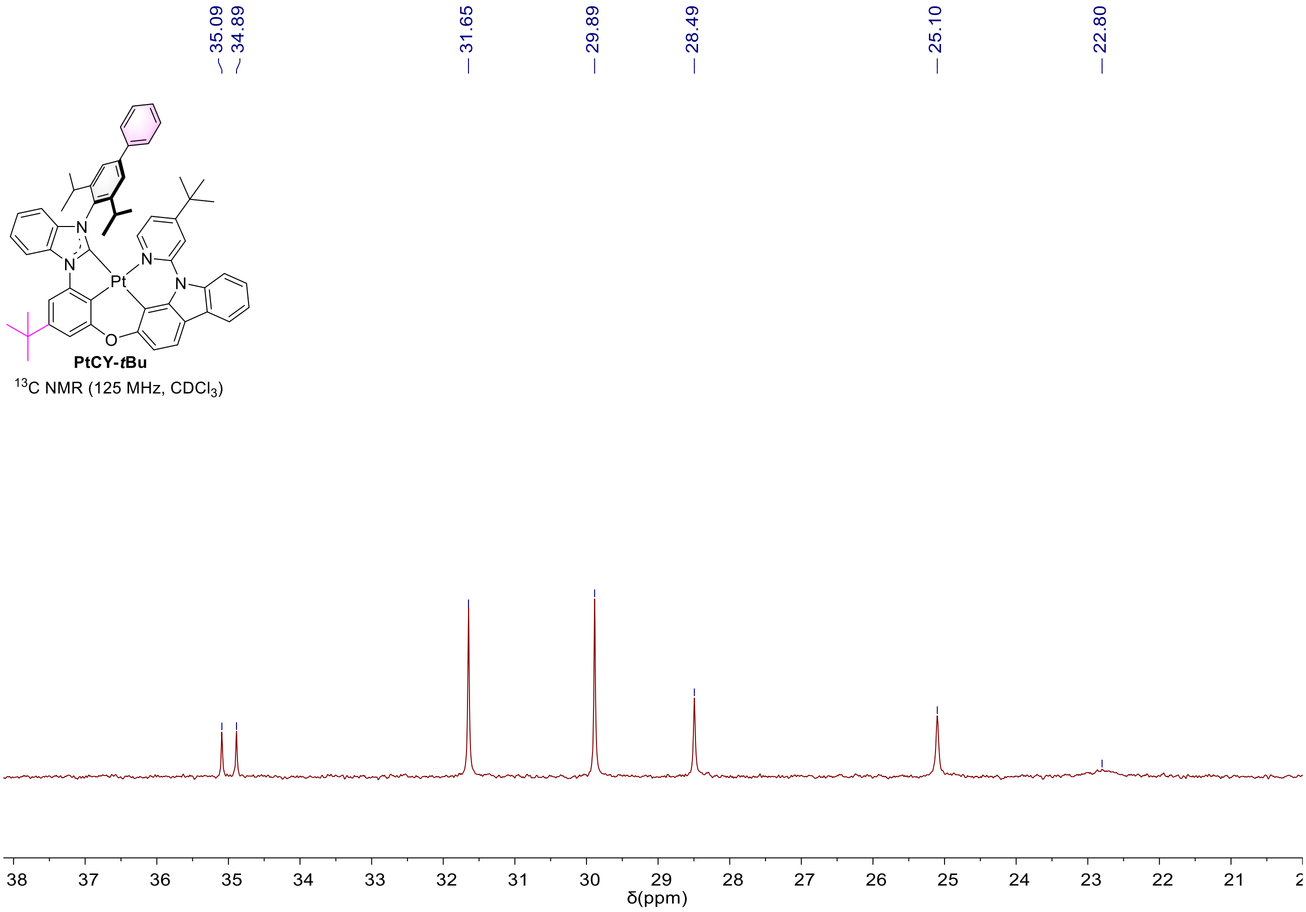
35.09
34.89
31.65
29.89
28.49
25.10
22.80
PtCY-tBu
^{13}C NMR (125 MHz, CDCl$_3$)
δ(ppm)

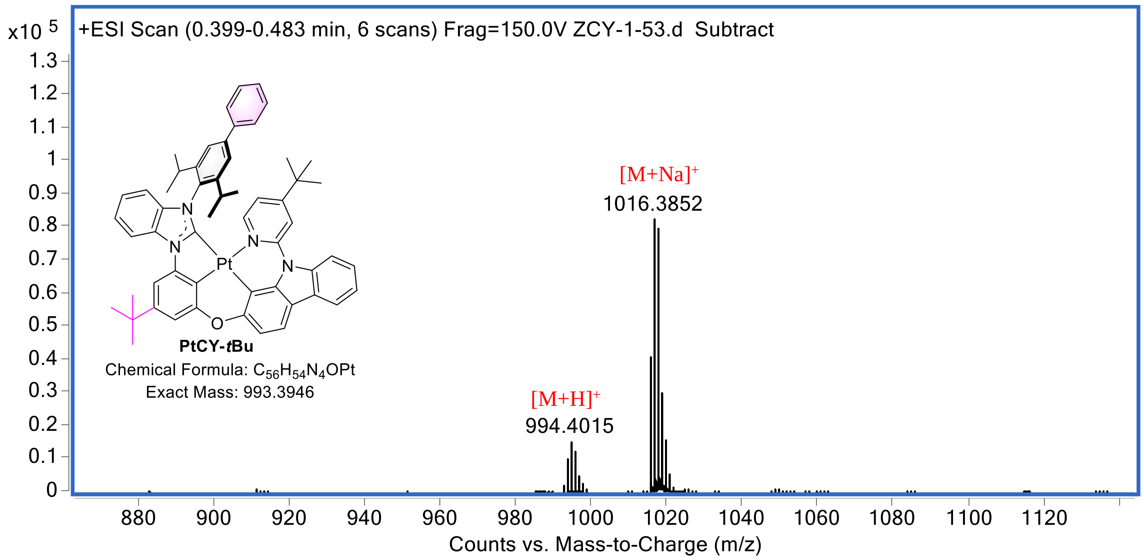
x10 5
+ESI Scan (0.399-0.483 min, 6 scans) Frag=150.0V ZCY-1-53.d Subtract
[M+Na]+
1016.3852
[M+H]+
994.4015
PtCY-tBu
Chemical Formula: C56H54N4OPt
Exact Mass: 993.3946
Counts vs. Mass-to-Charge (m/z)

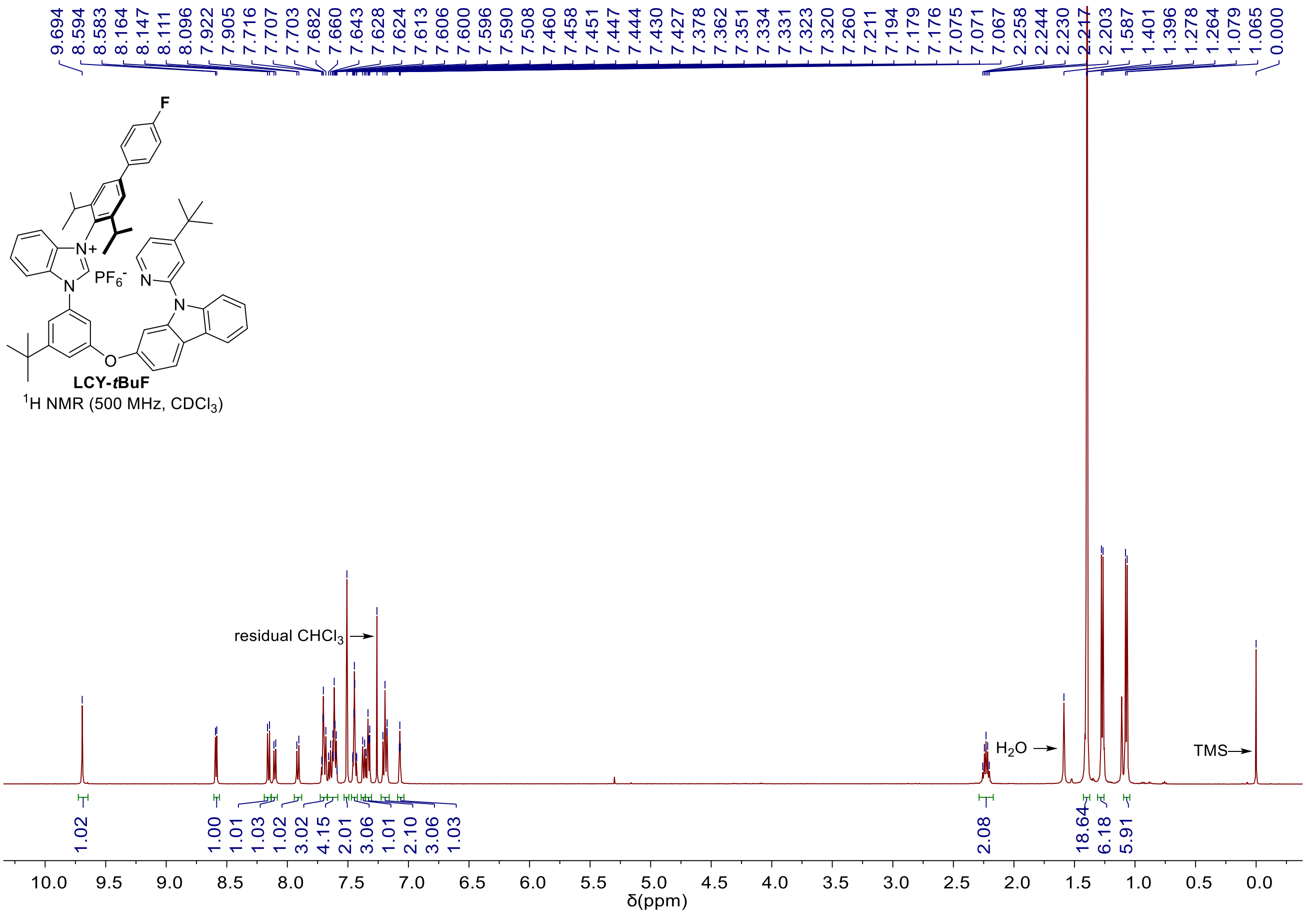
LCY-tBuF
1H NMR (500 MHz, CDCl3)
residual CHCl3
H2O
TMS
δ(ppm)

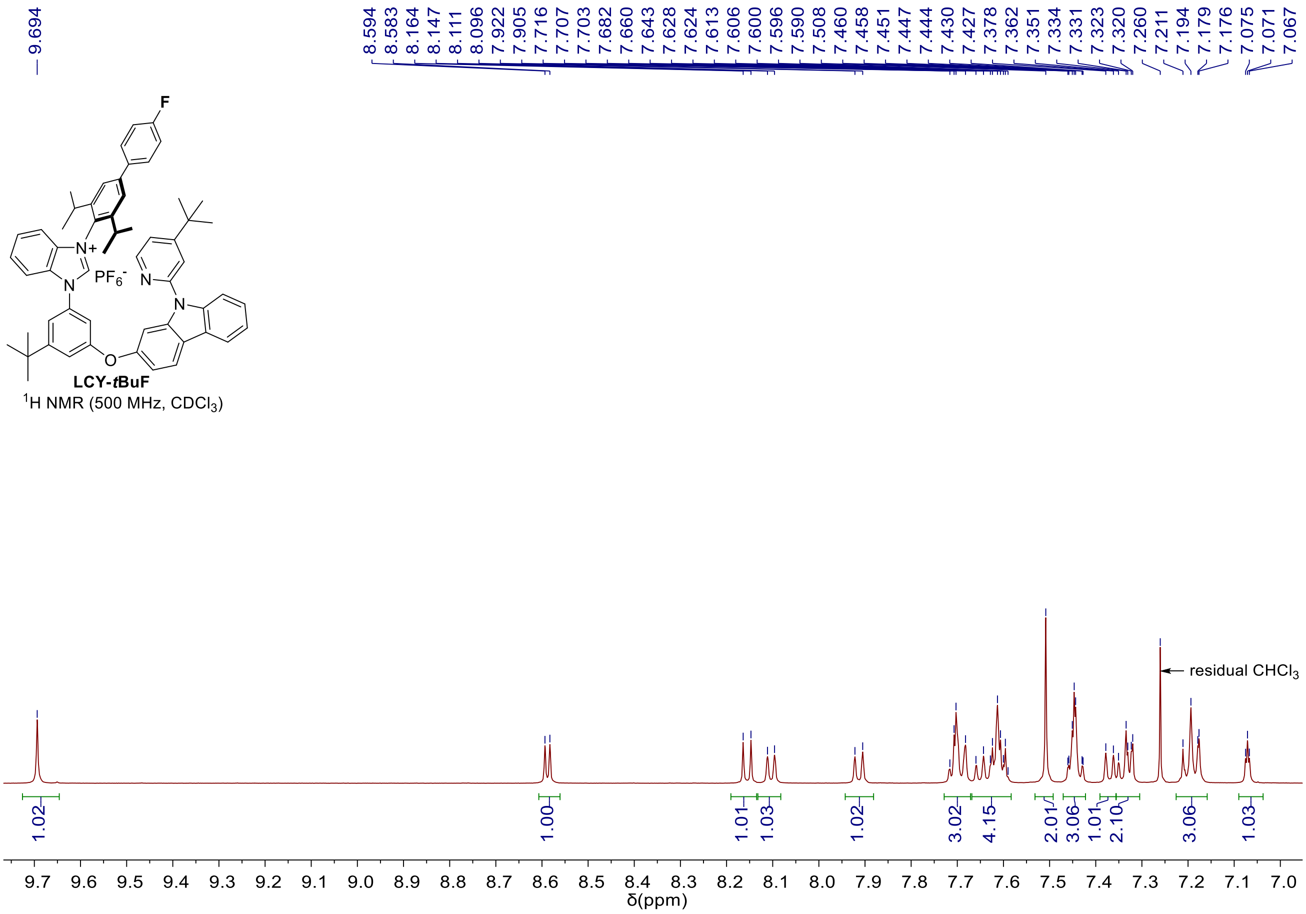

LCY-*t*BuF

^{1}H NMR (500 MHz, $CDCl_3$)

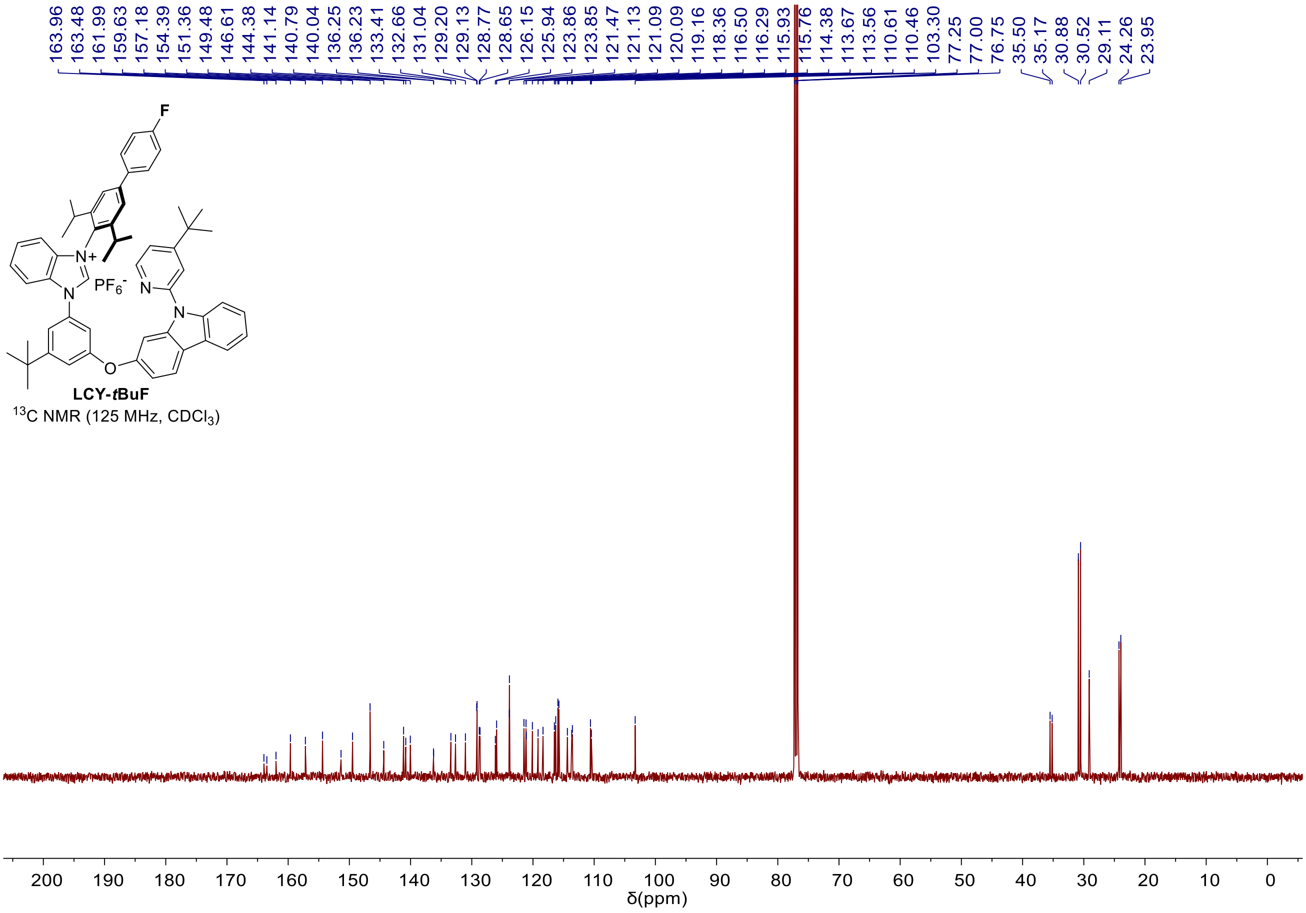

LCY-*t*BuF

^{13}C NMR (125 MHz, $CDCl_3$)

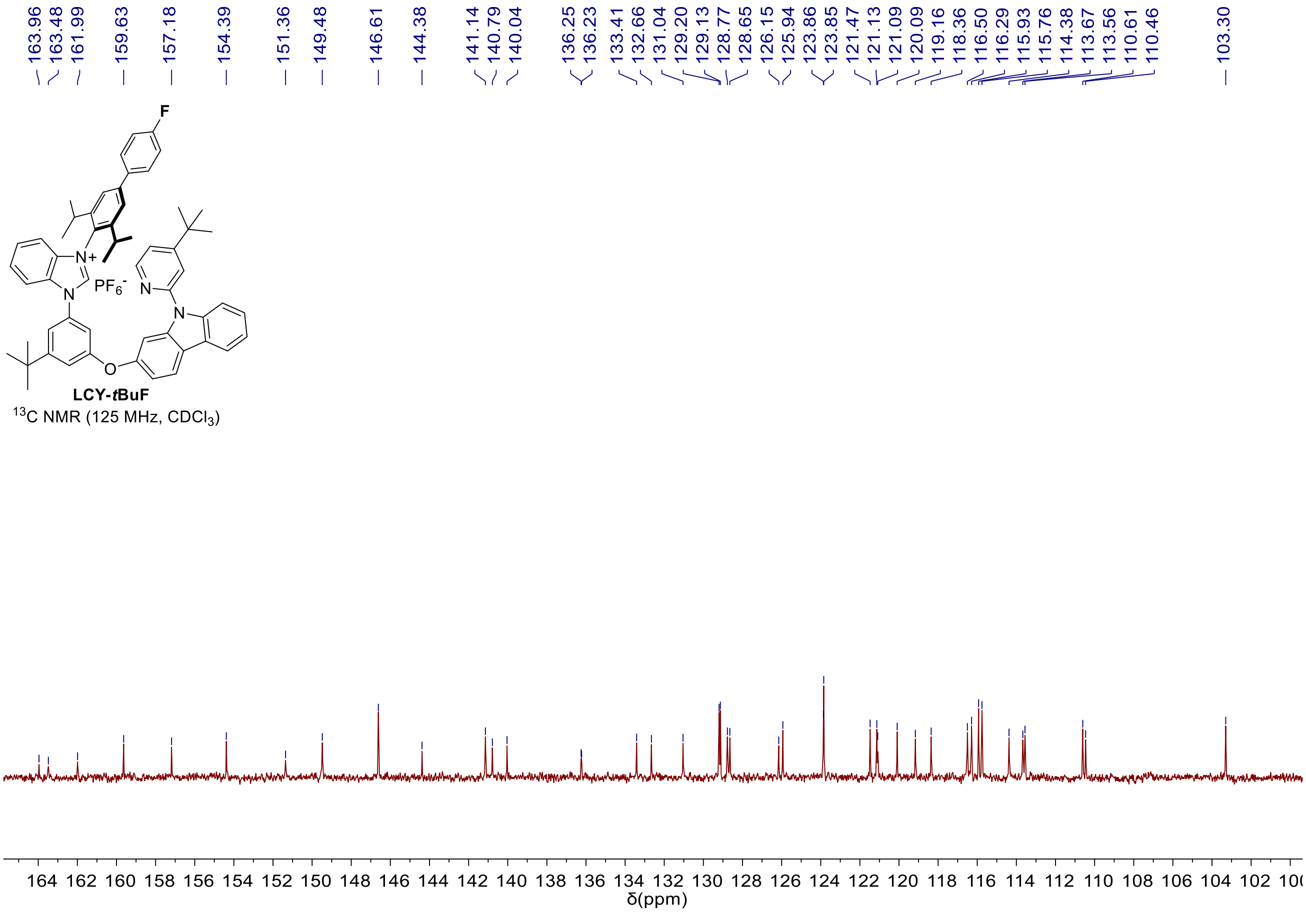
163.96
163.48
161.99
159.63
157.18
154.39
151.36
149.48
146.61
144.38
141.14
140.79
140.04
136.25
136.23
133.41
132.66
131.04
129.20
129.13
128.77
128.65
126.15
125.94
123.86
123.85
121.47
121.13
121.09
120.09
119.16
118.36
116.50
116.29
115.93
115.76
114.38
113.67
113.56
110.61
110.46
103.30
F
N+
N
PF6-
N
N
O
LCY-tBuF
13C NMR (125 MHz, CDCl3)
δ(ppm)

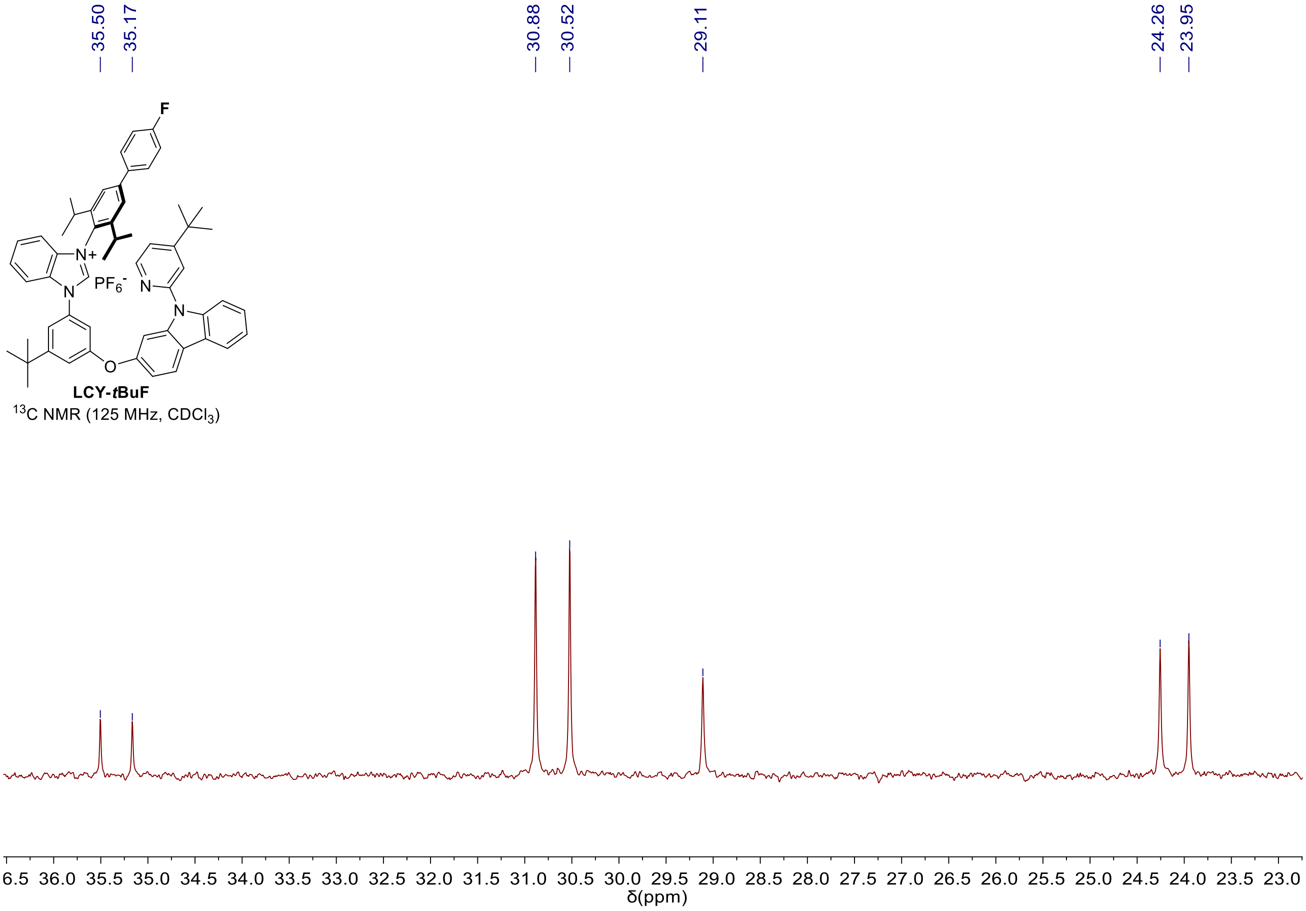
35.50
35.17
30.88
30.52
29.11
24.26
23.95
F
N+
N
PF6-
N
N
O
LCY-tBuF
13C NMR (125 MHz, CDCl3)
δ(ppm)

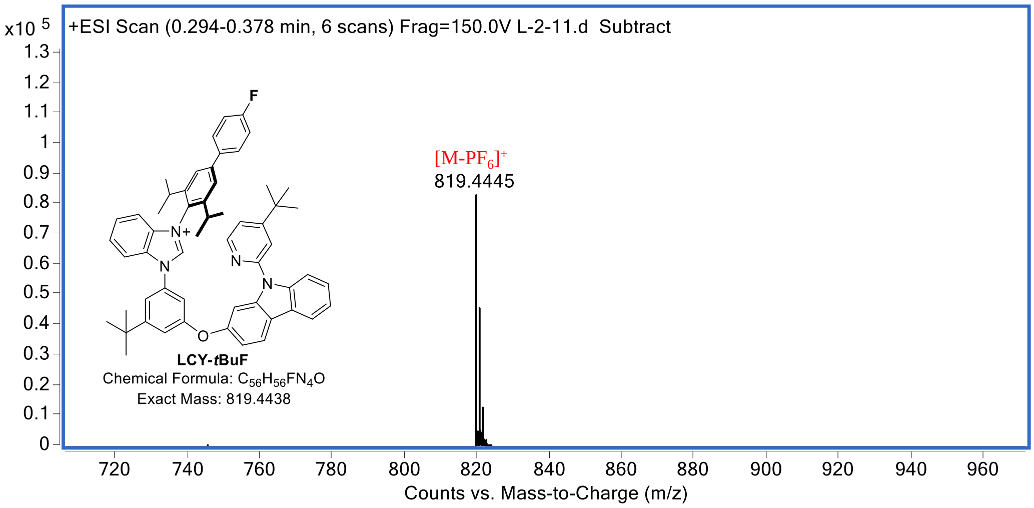

x10 5
+ESI Scan (0.294-0.378 min, 6 scans) Frag=150.0V L-2-11.d Subtract
[M-PF6]+
819.4445
LCY-tBuF
Chemical Formula: C56H56FN4O
Exact Mass: 819.4438
Counts vs. Mass-to-Charge (m/z)

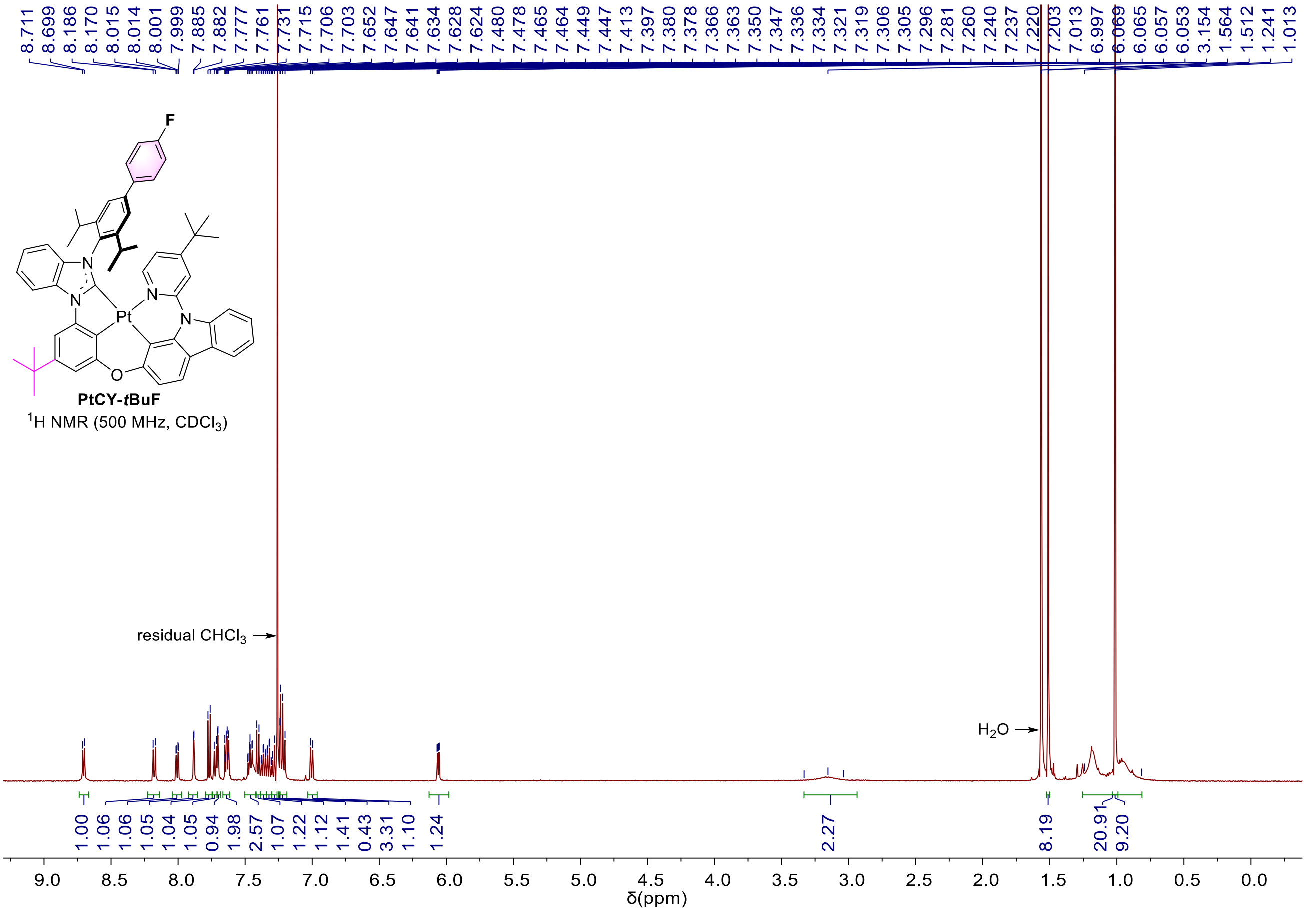

PtCY-tBuF
1H NMR (500 MHz, CDCl3)
residual CHCl3
H2O
δ(ppm)

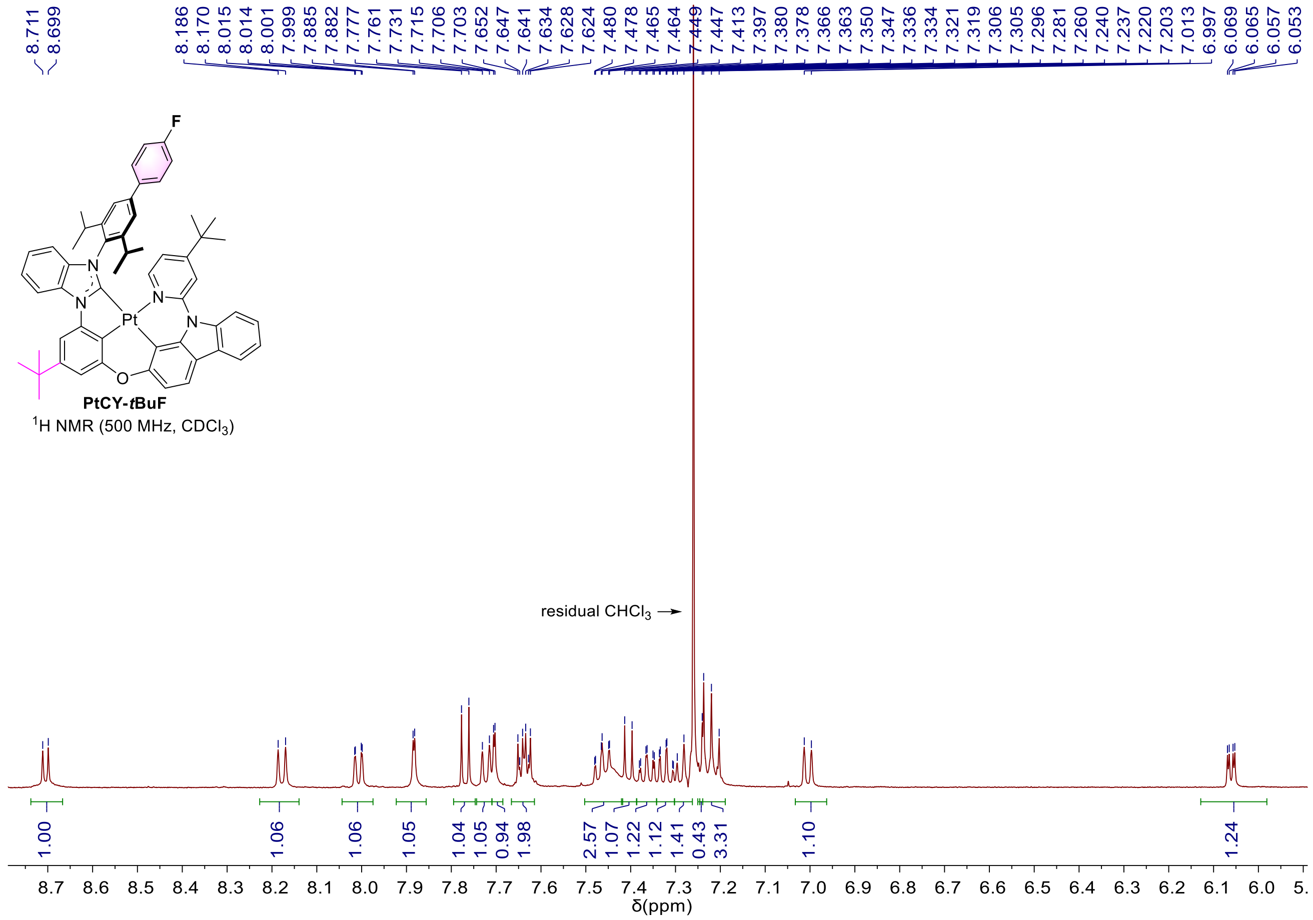

8.711
8.699
8.186
8.170
8.015
8.014
8.001
7.999
7.885
7.882
7.777
7.761
7.731
7.715
7.706
7.703
7.652
7.647
7.641
7.634
7.628
7.624
7.480
7.478
7.465
7.464
7.449
7.447
7.413
7.397
7.380
7.378
7.366
7.363
7.350
7.347
7.336
7.334
7.321
7.319
7.306
7.305
7.296
7.281
7.260
7.240
7.237
7.220
7.203
7.013
6.997
6.069
6.065
6.057
6.053
F
N
N
Pt
N
N
O
PtCY-tBuF
1H NMR (500 MHz, CDCl3)
residual CHCl3
1.00
1.06
1.06
1.05
1.04
1.05
0.94
1.98
2.57
1.07
1.22
1.12
1.41
0.43
3.31
1.10
1.24
δ(ppm)

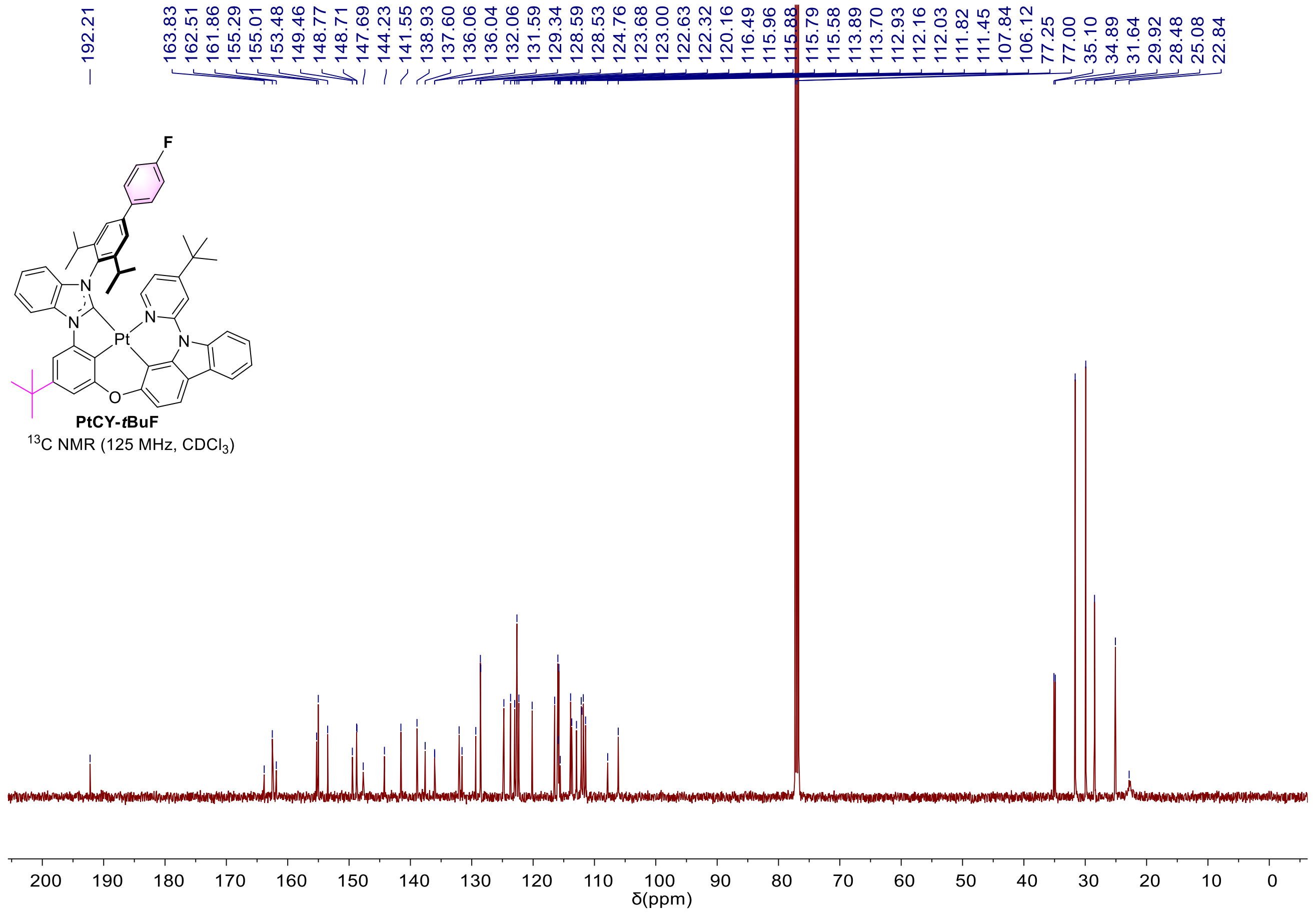

192.21
163.83
162.51
161.86
155.29
155.01
153.48
149.46
148.77
148.71
147.69
144.23
141.55
138.93
137.60
136.06
136.04
132.06
131.59
129.34
128.59
128.53
124.76
123.68
123.00
122.63
122.32
120.16
116.49
115.96
115.88
115.79
115.58
113.89
113.70
112.93
112.16
112.03
111.82
111.45
107.84
106.12
77.25
77.00
35.10
34.89
31.64
29.92
28.48
25.08
22.84
F
N
N
Pt
N
N
O
PtCY-tBuF
13C NMR (125 MHz, CDCl3)
δ(ppm)

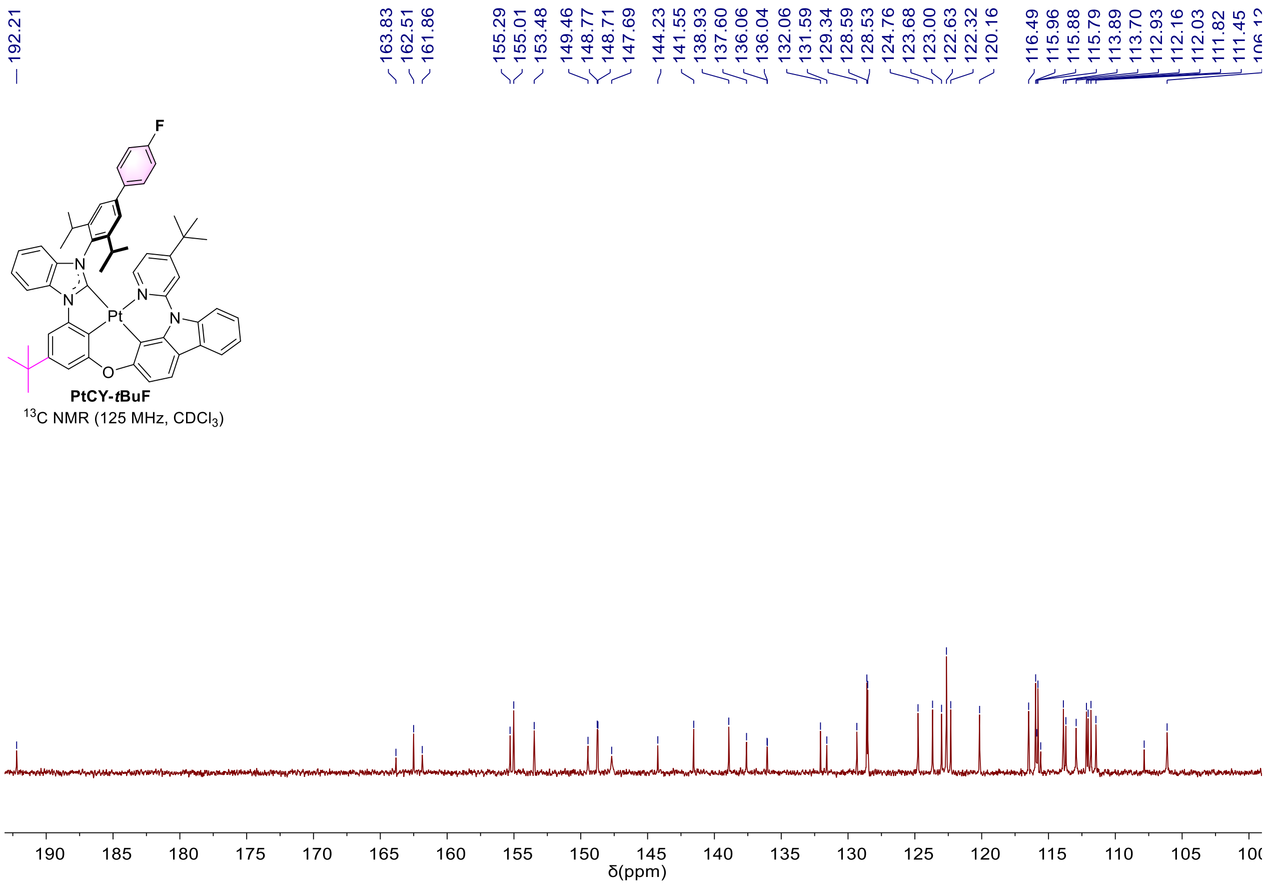
192.21
163.83
162.51
161.86
155.29
155.01
153.48
149.46
148.77
148.71
147.69
144.23
141.55
138.93
137.60
136.06
136.04
132.06
131.59
129.34
128.59
128.53
124.76
123.68
123.00
122.63
122.32
120.16
116.49
115.96
115.88
115.79
113.89
113.70
112.93
112.16
112.03
111.82
111.45
F
N
N
Pt
N
N
O
PtCY-tBuF
13C NMR (125 MHz, CDCl3)
190
185
180
175
170
165
160
155
150
145
140
135
130
125
120
115
110
105
δ(ppm)

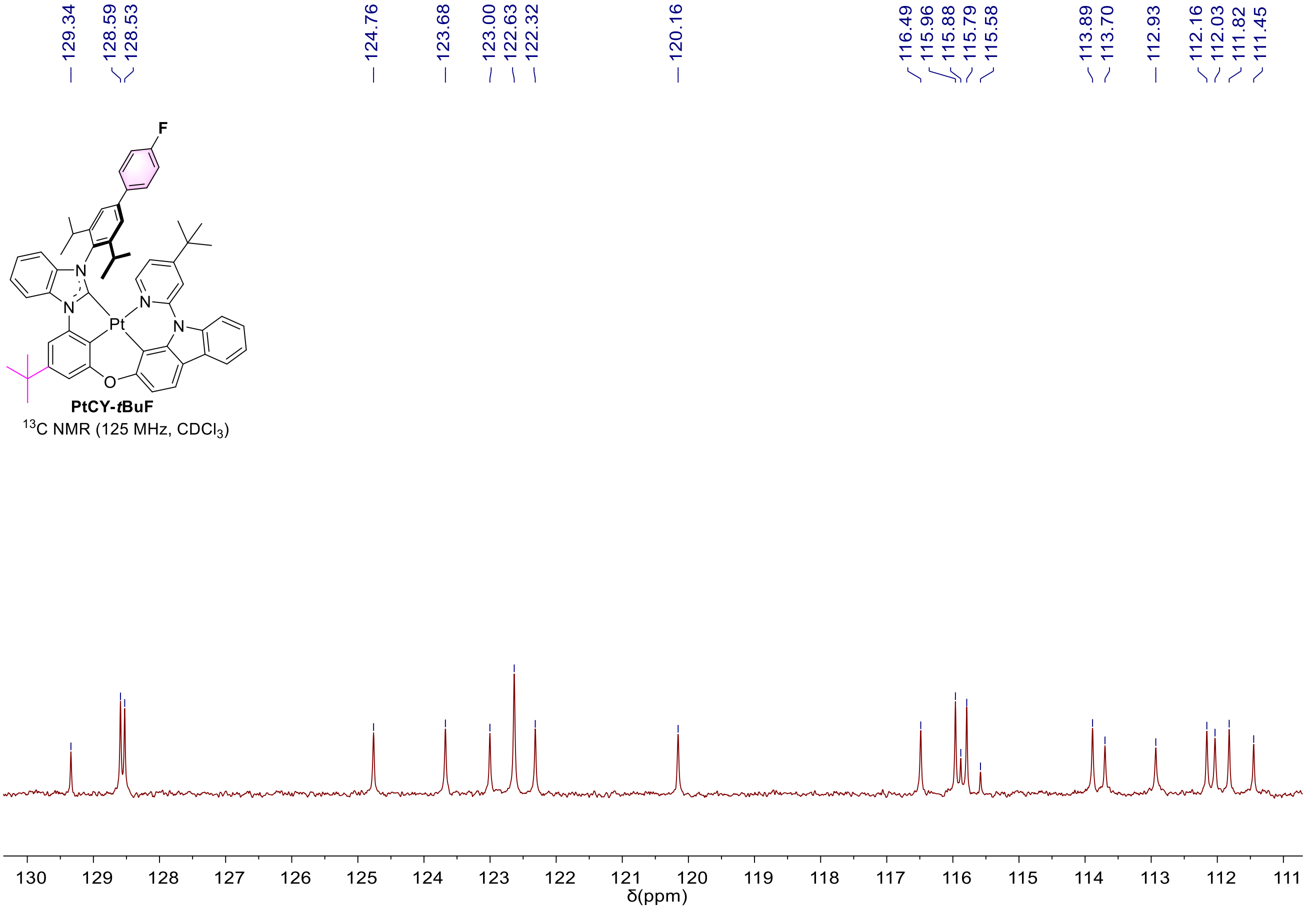
129.34
128.59
128.53
124.76
123.68
123.00
122.63
122.32
120.16
116.49
115.96
115.88
115.79
115.58
113.89
113.70
112.93
112.16
112.03
111.82
111.45
F
N
N
Pt
N
N
O
PtCY-tBuF
13C NMR (125 MHz, CDCl3)
130
129
128
127
126
125
124
123
122
121
120
119
118
117
116
115
114
113
112
111
δ(ppm)

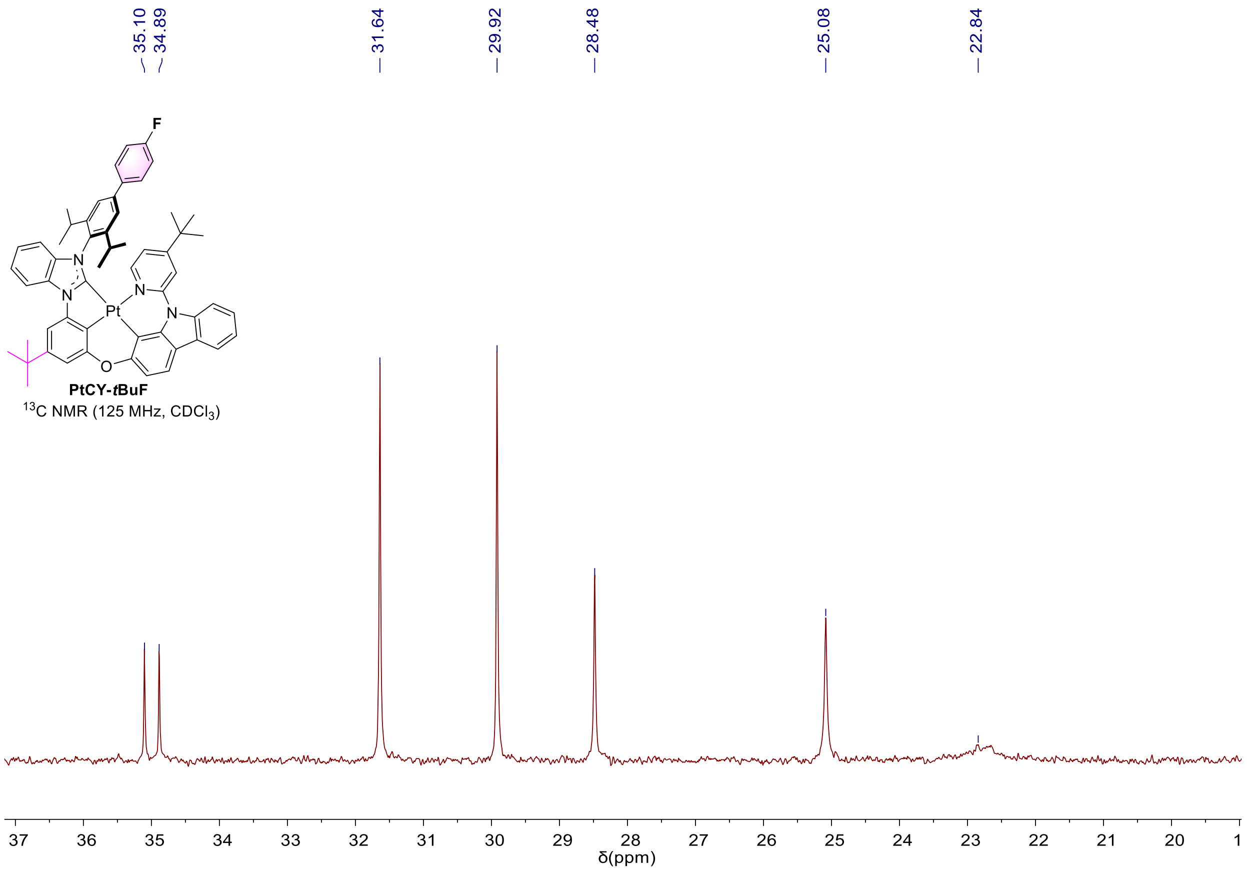
35.10
34.89
31.64
29.92
28.48
25.08
22.84
F
N
N
Pt
N
N
O
PtCY-tBuF
13C NMR (125 MHz, CDCl3)
37
36
35
34
33
32
31
30
29
28
27
26
25
24
23
22
21
20
δ(ppm)

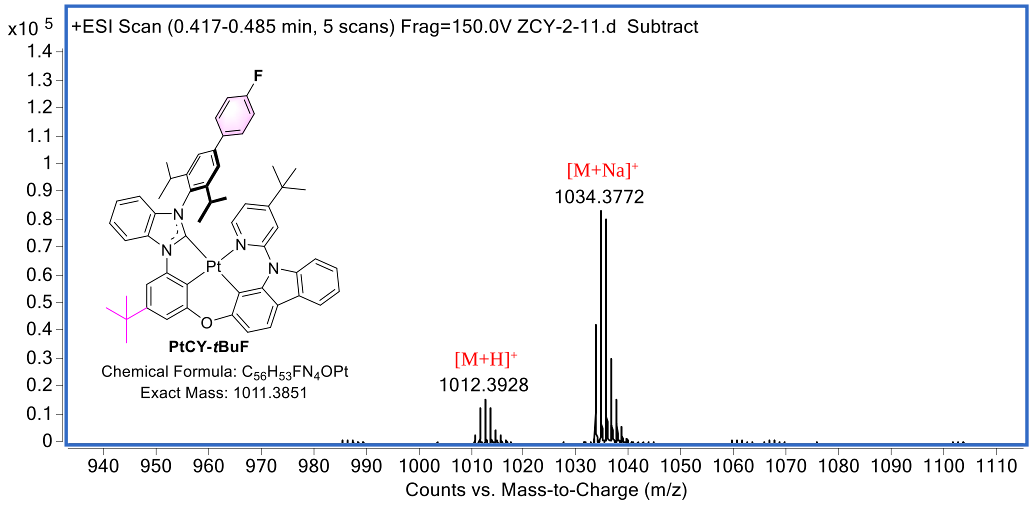
x10 5
+ESI Scan (0.417-0.485 min, 5 scans) Frag=150.0V ZCY-2-11.d Subtract
F
N
N
Pt
N
N
O
PtCY-tBuF
Chemical Formula: C56H53FN4OPt
Exact Mass: 1011.3851
[M+Na]+
1034.3772
[M+H]+
1012.3928
1.4
1.3
1.2
1.1
1
0.9
0.8
0.7
0.6
0.5
0.4
0.3
0.2
0.1
0
940
950
960
970
980
990
1000
1010
1020
1030
1040
1050
1060
1070
1080
1090
1100
1110
Counts vs. Mass-to-Charge (m/z)

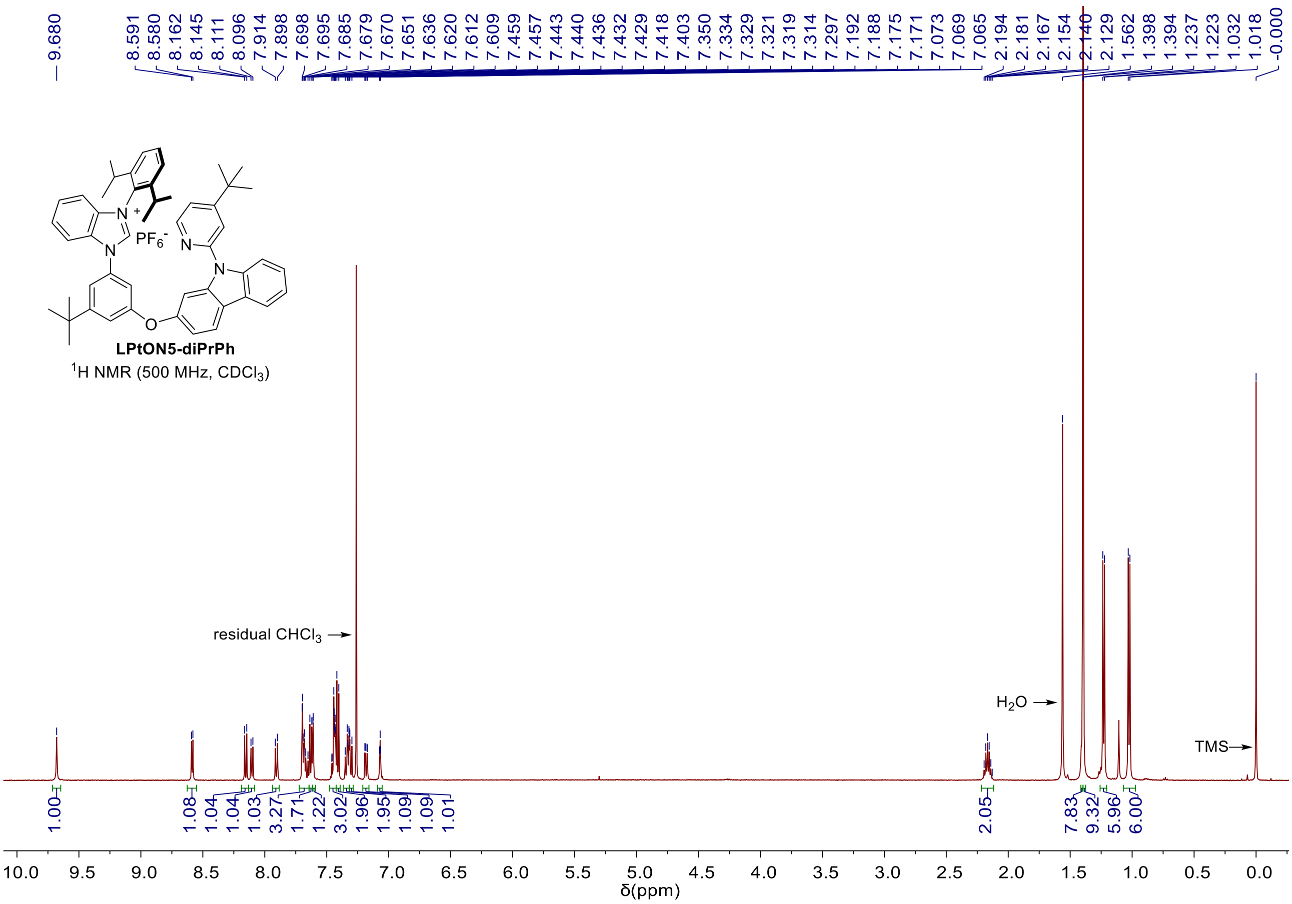

9.680
8.591 8.580 8.162 8.145 8.111 8.096 7.914 7.898 7.698 7.695 7.685 7.679 7.670 7.651 7.636 7.620 7.612 7.609 7.459 7.457 7.443 7.440 7.436 7.432 7.429 7.418 7.403 7.350 7.334 7.329 7.321 7.319 7.314 7.297 7.192 7.188 7.175 7.171 7.073 7.069 7.065 2.194 2.181 2.167 2.154 2.140 2.129 1.562 1.398 1.394 1.237 1.223 1.032 1.018 -0.000
PF6-
LPtON5-diPrPh
1H NMR (500 MHz, CDCl3)
residual CHCl3
H2O
TMS
1.00 1.08 1.04 1.04 1.03 3.27 1.71 1.22 3.02 1.96 1.95 1.09 1.01 2.05 7.83 9.32 5.96 6.00
δ(ppm)

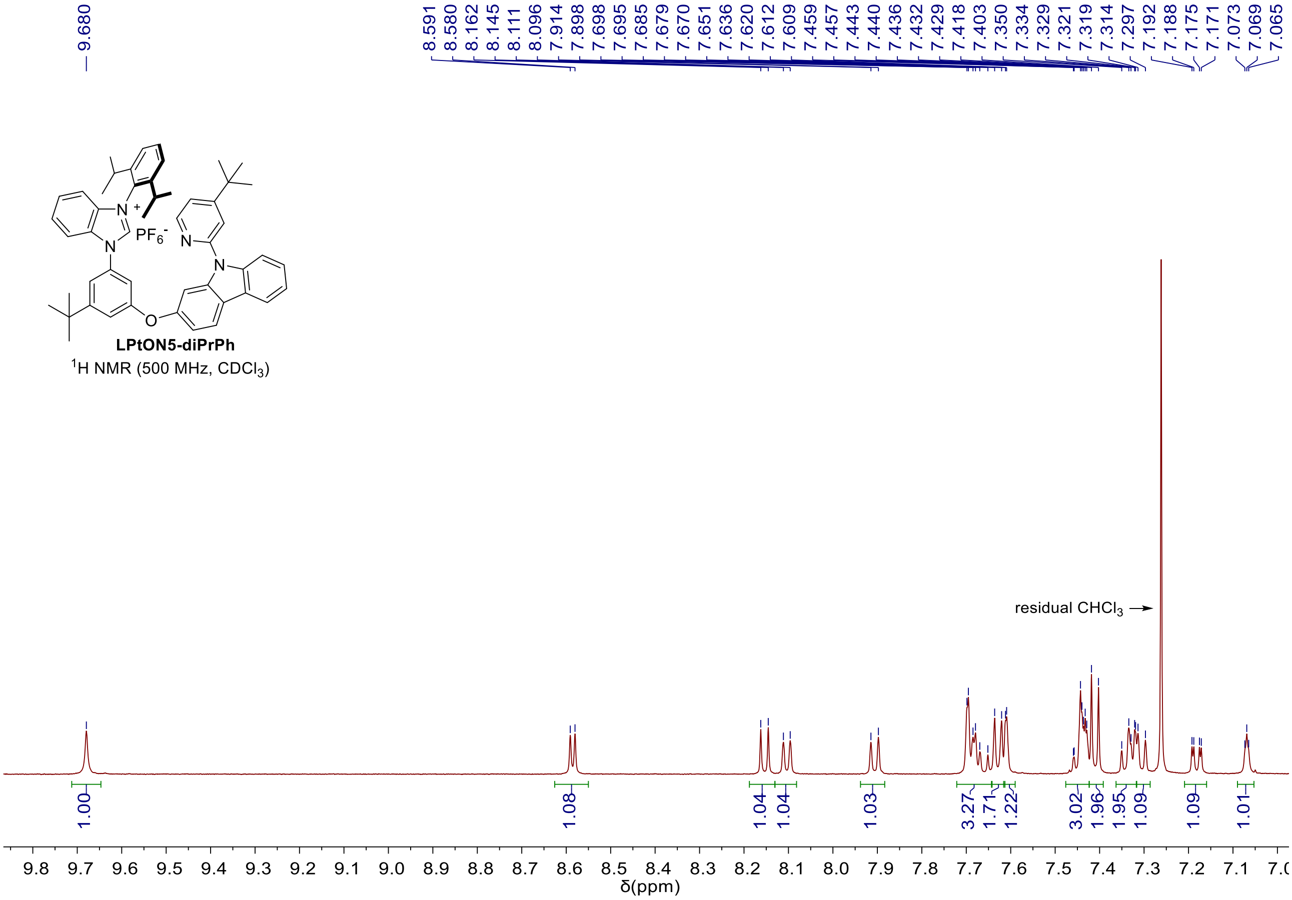

9.680
8.591 8.580 8.162 8.145 8.111 8.096 7.914 7.898 7.698 7.695 7.685 7.679 7.670 7.651 7.636 7.620 7.612 7.609 7.459 7.457 7.443 7.440 7.436 7.432 7.429 7.418 7.403 7.350 7.334 7.329 7.321 7.319 7.314 7.297 7.192 7.188 7.175 7.171 7.073 7.069 7.065
PF6-
LPtON5-diPrPh
1H NMR (500 MHz, CDCl3)
residual CHCl3
1.00 1.08 1.04 1.04 1.03 3.27 1.71 1.22 3.02 1.96 1.95 1.09 1.01
δ(ppm)

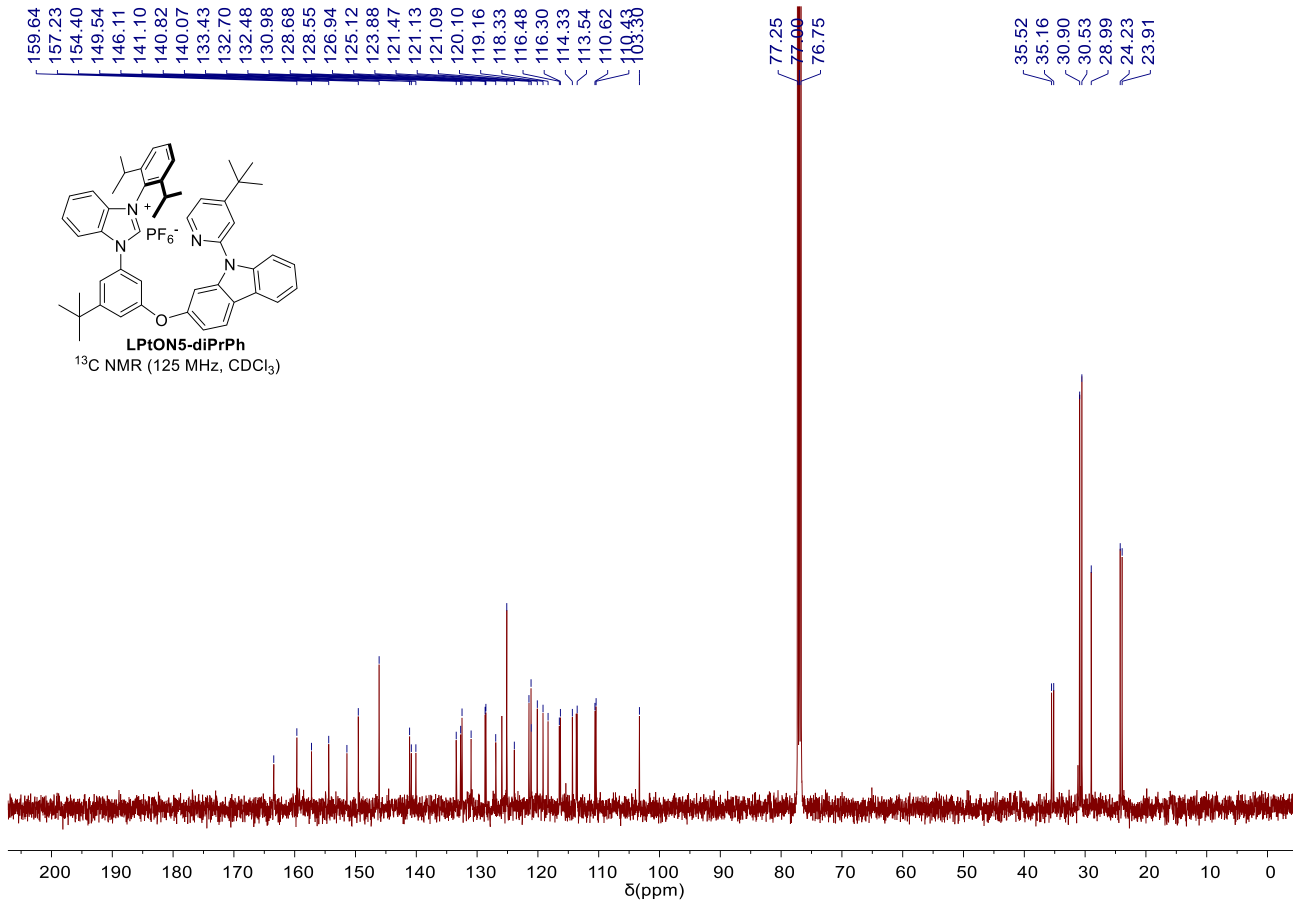
159.64
157.23
154.40
149.54
146.11
141.10
140.82
140.07
133.43
132.70
132.48
130.98
128.68
128.55
126.94
125.12
123.88
121.47
121.13
121.09
120.10
119.16
118.33
116.48
116.30
114.33
113.54
110.62
110.43
103.30
77.25
77.00
76.75
35.52
35.16
30.90
30.53
28.99
24.23
23.91
LPtON5-diPrPh
^{13}C NMR (125 MHz, $CDCl_3$)
δ(ppm)

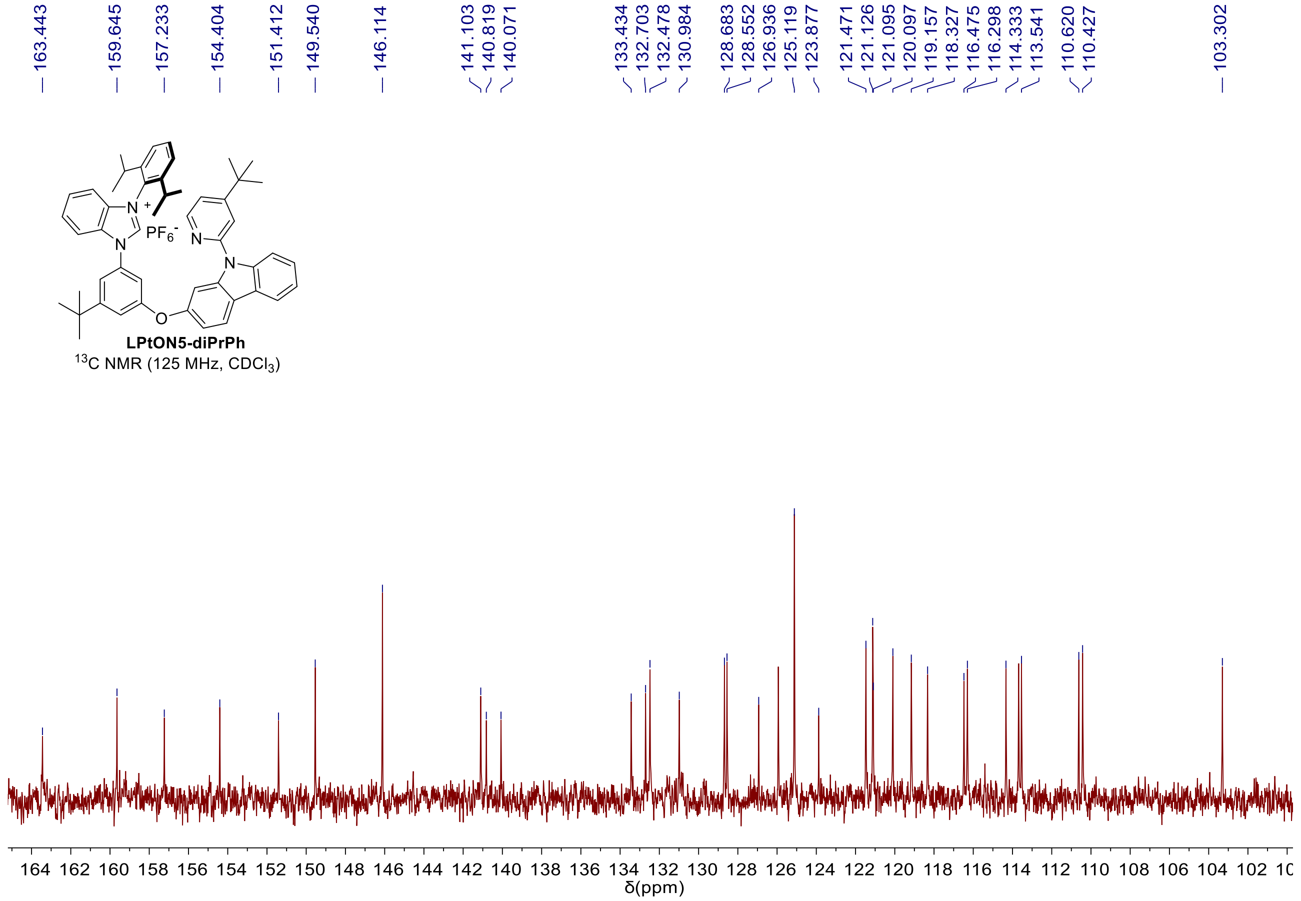
163.443
159.645
157.233
154.404
151.412
149.540
146.114
141.103
140.819
140.071
133.434
132.703
132.478
130.984
128.683
128.552
126.936
125.119
123.877
121.471
121.126
121.095
120.097
119.157
118.327
116.475
116.298
114.333
113.541
110.620
110.427
103.302
LPtON5-diPrPh
^{13}C NMR (125 MHz, $CDCl_3$)
δ(ppm)

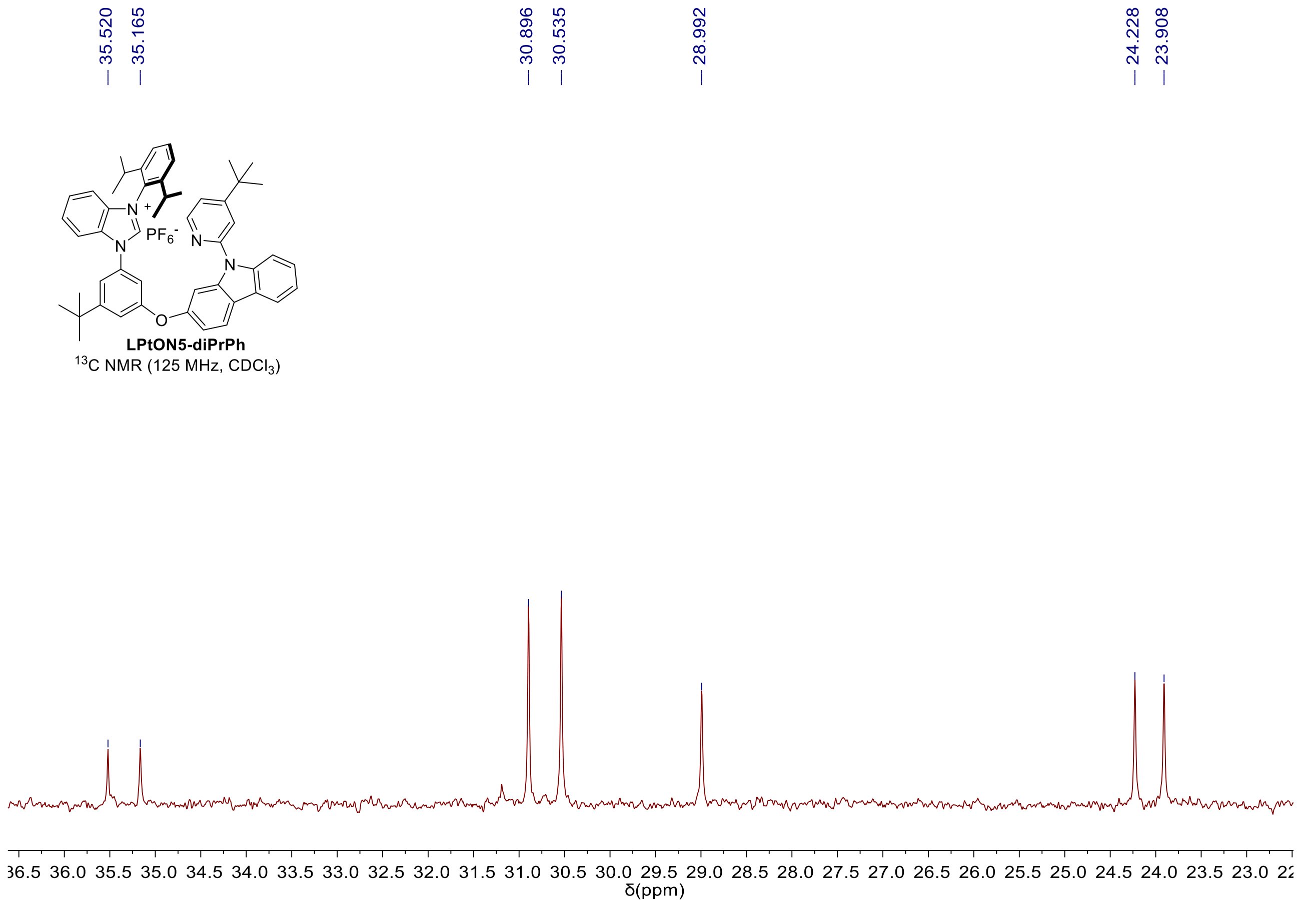

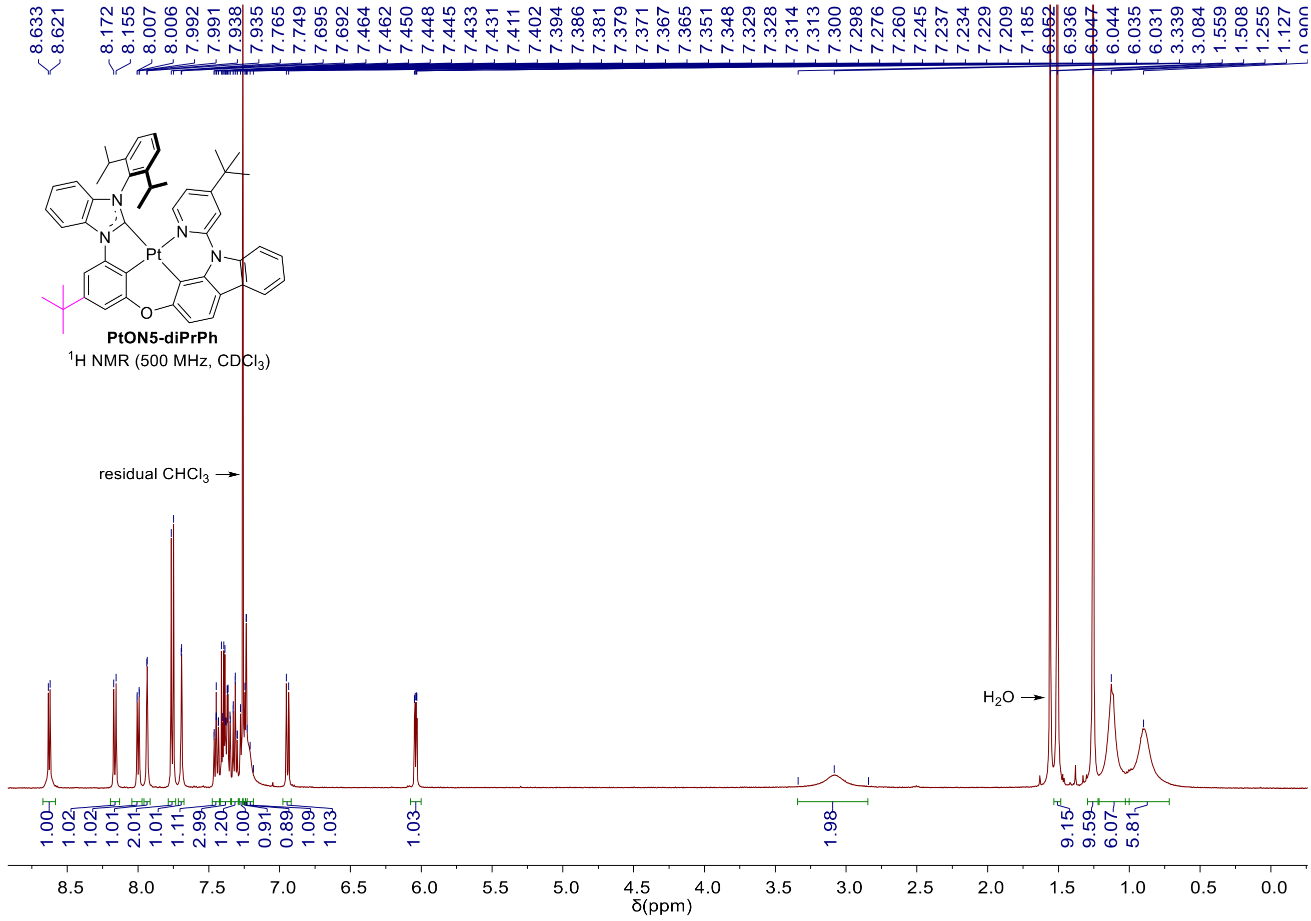

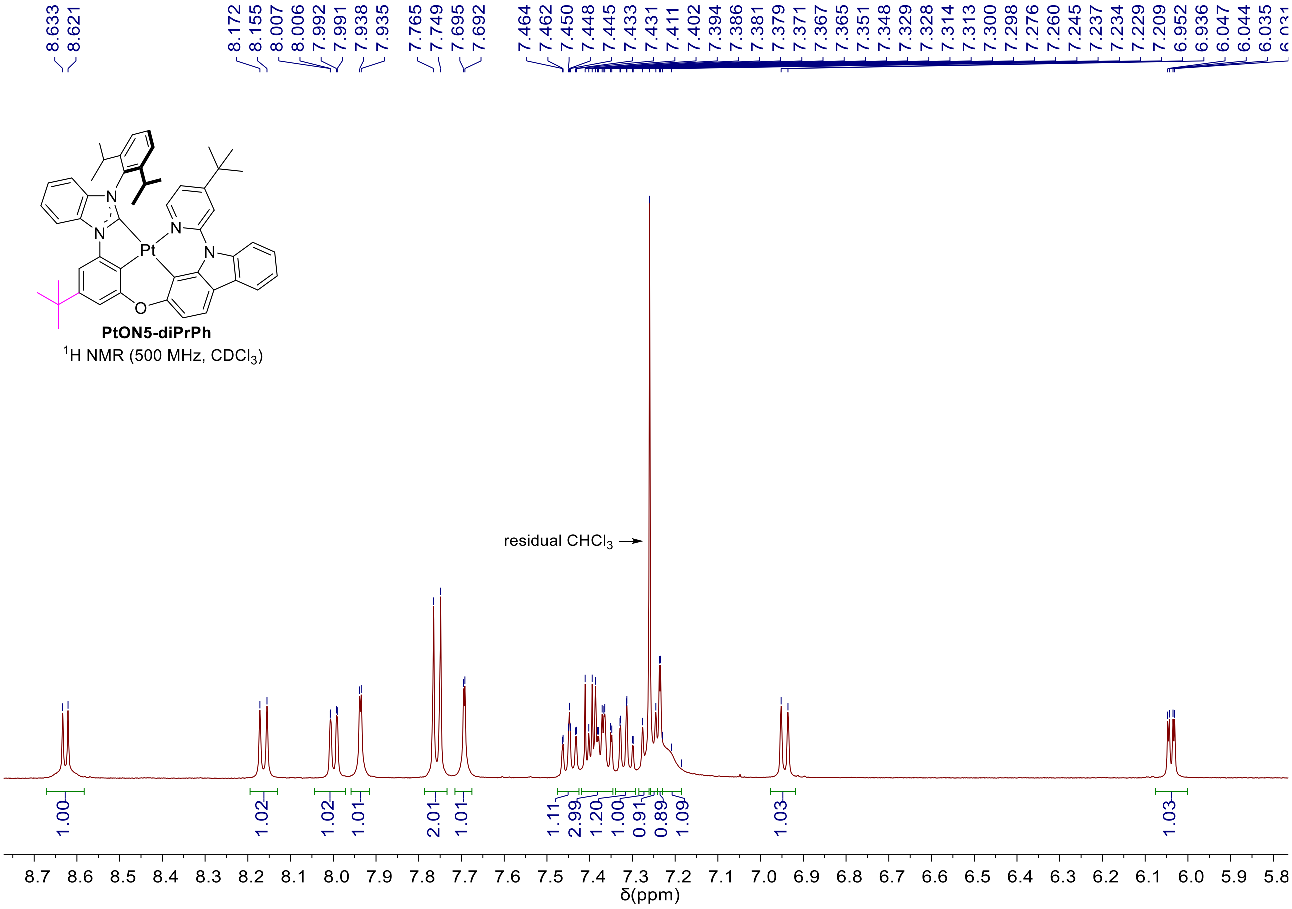
8.633 8.621 8.172 8.155 8.007 8.006 7.992 7.991 7.938 7.935 7.765 7.749 7.695 7.692 7.464 7.462 7.450 7.448 7.445 7.433 7.431 7.411 7.402 7.394 7.386 7.381 7.379 7.371 7.367 7.365 7.351 7.348 7.329 7.328 7.314 7.313 7.300 7.298 7.276 7.260 7.245 7.237 7.234 7.229 7.209 6.952 6.936 6.047 6.044 6.035
PtON5-diPrPh
^{1}H NMR (500 MHz, CDCl$_3$)
residual CHCl$_3$
1.00 1.02 1.02 1.01 2.01 1.01 1.11 2.99 1.20 1.00 0.91 0.89 1.09 1.03 1.03
δ(ppm)

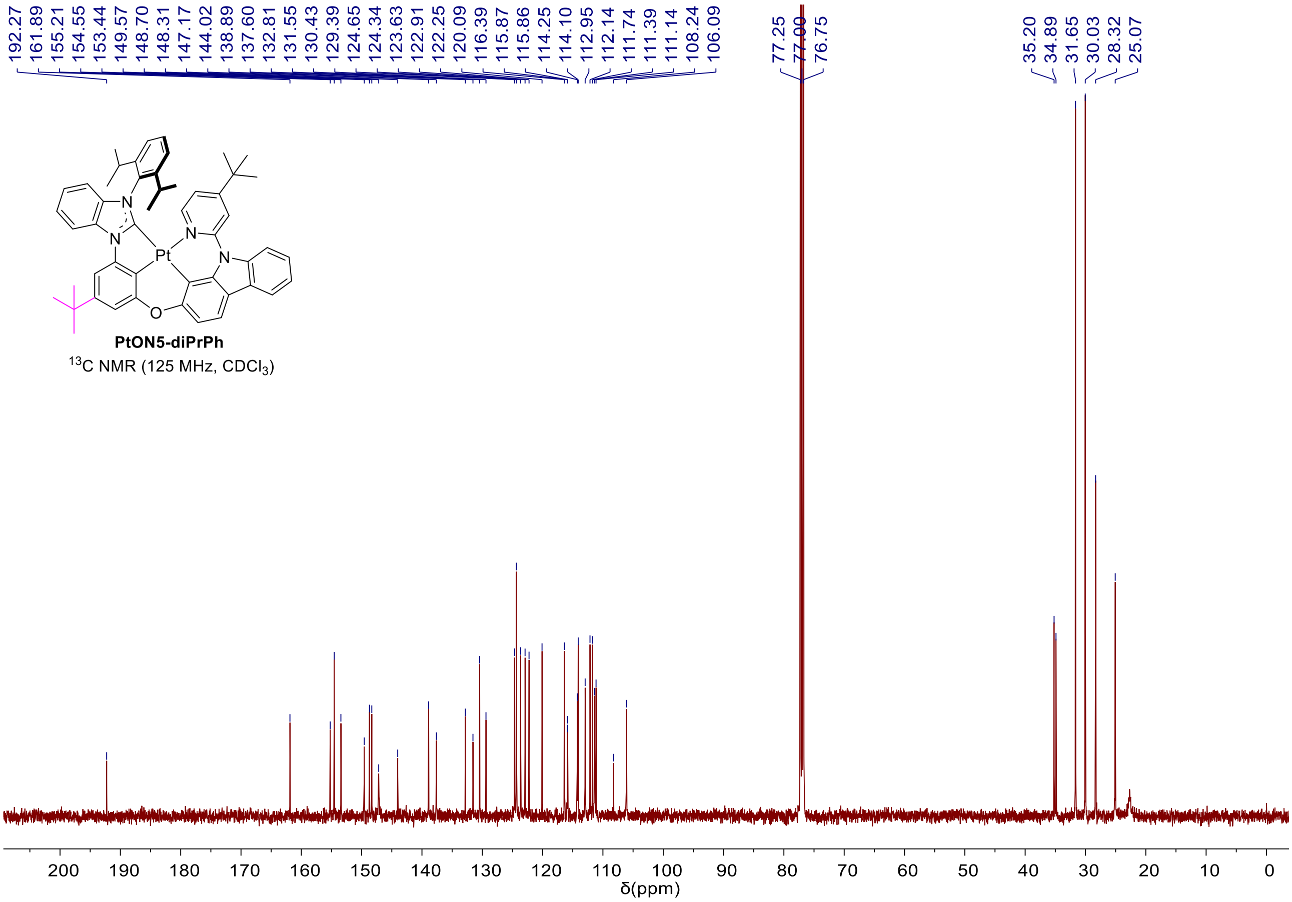
192.27 161.89 155.21 154.55 153.44 149.57 148.70 148.31 147.17 144.02 138.89 137.60 132.81 131.55 130.43 129.39 124.65 124.34 123.63 122.91 122.25 120.09 116.39 115.87 115.86 114.25 114.10 112.95 112.14 111.74 111.39 111.14 108.24 106.09 77.25 77.00 76.75 35.20 34.89 31.65 30.03 28.32 25.07
PtON5-diPrPh
^{13}C NMR (125 MHz, CDCl$_3$)
δ(ppm)

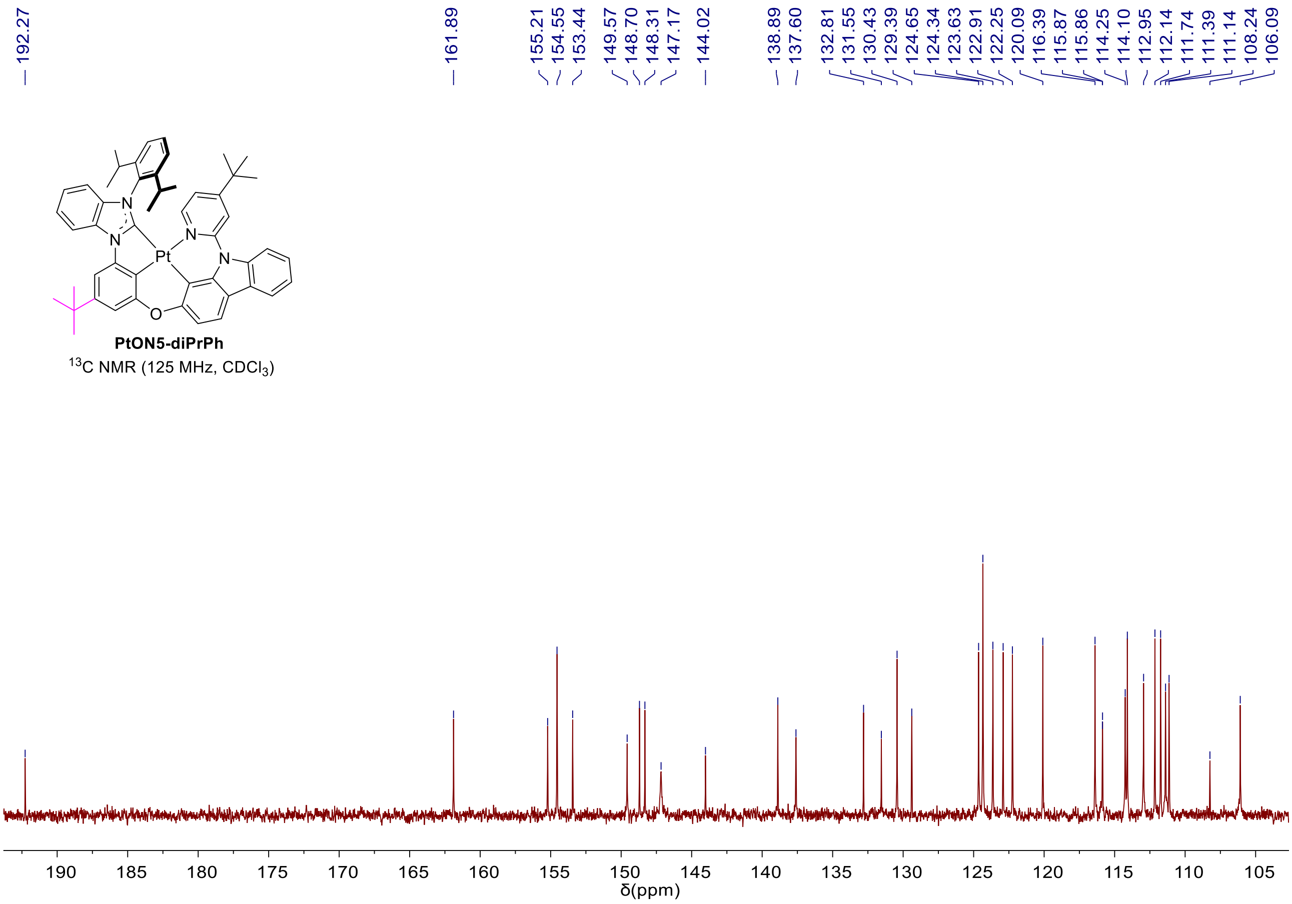
192.27
161.89
155.21
154.55
153.44
149.57
148.70
148.31
147.17
144.02
138.89
137.60
132.81
131.55
130.43
129.39
124.65
124.34
123.63
122.91
122.25
120.09
116.39
115.87
115.86
114.25
114.10
112.95
112.14
111.74
111.39
111.14
108.24
106.09
PtON5-diPrPh
^{13}C NMR (125 MHz, $CDCl_3$)
δ(ppm)

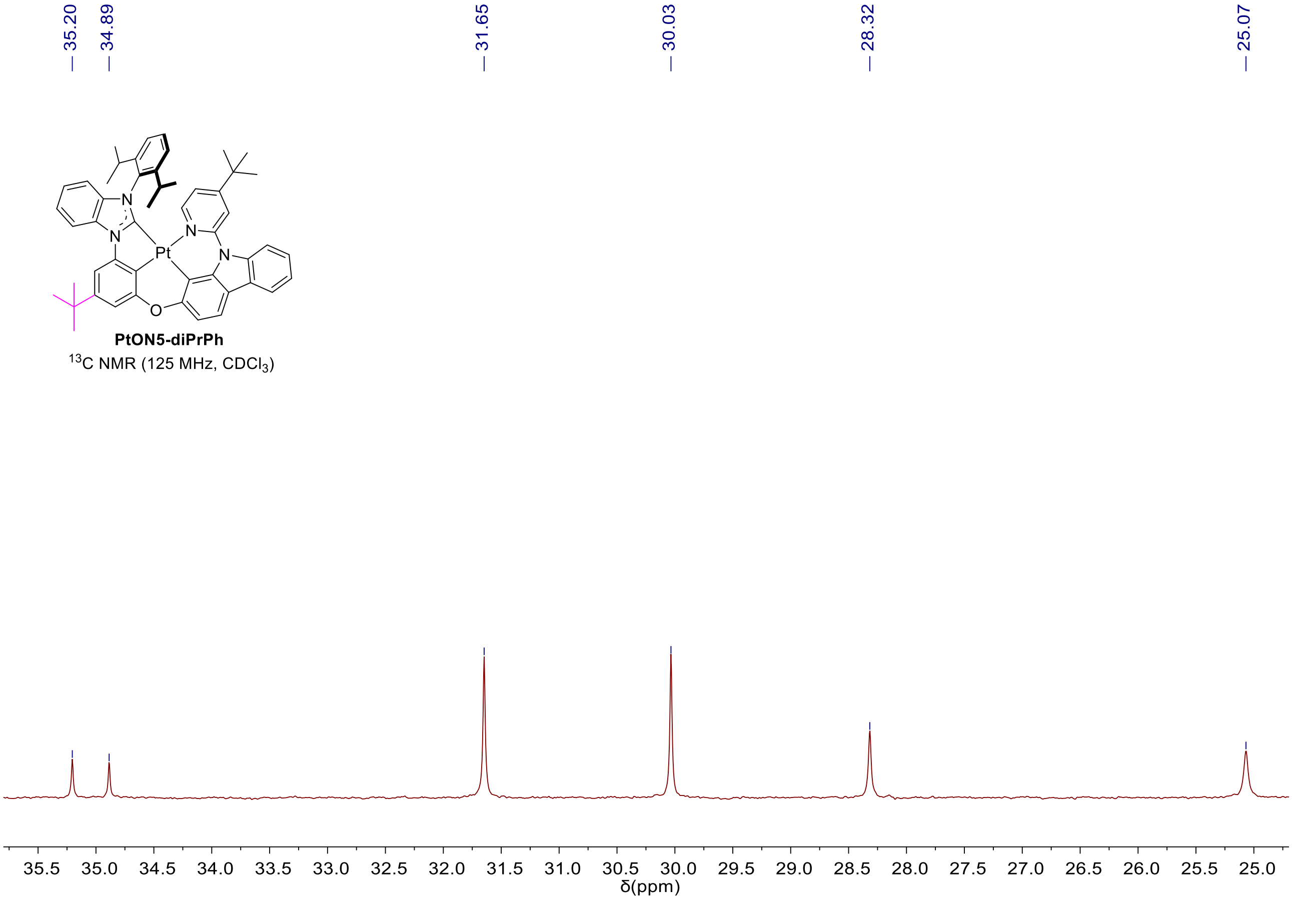
35.20
34.89
31.65
30.03
28.32
25.07
PtON5-diPrPh
^{13}C NMR (125 MHz, $CDCl_3$)
δ(ppm)

**Table S1. DFT Calculations for Pt(II) Complexes** [a]			
Complexes	Front view	Side view	Top view
PtCY	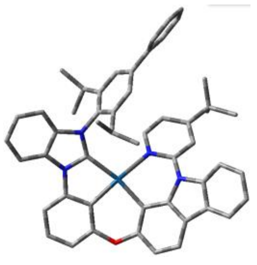	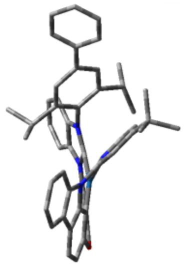	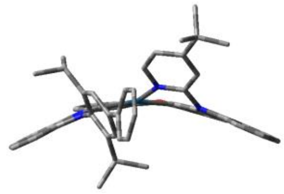
PtCY-*t*Bu	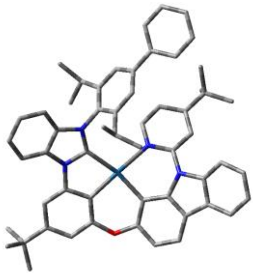	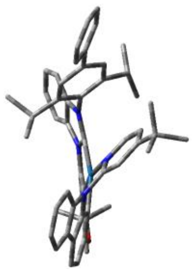	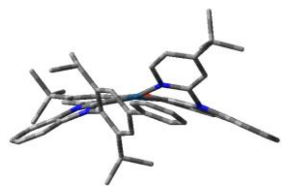
PtCY-F	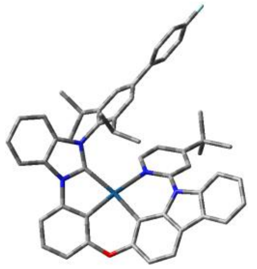	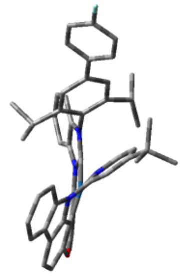	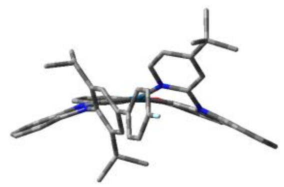
PtCY-*t*BuF	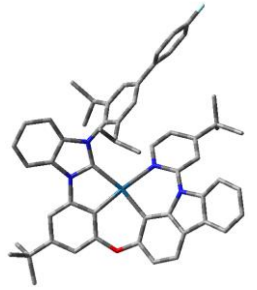	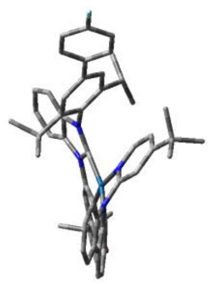	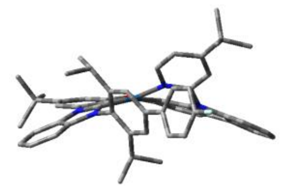

[a]Optimized S_0 were calculated using a B3LYP method with a basic set of 6-31G(d) for C, H, O and N atoms and a LANL2DZ basic set for Pt atoms.

Table S2. Selected Bond Lengths (Å), Bond Angles (°) and Dihedral (°) for Tetradentate Pt(II) Complexes Based on the X-ray Crystallographic Analysis and DFT Calculation Analysis.

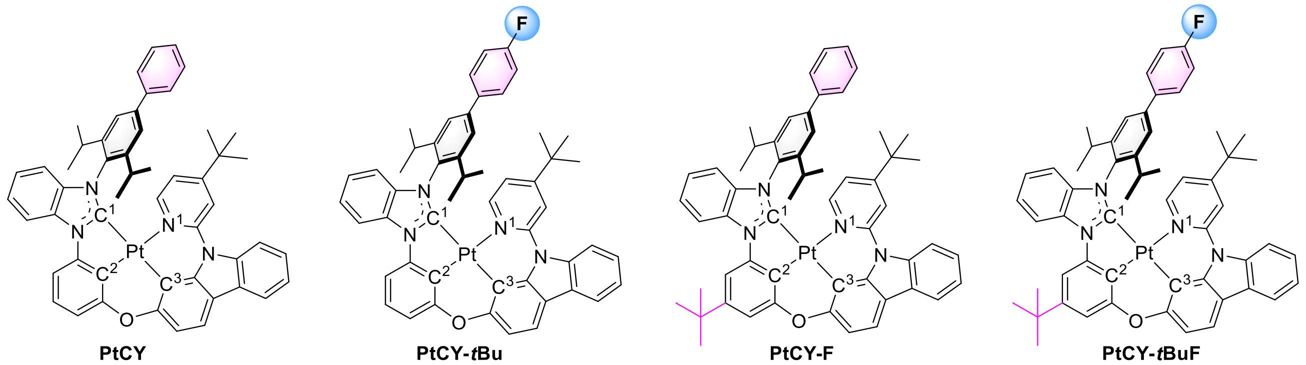

PtCY **PtCY-*t*Bu** **PtCY-F** **PtCY-*t*BuF**

metal complexes	Pt–C^1	Pt –C^2	Pt – C^3	Pt –N^1
PtCY_S0	2.082	1.990	2.028	2.205
PtCY-F_S0	2.081	1.991	2.029	2.206
PtCY-F(X-ray)	2.040	1.980	2.010	2.119
PtCY-*t*Bu_S0	2.083	1.988	2.029	2.204
PtCY-*t*BuF_S0	2.084	1.988	2.029	2.208
PtCY-*t*BuF(X-ray)	2.038	1.979	2.002	2.114

metal complexes	C^1– Pt –C^2	C^2– Pt – C^3	C^3–Pt–N^1	N^1–Pt–C^1	C^1–Pt–C^3	C^2–Pt–N^1	dihedral angle[a]
PtCY_S0	79.72	89.90	87.87	104.67	163.93	168.50	57.72
PtCY-F_S0	79.72	89.93	87.81	104.72	163.99	168.39	81.65
PtCY-F(X-ray)	79.76	89.09	88.61	104.10	162.36	172.46	56.92
PtCY-*t*Bu_S0	79.58	89.72	87.76	104.93	164.04	168.95	82.14
PtCY-*t*BuF_S0	79.60	89.56	87.89	105.01	163.69	168.79	81.79
PtCY-*t*BuF(X-ray)	79.55	89.48	89.00	103.33	162.23	173.82	56.85

[a]Dihedral angle between terminal pyridine and carbazole planes. Optimized S_0 were calculated using a B3LYP method with a basic set of 6-31G(d) for C, H, O and N atoms and a LANL2DZ basic set for Pt atoms.

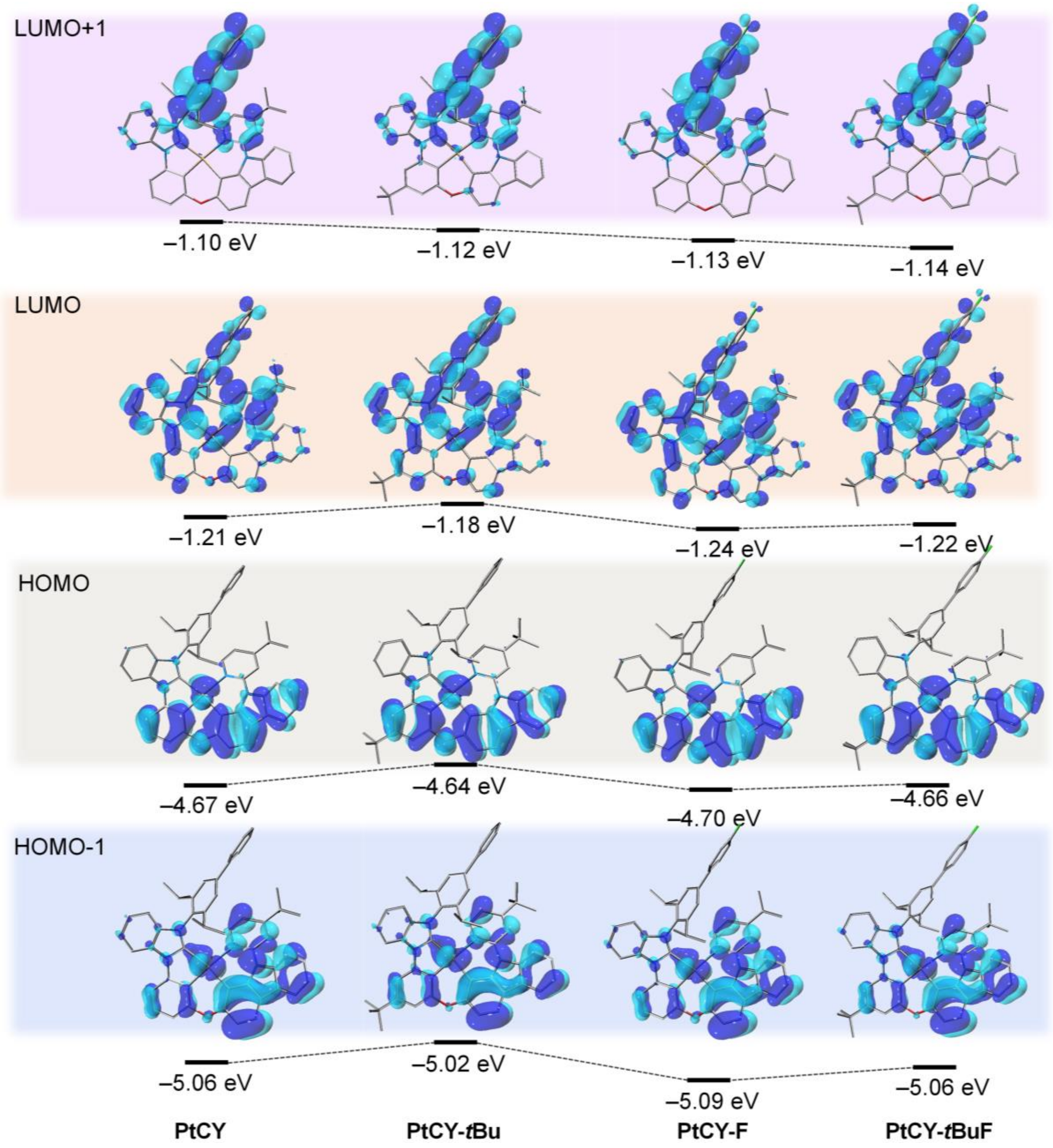

Figure S1. Density functional theory (DFT) calculated frontier orbits of Pt(II) complexes. Frontier orbits and energy levels of PtCY, PtCY-*t*Bu, PtCY-F and PtCY- *t*BuF. Optimized S_0 were calculated using a B3LYP method with a basic set of 6-31G* for C, H, O, and N atoms and a LANL2DZ basic set for Pt atom.

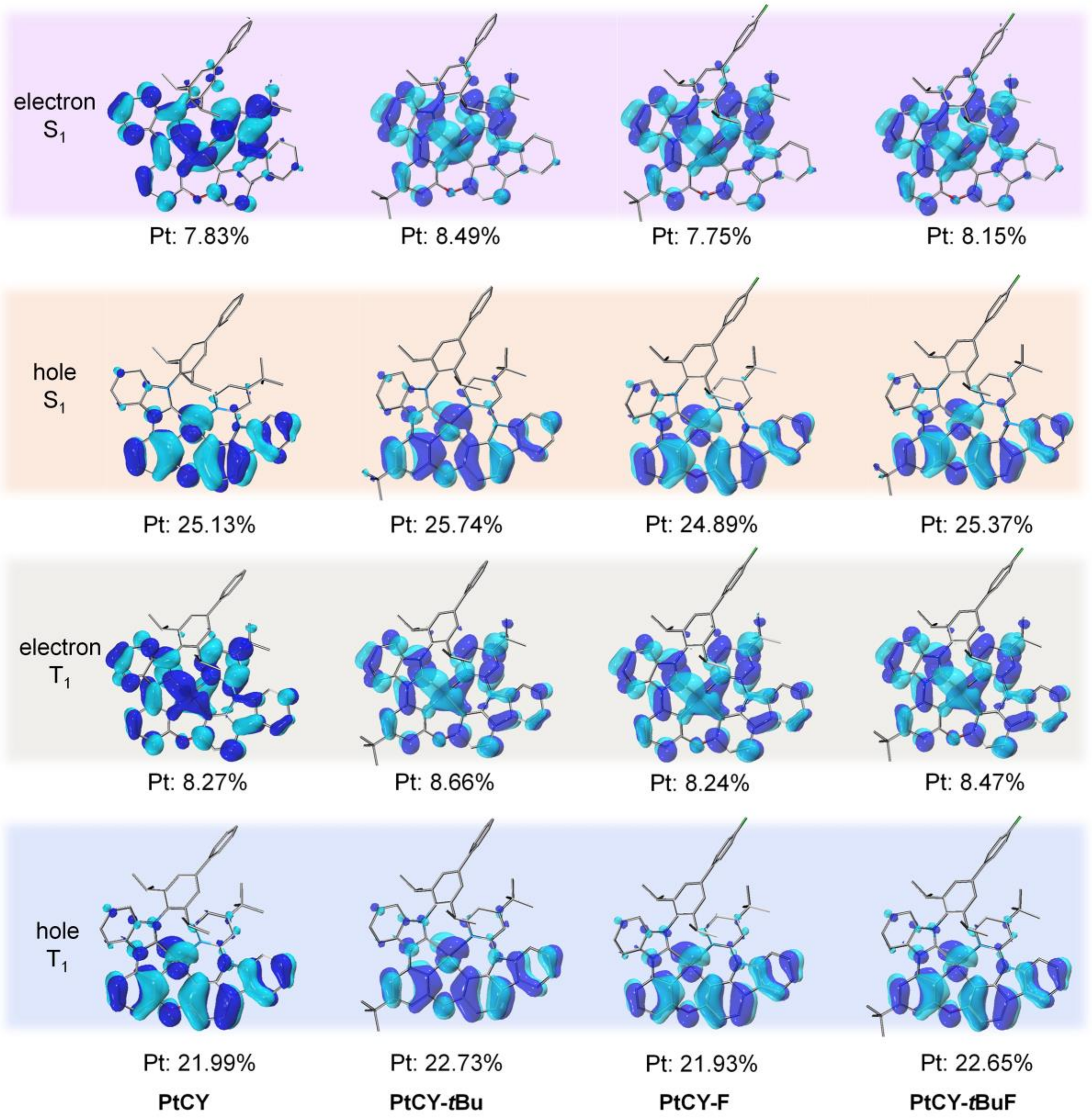

Figure S2. Time-dependent density functional theory (TD-DFT) calculations of Pt(II) complexes. Natural transition orbital (NTO) analyses of PtCY, PtCY-*t*Bu, PtCY-F and PtCY-*t*BuF. Optimized S_0 were calculated using a B3LYP method with a basic set of 6-31G* for C, H, O, and N atoms and a LANL2DZ basic set for Pt atom.

Table S3. Crystal data and structure refinements of PtCY-F

Compound	PtCY-F
Empirical formula	$C_{52}H_{45}FN_4OPt$
Formula weight	956.01
Temperature/K	170.00
Crystal system	monoclinic
Space group	$P2_1/n$
a/Å	16.9383(7)
b/Å	11.3880(5)
c/Å	22.5808(8)
α/°	90
β/°	109.1660(10)
γ/°	90
Volume/Å^3	4114.3(3)
Z	4
ρ_{calc}g/cm^3	1.543
μ/mm^{-1}	3.460
F(000)	1920.0
Crystal size/mm^3	0.13 × 0.09 × 0.06
Radiation	MoKα (λ = 0.71073)
2Θ range for data collection/°	4.054 to 56.654
Index ranges	$-22 \leq h \leq 22$, $-15 \leq k \leq 15$, $-30 \leq l \leq 30$
Reflections collected	91033
Independent reflections	10236 [R_{int} = 0.1001, R_{sigma} = 0.0517]
Data/restraints/parameters	10236/38/560
Goodness-of-fit on F^2	1.161
Final R indexes [I>=2σ (I)]	R_1 = 0.0419, wR_2 = 0.0591
Final R indexes [all data]	R_1 = 0.0678, wR_2 = 0.0673
Largest diff. peak/hole / e Å^{-3}	1.35/-0.89

Table S4. Crystal data and structure refinements of PtCY-*t*BuF

**Compound**	**PtCY-*t*BuF**
Empirical formula	$C_{56}FN_4OPtH_{53}$
Formula weight	1012.11
Temperature/K	170.00
Crystal system	triclinic
Space group	P-1
a/Å	13.0813(8)
b/Å	15.0171(9)
c/Å	25.2575(15)
α/°	94.028(2)
β/°	102.219(2)
γ/°	107.668(2)
Volume/Å^3	4572.1(5)
Z	4
ρ_{calc}g/cm^3	1.470
μ/mm^{-1}	4.190
F(000)	2048.0
Crystal size/mm^3	0.07 × 0.04 × 0.04
Radiation	GaKα (λ = 1.34139)
2Θ range for data collection/°	6.37 to 107.81
Index ranges	-15 ≤ h ≤ 15, -18 ≤ k ≤ 18, -30 ≤ l ≤ 30
Reflections collected	56431
Independent reflections	16524 [R_{int} = 0.0384, R_{sigma} = 0.0382]
Data/restraints/parameters	16524/0/1155
Goodness-of-fit on F^2	1.051
Final R indexes [I>=2σ (I)]	R_1 = 0.0388, wR_2 = 0.0869
Final R indexes [all data]	R_1 = 0.0494, wR_2 = 0.0918
Largest diff. peak/hole / e Å^{-3}	3.11/-1.83

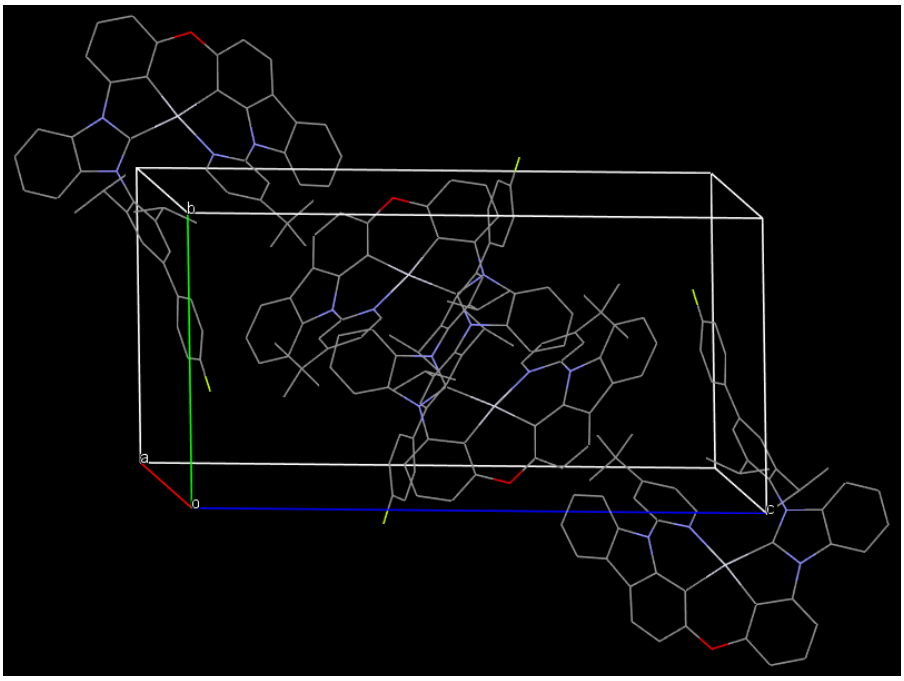

Figure S3. The molecular packing structure of PtCY-F. Solvent molecules and hydrogen atoms were omitted for clarity.

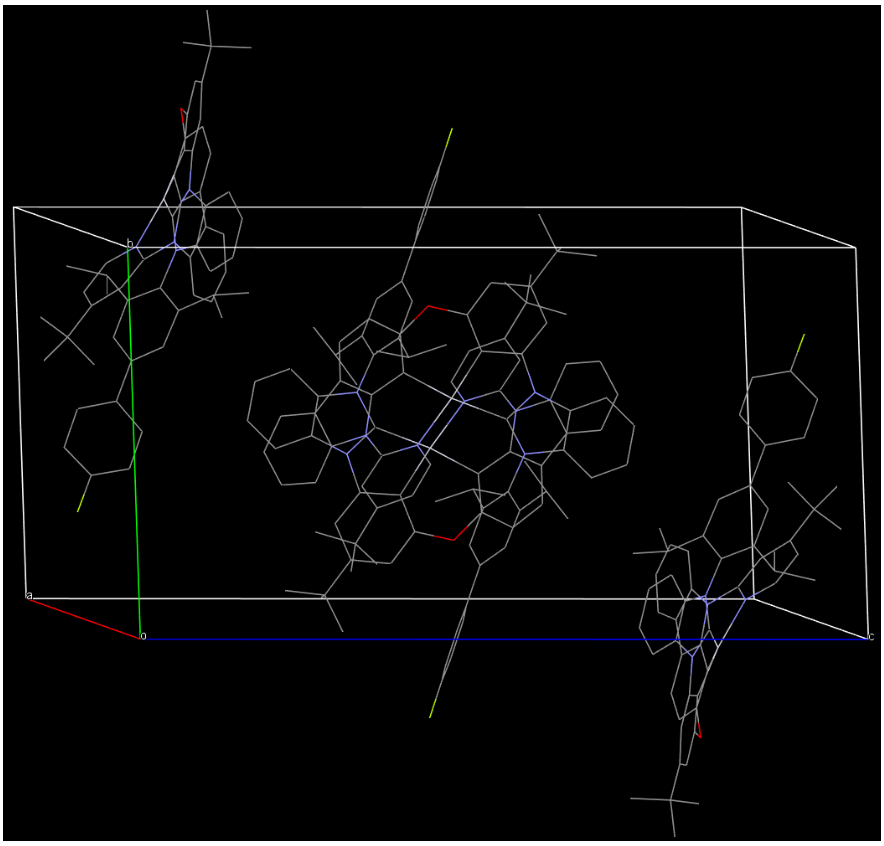

Figure S4. The molecular packing structure of PtCY-*t*BuF. Solvent molecules and hydrogen atoms were omitted for clarity.

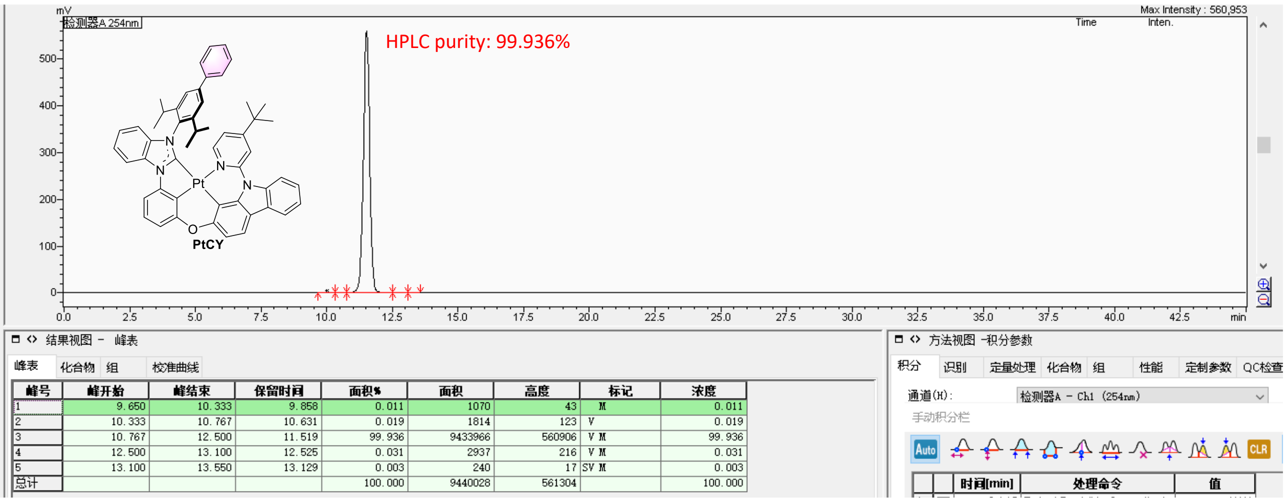

峰号	峰开始	峰结束	保留时间	面积%	面积	高度	标记	浓度
1	9.650	10.333	9.858	0.011	1070	43	M	0.011
2	10.333	10.767	10.631	0.019	1814	123	V	0.019
3	10.767	12.500	11.519	99.936	9433966	560906	V M	99.936
4	12.500	13.100	12.525	0.031	2937	216	V M	0.031
5	13.100	13.550	13.129	0.003	240	17	SV M	0.003
总计				100.000	9440028	561304		100.000

Figure S5. HPLC analysis of PtCY-sublimated sample. HPLC analysis condition: column: Masiall® C18-BlO, 5 µm, 250 × 4.6 mm; mobile phase: acetonitrile/isopropanol = 95/5(v/v); flow rate: 1.0 mL/min; Abs. detector: 254 nm

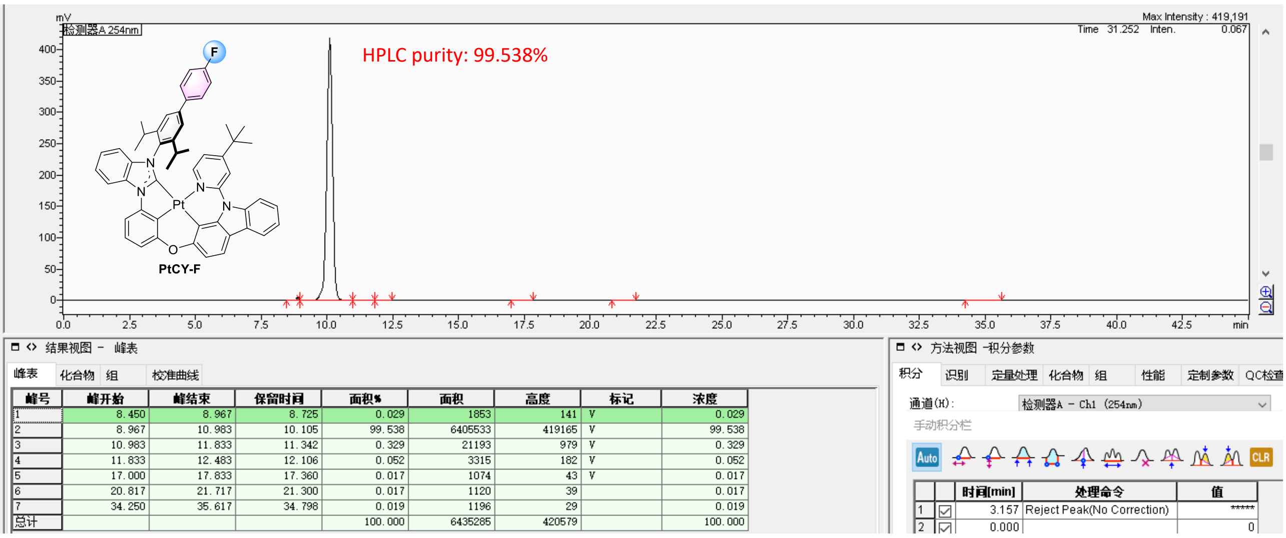

峰号	峰开始	峰结束	保留时间	面积%	面积	高度	标记	浓度
1	8.450	8.967	8.725	0.029	1853	141	V	0.029
2	8.967	10.983	10.105	99.538	6405533	419165	V	99.538
3	10.983	11.833	11.342	0.329	21193	979	V	0.329
4	11.833	12.483	12.106	0.052	3315	182	V	0.052
5	17.000	17.833	17.360	0.017	1074	43	V	0.017
6	20.817	21.717	21.300	0.017	1120	39		0.017
7	34.250	35.617	34.798	0.019	1196	29		0.019
总计				100.000	6435285	420579		100.000

Figure S6. HPLC analysis of PtCY-F-sublimated sample. HPLC analysis condition: column: Masiall® C18-BlO, 5 µm, 250 × 4.6 mm; mobile phase: acetonitrile/isopropanol = 95/5(v/v); flow rate: 1.0 mL/min; Abs. detector: 254 nm

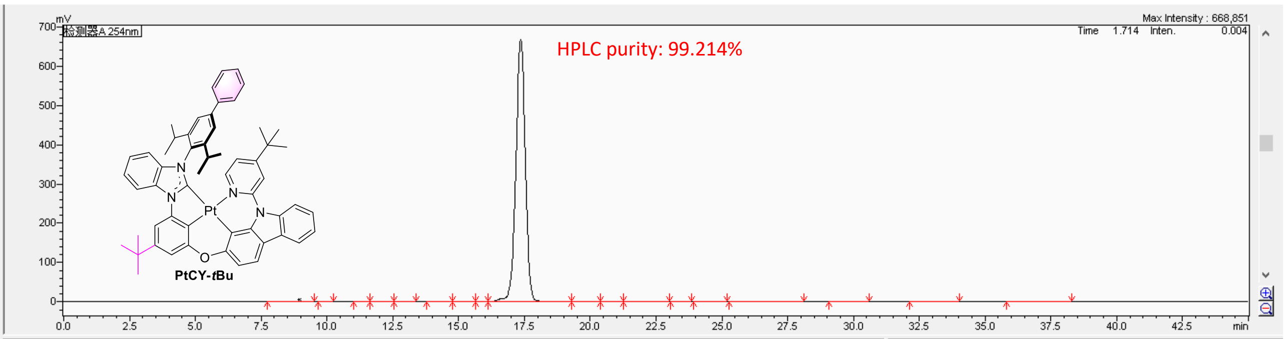

结果视图 - 峰表

峰号	峰开始	峰结束	保留时间	面积%	面积	高度	标记	浓度
1	7.717	9.517	8.773	0.011	1789	36	M	0.011
2	9.667	10.233	10.049	0.001	241	12	M	0.001
3	11.017	11.633	11.361	0.012	2032	125	M	0.012
4	11.633	12.533	11.887	0.011	1885	90	V M	0.011
5	12.533	13.400	12.906	0.007	1169	66	M	0.007
6	13.800	14.783	14.396	0.221	37198	1830	M	0.221
7	14.783	15.633	15.189	0.204	34263	1642	V M	0.204
8	15.633	16.117	15.940	0.033	5554	298	M	0.033
9	16.117	19.300	17.360	99.214	16673759	668731	V M	99.214
10	19.300	20.383	20.107	0.027	4556	138	M	0.027

检测器A - Ch1 (254nm)

方法视图 -积分参数

通道(H): 检测器A - Ch1 (254nm)

	时间[min]	处理命令	值
1	3.158	Reject Peak(No Correction)	*****
2	16.659	Split Peak	*****
3	16.658	Reject Peak(No Correction)	*****
4	17.360	Reject Peak(No Correction)	*****

Figure S7. HPLC analysis of PtCY-*t*Bu-sublimated sample. HPLC analysis condition: column: Masiall® C18-BlO, 5 μm, 250 × 4.6 mm; mobile phase: acetonitrile/isopropanol = 95/5(v/v); flow rate: 1.0 mL/min; Abs. detector: 254 nm

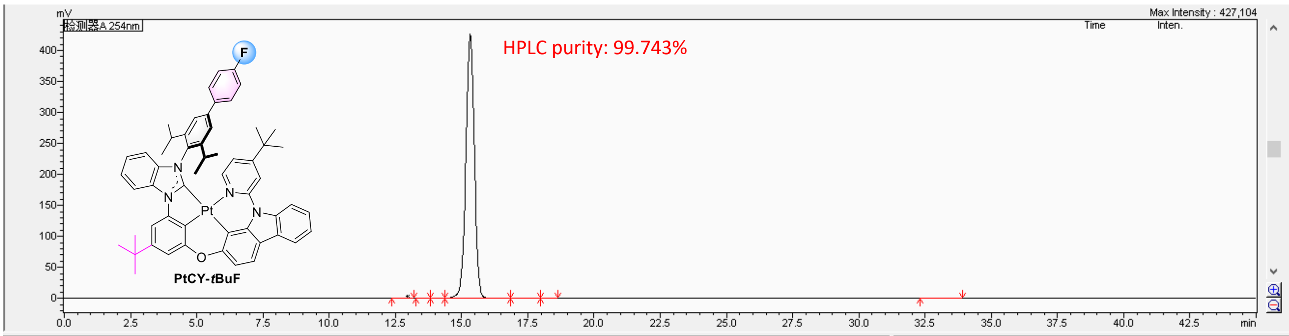

结果视图 - 峰表

峰号	峰开始	峰结束	保留时间	面积%	面积	高度	标记	浓度
1	12.350	13.217	12.784	0.029	2716	149		0.029
2	13.267	13.817	13.671	0.003	265	23	M	0.003
3	13.817	14.350	14.164	0.072	6876	348		0.072
4	14.350	16.850	15.326	99.743	9500720	427055	V	99.743
5	16.850	17.967	17.363	0.105	10039	358	V	0.105
6	17.967	18.650	18.197	0.012	1143	49	V	0.012
7	32.317	33.917	33.079	0.036	3404	77		0.036
总计				100.000	9525163	428060		100.000

方法视图 -积分参数

通道(H): 检测器A - Ch1 (254nm)

	时间[min]	处理命令	值
1	3.155	Reject Peak(No Correction)	*****
2	13.269	Insert Peak	13.846

Figure S8 HPLC analysis of PtCY-*t*BuF-sublimated sample. HPLC analysis condition: column: Masiall® C18-BlO, 5 μm, 250 × 4.6 mm; mobile phase: acetonitrile/isopropanol = 95/5(v/v); flow rate: 1.0 mL/min; Abs. detector: 254 nm

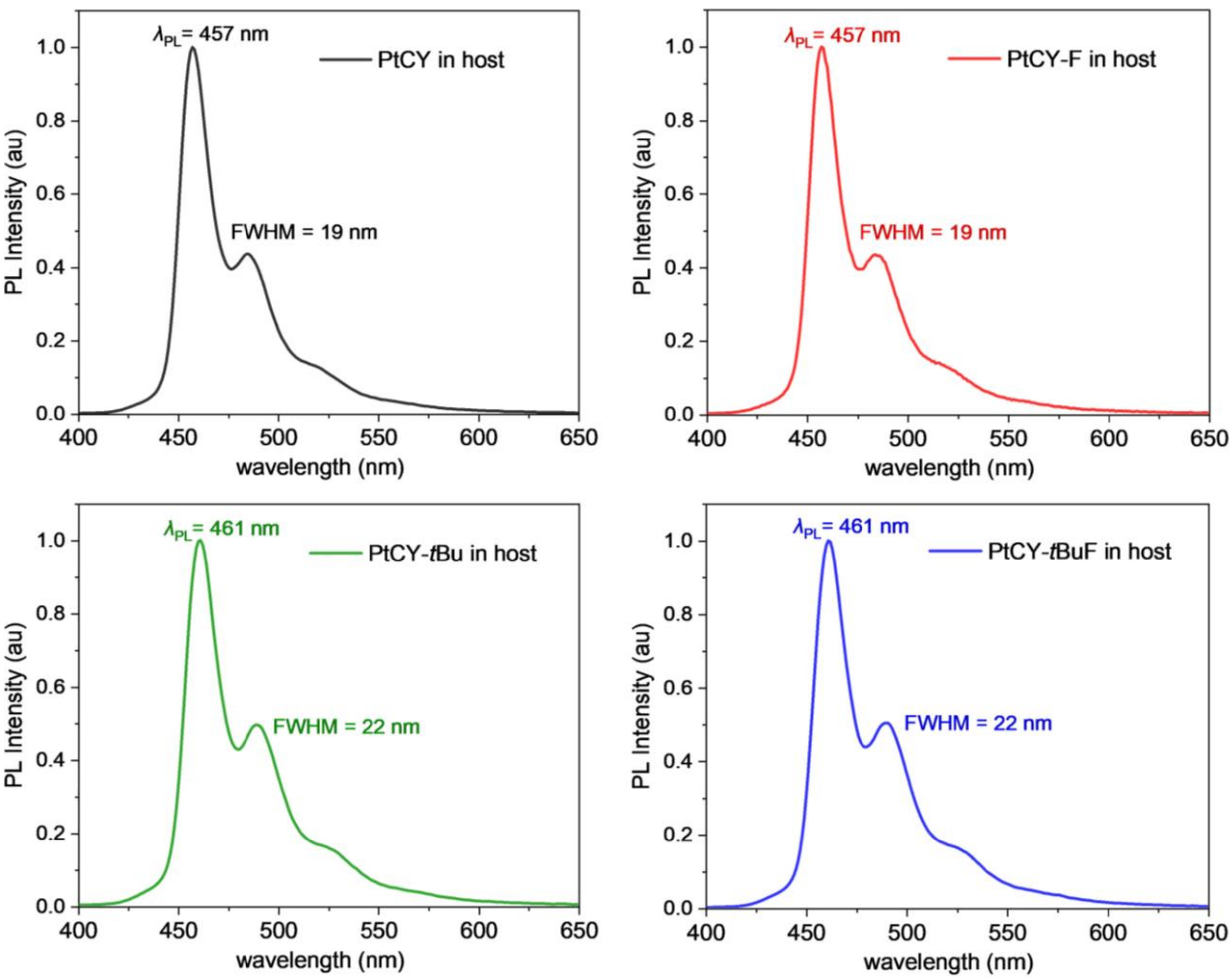

Figure S9. PL spectra of Pt(II) complexes at RT in host (65 wt.% SiCzCz:27 wt.% $SiTrzCz_2$) film.

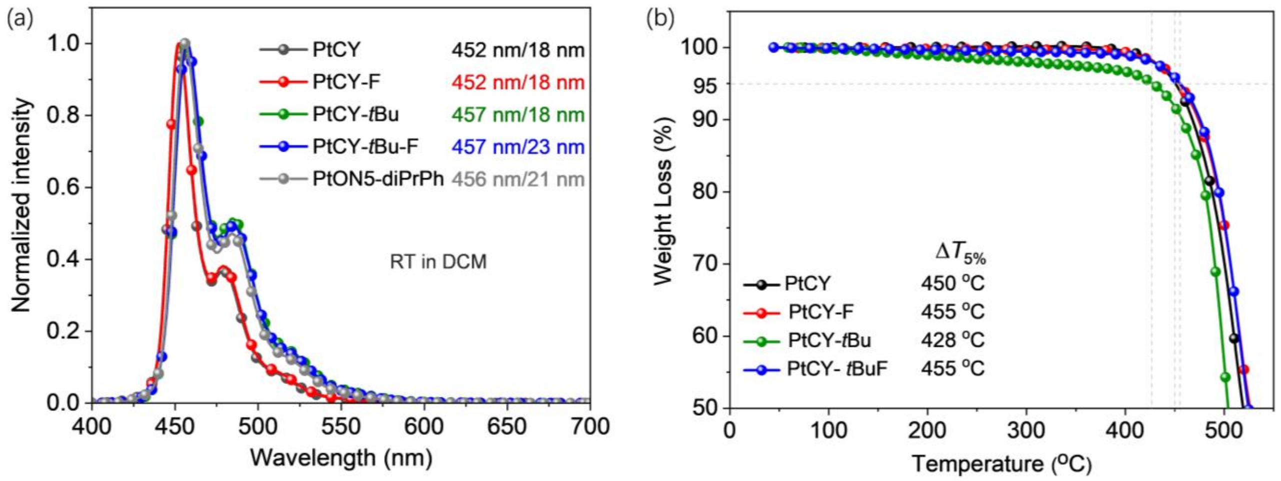

Figure S10. (a) Comparison of PL spectra in dichloromethane at room temperature for the Pt(II) complex. (b) TGA curves of Pt(II) complexes.

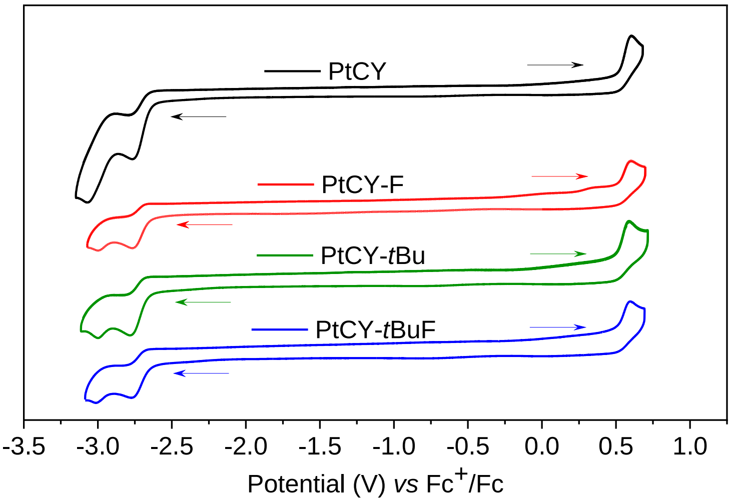

Figure S11. Electrochemical properties of Pt(II) complexes. Cyclic voltammograms.

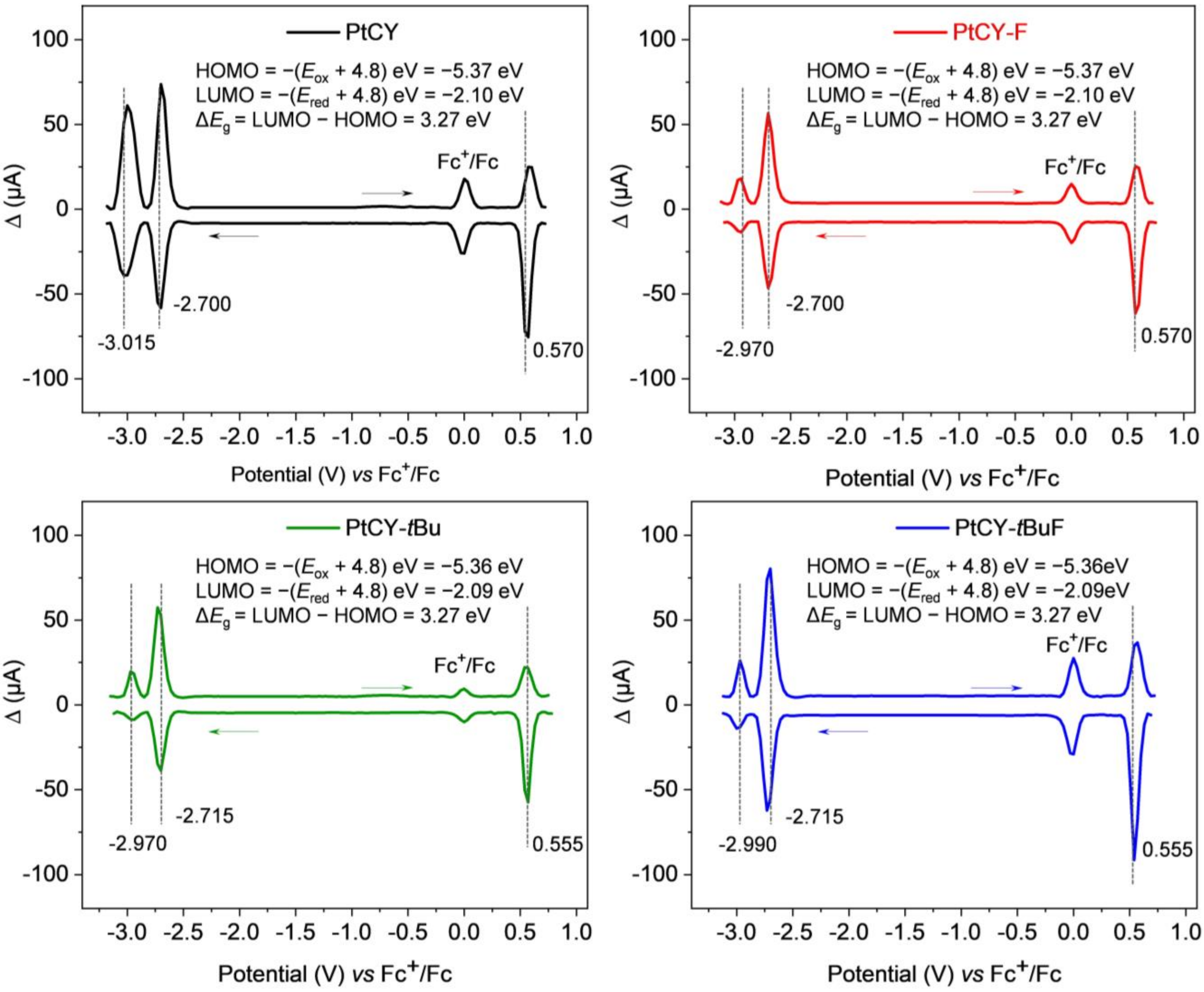

Figure S12. Electrochemical properties of Pt(II) complexes. differential pulse voltammetry (DPV) curves of tetradentate Pt(II) complexes measured in *N*, *N*-dimethylformamide under a nitrogen atmosphere.

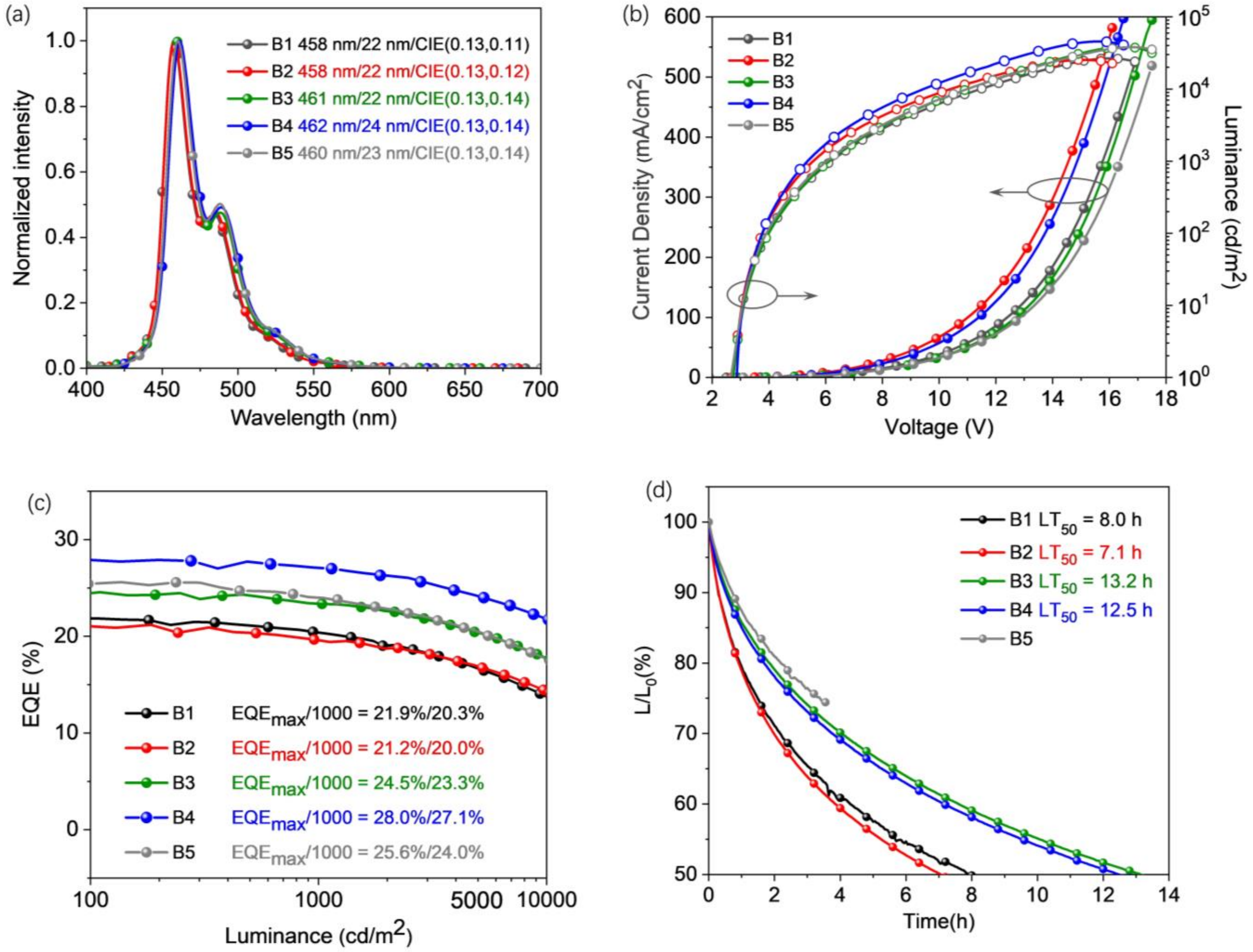

Figure S13. EL performances of PtON5-diPrPh (B5) and PtCY (B1–B4) a) Current density-voltage-luminance curve. b) EL spectra of deep-blue OLEDs at 1000 cd/m^2. c) External quantum efficiency (EQE) vs. luminance curves. d) The operational lifetimes of OLEDs based on Pt(II) emitters with a doping concentration of 8 wt.% at an L_0 of 1000 cd/m^2.

Table S5. **Summary of device performance for Pt(II)-based deep-blue OLEDs**

Device	Emitter	V_{on} (V)	λ_{EL} (nm)	FWHM (nm)	CIE (x, y)	EQE [a] (%)	L_{max} (cd/m^2)	LT$_{50}$ (h)
B1	PtCY	2.5	458	22	(0.13, 0.11)	21.9/20.3/16.7/13.5	26594	8.0
B2	PtCY-F	2.9	458	22	(0.13, 0.12)	21.2/20.0/16.8/14.2	25423	7.1
B3	PtCY-*t*Bu	2.5	461	22	(0.13, 0.14)	24.5/23.3/20.4/17.6	38306	13.2
B4	PtCY-*t*BuF	2.7	462	24	(0.13, 0.14)	28.0/27.1/24.3/21.7	45621	12.5
B5	PtON5-diPrPh	2.7	460	23	(0.13, 0.14)	25.6/24.0/20.4/17.4	38079	/

[a] Maximum EQE, along with EQEs at 1000, 5000, and 10000 cd/m^2.

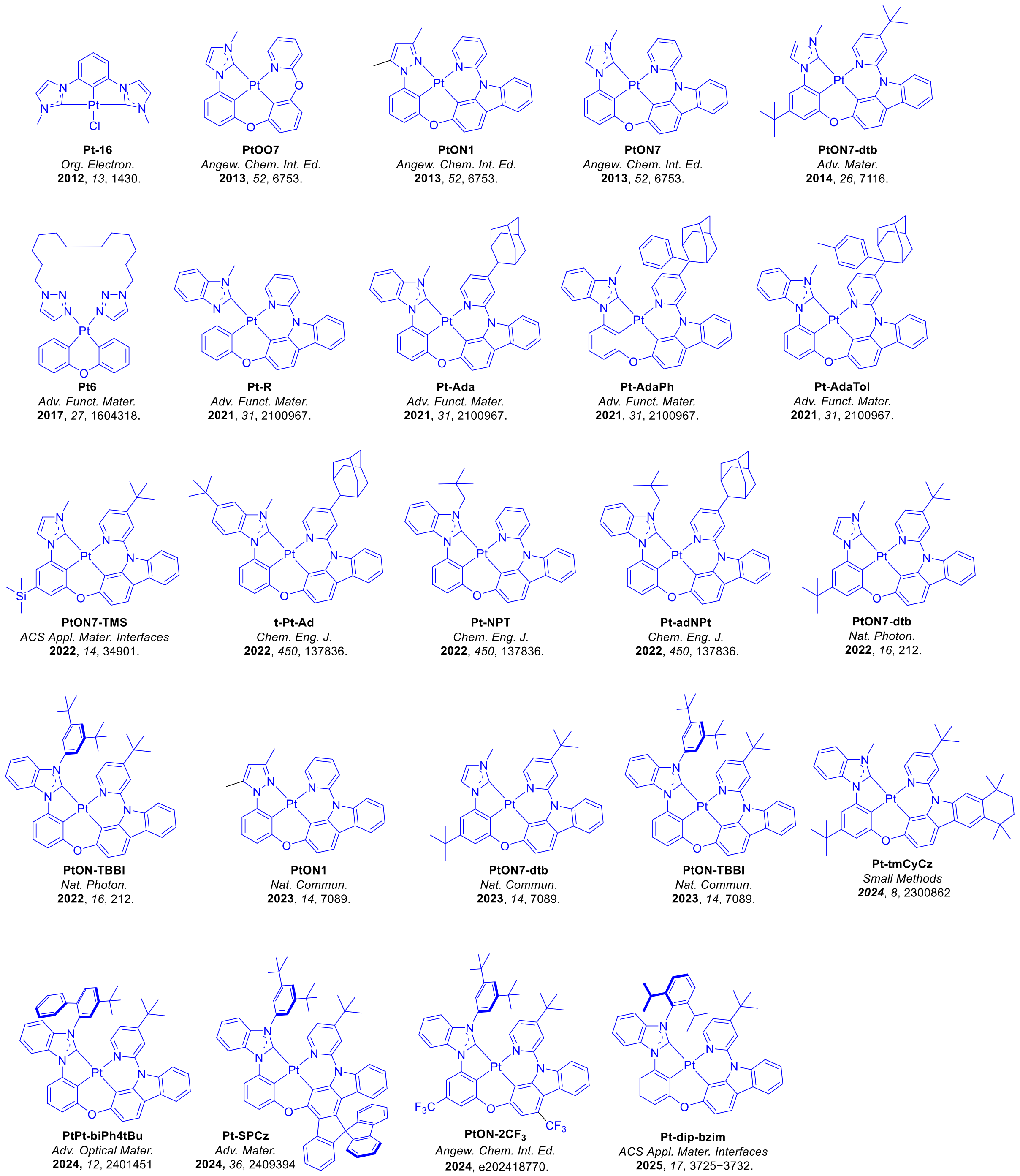

Figure S14. Pt(II) complexes-based deep-blue emitters. Chemical structures of dopants for Pt(II)-based deep-blue phosphorescent OLEDs discussed in this work.

Table S6. Device performance data for Pt(II)-based single-layer deep-blue phosphorescent OLEDs with CIE_y < 0.15

emitter/host	CIE (x, y)	λ_{EL} (nm)/ FWHM (nm)	L_{max} (cd/m^2)	EQE (%) [a] Max/1000/5000/10000	Efficiency roll-off rate (%) [b]	reference/year
**PtCY** /SiCzCz:SiTrzCz2 [b]	(0.13, **0.11**)	**458/22**	**26594**	**21.9/20.3/16.7/13.5**	**7.3**	**This work**
**PtCY-F**/SiCzCz:SiTrzCz2 [b]	(0.13, **0.12**)	**458/22**	**25423**	**21.2/20.0/16.8/14.2**	**5.7**	**This work**
**PtCY-*t*Bu**/SiCzCz:SiTrzCz2 [b]	(0.13, **0.14**)	**461/22**	**38306**	**24.5/23.3/20.4/17.6**	**4.9**	**This work**
**PtCY-*t*BuF**/SiCzCz:SiTrzCz2 [b]	(0.13, **0.14**)	**462/24**	**45621**	**28.0/27.1/24.3/21.7**	**3.2**	**This work**
**PtON5-diPrPh**/SiCzCz:SiTrzCz2 [b]	(0.13, **0.14**)	**460/23**	**38079**	**25.6/24.0/20.4/17.4**	**6.3**	**This work**
Pt-16/26mCPy	(0.16, 0.13)	450/~45	----	15.7/<5/----/----	68.2	[1]/2012
PtOO7/26mCPy	(0.15, 0.10)	448/~50	----	<9/0.5/----/----	94.4	[2]/2013
PtON1/26mCPy	(0.15, 0.13)	454/~49	----	25.2/16.8/----/----	33.3	[2]/2013
PtON7/26mCPy	(0.15, 0.14)	456/~51	----	23.7/15.4/----/----	35.0	[2]/2013
PtON7-dtb/TAPC:PO15	(0.148, 0.079)	452/29	1555	24.8/11.0/----	55.6	[3]/2014
Pt6/BCPO	(0.14, 0.14)	452/---	10676	9.7/7.6/5.5/ <4	21.6	[4]/2017
Pt-R/mCP	(0.138, 0.122)	~453/~37	1645	22.0/17.0/----/----	22.7	[5]/2021
Pt-Ada/mCP	(0.145, 0.110)	~451/~25	1078	21.2/14.5/----/----	31.6	[5]/2021
Pt-AdaPh/mCP	(0.137, 0.122)	~453/~37	4528	23.5/16.5/----/----	29.8	[5]/2021
Pt-AdaTol/mCP	(0.140, 0.120)	~453/~27.5	1593	22.0/15.5/----/----	29.5	[5]/2021
PtON7-TMS/mCBP	(0.142, 0.099)	452/30	2722	15.6/10.7/----/----	31.4	[6]/2022
t-Pt-Ad/mCP	(0.141, 0.092)	~453/~24	3258	20.3/16.3/----/----	19.7	[7]/2022
Pt-NPT/mCP	(0.139, 0.118)	~451/~37.5	3389	19.8/16.8/----/----	15.2	[7]/2022
Pt-adNPT/mCP	(0.143, 0.090)	~453/~24	2512	15.7/11.2/----/----	28.7	[7]/2022
PtON7-tBu/oCBP:CNmCBP-CN	(0.142, 0.143)	~452/~45	----	14.7/11.8/----/----	19.7	[8]/2022
PtON-TBBI/oCBP:CNmCBP-CN	(0.132, 0.147)	~453/~37	----	26.4/23.7/----/----	10.2	[8]/2022
PtON1/BO1b/26mCPy	(0.138, 0.130)	454/50	12982	24.8/20.9/15.0/10.2	15.7	[9]/2023
PtON1/BO1b/mCBP	(0.138, 0.142)	456/51	15722	27.1/21.8/15.6/11.4	19.6	[9]/2023
PtON7-dtb/BO1b/mCBP	(0.138, 0.088)	453/28	5670	27.6/16.0/7.9/----	42.0	[9]/2023
PtON-TBBI/BO1b/mCBP	(0.134, 0.104)	458/21	9377	28.0/19.0/13.4/7.3	32.1	[9]/2023
PtON-TBBI/BO2/mCB	(0.135, 0.103)	458/21	14481	22.2/14.9/10.4/7.3	32.9	[9]/2023
PtON-TBBI/BO3a/mCBP	(0.134, 0.107)	459/22	15765	19.6/14.8/10.8/8.2	24.5	[9]/2023
PtON-TBBI/BO6/mCBP	(0.133, 0.111)	459/22	10071	21.7/13.4/8.7/4.0	38.2	[9]/2023
Pt-tmCyCz/oCBP:CNmCBP-CN	(0.132, 0.138)	462/24	30000	21.5/20.5/18/~16	4.7	[10]/2024
Pt-biPh4tBu/SiBCz:SiTrzCz2	(0.139, 0.149)	461/21	~45000	21.8/19.9/16.4/15	8.7	[11]/2024
Pt-SPCz/SiBCz:SiTrzCz2	(0.141, 0.131)	461/22	----	25.1/21.1/18/15	15.9	[12]/2024
Pt-2CF3/SiBCz:SiTrzCz2	(0.140, 0.140)	461/18	48726	27.6/26.7/----/20.5	3.3	[13]/2024
Pt-dip-bzim/3-CzPB	(0.139, 0.090)	455/17	----	24.1/19.6/~12/----	18.7	[14]/2025

[a] EQE @ Max/1000/5000/10000 cd m^{-2}. [b] Efficiency roll-off rate @1000cd m^{-2}. [c] Device structure: ITO/HATCN (20 nm)/TAPC (60 nm)/SiCzCz (5 nm)/ emitter:SiCzCz:SiTrzCz2 (8:65:27, 35 nm)/mSiTrz (5 nm)/mSiTrz:Liq (50:50, 31 nm)/LiF (1.5 nm)/Al.

Supplementary References:

[1] T. Fleetham, Z. Wang, J. Li, *Org. Electron.* **2012,** *13,* 1430−1435.

[2] X.-C. Hang, T. Fleetham, E. Turner, J. Brooks, J. Li, *Angew. Chem. Int. Ed.* **2013,** *52,* 6753−6756.

[3] T. Fleetham, G. Li, L. Wen, J. Li, *Adv. Mater.* **2014,** *26,* 7116−7121.

[4] X. Wang, T. Peng, C. Nguyen, Z.-H. Lu, N. Wang, W. Wu, Q. Li, S. Wang, *Adv. Funct. Mater.* **2017,** *27,* 1604318.

[5] J.-S. Huh, M. J. Sung, S.-K. Kwon, Y.-H. Kim, J.-J. Kim, *Adv. Funct. Mater.* **2021,** *31,* 2100967.

[6] H. J. Park, J.-H. Jang, J.-H. Lee, D.-H. Hwang, *ACS Appl. Mater. Interfaces.* **2022,** *14,* 34901–34908.

[7] J.-S. Huh, D. Y. Lee, K. H. Park, S.-K. Kwon, Y.-H. Kim, J.-J. Kim, *Chem. Eng. J.* **2022,** *450,* 137836.

[8] J. Sun, H. Ahn, S. Kang, S.-B. Ko, D. Song, H. A. Um, S. Kim, Y. Lee, P. Jeon, S.-H. Hwang, Y. You, C. Chu, S. Kim, *Nat. Photon.* **2022,** *16,* 212−218.

[9] G. Li, K. Xu, J. Zheng, X. Fang, Y.-F. Yang, W. Lou, Q. Chu, J. Dai, Q. Chen, Y. Yang, Y.-B. She, *Nat. Commun.* **2023,** *14,* 7089.

[10] K. Cheong, U. Jo, W. P. Hong, J. Y. Lee, *Small Methods*. **2024,** *8,* 2300862.

[11] J. Choi, K. Cheong, S. Han, J. Y. Lee, *Adv. Opt. Mater*. **2024,** *12,* 2401451.

[12] H. Lee, B. Park, G. R. Han, M. S. Mun, S. Kang, W. P. Hong, H. Y. Oh, T. Kim, *Adv. Mater.* **2024,** *36,* 2409394.

[13] J. Zhu, M. Huang, Y. Zhang, Z. Chen, Y. Deng, H. Zhang, X. Wang, C. Yang, *Angew. Chem. Int. Ed.* **2024,** *64,* e202418770.

[14] K. Cheong, S. Han, J. Y. Lee, *ACS Appl. Mater. Interfaces* **2025,** *17,* 3725−3732.

Cartesian coordinates of the optimized structures

PtCY_S$_0$

C	4.88090300	0.25086600	0.68095000
C	4.77503400	3.04782900	0.73523100
C	6.04218400	0.99862000	0.94224300
C	3.64943700	0.86856100	0.45587800
C	3.65055700	2.27753100	0.45212900
C	5.98278600	2.38464300	0.98252400
H	6.97281500	0.46701800	1.11506200
C	-0.08959700	5.08136600	-1.11320000
C	2.71652400	5.25154600	-0.63120300
C	0.62441600	4.01399300	-0.57120000
C	0.62153500	6.24617700	-1.40154800
C	2.00310900	6.32420100	-1.16660100
C	2.00509000	4.08790000	-0.32123000
H	0.10001000	7.09846300	-1.82673600
H	2.53630400	7.23646200	-1.41746800
H	3.78716900	5.32066900	-0.49531600
N	0.21975400	2.71773800	-0.22408700
C	1.29122900	1.97076900	0.19742200
N	2.36455000	2.83365800	0.17571200
Pt	1.87082700	-0.02272000	0.39801900
O	5.08615200	-1.10188300	0.67450800
C	4.17059700	-2.03283100	0.23216100
C	2.65613800	-4.12377700	-0.76928400
C	2.78261200	-1.82057400	0.18094200
C	4.79202300	-3.23374800	-0.16855200
C	4.04470900	-4.27633200	-0.69127400
C	2.07171400	-2.93129800	-0.29229000
H	5.87285600	-3.29917000	-0.09464900
H	4.52948600	-5.18424900	-1.03928600
N	0.65152600	-3.04522600	-0.42967200
C	0.38069500	-4.26450100	-1.09740900
C	1.59854000	-4.95501000	-1.30311100
C	-0.80623500	-5.98204200	-2.27560000
C	1.59458900	-6.18138700	-1.97619100
C	-0.82399600	-4.75733300	-1.60437200
C	0.38876100	-6.69481300	-2.45031700
H	-1.75036000	-4.20138000	-1.50663400
H	-1.73477500	-6.37868700	-2.67679200
H	2.52472300	-6.71913100	-2.13943300
H	0.37609800	-7.64572100	-2.97579700
H	4.72488400	4.12658600	0.80997700
H	-1.15126300	4.99777000	-1.31858000

C	-0.25125900	-2.39291200	0.41200600
C	-1.98754500	-1.13328000	2.13127400
C	-1.34955400	-3.09431600	0.95345800
N	-0.01169800	-1.10468500	0.74145700
C	-0.87830500	-0.51396100	1.59539400
C	-2.24334800	-2.48319200	1.82268800
H	-1.44788900	-4.14125000	0.71138300
H	-0.63696300	0.50892500	1.84453000
H	-2.62859900	-0.56891300	2.79862700
C	-3.42576200	-3.22444100	2.46216200
C	-3.55040500	-4.67520700	1.96049700
H	-4.41649300	-5.15271000	2.43176100
H	-2.66791900	-5.27369000	2.21238700
H	-3.69736000	-4.72087900	0.87542300
C	-4.73737800	-2.47614800	2.12474500
H	-5.59192800	-2.99736600	2.57215000
H	-4.89893400	-2.42358500	1.04251600
H	-4.73392400	-1.45192400	2.51188600
C	-3.22915400	-3.25069400	3.99741800
H	-4.07003500	-3.76658700	4.47603000
H	-3.17197400	-2.24133600	4.41824000
H	-2.30783300	-3.77890400	4.26715900
H	6.87721600	2.95976900	1.20404500
C	-1.14950500	2.28889400	-0.35447300
C	-3.84428600	1.57710400	-0.66417500
C	-1.54285900	1.58629900	-1.51272900
C	-2.07756700	2.66201000	0.64347900
C	-3.41361800	2.29360900	0.46040900
C	-2.89146800	1.23586400	-1.63245700
H	-4.14823300	2.60016300	1.19914300
H	-3.20861000	0.66953900	-2.50241100
C	-5.27374100	1.20613500	-0.83217800
C	-7.98858200	0.50631700	-1.14951200
C	-5.88058700	1.22082800	-2.09997100
C	-6.05902400	0.83486400	0.27284200
C	-7.40088700	0.48882500	0.11696000
C	-7.22200900	0.87413400	-2.25714300
H	-5.30283300	1.53189500	-2.96569000
H	-5.60631200	0.79423800	1.25942500
H	-7.98569800	0.19789800	0.98561900
H	-7.67130200	0.90050900	-3.24636100
H	-9.03398800	0.23676600	-1.27209700
C	-0.57371200	1.26014500	-2.64732100
H	0.44293000	1.43958700	-2.28898500

C	-0.81902500	2.19956100	-3.84745100
H	-0.09462600	1.99357600	-4.64399300
H	-0.71668400	3.25221700	-3.56195900
H	-1.82465900	2.05863300	-4.26265400
C	-0.63375300	-0.21413500	-3.08463400
H	0.11686000	-0.39905100	-3.86112900
H	-1.61109200	-0.48234600	-3.50347900
H	-0.41931300	-0.88469000	-2.24797000
C	-1.69026000	3.47926700	1.87811400
H	-0.60224600	3.59450000	1.88334100
C	-2.30851900	4.89256700	1.83117600
H	-3.40396400	4.84647600	1.84299200
H	-2.00205700	5.43746200	0.93365500
H	-1.98874200	5.47445500	2.70349400
C	-2.07924600	2.78443300	3.19851800
H	-1.76001500	3.39660500	4.04967100
H	-1.60554800	1.80326500	3.30175500
H	-3.16382600	2.64741100	3.28055200

PtCY-F_S_0

C	5.04593000	0.31450000	0.59294700
C	4.90266900	3.10960800	0.65685900
C	6.20220400	1.07767800	0.83078700
C	3.80147400	0.91565200	0.39642500
C	3.78306600	2.32441600	0.39630600
C	6.12457600	2.46263500	0.87599500
H	7.14361600	0.55862400	0.98193800
C	-0.02679200	5.07832100	-1.08626500
C	2.78618100	5.28592700	-0.66290700
C	0.71257200	4.02056800	-0.55972500
C	0.66246900	6.25262900	-1.38889700
C	2.04746900	6.34905400	-1.18275100
C	2.09699800	4.11281900	-0.33872100
H	0.12086200	7.09793000	-1.80274300
H	2.56307500	7.26839200	-1.44427200
H	3.85842000	5.36923800	-0.54950400
N	0.33274800	2.71868800	-0.20520400
C	1.42297100	1.98566900	0.19272700
N	2.48369000	2.86320600	0.14933200
Pt	2.03441500	0.00011400	0.37668200
O	5.26963900	-1.03514100	0.57823800
C	4.35714200	-1.97794700	0.15531600
C	2.84910500	-4.08893100	-0.81305900
C	2.96555800	-1.78471300	0.13591200

C	4.98572800	-3.16980300	-0.26089100
C	4.24106000	-4.22226900	-0.76728800
C	2.25932900	-2.90489400	-0.32183300
H	6.06875200	-3.22035700	-0.21171900
H	4.73012300	-5.12324400	-1.12709400
N	0.83801000	-3.03905300	-0.42584000
C	0.56888900	-4.26164500	-1.08844100
C	1.79117200	-4.93481600	-1.32279100
C	-0.62073100	-5.99466200	-2.24090900
C	1.78886900	-6.16080700	-1.99659100
C	-0.64009400	-4.77069900	-1.56834700
C	0.57972100	-6.69070200	-2.44336500
H	-1.57156600	-4.22762000	-1.44917300
H	-1.55256000	-6.40389300	-2.62126200
H	2.72239500	-6.68542300	-2.18166400
H	0.56819600	-7.64141700	-2.96919100
H	4.83927000	4.18739000	0.73560200
H	-1.09128700	4.98042000	-1.26943900
C	-0.05405800	-2.39996600	0.43675100
C	-1.76749000	-1.16503100	2.19664800
C	-1.13044600	-3.11648900	1.00230700
N	0.17545200	-1.10884100	0.76210100
C	-0.67906500	-0.53053500	1.63649700
C	-2.01233900	-2.51796200	1.89212400
H	-1.22046000	-4.16447100	0.76151400
H	-0.44549800	0.49523600	1.88142700
H	-2.40050800	-0.61015600	2.87949800
C	-3.17063800	-3.27490600	2.55687400
C	-3.28591600	-4.72777300	2.05897600
H	-4.13444300	-5.21712400	2.54957200
H	-2.38963100	-5.31336800	2.29163500
H	-3.45618700	-4.77674100	0.97745600
C	-4.49922900	-2.54503100	2.24614400
H	-5.33719700	-3.07698200	2.71184900
H	-4.68372500	-2.49683900	1.16730400
H	-4.50108100	-1.52027500	2.63220500
C	-2.94208400	-3.29685700	4.08773400
H	-3.76549200	-3.82414100	4.58391100
H	-2.89043000	-2.28648800	4.50685500
H	-2.00804800	-3.81176700	4.33872200
H	7.01558800	3.04955800	1.07955100
C	-1.03292100	2.27193300	-0.30808200
C	-3.72425000	1.52807000	-0.56625300
C	-1.44007700	1.56458900	-1.45876900

C	-1.94579900	2.63263700	0.70828100
C	-3.28073200	2.24862500	0.55066600
C	-2.78645700	1.19814200	-1.55291300
H	-4.00459000	2.54690900	1.30339700
H	-3.11272500	0.62718300	-2.41652800
C	-5.15196100	1.14164300	-0.70741100
C	-7.84017700	0.42153300	-0.97180400
C	-5.78587300	1.15597600	-1.96242800
C	-5.91240700	0.75577400	0.41037300
C	-7.25313700	0.39537500	0.28814300
C	-7.12509800	0.79741400	-2.10327900
H	-5.23138700	1.47665100	-2.83920100
H	-5.44243000	0.71394700	1.38828000
H	-7.84021700	0.08864300	1.14757300
H	-7.61978200	0.81624100	-3.06883600
C	-0.48834900	1.24757000	-2.61043700
H	0.53258700	1.43597400	-2.26954700
C	-0.76306700	2.18458700	-3.80602300
H	-0.05091800	1.98488500	-4.61506500
H	-0.66512300	3.23819500	-3.52256900
H	-1.77448600	2.03447400	-4.20367600
C	-0.54287000	-0.22736500	-3.04638300
H	0.19891900	-0.40680300	-3.83250500
H	-1.52345700	-0.50318600	-3.45261200
H	-0.31243900	-0.89590300	-2.21232000
C	-1.54469700	3.45379000	1.93584000
H	-0.45834600	3.58210600	1.92003100
C	-2.18042600	4.85975100	1.90188300
H	-3.27488900	4.80093400	1.93358700
H	-1.89666100	5.40928700	0.99975900
H	-1.85170200	5.44430200	2.76902700
C	-1.89954000	2.75364100	3.26294300
H	-1.56871400	3.36798900	4.10803600
H	-1.41418200	1.77708200	3.35496300
H	-2.98082500	2.60621300	3.36747700
F	-9.13690400	0.07358800	-1.09949600

PtCY-*t*Bu_S$_0$

C	4.23106500	-1.26903900	0.44143100
C	4.95048000	1.41352300	0.30773000
C	5.57121500	-0.88056000	0.61953400
C	3.22007200	-0.34166100	0.20755500
C	3.64012700	0.99787300	0.10747000
C	5.95243700	0.45777600	0.57160800

H	6.29040200	-1.67039500	0.79950800
C	0.81918400	4.74112800	-1.42883500
C	3.57216000	4.06217000	-1.12106600
C	1.21443000	3.52217100	-0.88027100
C	1.82651700	5.62488500	-1.81587800
C	3.18007800	5.28409600	-1.66806100
C	2.56634400	3.18099100	-0.71015700
H	1.55817000	6.58341900	-2.24989400
H	3.94481900	5.98183800	-1.99639200
H	4.62042800	3.80545900	-1.05232200
N	0.46246000	2.42153000	-0.44774800
C	1.28385200	1.39760900	-0.04501900
N	2.56331900	1.89570300	-0.16601800
Pt	1.25996500	-0.67398200	0.24262500
O	4.03914900	-2.62201200	0.52326800
C	2.88160800	-3.26779800	0.14444100
C	0.80476800	-4.87654800	-0.72956400
C	1.61168200	-2.66632500	0.10183200
C	3.12060500	-4.61382000	-0.20216300
C	2.09223900	-5.41938900	-0.66232100
C	0.59988700	-3.54667000	-0.30366800
H	4.13900100	-4.98376900	-0.13923500
H	2.28650500	-6.44265600	-0.97161900
N	-0.79589100	-3.25458800	-0.41541300
C	-1.42228700	-4.36956100	-1.02371100
C	-0.45956500	-5.38935700	-1.21155900
C	-3.07865600	-5.71670400	-2.11421100
C	-0.83096200	-6.58811800	-1.82974200
C	-2.72879800	-4.51388900	-1.49660400
C	-2.14359300	-6.75031800	-2.26907800
H	-3.45383000	-3.71117900	-1.41517000
H	-4.09052600	-5.84388100	-2.48900200
H	-0.09841100	-7.37688000	-1.97872900
H	-2.44097600	-7.67693000	-2.75227200
H	5.20120200	2.46597000	0.30542200
H	-0.23037500	4.98042000	-1.56217400
C	-1.45469900	-2.32851100	0.39389800
C	-2.74271600	-0.52705100	2.02261200
C	-2.70064300	-2.65538200	0.97128800
N	-0.84929400	-1.14895200	0.65539600
C	-1.50346500	-0.28747800	1.46740500
C	-3.37668900	-1.76334200	1.79276600
H	-3.09503000	-3.64348500	0.79175100
H	-0.97759900	0.63492400	1.66268800

H	-3.19223200	0.24011900	2.64258000
C	-4.72753600	-2.08842300	2.44495700
C	-5.21072400	-3.51337200	2.11664400
H	-6.17796700	-3.69285200	2.59891800
H	-4.51345500	-4.27611300	2.48108600
H	-5.34812600	-3.66140900	1.03958200
C	-5.78387400	-1.08157100	1.92984600
H	-6.75500000	-1.28278000	2.39760700
H	-5.90724300	-1.15892900	0.84389000
H	-5.50944200	-0.04690400	2.15886300
C	-4.59761400	-1.95902400	3.98165000
H	-5.55851200	-2.18364500	4.45948600
H	-4.30286000	-0.94913100	4.28502300
H	-3.85116200	-2.65882200	4.37381600
C	-0.97752700	2.45665900	-0.43368100
C	-3.77260200	2.68270400	-0.46929100
C	-1.69514900	1.89264200	-1.50873700
C	-1.62505100	3.14101600	0.61924500
C	-3.01865800	3.23842700	0.57279300
C	-3.08857400	2.01232500	-1.49205900
H	-3.53091400	3.79253400	1.35363400
H	-3.66111300	1.56047200	-2.29566900
C	-5.25272600	2.81631700	-0.49594000
C	-8.06216400	3.07918400	-0.54809800
C	-5.94063000	2.98978300	-1.70936000
C	-6.00531500	2.78079100	0.69092400
C	-7.39354800	2.91050200	0.66599100
C	-7.32880100	3.11869500	-1.73564300
H	-5.37981400	3.05100700	-2.63760900
H	-5.49952500	2.62940800	1.64049100
H	-7.95390200	2.87269100	1.59645700
H	-7.83715700	3.25985600	-2.68577000
H	-9.14377000	3.18029500	-0.56838400
C	-1.00243500	1.22081000	-2.69248600
H	0.03589200	1.02572100	-2.41125500
C	-0.98946700	2.16511200	-3.91373700
H	-0.45603000	1.69711700	-4.74928600
H	-0.49231900	3.11393800	-3.68518700
H	-2.00962200	2.39010500	-4.24905700
C	-1.62497700	-0.13721600	-3.06292600
H	-1.03863300	-0.60560300	-3.86115400
H	-2.65323300	-0.03453700	-3.43018800
H	-1.63025000	-0.81972400	-2.20840100
C	-0.86932300	3.79662400	1.77722500

H	0.19355100	3.56297200	1.66710500
C	-1.01210500	5.33239900	1.74638100
H	-2.05770400	5.63840500	1.87038400
H	-0.64640100	5.75110600	0.80411300
H	-0.43346100	5.78206500	2.56162600
C	-1.31314700	3.25253800	3.15031000
H	-0.72998300	3.72841800	3.94694000
H	-1.16161700	2.17123100	3.23123900
H	-2.37132300	3.46252400	3.34601200
C	7.40450700	0.91907100	0.79263900
C	8.35896900	-0.26171100	1.05452100
H	9.37755400	0.11322000	1.20821500
H	8.38580300	-0.95780100	0.20864200
H	8.07409500	-0.82452100	1.95050400
C	7.46549400	1.86605400	2.01505000
H	8.49463400	2.20689900	2.18473100
H	7.12148700	1.35546600	2.92168400
H	6.83976500	2.75423400	1.87520700
C	7.90803600	1.67141000	-0.46216300
H	8.94442900	2.00300300	-0.32093300
H	7.30245700	2.55912700	-0.67633700
H	7.87430200	1.02415600	-1.34587300

PtCY-*t*BuF_S_0

C	4.45400200	-1.04163300	0.36990400
C	5.00683900	1.68324700	0.30078700
C	5.77045900	-0.57631700	0.54098600
C	3.38529800	-0.17178100	0.17545300
C	3.72165700	1.19337200	0.10613300
C	6.06868600	0.78367300	0.52568900
H	6.53932800	-1.32470800	0.68984400
C	0.65649400	4.77795400	-1.33780300
C	3.44915600	4.27344900	-1.05367300
C	1.13240800	3.57897700	-0.80973800
C	1.60254100	5.73102100	-1.71501600
C	2.97608000	5.47637900	-1.57828600
C	2.50469200	3.32254900	-0.65299700
H	1.27048200	6.67673100	-2.13278200
H	3.69234900	6.22740700	-1.89820800
H	4.51216300	4.08381100	-0.99408700
N	0.45568600	2.42536100	-0.39073200
C	1.34440700	1.44978900	-0.01165600
N	2.58836600	2.03020800	-0.13204600
Pt	1.44994500	-0.62270700	0.23526500

O	4.34618300	-2.40547700	0.41684100
C	3.22056100	-3.11210600	0.05080200
C	1.22259900	-4.82727100	-0.80556600
C	1.91613100	-2.58785900	0.04902600
C	3.53111200	-4.43469400	-0.32807000
C	2.54169100	-5.29237700	-0.77931000
C	0.94909200	-3.51991700	-0.35008200
H	4.57096200	-4.74391600	-0.29588700
H	2.78884900	-6.29667900	-1.11205500
N	-0.46442700	-3.31108900	-0.42154500
C	-1.03840200	-4.45274500	-1.03215900
C	-0.02092100	-5.40830200	-1.26381700
C	-2.64002000	-5.88216400	-2.09910400
C	-0.33581600	-6.61768900	-1.89253100
C	-2.34674300	-4.66977200	-1.47086500
C	-1.64821600	-6.85353100	-2.29793400
H	-3.11669200	-3.91443200	-1.35533500
H	-3.65258100	-6.06578300	-2.44767200
H	0.43898800	-7.35747400	-2.07516800
H	-1.90216600	-7.78905500	-2.78864600
H	5.19258700	2.74887400	0.32374600
H	-0.40692300	4.95032100	-1.46392900
C	-1.15520000	-2.44025200	0.42174700
C	-2.49670800	-0.75112100	2.12583200
C	-2.35935200	-2.85368400	1.03135700
N	-0.61624700	-1.22923500	0.68501200
C	-1.29543900	-0.42380600	1.53329300
C	-3.05990800	-2.02081300	1.89326300
H	-2.69797400	-3.86109900	0.84521000
H	-0.82170500	0.52688800	1.72703900
H	-2.97031200	-0.02683700	2.77873100
C	-4.36003500	-2.44440300	2.59069100
C	-4.78062900	-3.88081300	2.22817400
H	-5.71436600	-4.13037400	2.74390200
H	-4.02826600	-4.61661200	2.53340900
H	-4.95715600	-3.99684600	1.15287500
C	-5.49318800	-1.48010800	2.16548000
H	-6.42996700	-1.75791400	2.66300900
H	-5.65863700	-1.51656400	1.08300700
H	-5.26791300	-0.44252600	2.43279200
C	-4.16434500	-2.36484800	4.12392700
H	-5.08881700	-2.65740300	4.63573000
H	-3.90733800	-1.35253100	4.45277300
H	-3.36533500	-3.03803400	4.45403800

C	-0.98298100	2.35374400	-0.39243300
C	-3.78637800	2.36569800	-0.46020300
C	-1.64329000	1.75471800	-1.48546600
C	-1.69311900	2.96839100	0.66329100
C	-3.08959300	2.96093900	0.59975300
C	-3.04180300	1.76611400	-1.48428400
H	-3.65225400	3.46187700	1.38208300
H	-3.56821100	1.28361200	-2.30162500
C	-5.27143000	2.38523200	-0.50383500
C	-8.06569500	2.43109100	-0.58694500
C	-5.95807300	2.53465400	-1.72180100
C	-6.03407000	2.26240400	0.67100700
C	-7.42717900	2.28453400	0.63924300
C	-7.35045000	2.55717000	-1.77242800
H	-5.39547900	2.66059900	-2.64194600
H	-5.53117000	2.12559800	1.62366800
H	-8.01832000	2.18118300	1.54324600
H	-7.88288600	2.68160000	-2.70965800
C	-0.89019600	1.15829600	-2.67282300
H	0.15930900	1.04801000	-2.38805700
C	-0.95158900	2.11522600	-3.88265500
H	-0.37300900	1.70622900	-4.71905600
H	-0.54118900	3.10104600	-3.63874700
H	-1.98483800	2.25432500	-4.22434700
C	-1.39358300	-0.24321200	-3.06123100
H	-0.77064700	-0.64813900	-3.86639900
H	-2.42770400	-0.22476000	-3.42587900
H	-1.33696400	-0.93487900	-2.21609900
C	-1.00233700	3.66274700	1.83919000
H	0.07475800	3.49284700	1.75027900
C	-1.23600800	5.18754400	1.80934500
H	-2.30084400	5.42935400	1.90938500
H	-0.87391700	5.63156400	0.87754200
H	-0.70477000	5.66760900	2.63940100
C	-1.44038600	3.09079300	3.20265300
H	-0.89623000	3.59390500	4.01001800
H	-1.23458200	2.01866500	3.28371600
H	-2.51093700	3.24524700	3.38199700
C	7.49354200	1.32632100	0.74043400
C	8.51851200	0.19986900	0.97361500
H	9.51442700	0.63190700	1.12568200
H	8.57865100	-0.47862100	0.11530000
H	8.27494400	-0.39417800	1.86157800
C	7.51194000	2.25563600	1.97780400

H	8.52222300	2.64929600	2.14631600
H	7.20257500	1.71307600	2.87829600
H	6.83845900	3.11086300	1.85622800
C	7.93703800	2.12782500	-0.50655200
H	8.95020500	2.52557900	-0.36767600
H	7.27120400	2.97553400	-0.70241800
H	7.93928200	1.49241100	-1.39945200
F	-9.41351000	2.45253900	-0.62707200
